# A general strategy towards multicolour fluorogenic peptides for wash-free bioassays

**DOI:** 10.1038/s41557-026-02228-0

**Published:** 2026-08-26

**Authors:** Man Sing Wong, Lorena Mendive-Tapia, Utsa Karmakar, Lovelesh Vashist, Zandile Nare, Karolina Tokarczyk, Kohei Iijima, Kazuya Kikuchi, Syed Ali Abbas Abedi, Xiaogang Liu, Marc Vendrell

**Affiliations:** 1 Centre for Inflammation Research, Institute for Regeneration and Repair, The University of Edinburgh, Edinburgh, UK; 2 IRR Chemistry Hub, Institute for Regeneration and Repair, The University of Edinburgh, Edinburgh, UK; 3 Fluorescence Research Group, Singapore University of Technology and Design, Singapore, Singapore; 4 Concept Life Sciences Ltd, Edinburgh Bioquarter, Edinburgh, UK; 5 Department of Applied Chemistry, Graduate School of Engineering, The University of Osaka, Suita, Japan; 6 School of Chemistry, Chemical Engineering and Biotechnology, Nanyang Technological University, Singapore, Singapore

**Keywords:** Peptides, Imaging, Fluorescent probes

## Abstract

Peptide-based fluorescent probes are valuable tools for live-cell imaging, but conventional labelling approaches often require washing or long incubation, limiting their signal-to-noise ratios. Fluorogenic amino acids (FgAAs) can overcome these limitations, yet their rational design remains challenging owing to the lack of generalizable quenching principles and synthetic routes compatible with diverse fluorophores. Here we developed a general strategy to convert constitutively bright fluorophores into turn-on FgAAs through intramolecular tryptophan-induced quenching. Computational analysis showed that quenching is governed by photoinduced electron transfer or twisted intramolecular charge transfer mechanisms, with adaptability across ultraviolet-to-near-infrared fluorophore scaffolds. We synthesized a large library of multicolour FgAAs and integrated them into solid-phase peptide synthesis to generate turn-on probes for real-time microscopy in live cells. We then designed fluorogenic peptides targeting programmed cell death protein 1 (PD-1), enabling T cell targeted drug screening and identifying avasimibe as a small-molecule modulator of PD-1 activity. This work establishes a rational strategy for designing multicolour FgAAs for peptide engineering, imaging and drug discovery.

**Figure:**
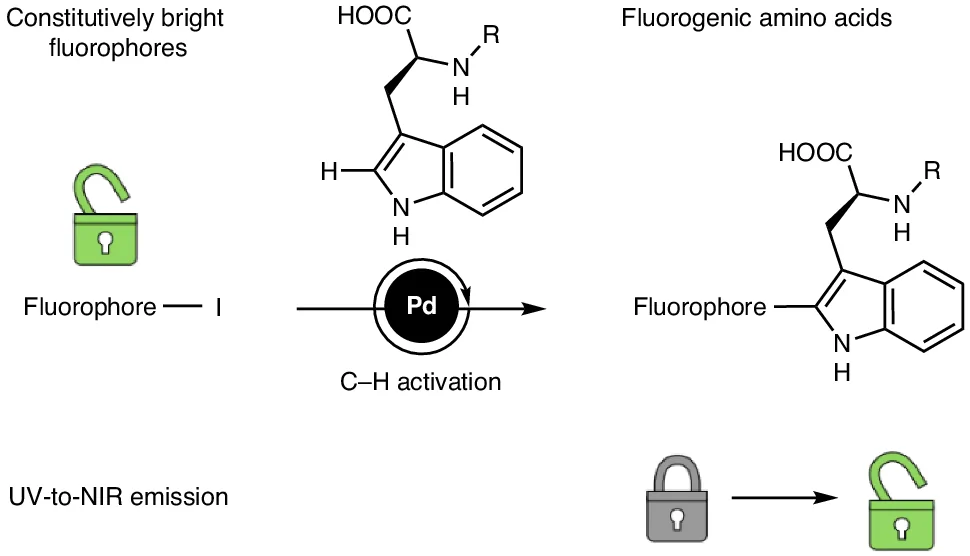

## Abstract (web summary)

The rational design of fluorogenic amino acids for peptide-based fluorescence imaging is challenging. Now a strategy has been developed to prepare fluorogenic amino acids with adaptability across UV-to-NIR fluorophore scaffolds. The integration of these amino acids into solid-phase peptide synthesis generates fluorogenic peptide probes for real-time microscopy and flow cytometry in live cells.

## Main

Fluorescence imaging constitutes an indispensable tool to study the biological functions of live cells, providing molecular insights with high spatiotemporal resolution^1–8^. Peptide-based fluorescent probes stand out within the chemical toolbox because of their structural modularity and target specificity^9,10^; however, most approaches to label peptide sequences rely on the coupling of commercial fluorophores to terminal groups or functionalized side chains^11–14^. These labelling strategies render probes with limited signal-to-noise ratios (SNRs), either because they require washing steps to remove excess ligands or long incubation times to ensure sufficient binding and contrast^15,16^. Alternatively, fluorogenic dyes that turn on in response to target recognition enable wash-free cell imaging with good SNRs^17,18^, but the rational design of turn-on peptides is limited by the lack of strategies to generate multicolour fluorogenic building blocks compatible with wash-free bioimaging assays.

Fluorogenic amino acids (FgAAs) are versatile building blocks to prepare peptide and protein-based probes for live-cell fluorescence imaging^19–21^. Current synthetic approaches for FgAAs typically involve the coupling of pre-formed fluorogens to amino acid cores via amide formation, nucleophilic substitution or cross-coupling reactions^22–26^; however, pre-formed fluorogenic dyes (for example, dansyl, nitrobenzodiazole, aminonaphthalimide and prodan) exhibit suboptimal properties for fluorescence-based cell experiments (for example, short excitation/emission wavelengths and limited brightness). Multicolour FgAAs based on new structures remain underexplored owing to the lack of (1) intramolecular quenching moieties with broad chemical applicability to different fluorescent cores and (2) systematic tools to predict fluorogenicity mechanisms. We envisaged that a universal strategy to convert constitutively bright fluorophores into FgAAs would accelerate the development of targeted peptides for real-time imaging and drug screening.

In the last decade, drug screening platforms have dedicated numerous efforts to the discovery of immunotherapy small molecules that modulate T cell function. The biological activity of T cells is tightly regulated by the expression of cell-surface receptors^27,28^, such as programmed cell death protein 1 (PD-1), which acts as an immune checkpoint receptor on the surface of active T cells^29^. The dysregulation of PD-1 expression in T cells is directly related to pathological conditions that evade the immune system (for example, cancer) or that lead to autoimmune diseases (for example, rheumatoid arthritis and multiple sclerosis)^30^. In this context, the development of immunotherapies would benefit from fluorescence screenings to accelerate the discovery of pharmacological structures targeting the PD-1 pathway.

In this study, we describe a general strategy for developing multicolour FgAAs directly from bright fluorophores via a common intramolecular tryptophan (Trp)-induced quenching mechanism. Computational analysis using density functional theory (DFT) and time-dependent DFT (TD-DFT) showed that Trp-induced quenching is governed by processes activated by the local environment through photoinduced electron transfer (PeT) or twisted intramolecular charge transfer (TICT). This strategy enabled us to generate several ultraviolet-to-near-infrared (UV-to-NIR) FgAAs by directly coupling Trp quenchers to chemically diverse iodinated fluorophores with variable optical signatures. In addition, we demonstrated that FgAAs behave as highly effective turn-on switches for the solid-phase synthesis of fluorogenic peptides, wash-free cell imaging and fluorescence-based drug screenings. This chemical strategy will accelerate the development of new fluorogens by leveraging the optical and physicochemical properties of bright fluorophores to facilitate their integration into peptides and proteins for real-time targeted bioimaging studies.

## Results

### Design and synthesis of a multicolour library of internally quenched FgAAs

The synthesis of FgAAs has been restricted, so far, to the derivatization of environmentally sensitive scaffolds with amino acid moieties that enable integration into peptides and proteins. These approaches have rendered encodable amino acids (for example, CouA, ANAP and dansylalanine) to produce recombinant fluorescent proteins^31–33^, tetrahydroquinoline rotor fluorogenic d-amino acids for imaging transpeptidation reactions in bacterial cells^20^ or small nitrobenzodiazoles for super-resolution microscopy^34^. While useful, the broad application of these amino acids is hampered by the optical properties of fluorogenic cores. For instance, CouA, ANAP and dansylalanine display short excitation wavelengths (that is, <400 nm) that overlap with cellular autofluorescence, whereas tetrahydroquinolines and nitrobenzodiazoles exhibit moderate-to-low extinction coefficients (that is, in the 10^3^–10^4^ M^−1^ cm^−1^ range). We envisaged that the preparation of FgAAs by direct conversion of fluorescent structures would expand not only the chemical diversity of the FgAA toolbox but also their optical properties and range of applications in fluorescence-based imaging.

**Fig. 1:**
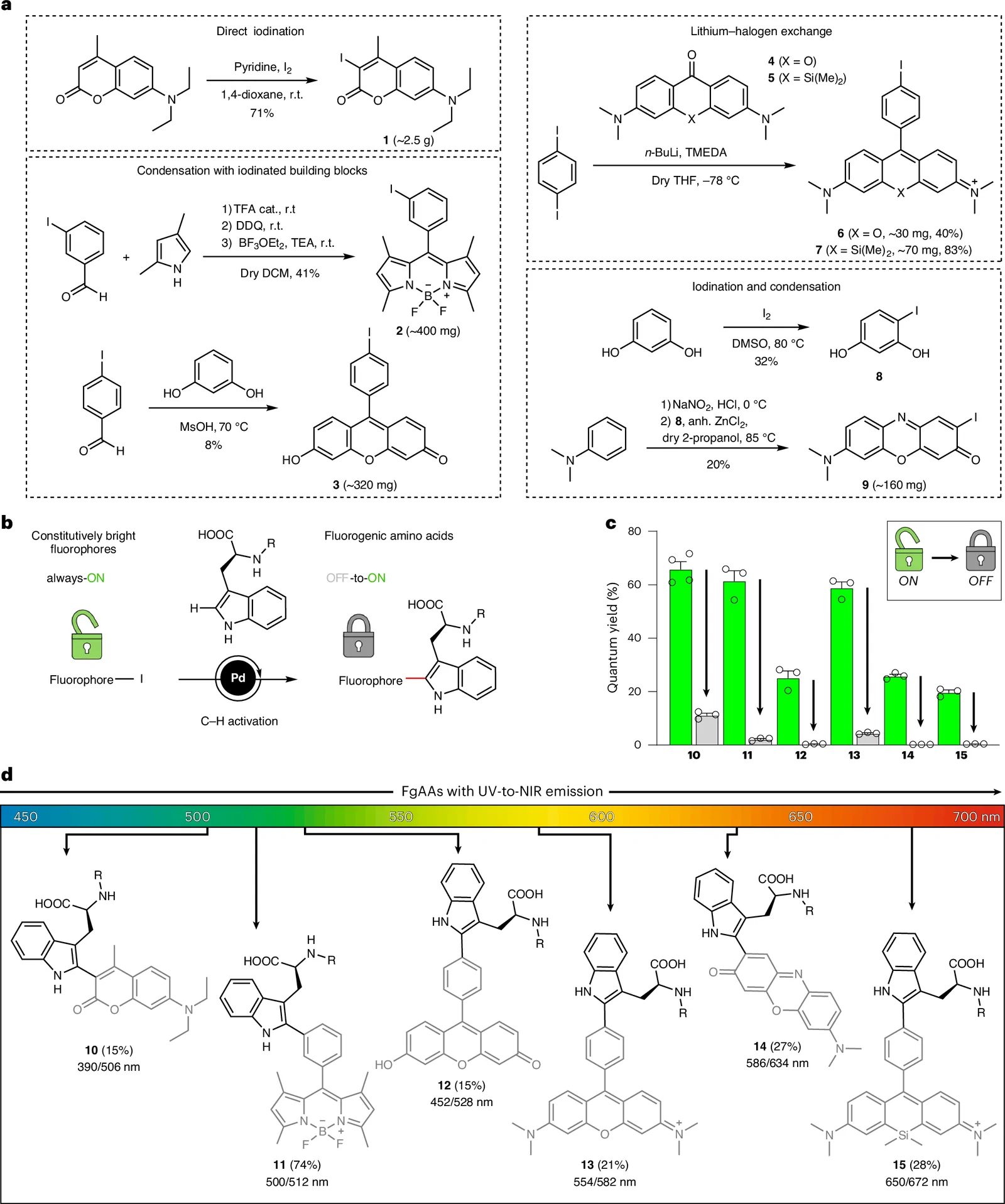
Design, synthesis and characterization of a chemically and optically diverse multicolour library of FgAAs. **a**, Synthetic routes for the preparation of iodinated precursors of coumarin (**1**), BODIPY (**2**), HPF (**3**), O-rhodamine (**6**), Si-rhodamine (**7**) and oxazone (**9**). cat, catalytic. **b**, The Pd-catalysed C–H activation between iodinated fluorophores and Fmoc-protected Trp. **c**, Fluorescence quantum yields of FgAAs (grey columns) and their corresponding constitutive fluorophores (green columns) under the same experimental conditions. Fluorescence quantum yields were determined in EtOH and are presented as means ± s.e.m. (*n* = 3 experimental replicates). **d**, The chemical structures of Trp-quenched FgAAs **10**–**15** and their respective excitation and emission maxima wavelengths (exc/em) across the UV-to-NIR spectrum. The chemical scaffolds of common fluorophores are highlighted in bold grey lines. R, Fmoc; r.t., room temperature; anh., anhydrous. Source data

Our group and others have reported the fine-tuning and optical modulation of organic fluorophores using a wide range of intramolecular quenching mechanisms^35–40^. Among others, these include the incorporation of metal ion chelators (for example, Cu^2+^ and Sn^2+^) to generate ion-sensitive fluorophores^41,42^, spirocyclization mechanisms to modulate pH sensitivity and blinking^43,44^, or the coupling of chemical groups to induce non-radiative energy transfer and charge separation processes (for example, Förster resonance energy transfer (FRET), PeT and TICT)^45–48^. In all these cases, the extent of fluorogenicity is optimized in a fluorophore-dependent manner, with very few quenching approaches being broadly applicable to multiple fluorophores. In proteins, fluorescent labels can be quenched by nearby endogenous Trp residues upon van der Waals contact to probe folding dynamics^49–54^. Similarly, other studies have shown that dyes can be quenched to varying degrees when mixed with Trp or covalently attached to its aliphatic amino group, probably due to complex formation upon close contact^55,56^. Inspired by these studies, we explored the direct intramolecular quenching of the Trp core as a versatile synthetic strategy to produce FgAAs from constitutively bright fluorophores. To test this hypothesis, we designed the synthesis of Trp-quenched FgAAs by coupling a Trp amino acid to several iodinated fluorophores via C–H activation. This methodology enabled the formation of C–C bonds between the different fluorophores and the position 2 of Trp, thus rendering compact FgAA building blocks with potential for enhanced quenching owing to short fluorophore–Trp distances. First, we prepared six iodinated fluorophores covering a range of chemical structures and excitation/emission profiles (that is, coumarin, fluorone, BODIPY, oxazone, O-rhodamine and Si-rhodamine) (Fig. 1a; see the Supplementary Information for full synthetic details). Notably, the synthesis of iodinated precursors was approachable via different synthetic routes. For coumarin (**1**), we employed direct iodination of the commercial 7-diethylamino-4-methylcoumarin with iodine to give compound **1** in 71% yield. BODIPY (**2**) and 6-hydroxy-9-phenyl-fluorone (HPF, **3**) compounds were prepared from commercial iodinated building blocks (for example, 3-iodobenzaldehyde in the case of BODIPY and 4-iodobenzaldehyde in the case of HPF). Specifically, the BODIPY compound **2** was synthesized in a conventional one-pot procedure through acid-catalysed condensation of *m*-iodobenzaldehyde with two units of 2,4-dimethylpyrrole, followed by 2,3-dichloro-5,6-dicyano-p-benzoquinone (DDQ) oxidation and BF_3_ complexation to give the BODIPY product **2** in 41% overall yield^57^. In the case of the HPF compound **3**, the iodinated precursor was formed by condensation of 4-iodobenzaldehyde with resorcinol in acidic media and isolated in good purity but very low (that is, under 10%) yield. For O-rhodamine (**6**) and Si-rhodamine (**7**), we performed lithium–halogen exchange reactions between 1,4-diiodobenzene and the xanthone precursors **4** and **5**, respectively (Fig. 1a). In these cases, we optimized the reaction conditions by using excess 1,4-diiodobenzene and *n*-BuLi to favour the incorporation of a single xanthone unit and minimize non-iodinated side products. These conditions afforded iodinated rhodamines **6** and **7** in 40% and 83% isolated yields, respectively. Finally, the oxazone compound **9** was prepared using a combined approach following the iodination of resorcinol and subsequent acid-catalysed condensation with the *N*,*N*-dimethyl-*p*-nitrosoaniline intermediate, which had been obtained by a reaction between *N*,*N*-dimethylaniline and sodium nitrite. Compound **9** was isolated in overall yields around 20%. Once all iodinated fluorophores were obtained, we coupled them to Fmoc-protected Trp using standard Pd-catalysed C(*sp*^2^) C–H activation conditions (for example, Pd(OAc)_2_, AgBF_4_ and TFA)^58^ to successfully obtain the FgAAs **10**–**15** (Fig. 1b) with recovery yields from 15% to 74% and purities >95% (see the Supplementary Information for full synthetic and characterization details). These results confirmed that our synthetic strategy could be in principle applicable to a broad range of fluorescent structures by simply accessing their aryl iodinated precursors.

Next, we evaluated the spectral properties of all FgAAs (**10**–**15**) and compared them with the exact parent fluorophore structures: coumarin (**16**), BODIPY (**17**), HPF (**18**), O-rhodamine (**19**), oxazone (**20**) and Si-rhodamine (**21**) (Fig. 1c, Table 1 and Supplementary Figs. 1–7). Notably, all FgAAs exhibited low fluorescence quantum yields (that is, averaging 1–2%), showing at least one order of magnitude lower values than their constitutively bright fluorophores. These results confirmed the strong intramolecular quenching effect of Trp across multiple fluorescent structures. In most FgAAs (that is, compounds **11**, **12**, **13** and **15**) the direct linkage to Trp did not affect their absorption and emission profiles. By contrast, coumarin and oxazone amino acids (that is, compounds **10** and **14**, respectively) showed slight bathochromic shifts in peak absorption and emission wavelengths (coumarin: 16 nm in excitation, 56 nm in emission; oxazone: 28 nm in excitation, 10 nm in emission) due to the extension of their *π*-conjugated systems. Importantly, molar extinction coefficients remained unaffected in the FgAAs, with values reaching 10^5^ M^−1^ cm^−1^ for O-rhodamine-AA (where ‘AA’ is amino acid) and Si-rhodamine-AA (Table 1). These results indicate the suitability of our strategy to directly produce highly fluorogenic amino acids from common fluorophores without compromising their optical signatures.

**Table 1:** Spectral properties of FgAAs and their corresponding parent fluorophores.^a^

Compounds	*λ*_abs_ (nm)	*λ*_em_ (nm)	*ε* (× 10^3^) (M^−1^ cm^−1^)	QY (%)	Brightness (M^−1^ cm^−1^)
Coumarin-AA (**10**)	390	506	26	11 ± 1	2,900
Coumarin (**16**)	374	450	22	66 ± 3	15,000
BODIPY-AA (**11**)	500	512	98	2.2 ± 0.3	2,200
BODIPY (**17**)	500	510	114	62 ± 4	71,000
HPF-AA (**12**)	452	528	20	<1	n.d.
HPF (**18**)	454	516	21	25 ± 2	5,300
O-rhodamine-AA (**13**)	554	582	96	4.4 ± 0.2	4,200
O-rhodamine (**19**)	552	578	120	59 ± 2	71,000
Oxazone-AA (**14**)	586	634	34	<1	n.d.
Oxazone (**20**)	558	624	36	26 ± 1	9,400
Si-rhodamine-AA (**15**)	650	672	148	<1	n.d.
Si-rhodamine (**21**)	650	670	127	20 ± 1	25,000

^a^Values determined in EtOH (concentrations 2–100 µM, *n* = 3). FgAAs were analysed as Fmoc-protected amino acids. Experimental conditions for FgAAs and their corresponding fluorophores were kept the same for direct comparison. *λ*_abs_, peak absorption wavelength; λ_em_, peak emission wavelength; *ε*, molar extinction coefficients at *λ*_abs_; n.d., not detected; QY, fluorescence quantum yields, measured using quinine sulfate (0.05 M H_2_SO_4_), fluorescein (0.1 M NaOH), coumarin 153 (EtOH), rhodamine B (EtOH), cresyl violet (MeOH) and 3,3′-diethylthiadicarbocyanine iodide (EtOH) as references, respectively.

### Computational analysis of the turn-on mechanisms in FgAAs

**Fig. 2:**
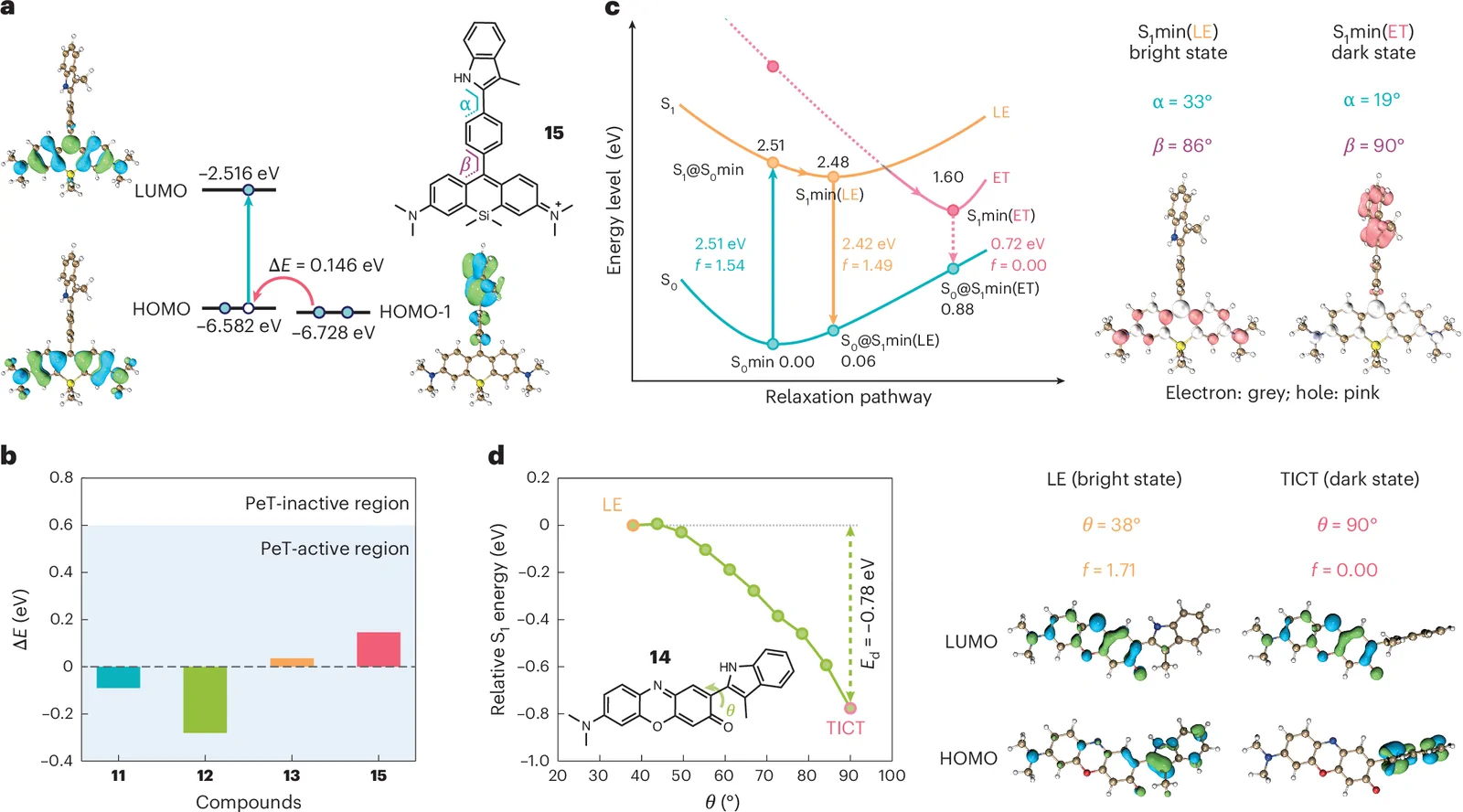
Computational analysis of frontier molecular orbitals and excited-state properties of representative FgAAs exhibiting PeT- or TICT-based quenching. **a**, The electron density and energy levels of HOMO-1, HOMO and LUMO for Si-rhodamine-AA (**15**) in water, calculated, on the basis of the optimized ground-state geometry using M06-2X/def2-SVP with the solvation model based on density (SMD) (water). Inset: the molecular structure of Si-rhodamine-AA (**15**), the energy gap (Δ*E*) between HOMO and HOMO-1, and key dihedral angles *α* and *β*. **b**, The calculated Δ*E* for FgAAs **11**, **12**, **13** and **15**, with small Δ*E* values indicating the PeT mechanism. **c**, The calculated excitation/de-excitation energy (including vertical oscillator strength *f*) of Si-rhodamine-AA (**15**) in water. Right: optimized molecular structures, the corresponding electron and hole distributions in the LE and ET states, and their dihedral angles *α* and *β*. For clarity, the relative energy levels are not depicted to scale. **d**, The S_1_ potential energy surface of oxazone-AA (**14**) as a function of the rotational angle (*θ*) towards the formation of the TICT state in water. Calculations were performed using M06-2X with def2-SVP geometry optimization and single-point energies at the def2-TZVP level, including cLR solvent-effect correction within the SMD solvation model (water). Inset: the molecular structure of oxazone-AA (**14**) and highlights *θ*. *E*_d_ represents the driving energy to enter the TICT state. Right: the electron density of HOMO and LUMO in the LE and TICT states, their corresponding dihedral angles *θ* and vertical oscillator strength *f* for oxazone-AA (**14**) in water. Source data

To understand the quenching mechanisms behind the fluorogenicity of FgAAs, we conducted computational studies using DFT and TD-DFT. We analysed the frontier molecular orbitals of all six amino acids (**10**–**15**) on the basis of their ground-state optimized geometries (Supplementary Figs. 8–13). As shown in Fig. 2a with the representative Si-rhodamine-AA (**15**), the Trp moiety introduced an additional filled molecular orbital (HOMO-1) slightly below the highest occupied molecular orbital (HOMO), separated by a small energy gap (Δ*E*) of 0.146 eV. Both the HOMO and the lowest unoccupied molecular orbital (LUMO) were localized on the fluorophore scaffold so that, upon photoexcitation, an electron transitions from the HOMO to the LUMO while Trp (HOMO-1) injects an electron to Si-rhodamine (HOMO). This PeT is facilitated by the small Δ*E* gap in polar solvents, which stabilizes the resulting electron transfer state and quenches fluorescence. Because the Trp moiety remains isolated from the *π*-conjugation of the Si-rhodamine core, the intramolecular quenching modulates the fluorescence while preserving peak absorption wavelengths (*λ*_abs_) and emission wavelengths (*λ*_em_) of the parent fluorophore (Table 1). We found that the same PeT quenching mechanism was applicable to the other three FgAAs, namely BODIPY-AA (**11**), HPF-AA (**12**) and O-rhodamine-AA (**13**) (Supplementary Figs. 9–11). In all these cases, the frontier molecular orbitals of Trp were energetically close to those of the different fluorophores with very small Δ*E* gaps (for example, −0.091 eV (**11**), −0.280 eV (**12**) and 0.013 eV (**13**)) (Fig. 2b; Supplementary Information).

Next, we performed excited-state calculations to obtain deeper insights into the photophysical mechanisms (Fig. 2c and Supplementary Figs. 14–18; Supplementary Information). The analysis of S_1_ potential energy surfaces for Si-rhodamine-AA (**15**) in hydrophilic environments showed two local minima: (1) a bright locally excited (LE) state, where the electron and hole remained confined to the fluorophore (oscillator strength *f* = 1.49) and (2) a dark electron transfer (ET) state (*f* ≈ 0), characterized by an electron transfer from Trp to the fluorophore (Fig. 2c). The ET state was 0.88 eV more energetically stable than the LE state. This stabilization arises from strong electrostatic interactions with water molecules, which also cause substantial fluorescence quenching. In addition, during the LE-to-ET state transition, the Trp underwent a considerable rotation of ~14° relative to the attached phenyl ring, driven by enhanced conjugation upon ionization. The meso-phenyl ring also rotated by ~4° with respect to the Si-rhodamine core, highlighting conformational changes during the PeT process. On the contrary, hydrophobic environments caused weaker solvent interactions that destabilized the ET state, which enabled Si-rhodamine-AA (**15**) to remain in its LE state with bright emission^59,60^ (Fig. 2 and Supplementary Fig. 18). Furthermore, the restriction of rotational freedom of the FgAA also suppressed the transition to the ET state with concomitant fluorescence turn-on. The excited-state calculations on the other PeT-quenched FgAAs (that is, compounds **11**, **12** and **13**) (Supplementary Figs. 14–17) supported the uniform dual sensitivity of the PeT process to environmental polarity and steric constraints that underpin the strong fluorogenic behaviour of Trp-quenched FgAAs.

We also analysed the absorption and emission shifts of coumarin-AA (**10**) and oxazone-AA (**14**) and determined their respective frontier molecular orbitals (Supplementary Figs. 8 and 12). In these cases, the Trp moiety showed substantial contributions to the HOMO, resulting in extended *π*-conjugation and bathochromic shifts in their absorption and emission spectra (Table 1). In oxazone-AA (**14**), ground-state optimized geometries indicated that the Trp moiety adopted a pre-twisted conformation relative to the oxazone core with an average dihedral angle of ~42°, suggesting that the fluorogenicity was primarily governed by TICT (Fig. 2d and Supplementary Fig. 12). Such geometry promoted TICT by enabling the Trp and oxazone fragments to rotate towards a perpendicular alignment, resulting in a charge-separated, non-emissive dark state. As shown in Fig. 2d, the potential energy surface scan of the S_1_ state as a function of the dihedral angle rotation (*θ*) in oxazone-AA (**14**) reveals an almost barrierless transition to the TICT state, with a strong driving energy of 0.78 eV in magnitude. Analogously, calculations on coumarin-AA (**10**) showed TICT as a key contributor to its fluorogenic behaviour (Supplementary Fig. 8; Supplementary Information). Similar to the PeT-based FgAAs (**11**, **12**, **13** and **15**), TICT is influenced by both polarity and steric hindrance^59,61^, therefore triggering substantial emission in hydrophobic or viscous environments over hydrophilic environments.

Altogether, these computational results indicate that the quenching in Trp-based FgAAs is governed by environmental and structural factors that modulate the activation of PeT or TICT mechanisms. By replicating the quenching effect observed in endogenous Trp residues in proteins, we demonstrated that Trp can serve as a universal intramolecular quencher where the electronic isolation or conjugation within the fluorophore cores dictates the dominant quenching pathway (for example, PeT for electronically isolated fluorophores and TICT for conjugated fluorophores). Finally, building on these computational analyses, our data indicate that such intramolecular quenching mechanisms could be in principle replicated in other commercial fluorophores (for example, cyanines and carbopyronines) (Supplementary Figs. 19–24), thus representing a general approach to prepare FgAAs from a wide range of organic fluorophores.

### FgAAs are efficient turn-on building blocks for peptide synthesis and wash-free imaging

**Fig. 3:**
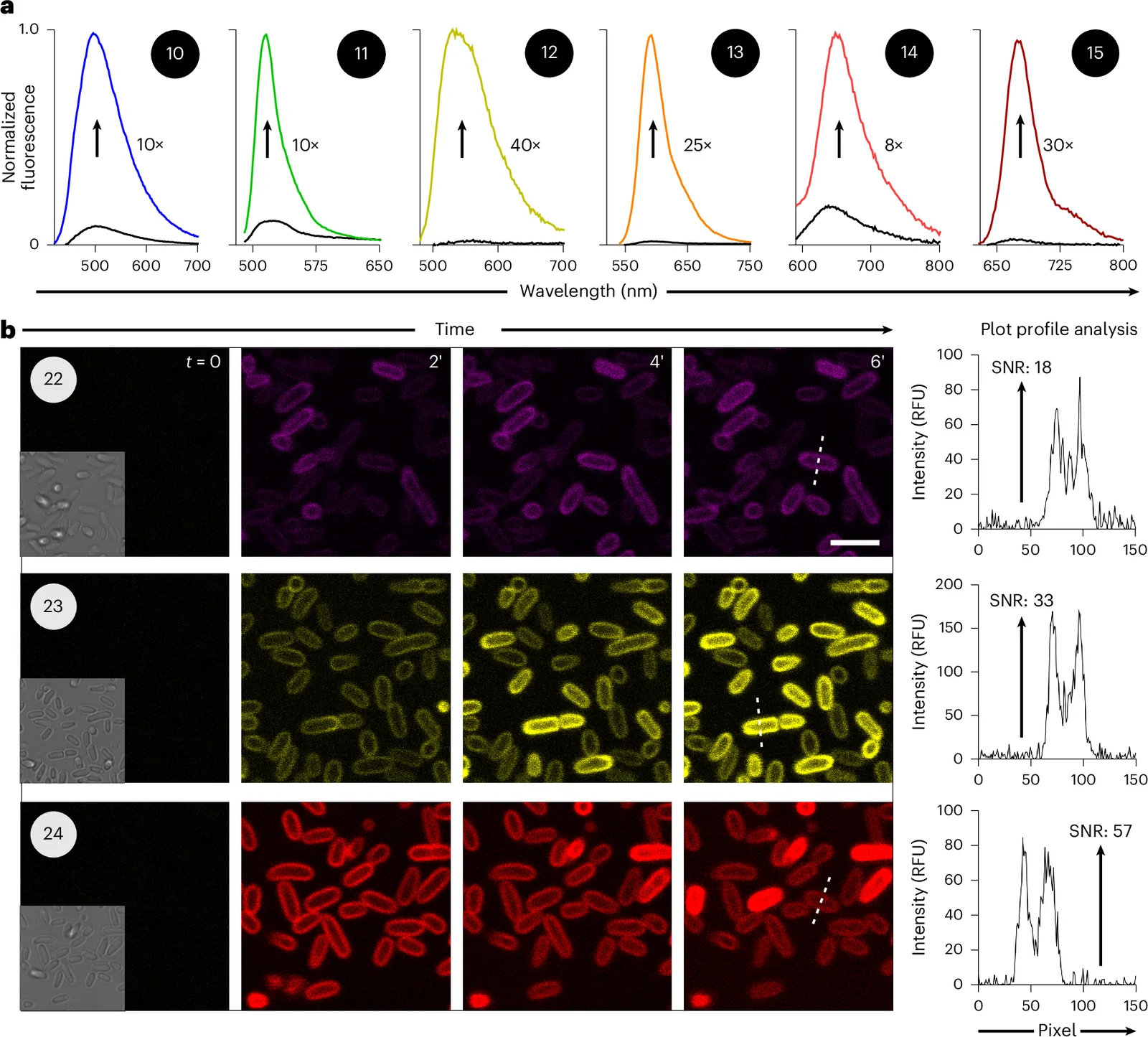
FgAAs are excellent fluorogenic building blocks for wash-free peptide-based imaging. **a**, Representative fluorescence spectra (from three independent experiments) of FgAAs **10**–**15** (10–100 µM) in glycerol:water (coloured lines) and in water (black lines). *λ*_exc_ of 390 nm (for **10**), 460 nm (for **11**), 450 nm (for **12**), 510 nm (for **13**), 560 nm (for **14**) and 590 nm (for **15**). **b**, Time-lapse (values in minutes) fluorescence microscopy images of *E. coli* cells after the addition of fluorogenic peptides **22** (25 µM, magenta), **23** (1 µM, yellow) and **24** (1 µM, red), imaged under wash-free conditions. *λ*_exc_ of 452 nm (**22**), 554 nm (**23**) and 650 nm (**24**). Scale bar, 3 µm. Representative plot profile analysis of peptide-stained cells (from *n* = 10; exemplars shown in corresponding fluorescence images as dashed white lines) and quantification of fluorescence SNRs for peptides **22**–**24**. RFU, relative fluorescence units. Source data

Having synthesized and analysed the fluorogenic mechanisms regulating the emission of our multicolour FgAAs, we evaluated their off-to-on switching and their capabilities for fluorogenic peptide synthesis. First, we analysed their fluorogenicity in response to glycerol and aqueous environments, where we confirmed that all FgAAs (**10**–**15**) were poorly emissive in water with remarkable turn-on fluorescence in glycerol (Fig. 3a). These results are in agreement with our computational observations, in which non-emissive PeT/TICT states are stabilized in polar environments, thereby activating PeT/TICT and effectively quenching their fluorescence. By contrast, PeT/TICT states are less accessible in hydrophobic or viscous environments, thereby enabling fluorescence recovery. Second, we examined the suitability of FgAAs as building blocks for solid-phase peptide synthesis (SPPS). Specifically, we confirmed that most FgAAs were stable to acid conditions (that is, 95% TFA) and scavengers (for example, triisopropylsilane (TIS) and dithiothreitol (DTT)) commonly used in SPPS, with the exception of BODIPY-AA (**11**), which degrades at high concentrations of TFA, as has been previously reported by our group and others^62,63^ (Supplementary Table 1).

Next, we synthesized model fluorogenic peptides to analyse their kinetics and determine their SNRs under wash-free imaging. For this purpose, we incorporated three multicoloured HPF-AA (**12**; excitation/emission 452/528 nm), O-rhodamine-AA (**13**; excitation/emission 554/582 nm) and Si-rhodamine-AA (**15**; excitation/emission 650/672 nm) covering different excitation and emission channels to the peptide sequence KKRFSFKKF with a reported affinity for lipid bilayers^64^. First, we synthesized the protected peptide sequence (for example, Arg(Pbf), Lys(Boc) and Ser(^*t*^Bu)) using standard Fmoc/^*t*^Bu SPPS conditions (that is, DIC and OxymaPure for couplings and piperidine:DMF (2:8) for Fmoc removal), and incorporated the three FgAAs at the N-terminal end (Supplementary Fig. 25). Cleavage with 95% TFA in the presence of TIS and H_2_O yielded the three FgAA-peptides (peptide **22** for HPF-AA, peptide **23** for O-rhodamine-AA and peptide **24** for Si-rhodamine-AA) (Supplementary Fig. 26) in purities >95% after HPLC purification (see the Supplementary Information for full synthetic and characterization details).

Finally, we evaluated the behaviour of the three fluorogenic peptides for real-time fluorescence imaging by incubating them in *Escherichia coli* bacterial cells and monitoring their binding to the cell envelope by confocal microscopy. In all three cases, we observed clear staining of the cell walls only after a few minutes, reaching excellent SNRs (peptide **22**, 18; peptide **23**, 33; and peptide **24**, 57) without the need for washing steps, which featured their strong fluorogenic character (Fig. 3b, Supplementary Fig. 27 and Supplementary Movies 1–3). Among the FgAAs, the HPF analogue showed the poorest contrast—in line with its lower extinction coefficient ~2 × 10^4^ M^−1^ cm^−1^ (Table 1). By contrast, O-rhodamine and Si-rhodamine derivatives (both showing extinction coefficients around 10^5^ M^−1^ cm^−1^) (Table 1) showed excellent contrast even at very short incubation times. Furthermore, we performed comparable wash-free microscopy experiments using the three FgAA-peptides **22**–**24** for labelling membranes of human neutrophils, proving compatibility with different cells and bioassays (Supplementary Fig. 28). Flow cytometry fluorescence quantification of peptide-stained *E. coli* bacterial cells confirmed the turn-on effect (Supplementary Fig. 29), as well as experiments in liposome suspensions as lipid-bilayer mimics, thus illustrating how de-quenching occurs upon binding (Supplementary Fig. 30). This effect arises from the transfer of the fluorophore from the polar aqueous phase into a less polar and more viscous lipid environment, which suppresses PeT or TICT quenching pathways and restores emission. Altogether, these results confirm the suitability of FgAAs as chemically stable building blocks to build fluorogenic peptides for wash-free imaging with high speed and contrast.

### Fluorogenic peptides targeting PD-1 receptors in activated T cells

PD-1 proteins are cell-surface receptors in active T cells, with key roles in regulating immune responses through interactions with their cognate ligand PD-L1^65^. The PD-1/PD-L1 pathway serves as a critical immune checkpoint and directly influences the efficacy of immunotherapies. Current methods to detect PD-1 receptors are limited to immunoassays^66^, small-molecule inhibitors^67^ or downstream signalling assays^68^; however, so far, there are no fluorogenic peptides for PD-1 receptors to build rapid screening platforms that identify immunomodulatory drugs in activated T cells. Given the near-infrared (NIR) emission and strong fluorogenic character of Si-rhodamine FgAAs, we decided to assess their utility in producing PD-1-targeting peptides for fluorescence-based bioassays in T cells.

**Fig. 4:**
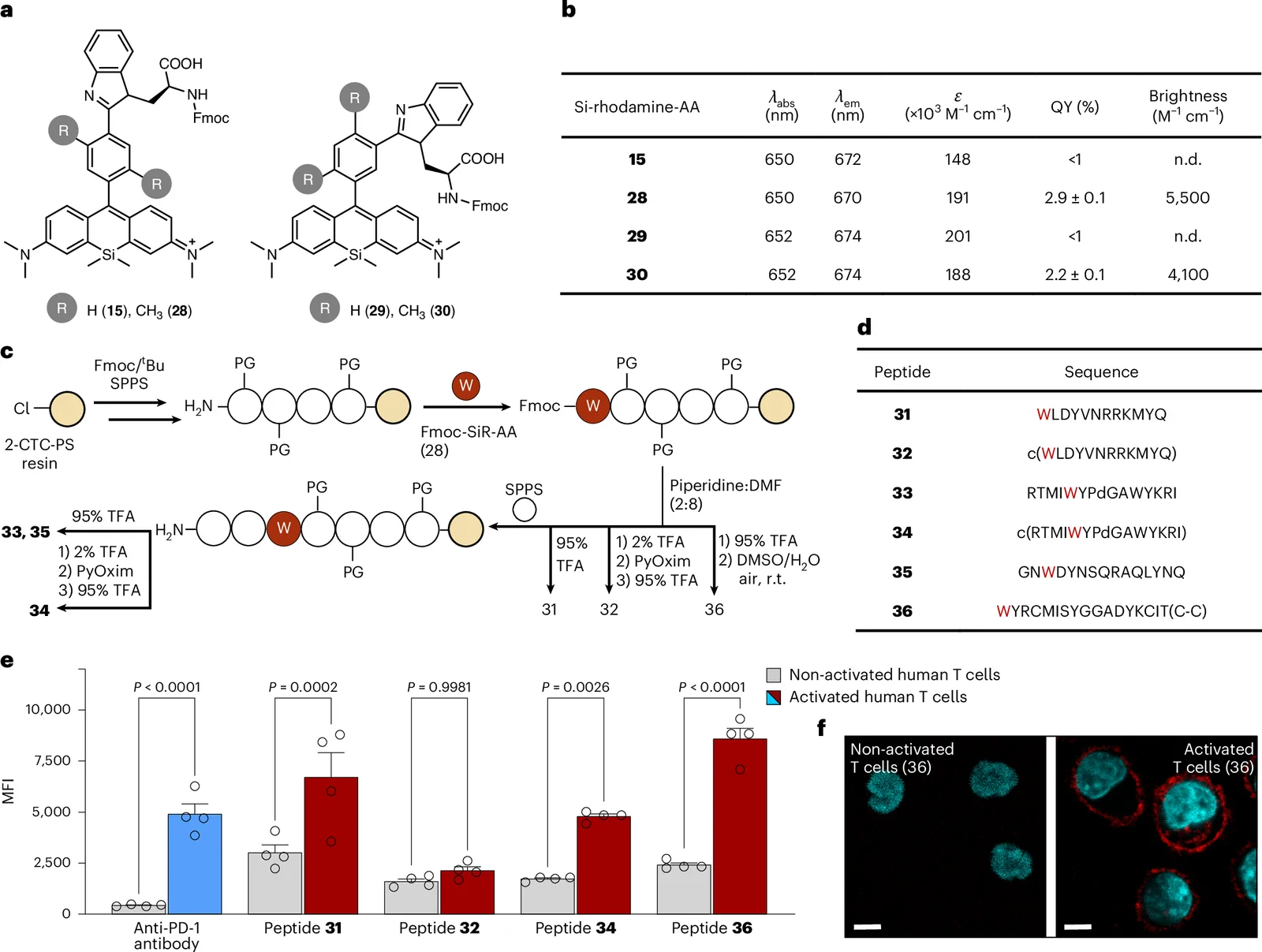
Synthesis and evaluation of fluorogenic peptides for activated T cells. **a**, Structures of Si-rhodamine amino acids **15**, **28**, **29** and **30**. **b**, Spectroscopic properties of Si-rhodamine amino acids. All values were measured in EtOH. **c**, The synthetic scheme for the preparation of fluorogenic peptides **31**–**36** containing the FgAA **28**. **d**, Sequences of the fluorogenic PD-1-binding peptides **31**–**36**. **e**, The MFIs of non-activated T cells (grey bars) and activated T cells (blue and red bars for anti-PD-1 and fluorogenic peptides, respectively) after labelling with anti-PD-1 antibody (20 min on ice) or with peptides **31**, **32**, **34** and **36** (5 µM, 30 min). Data were acquired on a NovoCyte flow cytometer and presented as means ± s.e.m. (*n* = 4 experimental replicates). *P* values were obtained from one-way ANOVA using multiple comparisons. **f**, Representative fluorescence microscopy images (from three independent experiments) of non-activated T cells and activated T cells after addition of nuclear stain Hoechst 33342 (cyan) and the fluorogenic peptide **36** (5 µM, 30 min; red) under wash-free conditions (*λ*_exc/em_ of 405 nm/430–492 nm (Hoechst 33342) and 650 nm/661–750 nm (**36**)). Scale bar, 4 µm. QY, fluorescence quantum yields measured using 3,3′-diethylthiadicarbocyanine iodide (EtOH) as the reference. Source data

Before the synthesis of fluorogenic PD-1-targeting peptides, we optimized the optical properties of Si-rhodamine FgAAs by introducing methyl groups to the meso-phenyl ring of the Si-rhodamine core. Previous experimental studies suggest that methyl substitution can enhance quantum yields by restricting phenyl ring rotation, though its effectiveness varies across fluorophore families^69^. Computational analysis indicated that the introduction of methyl substituents in the Si-rhodamine structure would not affect Trp-induced PeT quenching (Supplementary Figs. 31–36), thus we synthesized three additional Si-rhodamine FgAAs (**28**–**30**) to examine methylation patterns and positional effects of Trp (Fig. 4a; see the Supplementary Information for synthetic and characterization details). Neither methylation nor Trp position affected the absorption and emission profiles (excitation ~650 nm, emission ~672 nm), and strong PeT quenching was retained in all derivatives (Fig. 4b and Supplementary Fig. 37). From this chemical evaluation, we selected compound **28** as an NIR FgAA with high extinction coefficients and brightness, as well as excellent compatibility with SPPS (Fig. 4b and Supplementary Table 1).

Next, we explored the incorporation of FgAA **28** into different peptide sequences that bind human PD-1 receptors (Fig. 4c). In brief, we employed conventional SPPS^70,71^ to prepare a small collection of fluorogenic peptides (**31**–**36**), including linear and cyclic sequences binding PD-1^72–74^, where we incorporated the FgAA **28** as a replacement for their original Trp residue (for peptides **33**, **34** and **35**) (Fig. 4d) or at the N-terminal end (for peptides **31**, **32** and **36**) (Fig. 4d). All fluorogenic peptides were successfully synthesized using conventional SPPS and isolated in purities >95% after HPLC purification. The absorption and emission profiles for all peptides, including the FgAA **28**, were very similar and in line with the Si-rhodamine core (that is, *λ*_abs_/*λ*_em_ ~650/~670 nm) (Supplementary Fig. 38).

Next, we evaluated the labelling properties of peptides **31**–**36** in activated human T cells. In brief, we isolated primary human T cells from the peripheral blood of healthy donors and stimulated them with anti-CD3/CD28 antibody-coated beads to induce T cell activation^75,76^. After confirming that PD-1 receptors were highly expressed in activated T cells but absent in naïve T cells^77^ (Supplementary Fig. 39), we analysed their staining upon incubation with the peptides **31**–**36**. Four peptides (**31**, **32**, **34** and **36**) showed preferential labelling of activated T cells over non-activated T cells as well as a lack of cytotoxicity (Supplementary Fig. 40), thus we quantified their performance by flow cytometry analysis (Fig. 4e; see Supplementary Fig. 41 for the gating strategy). Peptide **36** displayed the best discrimination between PD-1-positive and PD-1-negative T cells, with optimal staining found at concentrations around 5 μM (Supplementary Figs. 42 and 43). Surface plasmon resonance (SPR) experiments with peptide **36** confirmed binding to human PD-1 receptors with a *K*_D_ value of 14 µM (Supplementary Fig. 44). Furthermore, competition experiments with the cognate ligand of PD-1 receptors (PD-L1) led to a marked reduction of the fluorescence signals of peptide **36** (Supplementary Fig. 45). Importantly, peptide **36** showed excellent discrimination of activated versus non-activated T cells in conventional culture media including serum (Supplementary Figs. 46 and 47). Overall, peptide **36** demonstrated strong fluorogenicity, enabling wash-free imaging of activated T cells with minimal signals in non-activated T cells (Fig. 4f and Supplementary Fig. 48), making it a promising tool for immunomodulatory drug screening.

### A fluorescence-based drug screen identifies modulators of T cell activation

**Fig. 5:**
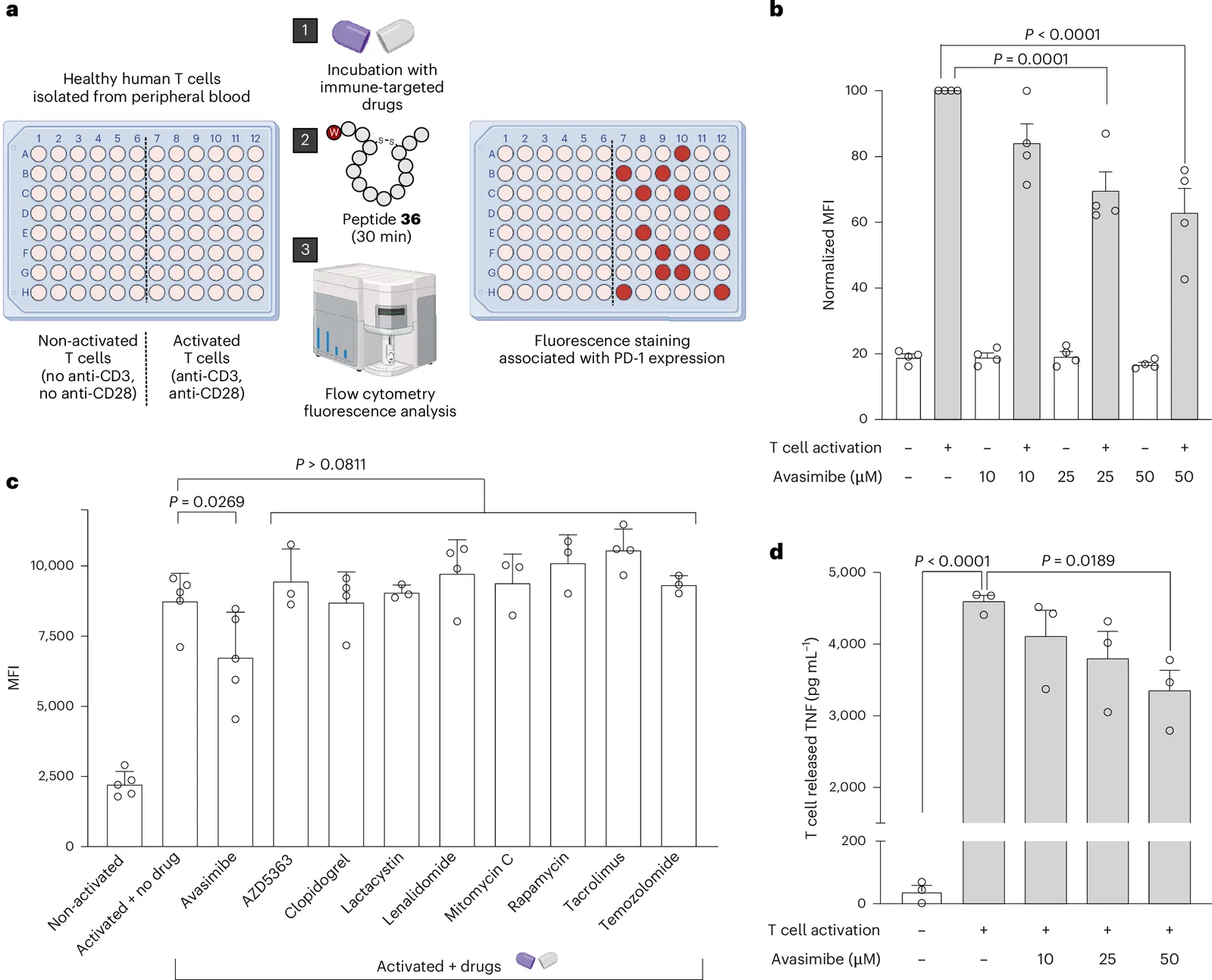
Fluorogenic peptide **36** identifies avasimibe as a modulator of PD-1 expression in human activated T cells. **a**, A schematic illustration of a flow cytometry drug screen in human T cells using fluorogenic peptide **36**. **b**, Normalized MFIs of PD-1-positive T cells after incubation with different concentrations of avasimibe (0–50 µM, 24 h) and subsequent incubation with peptide **36** (5 µM, 30 min). Data are presented as means ± s.e.m. (*n* = 4 experimental replicates). *P* values were obtained from one-way ANOVA. **c**, MFI values after incubation with immune-targeting drugs (experimental conditions detailed in Supplementary Table 2) and peptide **36** (5 µM, 30 min). Data are presented as means ± s.e.m. (*n* = 3 experimental replicates). *P* values were obtained from one-way ANOVA. **d**, The release of TNF (picograms per millilitre) from non-activated and activated T cells before and after treatment with avasimibe at the indicated concentrations. TNF levels were determined by ELISA. Data presented as means ± s.e.m. (*n* = 3 experimental replicates). *P* values were obtained from one-way ANOVA. Flow cytometer icon in **a** created in BioRender; Vendrell, M. https://biorender.com/y8fzuqk (2026). Source data

Having confirmed the suitability of peptide **36** as a fluorogenic probe for PD-1-expressing activated T cells, we next applied it to screen a small collection of immunomodulatory drugs in PD-1-positive activated T cells. To this end, we selected nine immune-targeting drugs (that is, rapamycin, AZD5363, mitomycin C, temozolomide, lactacystin, tacrolimus, lenalidomide, clopidogrel and avasimibe) that had not been previously investigated in PD-1 biology^78–84^. Building on the targeted fluorogenicity of peptide **36**, we built a fluorescence-based screen where activated human T cells were first treated with the drugs at their respective working concentrations (Supplementary Table 2) and subsequently labelled with peptide **36** to quantify their PD-1 levels (Fig. 5a). The majority of drugs did not display any substantial effect on the PD-1 expression of T cells, but we found that the drug avasimibe—known to regulate cholesterol metabolism in T cells^85^—showed a clear dose-dependent decrease in PD-1 expression (Fig. 5b,c and Supplementary Figs. 49–51). To the best of our knowledge, avasimibe has not been described as a potential modulator of PD-1 expression in activated human T cells.

To further analyse the effect of avasimibe on human T cells, we first corroborated the lack of cytotoxicity at the working concentrations (Supplementary Fig. 52) and then measured PD-1 downstream signalling in untreated and avasimibe-treated cells (Fig. 5d). Specifically, we employed enzyme-linked immunosorbent assays (ELISAs) to monitor the release of tumour necrosis factor (TNF), a cytokine secreted by activated T cells to induce cell differentiation and proliferation^86,87^. In these experiments, we observed that avasimibe significantly reduced cytokine release in human T cells, confirming its immunomodulatory effect. Altogether, these results demonstrate that FgAAs are excellent building blocks to generate turn-on fluorogenic peptides for wash-free imaging of immune biomarkers, as highlighted by the identification of avasimibe as a small-molecule drug with previously unreported PD-1 modulation activity.

## Discussion

The development of fluorogenic peptides for real-time bioassays has been hindered by the lack of general quenching approaches to build multicolour FgAAs. Herein, we describe a modular chemical strategy for the rapid generation of FgAAs with excellent brightness and turn-on ratios using chemically and optically diverse fluorophores. We demonstrated the versatility of our approach with the synthesis of multiple FgAAs via C–H activation of iodinated fluorophores covering the entire UV-to-NIR range, from blue-emitting coumarin structures to NIR Si-rhodamine fluorophores. Notably, iodinated precursors could be accessed via different synthetic strategies, expanding the potential applicability of this approach. Our detailed computational analysis showed that PeT and TICT mechanisms govern fluorescence quenching in Trp-linked fluorophores, offering key principles for the future design of FgAAs. Our analysis highlighted that the electronic isolation or conjugation of the Trp quencher with the fluorophore scaffold dictates the predominant quenching pathway as PeT or TICT, respectively, and that the brightness of FgAAs can be modulated by simple chemical modifications that suppress non-radiative decays (that is, insertion of methyl groups). Furthermore, our analyses indicate that this chemical strategy could be in principle applicable to many families of fluorophores (for example, cyanines), opening broad avenues for the rational design of fluorescent probes.

The integration of FgAAs into peptides enabled wash-free fluorescence imaging in real-time and live cells, including both prokaryotic and mammalian cells. In contrast to pre-formed fluorogens with short emission wavelengths and weak extinction coefficients (for example, dansyl, NBD, naphthalimide and prodan)^88–90^, FgAAs built from bright fluorescent scaffolds (for example, O-rhodamine and Si-rhodamine) exhibit unprecedented brightness (for example, in the range of 10^5^ M^−1^ cm^−1^) that result in excellent SNRs. Furthermore, FgAAs can be scaled up as Fmoc-protected building blocks that are compatible with SPPS (that is, with high chemical stability to coupling agents, strong acid media and scavenger compounds). We exploited the robust properties of NIR Si-rhodamine FgAAs to prepare a collection of PD-1 binding peptides, including variable sequences and both linear and cyclic structures. To the best of our knowledge, our lead peptide **36** is a unique example of a fluorogenic probe for human PD-1 receptors, whose expression is widely regarded as a biomarker of immune checkpoint modulation.

We leveraged the turn-on properties of peptide **36** to develop a fluorescence-based bioassay to monitor the PD-1 levels in activated human T cells after treatment with multiple immunomodulatory drugs. Using this assay, we identified the previously unreported role of avasimibe as a drug that reduces PD-1 levels in activated T cells and lowers downstream cytokine production. These findings establish a versatile and modular approach for designing fluorogenic peptides labelling a wide range of molecular and cellular targets, paving the way for advanced imaging tools, high-throughput screening assays and immunotherapy drug discovery.

## Methods

All research complies with the relevant ethical regulations approved by the University of Edinburgh (25-EMREC-023).

### General experimental information

The compounds 3,6-bis(dimethylamino)-9*H*-xanthen-9-one (**4**), 7-diethylamino-4-methylcoumarin (**16**) and 1,3,5,7-tetramethyl-8-phenyl-4,4-difluoroboradiazaindacene (**17**) were purchased from Enamine, Fluorochem and BLDpharm, respectively. Compounds **2** and **11** were prepared according to reported methods^57^. Silver tetrafluoroborate, trifluoroacetic acid, 1,4-diiodobenzene, 1,3-diiodobenzene, 2-iodo-1,4-dimethylbenzene, iodobenzene, 1-iodo-2,4-dimethylbenzene, methanesulfonic acid, 3-bromo-*N*,*N*-dimethylaniline, formaldehyde solution (37%) and 4-iodobenzaldehyde were purchased from Fluorochem. Pyridine, iodine, palladium(II) acetate, Fmoc-Trp-OH, *n*-butyllithium solution (2.5 M in hexanes), anhydrous tetrahydrofuran (THF), tetramethylethylenediamine (TMEDA), sodium acetate, resorcinol, benzaldehyde and coumarin 153 were purchased from Merck. The compounds 1,4-dioxane, DMF and acetic acid were obtained from Fisher Chemical. The compounds 1,4-diiodo-2,5-dimethylbenzene, 1,5-diiodo-2,4-dimethylbenzene and 3-ethyl-2-methylbenzothiazolium iodide were purchased from Tokyo Chemical Industry. For the SPPS, all Fmoc-AA-OH were obtained from Merck and Iris Biotech. The compounds 2-chlorotrityl chloride resin (100–200 mesh, 1% DVB), *N*,*N*’-diisopropylcarbodiimide (DIC), *N*,*N*-diisopropylethylamine (DIPEA), piperidine and dimethyl sulfoxide were purchased from Merck. OxymaPure and PyOxim were brought from Luxembourg Bio Technologies. For the biological experiments, reagents were purchased from Gibco (Thermo Fisher Scientific), GE Healthcare (Amersham) and BioLegend (antibodies). Unless indicated otherwise, reagents of the lowest possible endotoxin level were from Merck Life Science. Commercially available reagents were used without further purification. Thin-layer chromatography was conducted on Merck silica gel 60 F254 sheets and visualized by ultraviolet (254 and 365 nm). Reactions were monitored by high-performance liquid chromatography–mass spectrometry (HPLC-MS) using an HPLC Agilent Technologies 1200 with a Kinetex XB-C18 (50 × 4.6 mm, 5 μm) column in H_2_O (0.1% formic acid) and ACN (0.1% formic acid) at a 1.0 ml min^−1^ flow rate. The MS detector was configured with an electrospray ionization source and nitrogen as the nebulizer gas. All microwave reactions were carried out in 10-ml sealed glass tubes in a focused mono-mode microwave reactor (Biotage) featuring a surface sensor for internal temperature determination. Cooling was provided by compressed air ventilating the microwave chamber during the reaction. ^1^H and ^13^C spectra were recorded in a Bruker Avance 500 spectrometer (at 500 and 126 MHz, respectively). Chemical shifts were reported in ppm (δ) relative to the residual solvent signal. Multiplicities are referred to by the following abbreviations: s, singlet; d, doublet; t, triplet; dd, double doublet; ddd, double double doublet; dt, double triplet; q, quartet; m, multiplet. High-resolution mass spectrometry (HRMS) spectra were collected using an electrospray ionization (ESI) ThermoElectron MAT 900. MALDI analysis was performed on a Bruker UltrafleXtreme MALDI TOF-TOF mass spectrometer. Spectroscopic data were measured on a Synergy HT spectrophotometer (BioTek) and analysed using GraphPad Prism 10.0. Semi-preparative HPLC purifications were conducted in an Agilent 1220 HPLC with a Kinetex 150 × 10 mm (5 μm, 100 Å) C18 column in H_2_O (0.1% formic acid) and ACN (0.1% formic acid) at a 5.0 ml min^−1^ flow rate. Preparative HPLC was performed using an Agilent 1100 preparative HPLC with a Kinetex 150 × 21.2 mm (5 μm, 100 Å) C18 column in H_2_O (0.1% TFA) and ACN (0.1% TFA) at 20.0 ml min^−1^. Cleavage cocktail conditions were tested on an Agilent 1220 HPLC with a Kinetex 150 × 4.6 mm (5 μm, 100 Å) C18 column using A: H_2_O (0.1% formic acid) and B: ACN (0.1% formic acid) as eluents in a gradient from 5–95% B over 23 min at 1.8 ml min^−1^. Liquid chromatography–mass spectrometry analyses were performed in an Agilent 1260 Infinity at 35 °C using a Kinetex XB-C18 column (4.6 × 50 mm, 5 μm). H_2_O (0.1% formic acid) and ACN (0.1% formic acid) were used as mobile phases in a gradient from 5–95% ACN at a 1.0 ml min^−1^ flow rate.

### Spectroscopy measurements

Optical properties were measured in BioTek Cytation 3 or NanoDrop One C microvolume ultraviolet–visible spectrophotometers. For fluorescence quantum yield measurements, quinine sulfate (0.05 M H_2_SO_4_), fluorescein (0.1 M NaOH), coumarin 153 (EtOH), rhodamine B (EtOH), cresyl violet (MeOH) and 3,3′-diethylthiadicarbocyanine iodide (EtOH) were used as references^91,92^. Fluorescence quantum yields were determined using the equation QY = QY_ref_ × (*m*/*m*_ref_) × (*n*^2^/*n*_ref_^2^), where *m* is the slope of the line obtained from the plot of the area of fluorescence versus absorbance, *n* is the refractive index of the solvent and the subscript ‘ref’ refers to the reference with a known quantum yield. For extinction coefficient measurements, the absorbance of each sample at the maximum absorption wavelength was recorded, and the extinction coefficient was determined by fitting the data to Beer’s law. For liposome experiments, phosphatidylcholine-based liposomes were purchased from Liposoma, diluted in PBS and transferred into a black flat-bottom 96-well plate for optical measurements. Data analysis was performed using GraphPad Prism 10.0.

### T cell isolation and activation

Blood was collected from healthy human volunteers (male and female volunteers, age range 20–60 years old) following written informed consent and ethical approval at the University of Edinburgh (25-EMREC-023). All donor samples were anonymized following blood sample collection, and information on age, sex and gender was not available to the researchers. T cells were isolated from the peripheral blood by immunomagnetic negative selection using the EasySep Direct Human T Cell Isolation Kit (Stemcell Technologies). Isolated T cells were plated at 100,000 cells per well on a 96-well flat-bottomed plate and cultured for 72 h in DMEM containing 10% fetal bovine serum, 2-mercaptoethanol, l-glutamine and penicillin–streptomycin at 37 °C and 5% CO_2_. For activation, T cells were stimulated using Dynabeads human T-activator CD3/CD28 for 72 h (cell-to-bead ratio 1:1).

### PD-1 expression analysis

Cells were collected after 72 h, and beads were separated using an EasySep magnet (Stemcell Technologies). Both non-activated and activated T cells were treated with anti-PD-1 antibody (BioLegend, clone EH12.2H7, cat. no. 329936; dilution 1:100) for 20 min at 4 °C followed by washes with PBS. The cells were resuspended in FACS buffer (PBS + 0.1% BSA), and PD-1 expression was measured using flow cytometry (NovoCyte). The results were analysed using FCS Express 7 (De Novo Software).

### Flow cytometry and wash-free confocal imaging of T cells

Both non-activated and activated T cells were treated with PD-1 binding peptides for 30 min at room temperature, washed with PBS (unless stated otherwise) and then analysed using flow cytometry. Readouts are presented as either percentages or mean fluorescence intensity (MFI). For microscopy, cells were suspended in PBS after removing culture media and stained with Hoechst (1:5,000) for 15 min at room temperature followed by peptide treatment for 30 min at room temperature in the dark. Cells were then directly transferred without any washing steps to an 18-well IBIDI glass bottomed chamber and taken to the Leica Stellaris FALCON microscope directly for wash-free imaging (20× objective).

### Cytotoxicity assays

Non-activated and activated T cells were treated with Zombie Violet stain (dilution 1:100) for 20 min at 4 °C, followed by washes with PBS. The cells were then resuspended in FACS buffer (PBS + 0.1% BSA), and cytotoxicity was measured using flow cytometry (NovoCyte).

### Fluorescence detection in bacteria with peptides 22, 23 and 24

*E. coli* 2231734 were grown in fresh Lysogeny Broth overnight until they reached an OD_595_ of 1 on the next day. Bacteria were washed twice with PBS before adding 50 μl bacterial suspensions and 50 μl of peptide (final 25 μM for **22**, 1 μM for **23** and 1 μM for **24**) to a 96-well plate for flow cytometry (OD_595_ of 1) and an 18-well IBIDI chamber pre-coated with poly-d-Lysine for imaging experiments (OD_595_ of 0.25). Imaging was performed immediately after addition of the peptides without pre-incubation or washing steps. Readouts were presented as MFIs. Time-lapse wash-free images were captured on the Leica Stellaris FALCON microscope (63× objective).

### Human neutrophil isolation, induction of apoptosis and wash-free imaging

Neutrophils were isolated from whole blood using dextran sedimentation and a Percoll gradient^93^. In total, 40 million cells (5 million ml^−1^) were cultured in a T25 flask with RPMI with 10% autologous serum for 24 h in the incubator to induce apoptosis. The following day, the cells were stained with Hoechst for 15 min at room temperature followed by PBS washing. The cells were then resuspended in 100 μl PBS and incubated with peptides **22**–**24** for 15 min. Cells were imaged wash-free on a Leica Stellaris FALCON microscope (20× objective).

### TNF measurements

Supernatants were collected from T cell cultures after avasimibe treatment. TNF levels were measured in the undiluted T cell supernatant samples using an R&D ELISA kit following the manufacturer’s protocol (R&D Systems, DY210).

### SPR binding assays

Equilibrium dissociation constant (*K*_D_) values were assessed using a Biacore 8K SPR instrument (Cytiva) and multi-cycle kinetic analysis. A combined histidine-tag capture/amine coupling method was used to immobilize PD-1 on a Series S NTA sensor chip in HBS-EP+ running buffer (10 mM HEPES, pH 7.4, 150 mM NaCl, 3 mM EDTA and 0.005% Tween-20). Nitrilotriacetic acid groups were first chelated with nickel ions (0.5 mM NiCl_2_; Cytiva), activated with a 1:1 ratio mix of 0.2 M 1-ethyl-3-(3-dimethylaminopropyl) carbodiimide (Cytiva) and 50 mM *N*-hydroxysuccinimide, before a 420-s injection of recombinant human histidine-tagged PD-1 protein (150 µg ml^−1^; ACRO Biosystems, cat. no. PD1-H5221) and subsequent covalent immobilization at a level of approximately 2000 RU. Following successful immobilization, excess Ni^2+^ ions were removed using an injection of 0.35 M EDTA. Multi-cycle kinetic experiments were then carried out using HBS-EP+ supplemented with 5% DMSO (v/v). Peptide **36** and recombinant untagged human PD-L1 flowed over immobilized PD-1 for 120 s at a flow rate of 30 µl min^−1^. All experiments were carried out at 25 °C. The sensorgrams were referenced (activated and deactivated surface) and blank-subtracted (buffer injection). The data were evaluated using a steady-state binding model in the Biacore Insight Evaluation Software.

### Image and statistical analysis

The images were analysed by ImageJ or Leica Application Suite X software. Statistical analysis was performed with GraphPad Prism 10. Where data met the assumptions for parametric tests, one-way ANOVA was performed with Dunnett’s multiple comparison test. Individual values with standard errors of means are plotted throughout to show the variance of each dataset.

#### Synthetic procedures and characterization data

##### General procedure A: lithium–halogen exchange reaction

A solution of aryl iodide (1.5 mmol, 10.0 equiv.) in anhydrous THF (6.0 ml) was cooled down at −78 °C under a nitrogen atmosphere. In another flask, *n*-BuLi (2.5 M in hexane, 1.0 ml) was added dropwise to a mixture of anhydrous THF (2.0 ml) and TMEDA (220 µl, 1.5 mmol, 10.0 equiv.) at −78 °C under nitrogen atmosphere. After 5 min, a portion of the *n*-BuLi mixture (1.9 ml, 1.5 mmol) was added dropwise to the aryl iodide solution, and the mixture was stirred at −78 °C for 30 min. Then, compound **4** (42 mg, 0.15 mmol, 1.0 equiv.) or compound **5** (50 mg, 0.15 mmol, 1.0 equiv.) in anhydrous THF (3.0 ml) was added to the mixture under a nitrogen atmosphere and stirred at −78 °C for 30 min. The reaction mixture was diluted with DCM (20 ml) and quenched with 0.5 M HCl (15 ml). Organic fractions were extracted with DCM (50 ml × 3), combined, washed with brine and dried with MgSO_4_. The mixture was filtered and concentrated under reduced pressure. The crude material was purified as indicated to isolate the corresponding product.

##### 7-(Diethylamino)-3-iodo-4-methyl-2*H*-chromen-2-one (**1**)

Compound **1** was prepared following previously reported procedures^94^. To a mixture of iodine (7.6 g, 30.0 mmol) and pyridine (4.8 ml, 60.0 mmol) in 1,4-dioxane (100 ml) was added 7-diethylamino-4-methylcoumarin (2.3 g, 10.0 mmol). The mixture was stirred at room temperature for 16 h. A 50-ml saturated sodium thiosulfate solution was poured into the reaction mixture and extracted with DCM (150 ml × 3). The organic fractions were combined, washed with brine and dried with MgSO_4_. The mixture was filtered and concentrated under reduced pressure. The crude material was purified by normal-phase chromatography in hexane:ethyl acetate:DCM (4:0.5:1) to isolate the product as a yellow solid (2.5 g, 71%).

^1^H NMR (500 MHz, CD_3_OD): δ 7.57 (d, *J* = 9.2 Hz, 1H), 6.68 (dd, *J* = 9.2, 2.6 Hz, 1H), 6.47 (d, *J* = 2.6 Hz, 1H), 3.44 (q, *J* = 7.1 Hz, 4H), 2.58 (s, 3H) and 1.17 (t, *J* = 7.1 Hz, 6H) ppm.

^13^C NMR (126 MHz, CD_3_OD): δ 160.9, 159.3, 156.4, 152.7, 127.9, 110.6, 109.9, 97.5, 82.7, 45.7, 25.2 and 12.7 ppm.

HRMS (ESI+) *m*/*z* calculated for C_14_H_17_INO_2_ [M+H]^+^: 358.0299; found: 358.0295.

##### 6-Hydroxy-9-(4-iodophenyl)-3*H*-xanthen-3-one (**3**)

A mixture of 4-iodobenzaldehyde (2.3 g, 10.0 mmol) and resorcinol (2.2 g, 20.0 mmol) in methanesulfonic acid (100 ml) was stirred at 70 °C for 16 h. The mixture was cooled to room temperature and poured into sodium acetate solution (200 ml, 1 M). The solid was collected by filtration and washed with H_2_O_._ The crude was purified by preparative HPLC. After lyophilization, compound **3** was isolated as an orange solid (320 mg, 8%).

^1^H NMR (500 MHz, DMSO-*d*_*6*_): δ 8.02 (d, *J* = 8.1 Hz, 2H), 7.29 (d, *J* = 8.2 Hz, 2H), 7.16 (s, 1H), 7.14 (s, 1H) and 6.81–6.62 (m, 4H) ppm.

^13^C NMR (126 MHz, DMSO-*d*_*6*_): δ 156.7, 155.6, 151.1, 142.0, 137.6, 131.8, 131.4, 131.0, 128.5, 128.0, 125.4, 114.7, 106.0, 103.2, 102.4 and 96.8 ppm.

HRMS (ESI+) *m*/*z* calculated for C_19_H_12_IO_3_ [M+H]^+^: 414.9826; found, 414.9808.

##### 4,4’-Methylenebis(3-bromo-*N*,*N*-dimethylaniline)

To a mixture of 3-bromo-*N*,*N*-dimethylaniline (6.0 g, 30.0 mmol) in acetic acid (60.0 ml) was added 37% formaldehyde solution (1.4 ml, 19.5 mmol). The reaction was refluxed for 3 h. Once the reaction was complete, the solvent was removed under reduced pressure, and the mixture was basified using saturated aqueous NaHCO_3_ solution (pH 8). The aqueous layer was extracted with DCM (70 ml × 3). The organic fractions were combined, washed with brine and dried with MgSO_4_. The mixture was filtered and concentrated under reduced pressure. The crude material was purified by normal-phase chromatography using DCM:hexane (8:2) to isolate the product as a white solid (4.3 g, 70%).

^1^H NMR (500 MHz, CDCl_3_): δ 6.94 (d, *J* = 2.7 Hz, 2H), 6.85 (d, *J* = 8.5 Hz, 2H), 6.58 (dd, *J* = 8.6, 2.7 Hz, 2H), 4.00 (s, 2H) and 2.91 (s, 12H) ppm.

^13^C NMR (126 MHz, CDCl_3_): δ 150.2, 130.9, 127.3, 125.8, 116.4, 112.0, 40.7 and 40.0 ppm.

HRMS (ESI+) *m*/*z* calculated for C_17_H_21_Br_2_N_2_ [M+H]^+^: 411.0066; found: 411.0061.

##### 3,7-Bis(dimethylamino)-5,5-dimethyldibenzo[*b*,*e*]silin-10(5*H*)-one (**5**)

A solution of 4,4’-methylenebis(3-bromo-*N*,*N*-dimethylaniline) (2.0 g, 4.9 mmol) in anhydrous THF (15 ml) was cooled down at −78 °C under nitrogen atmosphere. To a mixture of anhydrous THF (20 ml) and TMEDA (2.9 ml, 19.6 mmol) at −78 °C under nitrogen atmosphere was added *n*-BuLi (2.5 M in hexane, 10 ml) dropwise. After 5 min, the *n*-BuLi mixture (25.8 ml, 19.6 mmol) was added dropwise to the dimethylaniline solution, and the mixture was stirred at −78 °C for 30 min. SiMe_2_Cl_2_ (1.2 ml, 9.8 mmol) in anhydrous THF (6.0 ml) was added into the mixture under nitrogen atmosphere. The mixture was stirred at room temperature overnight and quenched with 2 M HCl (24 ml). The aqueous layer was extracted with DCM (70 ml × 3). The organic fractions were combined, washed with brine and dried with MgSO_4_. The mixture was filtered and concentrated under reduced pressure. To the mixture in acetone (40 ml) at 0 °C was added KMnO_4_ (2.3 g, 14.6 mmol) over 30 min, and the solution was stirred at room temperature for 3 h. Once the reaction was completed, the solid was filtered, and solvents were removed under reduced pressure. The crude material was purified by normal-phase chromatography in hexane:ethyl acetate:DCM (5:1:1) to isolate the product as a yellow solid (503 mg, 32%).

^1^H NMR (500 MHz, CDCl_3_): δ 8.40 (d, *J* = 8.9 Hz, 2H), 6.87 (dd, *J* = 8.9, 2.6 Hz, 2H), 6.85 (d, *J* = 2.6 Hz, 2H), 3.10 (s, 12H) and 0.47 (s, 6H) ppm.

^13^C NMR (126 MHz, CDCl_3_): δ 185.3, 151.4, 140.7, 131.8, 114.85, 113.6, 40.4 and −0.9 ppm.

HRMS (ESI+) *m*/*z* calculated for C_19_H_24_N_2_OSiNa [M+Na]^+^: 347.1550; found: 347.1537.

##### *N*-(6-(dimethylamino)-9-(4-iodophenyl)-3*H*-xanthen-3-ylidene)-*N*-methylmethan-aminium chloride (**6**)

Following general procedure A, the reaction was run using 1,4-diiodobenzene (211 µl, 1.5 mmol) and compound **4** (42 mg, 0.15 mmol). The mixture was purified by normal-phase chromatography in DCM:MeOH (9:1) to isolate the product as a red solid (30 mg, 40%).

^1^H NMR (500 MHz, CD_3_OD): δ 8.11–8.01 (m, 2H), 7.41 (s, 1H), 7.39 (s, 1H), 7.31–7.24 (m, 2H), 7.14 (d, *J* = 2.5 Hz, 1H), 7.12 (d, *J* = 2.5 Hz, 1H), 7.01–6.98 (m, 2H) and 3.33 (s, 12H) ppm.

^13^C NMR (126 MHz, CD_3_OD): δ 159.3, 159.1, 158.4, 139.4, 133.0, 132.6, 132.5, 115.7, 114.4, 97.6, 97.4 and 40.9 ppm.

HRMS (ESI+) *m*/*z* calculated for C_23_H_22_IN_2_O [M]^+^: 469.0771; found: 469.0761.

##### *N*-(7-(dimethylamino)-10-(4-iodophenyl)-5,5-dimethyldibenzo[*b*,*e*]silin-3(5*H*)-ylidene)-*N*-methylmethanaminium chloride (**7**)

Following general procedure A, the reaction was run using 1,4-diiodobenzene (495 mg, 1.5 mmol) and compound **5** (50 mg, 0.15 mmol). The mixture was purified by normal-phase chromatography in DCM:MeOH (95:5) to isolate the product as a blue solid (68 mg, 83%).

^1^H NMR (500 MHz, CDCl_3_): δ 7.87 (d, *J* = 8.2 Hz, 2H), 7.23 (d, *J* = 2.8 Hz, 2H), 7.11 (d, *J* = 9.7 Hz, 2H), 7.00 (d, *J* = 8.2 Hz, 2H), 6.64 (dd, *J* = 9.7, 2.8 Hz, 2H), 3.42 (s, 12H) and 0.65 (s, 6H) ppm.

^13^C NMR (126 MHz, CDCl_3_): δ 167.9, 154.2, 148.8, 142.2, 138.6, 137.5, 131.1, 127.9, 121.4, 114.0, 94.8, 41.6 and −0.4 ppm.

HRMS (ESI+) *m*/*z* calculated for C_25_H_28_IN_2_Si [M]^+^: 511.1061: found: 511.1077.

##### 4-iodobenzene-1,3-diol (**8**)

Resorcinol (2.0 g, 18.2 mmol) and iodine (4.6 g, 18.2 mmol) were dissolved in DMSO (20 ml) and stirred at 80 °C for 5 h. The reaction mixture was then quenched with saturated aqueous solution of Na_2_S_2_O_3_ and extracted with ethyl acetate (150 ml × 3). The combined organic layers were washed with brine, dried with Na_2_SO_4_ and concentrated under reduced pressure. The crude material was purified by normal-phase chromatography in ethyl acetate:DCM (1:9) to yield the product as a yellow solid (1.37 g, 32%).

^1^H NMR (500 MHz, CDCl_3_): δ 7.45 (d, *J* = 8.6 Hz, 1H), 6.54 (d, *J* = 2.8 Hz, 1H), 6.27 (dd, *J* = 8.6, 2.7 Hz, 1H) and 5.32 (bs, 2H) ppm.

^13^C NMR (126 MHz, CDCl_3_): δ 157.7, 155.8, 138.5, 110.6, 102.8 and 74.7 ppm.

HRMS (ESI−) *m*/*z* calculated for C_6_H_4_IO_2_ [M-H]^−^: 234.9262; found: 234.9269.

##### 7-(Dimethylamino)-2-iodo-3*H*-phenoxazin-3-one (**9**)

To a mixture of *N*,*N*-dimethylaniline (1.5 ml, 11.8 mmol) and concentrated HCl (5.0 ml) at 0 °C was added sodium nitrite (860 mg, 12.5 mmol, 1.1 equiv.) in 1.5 ml of distilled water dropwise. The mixture was stirred at 0 °C for 1 h. The resulting precipitate was filtered off, washed with cold ethanol and dried to obtain 1.5 g *N,N*-dimethyl-*p*-nitrosoaniline, which was used for the next step without further purification. A mixture of 4-iodobenzene-1,3-diol (8) (2 g, 8.5 mmol, 1.1 equiv.), *N*,*N*-dimethyl-4-nitrosoaniline (1.2 g, 8.0 mmol, 1.0 equiv.) and anhydrous ZnCl_2_ (600 mg, 4.4 mmol, 0.6 equiv.) in anhydrous 2-propanol (50 ml) was stirred at 85 °C for 3 h. The reaction mixture was quenched with saturated NaHCO_3_. The aqueous solution was extracted with ethyl acetate (150 ml x 3), and the combined organic layers were washed with brine, dried over anhydrous MgSO_4_ and concentrated under vacuum. The crude material was purified by flash column chromatography on silica gel in hexane:ethyl acetate (8:2) to yield the product as a purple solid (162 mg, 20%).

^1^H NMR (500 MHz, CDCl_3_): δ 8.21 (s, 1H), 7.60 (d, *J* = 9.1 Hz, 1H), 6.76 (dd, *J* = 9.2, 2.7 Hz, 1H), 6.48 (d, *J* = 2.7 Hz, 1H), 6.44 (s, 1H) and 3.18 (s, 6H) ppm.

^13^C NMR (126 MHz, CDCl_3_): δ 179.5, 154.3, 150.7, 146.9, 142.9, 140.8, 132.2, 126.9, 111.5, 111.5, 103.4, 96.7 and 40.7 ppm.

HRMS (ESI+) *m*/*z* calculated for C_14_H_11_IN_2_O_2_Na [M+Na]^+^: 388.9757; found, 388.9773.

##### (*S*)-2-((((9*H*-fluoren-9-yl)methoxy)carbonyl)amino)-3-(2-(7-(diethylamino)-4-methyl-2-oxo-2*H*-chromen-3-yl)-1*H*-indol-3-yl)propanoic acid (**10**)

Iodinated coumarin **1** (50 mg, 0.14 mmol, 1.0 equiv.), Fmoc-Trp-OH (60 mg, 0.14 mmol, 1.0 equiv.), AgBF_4_ (27 mg, 0.14 mmol, 1.0 equiv.) and Pd(OAc)_2_ (6.7 mg, 0.03 mmol, 0.2 equiv.) were dissolved in anhydrous DMF (3.0 ml) in a microwave reactor vessel, followed by the addition of trifluoroacetic acid (10.7 μl, 0.14 mmol, 1.0 equiv.). The mixture was subjected to two cycles of microwave irradiation for 30 min at 80 °C. Additional fresh AgBF_4_ (1.0 equiv.) and Pd(OAc)_2_ (0.2 equiv.) were added after each cycle. The mixture was diluted in DCM and filtered through celite. The solution was concentrated under reduced pressure and purified by reversed-phase preparative HPLC. After lyophilization, compound **10** was isolated as a brown solid (14 mg, 15%).

^1^H NMR (500 MHz, CDCl_3_): δ 8.45–8.01 (m, 1H), 7.81–7.59 (m, 3H), 7.57–7.42 (m, 3H), 7.39–7.29 (m, 3H), 7.25–7.04 (m, 3H), 6.80 (s, 1H), 6.68 (s, 1H), 6.57–6.27 (m, 1H), 5.58 (s, 1H), 4.80–4.59 (m, 1H), 4.27–3.95 (m, 3H), 3.63–3.21 (m, 5H), 3.15–2.90 (m, 1H), 2.26 (s, 3H) and 1.34–0.90 (m, 6H) ppm.

^13^C NMR (126 MHz, CDCl_3_): δ 174.0, 163.3, 157.5, 155.6, 154.5, 144.0, 141.3, 136.5, 129.4, 127.7, 127.3, 127.2, 126.9, 125.6, 123.0, 120.3, 120.0, 119.2, 111.4, 110.4, 98.5, 67.7, 54.2, 47.1, 45.7, 27.0, 16.6 and 12.4 ppm.

HRMS (ESI+) *m*/*z* calculated for C_40_H_38_N_3_O_6_ [M+H]^+^: 656.2755; found, 656.2754.

##### (*S*)-2-((((9*H*-fluoren-9-yl)methoxy)carbonyl)amino)-3-(2-(4-(6-hydroxy-3-oxo-3*H*-xanthen-9-yl)phenyl)-1*H*-indol-3-yl)propanoic acid (**12**)

Iodinated compound **3** (50 mg, 0.12 mmol, 1.0 equiv.), Fmoc-Trp-OH (51 mg, 0.12 mmol, 1.0 equiv.), AgBF_4_ (23 mg, 0.12 mmol, 1.0 equiv.) and Pd(OAc)_2_ (4.5 mg, 0.02 mmol, 0.2 equiv.) were dissolved in anhydrous DMF (3.0 ml) in a microwave reactor vessel, followed by the addition of trifluoroacetic acid (9.2 μl, 0.12 mmol, 1.0 equiv.). The mixture was subjected to three cycles of microwave irradiation for 30 min at 100 °C. Additional fresh AgBF_4_ (1.0 equiv.) and Pd(OAc)_2_ (0.2 equiv.) were added after each cycle. The mixture was diluted in MeOH and filtered through celite. The solution was concentrated under reduced pressure and purified by reversed-phase preparative HPLC. After lyophilization, compound **12** was isolated as an orange solid (13 mg, 15%).

^1^H NMR (500 MHz, DMSO-*d*_*6*_): δ 11.41 (s, 1H), 7.95 (d, *J* = 8.2 Hz, 2H), 7.89–7.80 (m, 3H), 7.77 (d, *J* = 8.0 Hz, 1H), 7.66–7.56 (m, 4H), 7.48–7.29 (m, 6H), 7.26 (t, *J* = 7.2 Hz, 1H), 7.21 (t, *J* = 7.8 Hz, 1H), 7.15 (t, *J* = 7.5 Hz, 1H), 7.05 (t, *J* = 7.5 Hz, 1H), 6.86–6.76 (m, 4H), 4.39 (q, *J* = 8.4 Hz, 1H), 4.17–4.03 (m, 3H), 3.51 (dd, *J* = 14.7, 5.7 Hz, 1H) and 3.34 (dd, *J* = 14.7, 8.6 Hz, 1H) ppm.

^13^C NMR (126 MHz, DMSO-*d*_*6*_): δ 174.0, 157.5, 156.4, 144.2, 144.2, 141.1, 136.7, 134.7, 134.7, 131.9, 131.4, 130.5, 129.4, 128.5, 128.1, 128.0, 127.5, 125.8, 125.8, 122.5, 120.5, 119.7, 119.5, 115.5, 111.8, 109.7, 103.7, 66.3, 55.6, 46.9 and 27.4 ppm.

HRMS (ESI+) *m*/*z* calculated for C_45_H_32_N_2_O_7_Na [M+Na]^+^: 735.2102; found: 735.2134.

##### (*S*)-*N*-(9-(4-(3-(2-((((9*H*-fluoren-9-yl)methoxy)carbonyl)amino)-2-carboxyethyl)-1*H*-indol-2-yl)phenyl)-6-(dimethylamino)-3*H*-xanthen-3-ylidene)-*N*-methylmeth-anaminium 2,2,2-trifluoroacetate (**13**)

Iodinated compound **6** (50 mg, 0.10 mmol, 1.0 equiv.), Fmoc-Trp-OH (43 mg, 0.10 mmol, 1.0 equiv.), AgBF_4_ (19 mg, 0.10 mmol, 1.0 equiv.) and Pd(OAc)_2_ (4.5 mg, 0.02 mmol, 0.2 equiv.) were dissolved in anhydrous DMF (3.0 ml) in a microwave reactor vessel, followed by the addition of trifluoroacetic acid (7.7 μl, 0.10 mmol, 1.0 equiv.). The mixture was subjected to three cycles of microwave irradiation for 30 min at 80 °C. Additional fresh AgBF_4_ (1.0 equiv.) and Pd(OAc)_2_ (0.2 equiv.) were added after each cycle. The mixture was diluted in MeOH and filtered through celite. The solution was concentrated under reduced pressure and purified by reversed-phase preparative HPLC. After lyophilization, compound **13** was isolated as a red solid (18 mg, 21%).

^1^H NMR (500 MHz, CD_3_OD): δ 7.91 (d, *J* = 7.8 Hz, 1H), 7.83 (d, *J* = 8.0 Hz, 1H), 7.71 (d, *J* = 7.9 Hz, 1H), 7.66–7.50 (m, 3H), 7.45–7.38 (m, 5H), 7.35–7.21 (m, 5H), 7.21–7.11 (m, 3H), 7.11–7.05 (m, 1H), 6.95–6.84 (m, 4H), 4.43 (dd, *J* = 8.0, 5.4 Hz, 1H), 3.96 (q, *J* = 7.0 Hz, 2H), 3.85 (dd, *J* = 8.8, 6.4 Hz, 1H), 3.67 (dd, *J* = 14.8, 5.4 Hz, 1H), 3.55 (dd, *J* = 14.8, 8.1 Hz, 1H) and 3.26 (s, 12H) ppm.

^13^C NMR (126 MHz, CD_3_OD): δ 175.2, 159.2, 158.8, 158.8, 158.0, 145.2, 145.0, 142.5, 142.3, 138.1, 137.0, 136.1, 132.9, 131.9, 131.5, 130.3, 129.7, 128.8, 128.7, 128.1, 126.5, 126.4, 123.5, 120.8, 120.7, 120.5, 120.0, 119.2, 115.4, 114.2, 112.2, 110.3, 97.6, 68.3, 56.4, 47.9, 40.9 and 27.9 ppm.

HRMS (ESI+) *m*/*z* calculated for C_49_H_43_N_4_O_5_ [M]^+^: 767.3228; found: 767.3238.

##### (*S*)-2-((((9*H*-fluoren-9-yl)methoxy)carbonyl)amino)-3-(2-(7-(dimethylami-no)-3-oxo-3*H*-phenoxazin-2-yl)-1*H*-indol-3-yl)propanoic acid (**14**)

To a mixture of Fmoc-Trp-OH (250 mg, 0.59 mmol, 1.0 equiv.), compound **9** (322 mg, 0.88 mmol, 1.5 equiv.), AgBF_4_ (230 mg, 1.2 mmol, 2 equiv.), TFA (45 μl, 0.88 mmol, 1.0 equiv.) and Pd(OAc)_2_ (6.6 mg, 0.03 mmol, 0.05 equiv.) in a microwave reactor vessel in dry DMF (9 ml) was heated under microwave irradiation (250 W) at 90 °C for 4 h. The mixture was filtered through celite and washed with ethyl acetate. The solution was concentrated and purified by flash column chromatography on silica gel, eluting with 20–50% ethyl acetate in toluene to yield compound **14** as a purple solid (100 mg, 27%).

^1^H NMR (500 MHz, DMSO-*d*_*6*_): δ 11.24 (s, 1H), 7.84 (d, *J* = 7.6 Hz, 2H), 7.71–7.63 (m, 2H), 7.61–7.53 (m, 3H), 7.42–7.34 (m, 3H), 7.24 (dt, *J* = 22.7, 7.1 Hz, 2H), 7.12 (t, *J* = 7.6 Hz, 1H), 7.01 (t, *J* = 7.5 Hz, 1H), 6.98 (dd, *J* = 9.2, 2.7 Hz, 1H), 6.77 (d, *J* = 2.6 Hz, 1H), 6.41 (s, 1H), 6.26 (s, 2H), 4.39–4.26 (m, 1H), 4.10–3.85 (m, 5H) and 3.19 (s, 6H) ppm.

^13^C NMR (126 MHz, DMSO-*d*_*6*_): δ 175.3, 158.0, 149.7, 145.1, 145.0, 142.3, 142.3, 137.9, 135.4, 134.5, 131.4, 129.8, 128.7, 128.7, 128.1, 126.3, 126.2, 123.5, 120.8, 120.8, 120.4, 120.0, 112.3, 112.0, 104.2, 98.1, 67.9, 56.1, 49.9, 42.3 and 28.6 ppm.

HRMS (ESI) calculated for C_40_H_33_N_4_O_6_ [M + H]^+^: 665.2395; found, 665.2370.

##### (*S*)-*N*-(10-(4-(3-(2-((((9*H*-fluoren-9-yl)methoxy)carbonyl)amino)-2-carboxyethyl)-1*H*-indol-2-yl)phenyl)-7-(dimethylamino)-5,5-dimethyldibenzo[*b*,*e*]silin-3(5*H*)-ylidene)-*N*-methylmethanaminium 2,2,2-trifluoroacetate (**15**)

Iodinated compound **7** (50 mg, 0.09 mmol, 1.0 equiv.), Fmoc-Trp-OH (38 mg, 0.09 mmol, 1.0 equiv.), AgBF_4_ (18 mg, 0.09 mmol, 1.0 equiv.) and Pd(OAc)_2_ (4.5 mg, 0.02 mmol, 0.2 equiv.) were dissolved in anhydrous DMF (3.0 ml) in a microwave reactor vessel, followed by the addition of trifluoroacetic acid (6.9 μl, 0.09 mmol, 1.0 equiv.). The mixture was subjected to two cycles of microwave irradiation for 30 min at 80 °C. Additional fresh AgBF_4_ (1.0 equiv.) and Pd(OAc)_2_ (0.2 equiv.) were added after each cycle. The mixture was diluted in DCM and filtered through celite. The solution was concentrated under reduced pressure and purified by reversed-phase preparative HPLC. After lyophilization, compound **15** was isolated as a blue solid (24 mg, 28%).

^1^H NMR (500 MHz, CDCl_3_): δ 9.75 (s, 1H), 7.72–7.52 (m, 5H), 7.46–7.28 (m, 5H), 7.25–7.15 (m, 4H), 7.04–6.87 (m, 6H), 6.55 (d, *J* = 8.8 Hz, 2H), 5.77 (bs, 1H), 4.64 (q, *J* = 6.8 Hz, 1H), 4.16 (t, *J* = 8.9 Hz, 1H), 4.10 (t, *J* = 9.0 Hz, 1H), 3.99 (t, *J* = 7.5 Hz, 1H), 3.56 (d, *J* = 5.7 Hz, 2H), 3.15 (s, 12H) and 0.52 (s, 6H) ppm.

^13^C NMR (126 MHz, CDCl_3_): δ 174.0, 170.7, 160.9, 160.6, 160.3, 160.0, 155.9, 154.1, 148.1, 144.1, 143.9, 143.2, 137.8, 136.5, 135.6, 133.8, 129.8, 129.3, 128.3, 128.2, 127.8, 127.8, 127.3, 125.5, 125.4, 122.3, 120.3, 119.9, 119.7, 119.0, 117.3, 115.0, 114.2, 112.7, 112.1, 107.9, 67.2, 55.3, 47.1, 40.7, 27.8 and −0.8 ppm.

HRMS (ESI+) *m*/*z* calculated for C_51_H_49_N_4_O_4_Si [M]^+^: 809.3518; found: 809.3510.

##### 6-Hydroxy-9-phenyl-3*H*-xanthen-3-one (**18**)

A mixture of benzaldehyde (204 μl, 2.0 mmol) and resorcinol (440 mg, 4.0 mmol) in methanesulfonic acid (10 ml) was stirred at 70 °C for 16 h. The mixture was cooled to room temperature and poured into a sodium acetate solution (80 ml, 3 M). The solid obtained was collected by filtration and washed with H_2_O_._ The crude was purified by preparative HPLC. After lyophilization, compound **18** was isolated as an orange solid (22 mg, 4%).

^1^H NMR (500 MHz, CD_3_OD): δ 7.78–7.71 (m, 3H), 7.71 (s, 1H), 7.69 (s, 1H), 7.58 (d, *J* = 1.4 Hz, 1H), 7.57 (d, *J* = 1.7 Hz, 1H), 7.27 (d, *J* = 2.3 Hz, 2H), 7.19 (d, *J* = 2.3 Hz, 1H) and 7.18 (d, *J* = 2.3 Hz, 1H) ppm.

^13^C NMR (126 MHz, CD_3_OD): δ 173.5, 165.7, 161.0, 134.7, 133.0, 132.1, 130.9, 130.1, 121.6, 117.7 and 103.6 ppm.

HRMS (ESI+) *m*/*z* calculated for C_19_H_13_O_3_ [M+H]^+^: 289.0859; found, 289.0865.

##### *N*-(6-(dimethylamino)-9-phenyl-3*H*-xanthen-3-ylidene)-*N*-methylmethanaminium chloride (**19**)

Following general procedure A, the reaction was run using iodobenzene (167 µl, 1.5 mmol) and 3,6-bis(dimethylamino)-9*H*-xanthen-9-one (42 mg, 0.15 mmol). The mixture was purified by normal-phase chromatography in DCM:MeOH (95:5) to isolate compound **19** as a red solid (31 mg, 55%).

^1^H NMR (500 MHz, CD_3_OD): δ 7.77–7.69 (m, 3H), 7.55–7.50 (m, 2H), 7.45 (s, 1H), 7.43 (s, 1H), 7.16 (d, *J* = 2.5 Hz, 1H), 7.14 (d, *J* = 2.5 Hz, 1H), 7.03 (d, *J* = 2.5 Hz, 2H) and 3.36 (s, 12H) ppm.

^13^C NMR (126 MHz, CD_3_OD): δ 159.7, 159.4, 159.0, 133.6, 132.8, 131.4, 130.7, 130.0, 115.6, 114.7, 97.6 and 40.9 ppm.

HRMS (ESI+) *m*/*z* calculated for C_23_H_23_N_2_O [M]^+^: 343.1805; found: 343.1790.

##### 7-(Dimethylamino)-3*H*-phenoxazin-3-one (**20**)

To a mixture of *N*,*N*-dimethylaniline (1.5 ml, 11.8 mmol, 1 equiv.) and concentrated HCl (5.0 ml) at 0 °C, sodium nitrite (860 mg, 12.5 mmol, 1.1 equiv.) in 1.5 ml of distilled water was added dropwise. The mixture was stirred at 0 °C for 1 h. The precipitate was collected, washed with cold ethanol and dried to obtain 1.6 g *N*,*N*-dimethyl-*p*-nitrosoaniline, which was used for the next step without further purification. The mixture of resorcinol (1.2 g, 11.0 mmol, 1 equiv.), *N*,*N*-dimethyl-4-nitrosoaniline (1.6 g, 11.0 mmol, 1 equiv.) and acetic acid (20 ml) was stirred at 130 °C for 1 h. The mixture was concentrated and purified using reverse-phase chromatography to yield compound 20 as purple-red solid (46 mg, 1%).

^1^H NMR (500 MHz, CD_3_OD): δ 7.83–7.64 (m, 2H), 7.40 (d, *J* = 9.0 Hz, 1H), 6.99 (d, *J* = 9.2 Hz, 1H), 6.94 (s, 1H), 6.71 (s, 1H) and 3.41 (s, 6H) ppm.

^13^C NMR (126 MHz, CD_3_OD): δ 174.5, 160.4, 150.6, 150.1, 138.3, 135.8, 135.3, 135.2, 123.1, 120.5, 103.7, 98.1 and 42.3 ppm.

HRMS (ESI) calculated for C_14_H_13_N_2_O_2_ [M+H]^+^: 241.0972; found, 241.0968.

##### *N*-(7-(dimethylamino)-5,5-dimethyl-10-phenyldibenzo[*b*,*e*]silin-3(5*H*)-ylidene)-*N*-methylmethanaminium chloride (**21**)

Following general procedure A, the reaction was run using iodobenzene (167 µl, 1.5 mmol) and compound **5** (50 mg, 0.15 mmol). The mixture was purified by normal-phase chromatography in DCM:MeOH (95:5) to isolate compound **21** as a blue solid (55 mg, 87%).

^1^H NMR (500 MHz, CDCl_3_): δ 7.54–7.50 (m, 3H), 7.24–7.22 (m, 2H), 7.21 (d, J = 2.8 Hz, 2H), 7.14 (d, J = 9.6 Hz, 2H), 6.63 (dd, J = 9.6, 2.8 Hz, 2H), 3.41 (s, 12H) and 0.65 (s, 6H) ppm.

^13^C NMR (126 MHz, CDCl_3_): δ 170.0, 154.2, 148.8, 142.6, 139.0, 129.3, 128.9, 128.3, 128.2, 121.1, 113.9, 41.5 and −0.39 ppm.

HRMS (ESI+) *m*/*z* calculated for C_25_H_29_N_2_Si [M]^+^: 385.2095; found: 385.2091.

##### *N*-(7-(dimethylamino)-10-(4-iodo-2,5-dimethylphenyl)-5,5-dimethyldibenzo[*b*,*e*]-silin-3(5*H*)-ylidene)-*N*-methylmethanaminium chloride (**25**)

Following general procedure A, the reaction was run using 1,4-diiodo-2,5-dimethylbenzene (537 mg, 1.5 mmol) and compound **5** (50 mg, 0.15 mmol). The mixture was purified by normal-phase chromatography in DCM:MeOH (95:5) to isolate the product as a blue solid (63 mg, 71%).

^1^H NMR (500 MHz, CDCl_3_): δ 7.80 (s, 1H), 7.22 (d, *J* = 2.9 Hz, 2H), 7.09 (d, *J* = 9.6 Hz, 2H), 6.96 (s, 1H), 6.66 (dd, *J* = 9.6, 2.8 Hz, 2H), 3.42 (s, 12H), 2.44 (s, 3H), 1.95 (s, 3H), 0.64 (s, 3H) and 0.63 (s, 3H) ppm.

^13^C NMR (126 MHz, CDCl_3_): δ 168.3, 154.3, 148.6, 141.5, 140.5, 139.1, 138.8, 135.2, 129.8, 127.5, 121.2, 114.3, 101.5, 41.5, 27.6, 18.6, −0.5 and −0.6 ppm.

HRMS (ESI+) *m*/*z* calculated for C_27_H_32_IN_2_Si [M]^+^: 539.1374; found: 539.1374.

##### *N*-(7-(dimethylamino)-10-(3-iodophenyl)-5,5-dimethyldibenzo[*b*,*e*]silin-3(5*H*)-ylidene)-*N*-methylmethanaminium chloride (**26**)

Following general procedure A, the reaction was run using 1,3-diiodobenzene (495 mg, 1.5 mmol) and compound **5** (50 mg, 0.15 mmol). The mixture was purified by normal-phase chromatography in DCM:MeOH (95:5) to isolate the product as a blue solid (60 mg, 73%).

^1^H NMR (500 MHz, CDCl_3_): δ 7.87 (dt, *J* = 7.7, 1.5 Hz, 1H), 7.60 (t, *J* = 1.5 Hz, 1H), 7.28 (t, *J* = 7.7 Hz, 1H), 7.24 (t, *J* = 1.5 Hz, 1H), 7.22 (d, *J* = 2.9 Hz, 2H), 7.10 (d, *J* = 9.6 Hz, 2H), 6.66 (dd, *J* = 9.6, 2.8 Hz, 2H), 3.42 (s, 12H), 0.66 (s, 3H) and 0.63 (s, 3H) ppm.

^13^C NMR (126 MHz, CDCl_3_): δ 167.0, 154.2, 148.8, 142.2, 141.2, 137.9, 137.6, 130.1, 128.6, 127.9, 121.4, 114.1, 94.2, 41.6, −0.3 and −0.4 ppm.

HRMS (ESI+) *m*/*z* calculated for C_25_H_28_IN_2_Si [M]^+^: 511.1061; found: 511.1079.

##### *N*-(7-(dimethylamino)-10-(5-iodo-2,4-dimethylphenyl)-5,5-dimethyldibenzo[*b*,*e*]-silin-3(5*H*)-ylidene)-*N*-methylmethanaminium chloride (**27**)

Following general procedure A, the reaction was run using 1,5-diiodo-2,4-dimethylbenzene (537 mg, 1.5 mmol) and compound **5** (50 mg, 0.15 mmol). The mixture was purified by normal-phase chromatography in DCM:MeOH (95:5) to isolate the product as a blue solid (69 mg, 78%).

^1^H NMR (500 MHz, CDCl_3_): δ 7.52 (s, 1H), 7.24–7.18 (m, 3H), 7.10 (d, *J* = 9.6 Hz, 2H), 6.66 (dd, *J* = 9.6, 2.8 Hz, 2H), 3.42 (s, 12H), 2.50 (s, 3H), 1.95 (s, 3H), 0.64 (s, 3H) and 0.64 (s, 3H) ppm.

^13^C NMR (126 MHz, CDCl_3_): δ 167.7, 154.3, 148.6, 142.3, 141.5, 138.5, 138.0, 136.3, 131.7, 127.6, 121.2, 114.4, 97.4, 41.6, 27.9, 19.2, −0.5 and −0.6 ppm.

HRMS (ESI+) *m*/*z* calculated for C_27_H_32_IN_2_Si [M]^+^: 539.1374; found: 539.1373.

##### (*S*)-*N*-(10-(4-(3-(2-((((9*H*-fluoren-9-yl)methoxy)carbonyl)amino)-2-carboxyethyl)-1*H*-indol-2-yl)-2,5-dimethylphenyl)-7-(dimethylamino)-5,5-dimethyldibenzo[*b*,*e*]-silin-3(5*H*)-ylidene)-*N*-methylmethanaminium 2,2,2-trifluoroacetate (**28**)

Iodinated compound **25** (100 mg, 0.17 mmol, 1.0 equiv.), Fmoc-Trp-OH (73 mg, 0.17 mmol, 1.0 equiv.), AgBF_4_ (33 mg, 0.17 mmol, 1.0 equiv.) and Pd(OAc)_2_ (6.7 mg, 0.03 mmol, 0.2 equiv.) were dissolved in anhydrous DMF (3.0 ml) in a microwave reactor vessel, followed by the addition of trifluoroacetic acid (13 μl, 0.17 mmol, 1.0 equiv.). The mixture was subjected to three cycles of microwave irradiation for 30 min at 110 °C. Additional fresh AgBF_4_ (1.0 equiv.) and Pd(OAc)_2_ (0.2 equiv.) were added after each cycle. The mixture was diluted in DCM and filtered through celite. The solution was concentrated under reduced pressure and purified by reversed-phase preparative HPLC. After lyophilization, compound **28** was isolated as a blue solid (35 mg, 21%).

^1^H NMR (500 MHz, CDCl_3_): δ 8.85 (s, 1H), 7.69 (d, *J* = 7.7 Hz, 3H), 7.50–7.37 (m, 3H), 7.35–7.27 (m, 5H), 7.22–7.13 (m, 3H), 7.09–6.97 (m, 4H), 6.83–6.63 (m, 2H), 5.58 (d, *J* = 8.1 Hz, 1H), 4.53 (q, *J* = 4.9 Hz, 1H), 4.22–4.14 (m, 2H), 4.07 (t, *J* = 7.3 Hz, 1H), 3.43–3.34 (m, 1H), 3.25 (s, 6H), 3.23 (s, 6H), 3.19–3.08 (m, 1H), 2.23 (s, 3H), 1.97 (s, 3H), 0.55 (s, 3H) and 0.54 (s, 3H) ppm.

^13^C NMR (126 MHz, CDCl_3_): δ 173.6, 170.1, 155.9, 154.6, 154.1, 148.3, 147.8, 144.1, 144.0, 142.6, 142.1, 141.3, 141.2, 139.0, 136.1, 135.9, 135.0, 132.7, 131.5, 129.9, 128.8, 128.2, 128.1, 127.7, 127.2, 125.5, 125.4, 122.1, 120.5, 120.1, 119.9, 119.0, 117.4, 115.3, 114.6, 111.2, 109.0, 67.0, 55.3, 53.6, 47.1, 41.0, 40.8, 29.8, 27.9, 20.0, 19.3, −0.3 and −1.5 ppm.

HRMS (ESI+) *m*/*z* calculated for C_53_H_53_N_4_O_4_Si [M]^+^: 837.3831; found: 837.3816.

##### (*S*)-*N*-(10-(3-(3-(2-((((9*H*-fluoren-9-yl)methoxy)carbonyl)amino)-2-carboxyethyl)-1*H*-indol-2-yl)phenyl)-7-(dimethylamino)-5,5-dimethyldibenzo[*b*,*e*]silin-3(5*H*)-ylidene)-*N*-methylmethanaminium 2,2,2-trifluoroacetate (**29**)

Iodinated compound **26** (50 mg, 0.09 mmol, 1.0 equiv.), Fmoc-Trp-OH (38 mg, 0.09 mmol, 1.0 equiv.), AgBF_4_ (18 mg, 0.09 mmol, 1.0 equiv.) and Pd(OAc)_2_ (4.5 mg, 0.02 mmol, 0.2 equiv.) were dissolved in anhydrous DMF (3.0 ml) in a microwave reactor vessel, followed by the addition of trifluoroacetic acid (6.9 μl, 0.09 mmol, 1.0 equiv.). The mixture was subjected to two cycles of microwave irradiation for 30 min at 80 °C. Additional fresh AgBF_4_ (1.0 equiv.) and Pd(OAc)_2_ (0.2 equiv.) were added after each cycle. The mixture was diluted in DCM and filtered through celite. The solution was concentrated under reduced pressure and purified by reversed-phase preparative HPLC. After lyophilization, compound **29** was isolated as a blue solid (32 mg, 37%).

^1^H NMR (500 MHz, CDCl_3_): δ 9.61 (s, 1H), 7.69–7.54 (m, 4H), 7.54–7.43 (m, 2H), 7.41–7.29 (m, 6H), 7.23–7.12 (m, 4H), 7.08–6.96 (m, 4H), 6.65–6.46 (m, 2H), 5.39 (s, 1H), 4.55 (q, *J* = 6.7 Hz, 1H), 4.13–4.02 (m, 2H), 3.96 (t, *J* = 7.4 Hz, 1H), 3.51–3.42 (m, 1H), 3.30–3.27 (m, 1H), 3.19 (s, 12H), 0.52 (s, 3H) and 0.50 (s, 3H) ppm.

^13^C NMR (126 MHz, CDCl_3_): δ 173.7, 170.0, 160.8, 160.5, 160.2, 159.9, 155.9, 154.2, 154.1, 148.3, 148.2, 144.0, 143.9, 143.2, 143.1, 141.2, 139.2, 136.4, 135.4, 133.3, 129.4, 129.4, 129.1, 128.9, 128.4, 128.2, 128.2, 127.8, 127.2, 125.4, 122.5, 120.4, 119.9, 119.5, 119.0, 117.2, 114.9, 114.3, 114.2, 111.9, 107.7, 67.1, 54.8, 47.1, 40.8, 40.8, 27.4, −0.5 and −1.1 ppm.

HRMS (ESI+) *m*/*z* calculated for C_51_H_49_N_4_O_4_Si [M]^+^: 809.3518; found: 809.3522.

##### (*S*)-*N*-(10-(5-(3-(2-((((9*H*-fluoren-9-yl)methoxy)carbonyl)amino)-2-carboxyethyl)-1*H*-indol-2-yl)-2,4-dimethylphenyl)-7-(dimethylamino)-5,5-dimethyldibenzo[*b*,*e*]-silin-3(5*H*)-ylidene)-*N*-methylmethanaminium 2,2,2-trifluoroacetate (**30**)

Iodinated compound **27** (50 mg, 0.09 mmol, 1.0 equiv.), Fmoc-Trp-OH (38 mg, 0.09 mmol, 1.0 equiv.), AgBF_4_ (18 mg, 0.09 mmol, 1.0 equiv.) and Pd(OAc)_2_ (4.5 mg, 0.02 mmol, 0.2 equiv.) were dissolved in anhydrous DMF (3.0 ml) in a microwave reactor vessel, followed by the addition of trifluoroacetic acid (6.9 μl, 0.09 mmol, 1.0 equiv.). The mixture was subjected to three cycles of microwave irradiation for 30 min at 110 °C. Additional fresh AgBF_4_ (1.0 equiv.) and Pd(OAc)_2_ (0.2 equiv.) were added after each cycle. The mixture was diluted in DCM and filtered through celite. The solution was concentrated under reduced pressure and purified by reversed-phase preparative HPLC. After lyophilization, compound **30** was isolated as a blue solid (12 mg, 15%).

^1^H NMR (500 MHz, CDCl_3_): δ 8.78 (s, 1H), 7.75–7.55 (m, 4H), 7.40–7.29 (m, 6H), 7.21–6.92 (m, 8H), 6.81 (d, *J* = 9.3 Hz, 1H), 6.71 (d, *J* = 9.3 Hz, 1H), 5.43 (d, *J* = 8.0 Hz, 1H), 4.44 (q, *J* = 6.7 Hz, 1H), 3.97–3.89 (m, 3H), 3.36–3.31 (m, 1H), 3.26 (s, 6H), 3.23 (s, 6H), 3.15–3.09 (m, 1H), 2.30 (s, 3H), 2.02 (s, 3H), 0.49 (s, 3H) and 0.48 (s, 3H) ppm.

^13^C NMR (126 MHz, CDCl_3_): δ 173.6, 170.1, 161.2, 160.9, 155.9, 154.6, 154.0, 148.2, 147.6, 144.2, 144.1, 142.6, 142.0, 141.2, 141.2, 139.1, 136.1, 136.0, 135.9, 135.0, 132.6, 131.6, 130.0, 128.9, 128.2, 128.1, 127.6, 127.2, 127.1, 125.5, 125.4, 121.9, 120.5, 120.1, 119.9, 119.7, 119.1, 117.9, 115.6, 115.5, 114.6, 111.2, 109.2, 66.9, 66.0, 55.5, 47.1, 41.0, 40.8, 28.1, 20.0, 19.3, 15.4, −0.2 and −1.5 ppm.

HRMS (ESI+) *m*/*z* calculated for C_53_H_53_N_4_O_4_Si [M]^+^: 837.3831; found: 837.3805.

#### General procedures for SPPS

Peptides were manually synthesized in 10-ml polystyrene syringes fitted with porous polyethylene discs using common Fmoc-SPPS protocols^95^. Solvents, excess reagents and soluble by-products were removed by suction. Unless indicated otherwise, the Fmoc group was removed with piperidine:DMF (1:4) (1 × 1 min, 2 × 5 min), followed by DMF (5×) and DCM (5×) washes. All syntheses were carried out at room temperature. Peptides bearing fluorescent moieties were always protected from light.

##### Resin loading for 2-chlorotrityl polystyrene resin

The resin (0.25 mmol g^−1^) was transferred into a 10-ml polystyrene syringe and washed with DCM (1 × 15 min), DMF (4 × 1 min) and DCM (4 × 1 min). The first amino acid was loaded onto the resin using Fmoc-AA-OH (1.0 equiv.) and DIPEA (3.0 equiv.) in DCM for 10 min followed by additional DIPEA (7.0 equiv.) for an extra 40 min. MeOH (0.8 μl mg^−1^ resin) was added to cap the remaining trityl groups. The resin was then filtered and washed using DCM (5 × 1 min) and DMF (5 × 1 min). The loading of the resin was determined by measuring the absorbance of piperidine–dibenzofulvene adduct at 290 nm after Fmoc removal using NanoDrop as an indirect reading of the extent of amino acid coupling.

##### Peptide elongation

After the Fmoc group was removed, the resin was washed with DMF (4 × 1 min), DCM (3 × 1 min) and DMF (4 × 1 min). Automated microwave-assisted SPPS was carried out in a Liberty Blue microwave peptide synthesizer (CEM). In general, Fmoc-AA-OH (0.2 M, 4.0 equiv.), DIC (0.25 M) and OxymaPure (0.5 M) reagents were used for each amide coupling, and piperidine:DMF (1:4) was employed for the removal of Fmoc protecting groups. Amide couplings with FgAAs were carried out using Fmoc-AA-OH (1.0 equiv.), DIC (3.0 equiv.) and OxymaPure (3.0 equiv.) in DMF.

##### Cleavage from resin of peptides **22**–**24**, **31**, **33**, **35** and **36**

The peptides were cleaved from the resin using 95% TFA, 2.5% TIS and 2.5% H_2_O for 1 h and washed with DCM (4 × 1 min). The combined filtrates were collected into a round bottom flask and concentrated under reduced pressure.

##### Cleavage from resin and cyclization of peptides **32** and **34**

The peptides were cleaved from the resin using 2% TFA/DCM (5 × 1 min) and washed with DCM (4 × 1 min). The combined filtrates were collected into a round bottom flask and concentrated under reduced pressure. The crude peptide was precipitated in cold Et_2_O (dropwise), and the resulting precipitate was decanted and dried. Cleaved peptide (1.0 equiv.) was dissolved in DMF:ACN (1:1, 1.5 mM) with PyOxim (3.0 equiv.), OxymaPure (3.0 equiv.) and DIPEA (10 equiv.) and the mixture was stirred for 16 h at room temperature. The crude peptide was concentrated under reduced pressure and precipitated in cold Et_2_O (dropwise). The crude peptide was dissolved in TFA:TIS:H_2_O (95:2.5:2.5) for 1 h to remove the side-chain protecting groups. The mixture was concentrated under reduced pressure and precipitated in cold Et_2_O (dropwise).

##### H-Trp(HPF)-Lys-Lys-Arg-Phe-Ser-Phe-Lys-Lys-Phe-NH_2_ (**22**)

The synthesis was performed on 50 mg of TentaGel R RAM resin (0.20 mmol g^−1^). Protected amino acids Fmoc-Lys(Boc)-OH, Fmoc-Arg(Pbf)-OH, Fmoc-Phe-OH, Fmoc-Ser(Trt)-OH and compound **12** were used as side-chain protected building blocks. After cleavage, as described above, the crude peptide was precipitated in cold Et_2_O (dropwise), and the resulting precipitate was decanted and dried. Purification was conducted by preparative HPLC to isolate peptide **22** as an orange solid.

HPLC-MS: *t*_R_: 3.0 min (>95% purity).

HRMS(ESI+) *m*/*z* calculated for C_90_H_116_N_19_O_14_ [M+H]^+^: 1686.8944; found: 1686.8946.

MALDI *m*/*z* calculated for [M+H]^+^: 1686.8944; found: 1686.8963.

##### H-Trp(ORh)-Lys-Lys-Arg-Phe-Ser-Phe-Lys-Lys-Phe-NH_2_ (**23**)

The synthesis was performed on 50 mg of TentaGel R RAM resin (0.20 mmol g^−1^). Protected amino acids Fmoc-Lys(Boc)-OH, Fmoc-Arg(Pbf)-OH, Fmoc-Phe-OH, Fmoc-Ser(Trt)-OH and compound **13** were used as side-chain protected building blocks. After cleavage, as described above, the crude peptide was precipitated in cold Et_2_O (dropwise), and the resulting precipitate was decanted and dried. Purification was conducted by preparative HPLC to isolate peptide **23** as a red solid.

HPLC-MS: *t*_R_: 3.2 min (>95% purity).

HRMS (ESI+) *m*/*z* calculated for C_94_H_126_N_21_O_12_ [M]^+^: 1740.9889; found: 1740.9900.

MALDI *m*/*z* calculated for [M]^+^: 1740.9889; found: 1740.9903.

##### H-Trp(SiRh)-Lys-Lys-Arg-Phe-Ser-Phe-Lys-Lys-Phe-NH_2_ (**24**)

The synthesis was performed on 50 mg of TentaGel R RAM resin (0.20 mmol g^−1^). Protected amino acids Fmoc-Lys(Boc)-OH, Fmoc-Arg(Pbf)-OH, Fmoc-Phe-OH, Fmoc-Ser(Trt)-OH and compound **15** were used as side-chain protected building blocks. After cleavage as described above, the crude peptide was precipitated in cold Et_2_O (dropwise) and the resulting precipitate was decanted and dried. Purification was conducted by preparative HPLC to isolate peptide **24** as a blue solid.

HPLC-MS: *t*_R_: 3.4 min (>95% purity).

HRMS (ESI+) *m*/*z* calculated for C_96_H_132_N_21_O_11_Si [M]^+^: 1783.0179; found: 1783.0179.

MALDI *m*/*z* calculated for [M]^+^: 1783.0179; found: 1783.0178.

##### H-Trp(SiRh)-Leu-Asp-Tyr-Val-Asn-Arg-Arg-Lys-Met-Tyr-Gln-OH (**31**)

The synthesis was performed on 40 mg of 2-chlorotrityl polystyrene resin (0.25 mmol g^−1^). Protected amino acids Fmoc-Gln(Trt)-OH, Fmoc-Tyr(^*t*^Bu)-OH, Fmoc-Met-OH, Fmoc-Lys(Boc)-OH, Fmoc-Arg(Pbf)-OH, Fmoc-Asn(Trt)-OH, Fmoc-Val-OH, Fmoc-Asp(O^*t*^Bu)-OH, Fmoc-Leu-OH and compound **28** were used as side-chain protected building blocks. After cleavage, as described above, the crude peptide was precipitated in cold Et_2_O (dropwise), and the resulting precipitate was decanted and dried. The residue was dissolved in ACN:H_2_O and lyophilized. Purification was conducted by semi-preparative HPLC to isolate peptide **31** as a blue solid.

HPLC-MS: *t*_R_: 3.3 min (>95% purity).

HRMS (ESI+) *m*/*z* calculated for C_103_H_148_N_24_O_19_SSi [M+3H]^3+^: 694.6916; found: 694.6939.

MALDI *m*/*z* calculated for [M]^+^: 2082.0602; found: 2082.0883.

##### c(Trp(SiRh)-Leu-Asp-Tyr-Val-Asn-Arg-Arg-Lys-Met-Tyr-Gln) (**32**)

The synthesis was performed on 40 mg of 2-chlorotrityl polystyrene resin (0.25 mmol g^−1^). Protected amino acids Fmoc-Gln(Trt)-OH, Fmoc-Tyr(^*t*^Bu)-OH, Fmoc-Met-OH, Fmoc-Lys(Boc)-OH, Fmoc-Arg(Pbf)-OH, Fmoc-Asn(Trt)-OH, Fmoc-Val-OH, Fmoc-Asp(O^*t*^Bu)-OH, Fmoc-Leu-OH and compound **28** were used as side-chain protected building blocks. After cleavage and cyclization, as described above, the crude peptide was precipitated in cold Et_2_O (dropwise), and the resulting precipitate was decanted and dried. The residue was dissolved in ACN:H_2_O and lyophilized. Purification was conducted by semi-preparative HPLC to isolate peptide **32** as a blue solid.

HPLC-MS: *t*_R_: 3.6 min (>95% purity).

HRMS (ESI+) *m*/*z* calculated for C_103_H_146_N_24_O_18_SSi [M+3H]^3+^: 688.6881; found: 688.6893.

MALDI *m*/*z* calculated for [M]^+^: 2064.0497; found: 2064.0681.

##### H-Arg-Thr-Met-Ile-Trp(SiRh)-Tyr-D-Pro-Gly-Ala-Trp-Tyr-Lys-Arg-Ile-OH (**33**)

The synthesis was performed on 40 mg of 2-chlorotrityl polystyrene resin (0.25 mmol g^−1^). Protected amino acids Fmoc-Ile-OH, Fmoc-Arg(Pbf)-OH, Fmoc-Lys(Boc)-OH, Fmoc-Tyr(^*t*^Bu)-OH, Fmoc-Trp(Boc)-OH, Fmoc-Ala-OH, Fmoc-Gly-OH, Fmoc-D-Pro-OH, Fmoc-Met-OH, Fmoc-Thr(^*t*^Bu)-OH and compound **28** were used as side-chain protected building blocks. After cleavage as described above, the crude peptide was precipitated in cold Et_2_O (dropwise) and the resulting precipitate was decanted and dried. The residue was dissolved in ACN:H_2_O and lyophilized. Purification was conducted by semi-preparative HPLC to isolate peptide **33** as a blue solid.

HPLC-MS: *t*_R_: 3.4 min (>95% purity).

HRMS (ESI+) *m*/*z* calculated for C_116_H_164_N_25_O_18_SSi [M+4H]^4+^: 563.5519; found: 563.5545.

MALDI [M] *m*/*z* calculated for [M]^+^: 2251.1858; found: 2251.2001.

##### c(Arg-Thr-Met-Ile-Trp(SiRh)-Tyr-D-Pro-Gly-Ala-Trp-Tyr-Lys-Arg-Ile) (**34**)

The synthesis was performed on 40 mg of 2-chlorotrityl polystyrene resin (0.25 mmol g^−1^). Protected amino acids Fmoc-Ile-OH, Fmoc-Arg(Pbf)-OH, Fmoc-Lys(Boc)-OH, Fmoc-Tyr(^*t*^Bu)-OH, Fmoc-Trp(Boc)-OH, Fmoc-Ala-OH, Fmoc-Gly-OH, Fmoc-D-Pro-OH, Fmoc-Met-OH, Fmoc-Thr(^*t*^Bu)-OH and compound **28** were used as side-chain protected building blocks. After cleavage and cyclization, as described above, the crude peptide was precipitated in cold Et_2_O (dropwise), and the resulting precipitate was decanted and dried. The residue was dissolved in ACN:H_2_O and lyophilized. Purification was conducted by semi-preparative HPLC to isolate peptide **34** as a blue solid.

HPLC-MS: *t*_R_: 3.6 min (>95% purity).

HRMS (ESI+) *m*/*z* calculated for C_116_H_161_N_25_O_17_SSi [M + 3H]^3+^: 745.0632; found: 745.0681.

MALDI *m*/*z* calculated for [M]^+^: 2233.1752; found: 2233.1941.

##### H-Gly-Asn-Trp(SiRh)-Asp-Tyr-Asn-Ser-Gln-Arg-Ala-Gln-Leu-Tyr-Asn-Gln-OH (**35**)

The synthesis was performed on 40 mg of 2-chlorotrityl polystyrene resin (0.25 mmol g^−1^). Protected amino acids Fmoc-Gln(Trt)-OH, Fmoc-Asn(Trt)-OH, Fmoc-Tyr(^*t*^Bu)-OH, Fmoc-Leu-OH, Fmoc-Ala-OH, Fmoc-Arg(Pbf)-OH, Fmoc-Ser(^*t*^Bu)-OH, Fmoc-Asp(O^*t*^Bu)-OH, Fmoc-Gly-OH and compound **28** were used as side-chain protected building blocks. After cleavage as described above, the crude peptide was precipitated in cold Et_2_O (dropwise) and the resulting precipitate was decanted and dried. The residue was dissolved in ACN:H_2_O and lyophilized. Purification was conducted by semi-preparative HPLC to isolate peptide **35** as a blue solid.

HPLC-MS: *t*_R_: 3.5 min (>95% purity).

HRMS (ESI+) *m*/*z* calculated for C_107_H_146_N_27_O_27_Si [M + 2H]^2+^: 1134.0281; found: 1134.0421.

MALDI *m*/*z* calculated for [M]^+^: 2267.0489; found: 2267.0603.

##### H-Trp(SiRh)-Tyr-Arg-Cys-Met-Ile-Ser-Tyr-Gly-Gly-Ala-Asp-Tyr-Lys-Cys-Ile-Thr-OH (Cys4-Cys15 disulfide) (**36**)

The synthesis was performed on 40 mg of 2-chlorotrityl polystyrene resin (0.25 mmol g^−1^). Protected amino acids Fmoc-Thr(^*t*^Bu)-OH, Fmoc-Ile-OH, Fmoc-Cys(Trt)-OH, Fmoc-Lys(Boc)-OH, Fmoc-Tyr(^*t*^Bu)-OH, Fmoc-Asp(O^*t*^Bu)-OH, Fmoc-Ala-OH, Fmoc-Gly-OH, Fmoc-Ser(^*t*^Bu)-OH, Fmoc-Met-OH, Fmoc-Arg(Pbf)-OH and compound **28** were used as side-chain protected building blocks. After cleavage as described above, the crude peptide was precipitated in cold Et_2_O (dropwise) and the resulting precipitate was decanted and dried. The crude was dissolved in DMSO:H_2_O (2:3, 0.17 mM), and the mixture was stirred for 3 days at room temperature. Purification was conducted by semi-preparative HPLC to isolate peptide **36** as a blue solid.

HPLC-MS: *t*_R_: 3.2 min (>95% purity).

HRMS (ESI+) *m*/*z* calculated for C_118_H_163_N_24_O_25_S_3_Si [M+2H]^2+^: 1219.5532; found: 1219.5685.

MALDI: *m*/*z* calculated for [M]^+^: 2438.0991; found: 2438.1404.

### Reporting summary

Further information on research design is available in the Nature Portfolio Reporting Summary linked to this article.

## Online content

Any methods, additional references, Nature Portfolio reporting summaries, source data, extended data, supplementary information, acknowledgements, peer review information; details of author contributions and competing interests; and statements of data and code availability are available at https://doi.org/10.1038/s41557-026-02228-0.

## Supplementary information

Supplementary Information Supplementary Figs. 1–52.

Reporting Summary

Supplementary Video 1 Time-lapse fluorescence confocal microscopy images of *E. coli* after incubation with peptide **22** (25 μM, magenta). Peptide **22** was added to the cells and fluorescence images were immediately recorded. Excitation laser: 452 nm. The movie was compressed (jpeg) at 10 fps.

Supplementary Video 2 Time-lapse fluorescence confocal microscopy images of *E. coli* after incubation with peptide **23** (1 μM, yellow). Peptide **23** was added to the cells and fluorescence images were immediately recorded. Excitation laser: 554 nm. The movie was compressed (jpeg) at 10 fps.

Supplementary Video 3 Time-lapse fluorescence confocal microscopy images of *E. coli* after incubation with peptide **24** (1 μM, red). Peptide **24** was added to the cells, and fluorescence images were immediately recorded. Excitation laser: 650 nm. The movie was compressed (jpeg) at 10 fps.

Supplementary Data 1 Charge, spin and coordinates of modelled compounds in water and toluene (ground and excited states).

Supplementary Data 2 Statistical source data for all figures in the Supplementary Information.

## Source data

Source Data Fig. 1 Statistical source data for Fig. 1.

Source Data Fig. 2 Statistical source data for Fig. 2.

Source Data Fig. 3 Statistical source data for Fig. 3.

Source Data Fig. 4 Statistical source data for Fig. 4.

Source Data Fig. 5 Statistical source data for Fig. 5.

## Data Availability

Raw computational data generated in this study are available via DR-NTU at https://doi.org/10.21979/N9/ZVQ0DH (ref. ^96^). Source data are provided with this paper.
