# Supplementary material for: A general strategy towards multicolour fluorogenic peptides for wash-free bioassays
Source: Nat Chem. 2026 Aug 26;18(11):1968–78. doi: 10.1038/s41557-026-02228-0 (PMC13719488; doi:10.1038/s41557-026-02228-0)
Supplement: Supplementary file 1 [file 41557_2026_2228_MOESM1_ESM.pdf]

nature chemistry

Supplementary information

https://doi.org/10.1038/s41557-026-02228-0

A general strategy towards multicolour fluorogenic peptides for wash-free bioassays

In the format provided by the authors and unedited

Electronic Supplementary Information

A general strategy towards multicolor fluorogenic peptides for wash-free bioassays

Table of Contents

1. Computational Methods and Extended Computational Results
2. Supplementary Figures and Tables
3. Supplementary Movie Legends
4. Experimental Isotopic Patterns from MALDI-TOF-TOF Mass Spectra
5. NMR Spectra
6. Supplementary References

1. Computational Methods and Extended Computational Results

Computational Methods

General computational framework. All quantum chemical simulations were performed using the *Gaussian 16 Rev. A.01* software package,[1] utilizing Density Functional Theory (DFT) and Time-Dependent DFT (TD-DFT) for ground- and excited-state analyses, respectively. We employed the M06-2X hybrid meta-GGA functional[2] and def2-family basis sets[3] for all calculations due to their established accuracy in describing charge-transfer (CT) and excited-state processes, unless stated otherwise. To ensure numerical stability, all calculations utilized the UltraFine integration grid (99,590 points). Benchmark tests using a SuperFine grid (175,974 points) on representative PeT systems showed energy deviations of less than 0.03 eV, confirming that the UltraFine grid provides sufficient stability for this molecular set. Default *Gaussian* convergence criteria were applied (SCF = Tight, Opt = Tight), with thresholds of max force = 4.5×10^{-4} a.u., RMS force = 3×10^{-4} a.u., max displacement = 1.8×10^{-3} Å, and RMS displacement = 1.2×10^{-3} Å.

Ground-State optimizations and vertical excitations. A two-level computational strategy was implemented to maintain systematic conformational sampling while ensuring high-quality energetic descriptors. Unless stated otherwise, ground-state (S_0) geometries were first relaxed at the M06-2X/def2-SVP. All stationary points were verified as true minima via frequency analysis, ensuring the absence of imaginary frequencies. We then performed vertical excitation calculations at the M06-2X/def2-TZVP level of theory on the S_0-optimized geometries. For HPF-AA (**12**, deprotonated form), the M06-2X/6-311+G(d,p)//M06-2X/6-31G(d,p) (SMD: water) protocol was adopted. Conformational searches were conducted for flexible systems (e.g., TICT-active compounds) to locate representative minima. Solvent effects were incorporated

using the SMD continuum model[4] for geometry optimizations (equilibrium solvation) and the corrected linear-response (cLR) model[5] for vertical excitations (non-equilibrium solvation) to account for the dynamic solvent response during electronic transitions.

Excited-state geometries and energy refinement. The S_1 geometries were optimized at the M06-2X/def2-SVP level using the SMD solvation within the linear-response formalism. For these structural optimizations, the solvent environment was modeled as either water (polar) or toluene (non-polar) to capture a representative range of dielectric effects. Emissive LE/ICT states were freely optimized and confirmed as true stationary points with no imaginary frequencies. For PeT-based ET states, optimizations were performed with the donor or meso-phenyl dihedrals constrained to 90° to ensure charge separation. Frequency analysis was conducted for all such states; small imaginary frequencies (typically <100 cm^{-1}) associated with the constrained torsional coordinates were deemed numerical artifacts of the constraint rather than physical instabilities. Final excited-state energies and state ordering were obtained from single-point calculations at the M06-2X/def2-TZVP level using the state-specific cLR solvation formalism in water/toluene (SMD). This tiered protocol, optimizing geometries at the def2-SVP level and refining energies at the def2-TZVP level, was validated by performing full S_1 re-optimizations and cLR energy evaluations at the def2-TZVP level for representative systems. The fully triple-ζ calculations produced energy differences of ≤0.03 eV and identical qualitative trends, confirming that the mechanistic conclusions are robust with respect to basis-set enlargement.

Modelling of the TICT mechanism. To investigate the transformation from a bright locally excited (LE) or intramolecular charge transfer (ICT) state to a dark TICT state,

we utilize a relaxed potential energy surface (PES) scan approach.

- **Coordinate selection**: The dihedral angle (θ) of the donor fragment (e.g., diethylamino or tryptophan group) is selected as the reaction coordinate.
- **Scan protocol**: The dihedral angle is incrementally varied from its optimized LE/ICT value to 90° in ten systematic steps. At each increment, all rotatable bonds were adjusted in nine successive steps (from the LE/ICT geometries) to approach the TICT configuration progressively.
- **Optimization and energy correction strategy**: At each fixed torsion angle, the S_1 geometry was fully optimized at the M06-2X/def2-SVP level with SMD (water) using the linear-response solvation formalism, while allowing all remaining internal coordinates to relax freely. The corresponding S_1 energies were then obtained from cLR-corrected single-point calculations at the M06-2X/def2-TZVP level with SMD (water) on these optimized geometries and used to construct the S_1 potential-energy surface.
- **Root-following and state tracking**: To avoid artifacts from state crossings, optimizations and cLR energy corrections are performed for both Root = 1 and Root = 2 at every point.
- **Character validation**: The S_1 surface is constructed by following the state that maintains consistent electronic character, verified through a progressive decrease in oscillator strength, an increase in Le Bahers' charge-transfer distance (d_{CT}),[6] and a decrease in the electron–hole overlap parameter (Λ, ranging from 0 to 1).[7] Smaller f and Λ values, as well as larger d_{CT} data, indicate stronger CT character.

Modelling of PeT mechanism. PeT modelling focuses on the feasibility of electron transfer between the donor (the Trp quencher) and the fluorophore scaffold, primarily

using frontier molecular orbital (FMO) analysis and discrete state optimizations.

- Molecular Orbital Descriptor (ΔE): The energy gap between the relevant electron donor (D) and acceptor (A) orbitals, typically HOMO-1 and HOMO, is calculated at the ground-state geometry in water.
- Threshold for Quenching: A frontier-orbital gap of approximately < 0.6 eV is used as a descriptor for strong electron-transfer propensity.[8] The calculated ΔE values at the M06-2X/def2-TZVP (SMD) level showed good agreement with those obtained at the M06-2X/def2-SVP (SMD) level. For consistency with the original ΔE descriptor, the latter values are reported.[8]
- Discrete State Optimizations: PeT systems are modelled by optimizing two distinct electronic configurations at the M06-2X/def2-SVP level using the SMD solvation model (in both water and toluene) within the linear-response (LR) formalism:
    - LE/ICT State: Optimized freely to represent the emissive state, unless stated otherwise.
    - ET (Electron Transfer) State: Optimized with the meso-phenyl or donor dihedral fixed at 90° to ensure complete electronic decoupling and a representative charge-separated character.
- Driving Force Calculation: The driving energy (ΔE(ET-LE)) is determined by comparing the cLR-corrected M06-2X/def2-TZVP single-point energies of the relaxed LE state relative to the optimized ET state.
- Counterion Effects: In water, the high dielectric constant provides strong electrostatic screening and strongly favors dissociation of ionic pairs, substantially weakening tight counterion association. Accordingly, no explicit counterion was included in the aqueous calculations, and the cationic fluorophores were treated as dissociated solvated species within the continuum

solvent model. In contrast, in nonpolar solvents such as toluene, dielectric screening is much weaker, and ion pairing is expected to remain significant. Therefore, an explicit Cl^- counterion was included near the cationic center in the toluene calculations to provide a more realistic description of the charged system in a low-polarity medium.

- Protonation/Deprotonation Effects: For HPF-AA (**12**), both the protonated and deprotonated forms were examined in water because the phenolic OH group can undergo acid–base equilibrium in aqueous media, making both species potentially relevant in biological environments. By contrast, in nonpolar solvents such as toluene, deprotonation is not expected to be favorable, and only the protonated form was considered. The protonated (neutral) form was treated using the same basis-set protocol adopted for the other neutral probes. For the deprotonated (anionic) form, the single-point energy calculations were carried out with a basis set including diffuse functions (M06-2X/6-311+G(d,p)), as required for a more reliable description of the more spatially extended electron density of the anionic species.

Analysis of solvation models and limitations. While we primarily report results using the corrected linear-response (cLR) formalism,[5] we performed a sensitivity analysis using the more recent cLR^2 model.[9] The cLR model is a well-established state-specific solvation correction that accounts for the dynamic response of the solvent to the excited-state density. In contrast, cLR^2 attempts to reconcile linear-response and state-specific formalisms by merging dynamic responses with relaxed-density corrections. cLR^2 systematically shifts absolute driving forces by predicting reduced stabilization of the TICT (and ET) states relative to cLR. Because cLR^2 is a perturbative approximation, it lacks fully self-consistent relaxation and may underestimate solvent

relaxation energies in systems exhibiting extreme charge reorganization.[10] Conversely, while cLR captures substantial solvent relaxation, it can over-stabilize fully charge-separated states (ET/TICT) relative to locally excited (LE) states.[9]

Despite these quantitative variances, the qualitative mechanistic conclusions, such as state ordering and the identification of the S_1 potential energy surface, remain consistent across both formalisms. Consequently, the computed energies in this work should be interpreted as semi-quantitative descriptors of relative energetic accessibility rather than absolute experimental values.

Visualization and analysis. Molecular structures and orbitals were visualized using Avogadro.[11] Electron–hole distributions and charge-transfer characteristics were analyzed with Multiwfn.[12] and visualized using IQmol and VMD.[13]

Extended Computational Results

Analysis of the S_1 potential energy surface of coumarin-AA (10)

We examined the first excited-state (S_1) potential energy surfaces (PESs) for both the diethylamino and Trp torsions of coumarin-AA (**10**) in water (Extended Computational Figures 1 and 2). The torsional scan of the diethylamino group shows no stable TICT minimum and a high rotational barrier. In contrast, the Trp torsion exhibits a well-defined TICT minimum with a relatively low barrier (0.16 eV). Accordingly, the Trp pathway represents the dominant TICT route in this series of molecules.

Analysis of the electron–hole distributions, along with the HOMO and LUMO plots, indicates that the Trp fragment serves as the primary electron donor during photoexcitation (i.e., the LE/ICT state). In contrast, the diethylamino group shows minimal changes in charge density. These results demonstrate that the Trp torsion facilitates charge transfer more effectively than the diethylamino rotation.

Overall, these calculations confirm that the Trp torsion represents the most favorable and representative TICT pathway for this series of compounds.

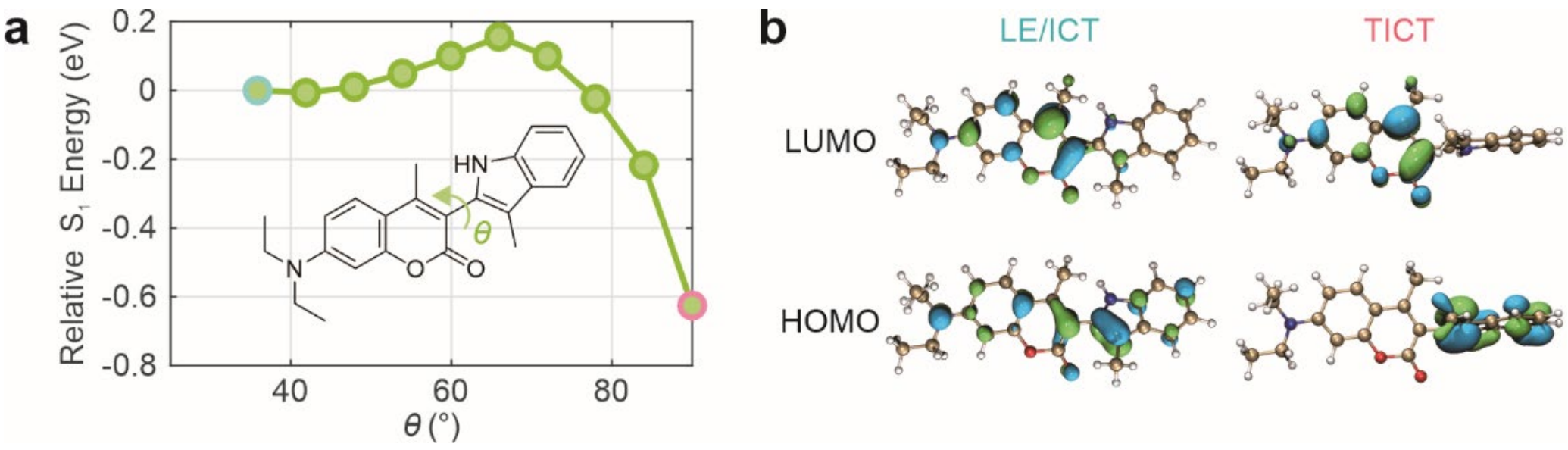

Extended Computational Figure 1. S_1 potential energy surface (PES) of compound coumarin-AA (**10**) as a function of the rotational angle (*θ*) of the Trp fragment towards the formation of twisted intramolecular charge transfer (TICT) state in water. The inset shows the molecular structure of compound coumarin-AA (**10**), highlighting the *θ* angle. The right panel shows the HOMO and LUMO distributions in the LE/ICT and TICT states. This PES exhibits an energy barrier of 0.16 eV and a driving energy of 0.63 eV from the LE/ICT to TICT states. The S_1 geometries were optimized at the M06-2X/def2-SVP level using the SMD solvation model within the linear-response (LR) formalism. Final single-point excited-state energies were calculated at the M06-2X/def2-TZVP level using the state-specific cLR formalism in conjunction with the SMD model in water.

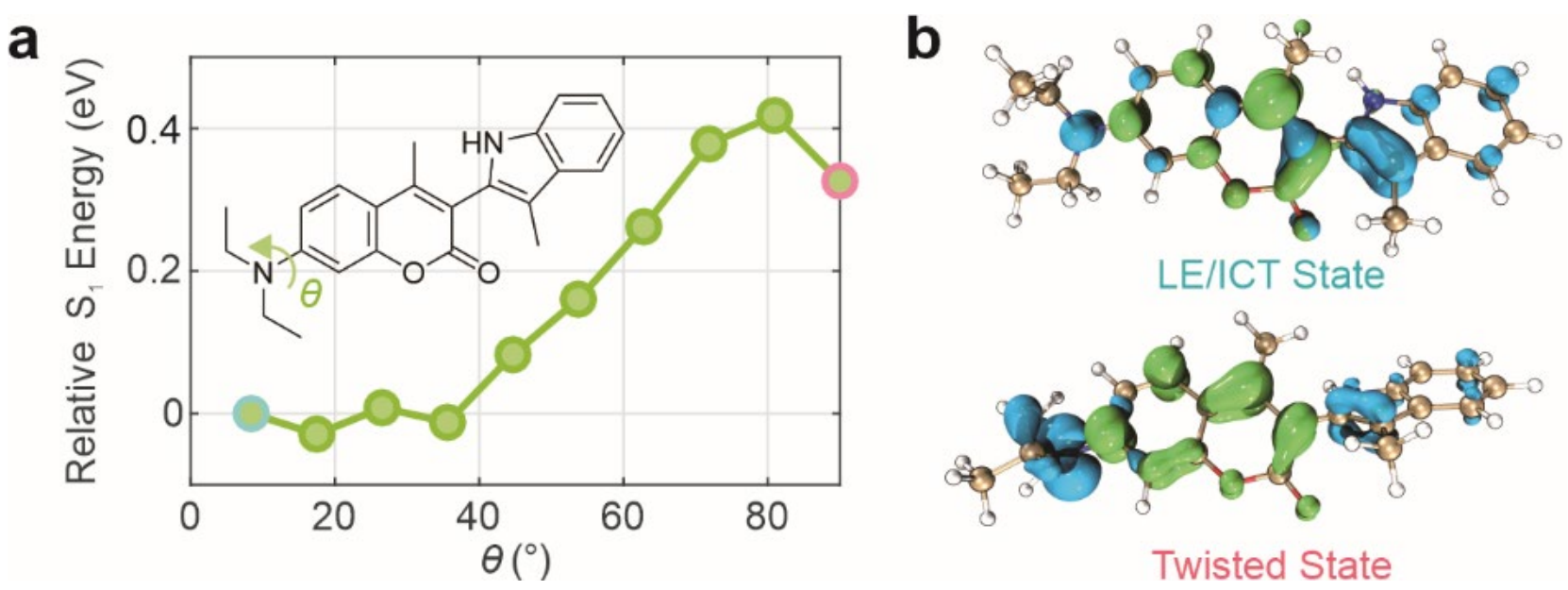

Extended Computational Figure 2. S_1 potential energy surface (PES) of compound coumarin-AA (**10**) as a function of the rotational angle (θ) of the diethylamino fragment towards the formation of twisted intramolecular charge transfer (TICT) state in water. The inset shows the molecular structure of compound coumarin-AA (**10**), highlighting the θ angle. The right panel shows the electron (green) and hole (blue) distributions in the LE/ICT and the twisted states. No stable TICT state is located. The S_1 geometries were optimized at the M06-2X/def2-SVP level using the SMD solvation model within the linear-response (LR) formalism. Final single-point excited-state energies were calculated at the M06-2X/def2-TZVP level using the state-specific cLR formalism in conjunction with the SMD model in water.

Solvent effect on PeT: a comparison between water and toluene

We compared the driving energy for the transition from the locally excited (LE) state to the electron-transfer (ET) state, ΔE(ET–LE), in water and toluene, representing polar and non-polar environments, respectively. In water, ΔE(ET–LE) becomes substantially more negative due to the strong stabilization of the ET state. This indicates that photoinduced electron transfer (PeT) is highly favourable in polar media, consistent with the experimentally observed fluorescence quenching of PeT-based probes. In contrast, in toluene, the ET state is much less stabilized, and ΔE(ET–LE) becomes less negative. Under these conditions, PeT is suppressed, allowing strong fluorescence from the LE state. This behaviour aligns with the observed fluorescence turn-on when probes bind within nonpolar environments. Altogether, these results show that PeT proceeds efficiently in polar solvents, whereas in nonpolar media the reduced stabilization of the ET state diminishes PeT efficiency and restores fluorescence.

Extended Computational Table 1. Frontier molecular orbital-derived descriptor, ΔE = E($HOMO_A$) − E($HOMO_D$), in water, together with the energies (eV) and oscillator strengths (*f*) of the Franck–Condon (FC), locally-excited (LE), and electron-transfer (ET) states for the reported probes, as well as the corresponding driving energy ΔE(ET-LE) in water and toluene.

**Probe**	**Water**					**Toluene**			
	**Δ*E* (eV)**	**FC state (eV), *f***	**LE state (eV), *f***	**ET state (eV), *f***	**ΔE(ET –LE) (eV)**	**FC state (eV), *f***	**LE state (eV), *f***	**ET state (eV), *f***	**ΔE(ET –LE) (eV)**
BODIPY-AA (**11**)	-0.09	2.96, *0.90*	2.93, *0.87*	1.94, *0.00*	-0.99	2.95, *0.67*	2.93, *0.87*	2.72, *0.00*	-0.22
HPF-AA (**12**, protonated form)	-0.28	3.28, *1.21*	3.05, *1.08*	1.95, *0.00*	-1.10	3.30, *0.75*	3.01, *0.58*	2.99, *0.00*	-0.02
HPF-AA (**12**, deprotonated form)	0.52	3.05, *0.95*	2.96, *0.85*	2.28, *0.00*	-0.67	-	-	-	-
O-rhodamine-AA (**13**)	0.01	2.87, *1.47*	2.80, *1.43*	1.87, *0.00*	-0.94	2.73, *0.95*	2.64, *0.83*	2.60, *0.00*	-0.04
Si-rhodamine-AA (**15**)	0.15	2.51, *1.54*	2.48, *1.49*	1.60, *0.00*	-0.89	2.53, *1.18*	2.49, *1.15*	2.30, *0.00*	-0.19
Si-rhodamine-AA (**28**)	0.18	2.50, *1.53*	2.47, *1.48*	1.68, *0.01*	-0.80	2.52, *1.16*	2.49, *1.13*	2.33, *0.01*	-0.16
Si-rhodamine-AA (**29**)	0.19	2.50, *1.52*	2.47, *1.47*	1.64, *0.04*	-0.83	2.52, *1.17*	2.49, *1.14*	2.32, *0.05*	-0.17
Si-rhodamine-AA (**30**)	0.17	2.50, *1.53*	2.47, *1.48*	1.59, *0.01*	-0.88	2.52, *1.15*	2.49, *1.12*	2.22, *0.02*	-0.26
carbopyronine-AA	0.21	2.64, *1.52*	2.62, *1.48*	1.82, *0.00*	-0.80	2.63, *1.13*	2.59, *1.10*	2.47, *0.00*	-0.13
phenyl-Cy3-AA	0.22	3.12, *1.98*	2.91, *1.67*	2.48, *0.00*	-0.43	3.43, *1.80*	3.35, *1.81*	3.17, *0.00*	-0.18
phenyl-Cy5-AA	0.63	2.74, *2.14*	2.68, *2.11*	2.11, *0.00*	-0.57	2.77, *2.04*	2.69, *1.96*	2.62, *0.00*	-0.07

Experimental spectroscopy was performed in ethanol because several probes have limited aqueous solubility, and improved solubility in ethanol enables more reliable absorption and fluorescence measurements. By contrast, the computational analysis employed water as the continuum solvent to better represent the aqueous biological environment relevant to probe deployment. To evaluate the effect of this solvent choice, representative calculations were also carried out using an ethanol-parameterized SMD model. Only minor changes in the LE/ET energetic balance were observed, and the relative state ordering remained unchanged. This is consistent with experimental observations that the probes already exhibit low fluorescence quantum yields in ethanol, indicating that PeT quenching is operative under the measurement conditions. The calculations further show that the ET state remains lower in energy than the LE state in both solvents, with a slightly larger driving force in water, supporting that the mechanistic conclusions are unaffected when using water in the calculations.

Extended Computational Table 2. Comparison of the calculated energy levels of the locally excited (LE) and electron-transfer (ET) states, together with the resulting driving energy, ΔE(ET–LE) (eV), for O-rhodamine-AA (**13**) and Si-rhodamine-AA (**15**) in water and ethanol.

Probe	Solvent	LE (eV)	ET (eV)	ΔE(ET–LE) (eV)
O-rhodamine-AA (**13**)	water	2.80	1.87	-0.94
	ethanol	2.81	1.91	-0.90
Si-rhodamine-AA (**15**)	water	2.48	1.60	-0.89
	ethanol	2.48	1.62	-0.86

PeT data representation. To facilitate interpretation of the results and clarify how data are presented for PeT-based dyes, the Extended Computational Figure 3 summarizes the computational conditions adopted in this study. All subsequent PeT results are displayed in the same format for consistency.

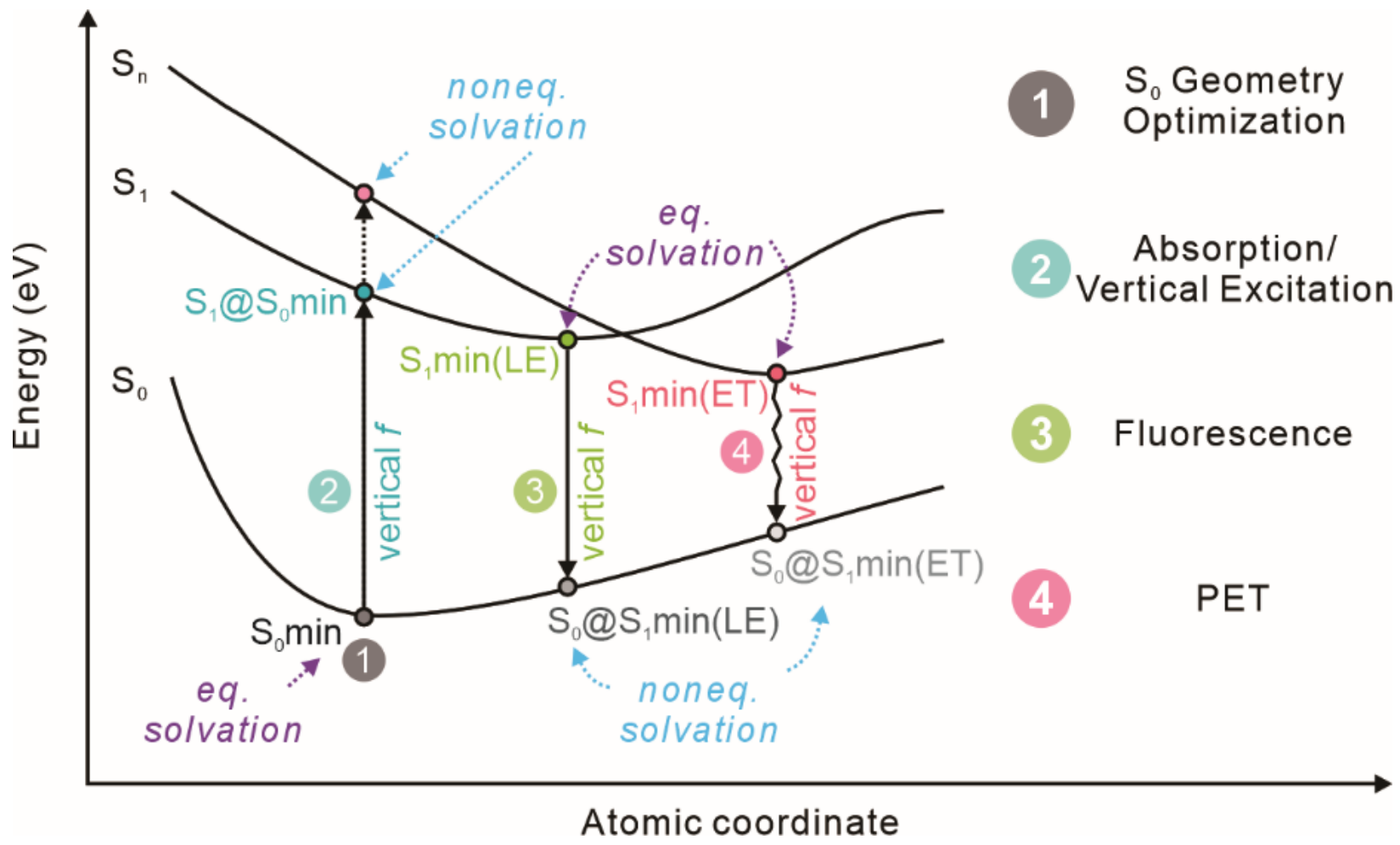

Extended Computational Figure 3. Stage-wise computational workflow for modeling PeT. (1) Ground-state optimization and frequency verification. (2) Vertical excitation calculations (Franck–Condon regime). (3) LE-state optimization and emission energy evaluation. (4) ET-state optimization and energetic analysis.

State tracking in TICT modelling

Together, these diagnostics provide mutually consistent evidence that the TICT pathway is being correctly followed in our modelling and that the increase in CT character with donor twisting is robustly captured.

Extended Computational Table 3. Evolution of charge-transfer descriptors during excited-state geometry optimization for coumarin-AA (**10**) and oxazone-AA (**14**) along the TICT pathways. At each optimization step, the tracked excited state is characterized by the Le Bahers charge-transfer distance (d_{CT}), the electron–hole overlap index (Λ), and the corresponding oscillator strength (f). Trends in these descriptors (larger d_{CT}, smaller Λ, and decreasing f) are used to monitor the transition from LE/ICT to TICT character.

**Step**	**coumarin-AA (10)**			**oxazone-AA (14)**		
	d_{CT}	Λ	f	d_{CT}	Λ	f
1	1.152	0.716	1.399	1.694	0.716	1.713
2	1.165	0.704	1.371	1.686	0.703	1.678
3	1.136	0.687	1.332	3.107	0.621	0.100
4	1.029	0.671	1.292	3.605	0.614	0.118
5	0.849	0.654	1.245	4.119	0.603	0.132
6	2.959	0.581	0.219	4.695	0.562	0.142
7	3.518	0.559	0.236	5.188	0.496	0.139
8	4.189	0.497	0.241	5.689	0.389	0.149
9	4.717	0.357	0.239	5.921	0.251	0.090
10	4.689	0.178	0.007	5.945	0.119	0.002

Effect of solvation formalism on TICT stabilization: comparison of cLR and cLR²

The choice of solvation formalism can significantly influence the quantitative stabilization of ET/TICT states. We therefore evaluated both cLR and cLR^2 for the TICT systems as part of a direct sensitivity analysis to compare their effects explicitly (Extended Computational Figures 4 and 5).

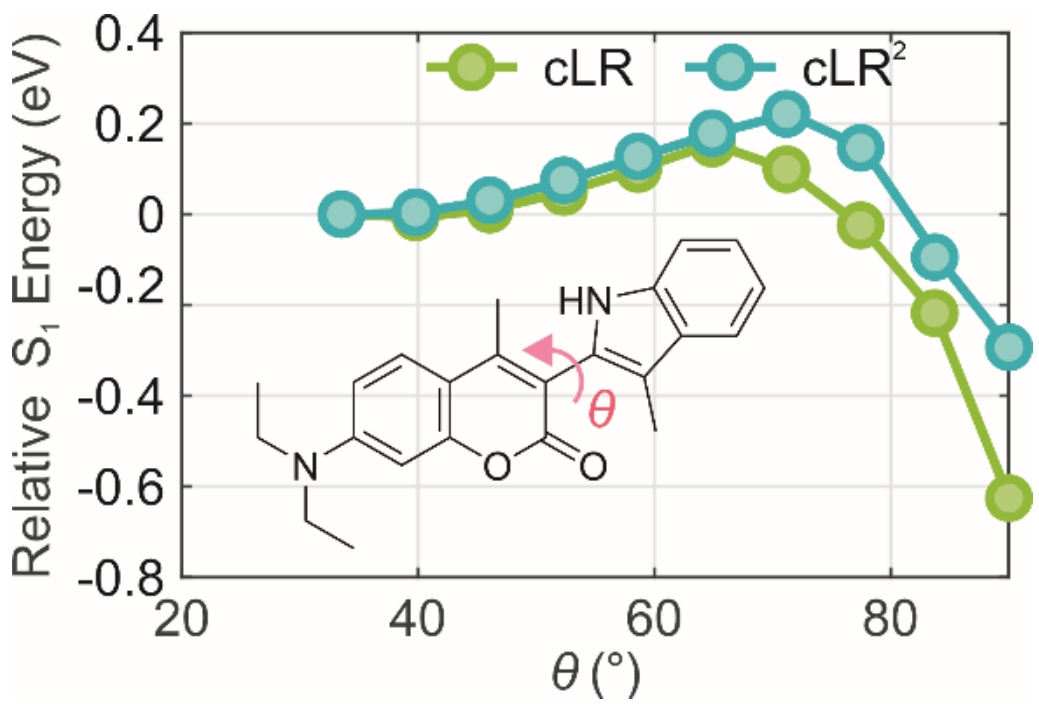

Extended Computational Figure 4. S_1 potential energy surfaces (PESs) of coumarin-AA (**10**) in water as a function of the rotational angle (θ) of the Trp fragment, illustrating the transformation from the bright locally excited (LE) or intramolecular charge transfer (ICT) state to the dark twisted intramolecular charge transfer (TICT) state. The S_1 geometries were optimized at the M06-2X/def2-SVP level using the SMD solvation model within the linear-response (LR) formalism. Final excited-state energies and state ordering were then obtained from single-point calculations at the M06-2X/def2-TZVP level using the cLR or cLR2 solvation formalism in water (SMD).

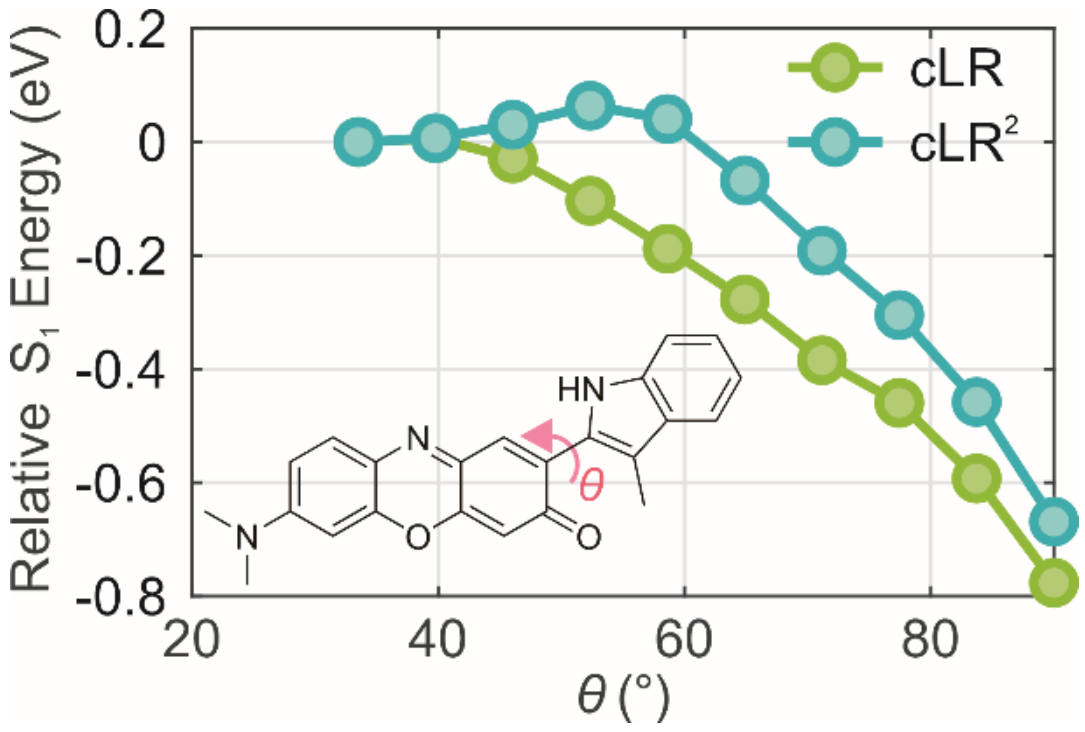

Extended Computational Figure 5. S_1 potential energy surfaces (PESs) of oxazone-AA (**14**) in water as a function of the rotational angle (*θ*) of the Trp fragment, illustrating the transformation from the bright locally excited (LE) or intramolecular charge transfer (ICT) state to the dark twisted intramolecular charge transfer (TICT) state. The S_1 geometries were optimized at the M06-2X/def2-SVP level using the SMD solvation model within the linear-response (LR) formalism. Final excited-state energies and state ordering were then obtained from single-point calculations at the M06-2X/def2-TZVP level using the cLR or cLR^2 solvation formalism in water (SMD).

Extended Computational Table 4. Relative energetics of the ICT and TICT geometries for coumarin-AA (**10**) and oxazone-AA (**14**) in water, computed using the cLR and cLR^2 solvation models. Driving energy is reported as E_d = E(TICT) − E(ICT), where negative values indicate that the TICT state is more stable. The corresponding activation barrier (E_b) from ICT to TICT is also listed.

**Probe**	**Solvent-Effect Correction Model**	**E_d (eV)**	**E_b (eV)**
coumarin-AA (**10**)	cLR	-0.63	0.16
	cLR^2	-0.29	0.22
oxazone-AA (**14**)	cLR	-0.78	0.01
	cLR^2	-0.67	0.06

The cLR^2 formalism generally predicts reduced stabilization of the TICT state and increased energy barriers compared with cLR, and the two approaches therefore differ quantitatively. However, the qualitative mechanistic trends remain unchanged across our systems: although the absolute driving forces shift, both approaches support the same underlying photophysical picture of TICT-mediated deactivation.

Given the known limitations of both cLR and cLR^2, the computed energies reported here should therefore be regarded as semi-quantitative descriptors of relative energetic accessibility, rather than as absolute experimental quantities.

Benchmarking the impact of specific solvent effects

In our excited-state modeling, we primarily employed implicit solvation models (SMD) to capture the bulk electrostatic response of the solvent, without explicitly accounting for short-range, directional solute–solvent interactions such as hydrogen bonding, a well-recognized limitation of continuum models. Our use of water as the solvent was therefore intended not to provide a microscopic description of hydration, but rather to evaluate the relative energetic accessibility of the LE and ET pathways under polar conditions, where the dielectric response is expected to dominate.

To directly probe the potential influence of specific solute–water interactions, we have now performed cluster–continuum microsolvation tests, introducing one explicit water molecule in a hydrogen-bonded configuration adjacent to the Trp donor fragment. These structures were optimized at the M06-2X/def2-SVP level and refined using single-point calculations at M06-2X/def2-TZVP with continuum solvation. Calculations were carried out for two representative probes, and the key excited-state energies are summarized below (Extended Computational Figures 6 and 7).

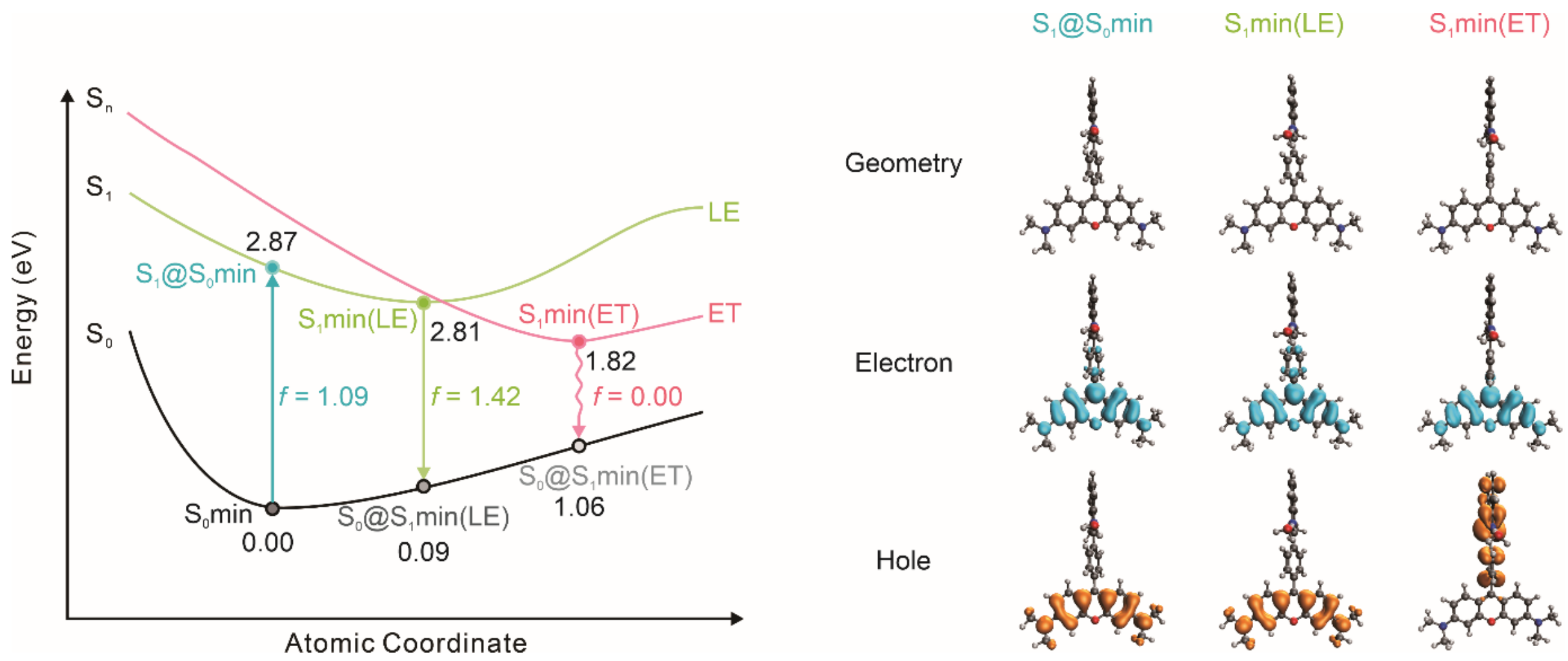

Extended Computational Figure 6. Calculated excitation/de-excitation energy (including vertical oscillator strength *f*) of compound O-rhodamine-AA (**13**), including one explicitly hydrogen-bonded water molecule embedded in an implicit-solvent environment. The right panel shows optimized molecular structures in representative states, along with the corresponding electron and hole distributions. LE: Locally Excited state; ET: Electron Transfer state. The driving energy from the LE (bright) state to the ET (dark) state decreases from water to toluene, indicating that PeT is suppressed in nonpolar solvents. For clarity, the energy levels are not plotted to scale. The S_1 geometries were optimized at the M06-2X/def2-SVP level using the SMD solvation model within the linear-response (LR) formalism. Final single-point excited-state energies were calculated at the M06-2X/def2-TZVP level using the state-specific cLR formalism in conjunction with the SMD model in water.

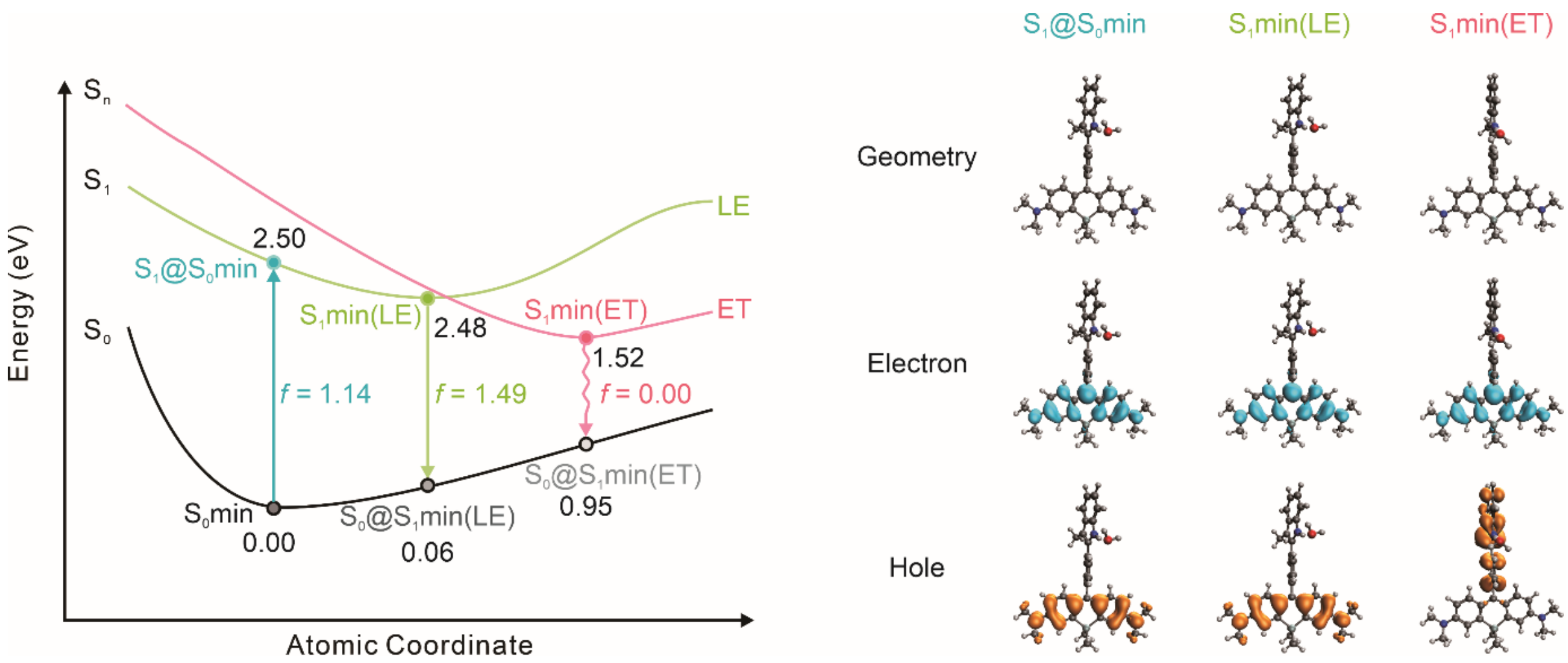

Extended Computational Figure 7. Calculated excitation/de-excitation energy (including vertical oscillator strength *f*) of compound Si-rhodamine-AA (**15**), including one explicitly hydrogen-bonded water molecule embedded in an implicit-solvent environment. The right panel shows optimized molecular structures in representative states, along with the corresponding electron and hole distributions. LE: Locally Excited state; ET: Electron Transfer state. The driving energy from the LE (bright) state to the ET (dark) state decreases from water to toluene, indicating that PeT is suppressed in nonpolar solvents. For clarity, the energy levels are not plotted to scale. The S_1 geometries were optimized at the M06-2X/def2-SVP level using the SMD solvation model within the linear-response (LR) formalism. Final single-point excited-state energies were calculated at the M06-2X/def2-TZVP level using the state-specific cLR formalism in conjunction with the SMD model in water.

Extended Computational Table 5. Comparison of the locally excited (LE) and electron-transfer (ET) state energies, and the corresponding driving force, ΔE(ET-LE), computed in water using either implicit solvation alone or a cluster–continuum microsolvation model including one explicit hydrogen-bonded water molecule at the Trp donor site. Energies are reported in eV.

**Probe**	**LE (implicit) (eV)**	**LE (explicit + implicit) (eV)**	**ET (implicit) (eV)**	**ET (explicit + implicit) (eV)**	**ΔE(ET-LE) (implicit) (eV)**	**ΔE(ET-LE) (explicit + implicit) (eV)**
O-rhodamine-AA (**13**)	2.80	2.81	1.87	1.82	-0.94	-0.98
Si-rhodamine-AA (**15**)	2.48	2.48	1.60	1.52	-0.89	-0.96

In both systems, inclusion of an explicit hydrogen-bonded water molecule produces only small quantitative changes (0.04-0.07 eV) in the LE–ET driving force, ΔE(ET-LE). These differences are minor compared with the absolute magnitude of the driving force (~0.9 eV), and the qualitative state ordering and mechanistic interpretation remain unchanged. These data suggest that hydrogen bonding plays a relatively minor role compared to the solvent dielectric in stabilizing ET states.

Consistent with this, the experimental polarity-dependent quenching behaviour does not indicate strong site-specific coordination effects but is instead compatible with a predominantly dielectric solvent response[8]. We also note that our previous studies on related PeT-based probes showed similarly strong polarity-dependent quenching, with no evidence of hydrogen-bond-dominated deactivation pathways.

Taken together, these computational benchmarks and experimental observations indicate that, for the present systems, continuum solvation provides an adequate description of the dominant solvent–response contributions to LE–ET energetics. Explicit hydrogen-bonding effects may slightly modulate the absolute energies, but they do not alter the qualitative state ordering or the mechanistic conclusions drawn in this work.

Benchmarking basis set and integration grid effects

Basis-set quality is particularly relevant for (i) twisted CT/TICT states, where the donor and acceptor π-systems become partially decoupled, and (ii) PeT/CT states, where substantial charge redistribution can increase sensitivity to polarization and, in some cases, diffuse character.

For the use of basis sets, we employ a two-level strategy, in which geometries are optimized at the lower-cost level (def2-SVP), and the energetic descriptors and state ordering are obtained from subsequent single-point calculations at the def2-TZVP level, unless stated otherwise.

In addition, all reported calculations were performed on the UltraFine grid, which we chose to balance numerical robustness and computational cost across the full molecular set. We have also repeated representative calculations for two PeT-type systems and two TICT-type systems using the SuperFine grid and compared the resulting excited-state energetics and state ordering with the UltraFine-grid results.

Specifically, to directly address the possibility of basis-set and integration grid artifacts in TICT and ET states, we performed the following validation tests:

1. Higher-level geometry benchmarks. For the two TICT systems and the two representative PeT systems, we re-optimized the excited-state geometries at the M06-2X/def2-TZVP level (with a SuperFine integration grid) and compared the resulting energetics with those from the production protocol (def2-SVP geometry optimization followed by def2-TZVP single-point calculations; with an UltraFine integration grid).
2. Consistent state-identity checks. In all cases, the same physical states were confirmed by inspecting the frontier molecular orbitals/charge-transfer descriptors to

ensure a meaningful comparison.

The results are summarised in the Extended Computational Table 6. The TICT/ET and LE/ICT energy differences obtained from the two protocols differ only marginally, with deviations of no more than 0.03 eV. Importantly, the state ordering and the qualitative mechanistic trends remain unchanged for all benchmark systems. These results indicate that, although def2-SVP is not intended for fully quantitative treatment of TICT or ET energetics, its use for excited-state geometry relaxation within a two-level protocol does not introduce qualitative bias into the mechanistic conclusions reported here. The computed energetics are also largely insensitive to the choice of integration grid (UltraFine vs. SuperFine). Accordingly, all key energetic comparisons in this work are based on the two-level strategy (def2-SVP geometries with def2-TZVP single-point energies, unless stated otherwise) in combination with the UltraFine grid.

Extended Computational Table 6. Comparison of the LE/ICT and TICT/ET state energies in water obtained using (i) a two-level protocol (M06-2X/def2-SVP S_1 geometry optimization followed by M06-2X/def2-TZVP single-point cLR calculations; with an UltraFine grid) and (ii) a higher-level protocol (M06-2X/def2-TZVP S_1 geometry optimization followed by M06-2X/def2-TZVP single-point cLR calculations; with a SuperFine grid). The corresponding driving energy is reported as ΔE = E(ET/TICT) − E(LE/ICT).

**Probe**	**LE/ICT State (def2-SVP/def2-TZVP; UltraFine grid) (eV)**	**LE/ICT State (def2-TZVP; SuperFine grid) (eV)**	**ET/TICT State (def2-SVP/def2-TZVP; UltraFine grid) (eV)**	**ET/TICT State (def2-TZVP; SuperFine grid) (eV)**	**Δ*E*(ET/TICT-LE/ICT) (def2-SVP/def2-TZVP; UltraFine grid) (eV)**	**Δ*E*(ET/TICT-LE/ICT) (def2-TZVP; SuperFine grid) (eV)**
coumarin-AA (**10**)	3.28	3.28	2.66	2.63	-0.63	-0.66
oxazone-AA (**14**)	2.46	2.46	1.68	1.69	-0.78	-0.77
O-rhodamine-AA (**13**)	2.80	2.81	1.87	1.84	-0.94	-0.96
Si-rhodamine-AA (**15**)	2.48	2.48	1.60	1.56	-0.89	-0.92

2. Supplementary Figures and Tables

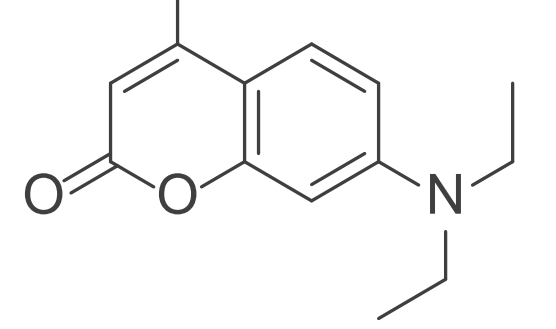

16

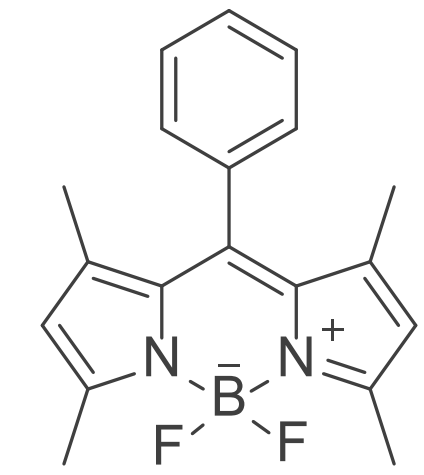

17

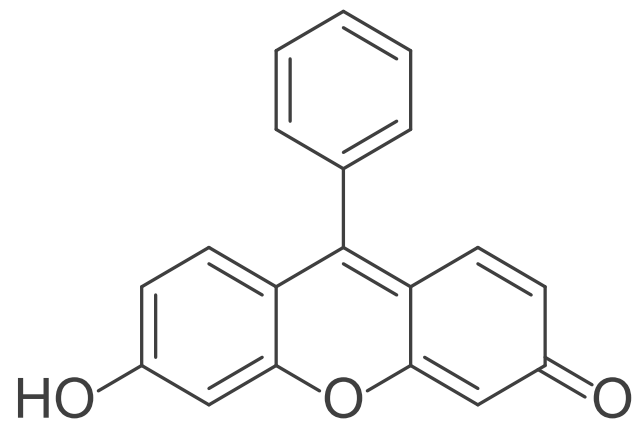

18

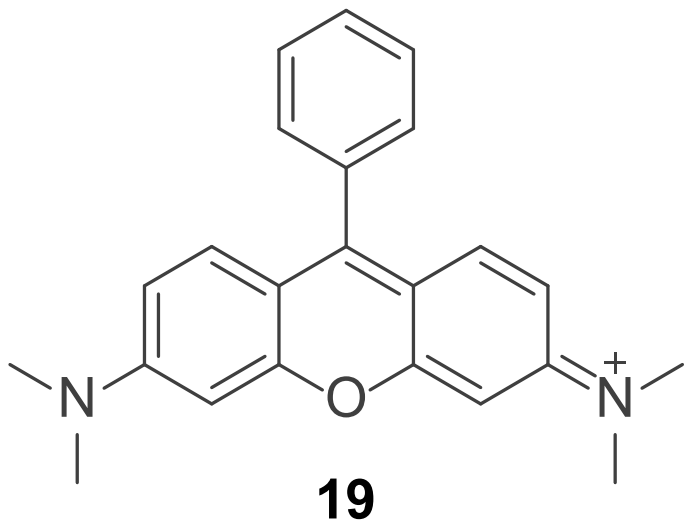

19

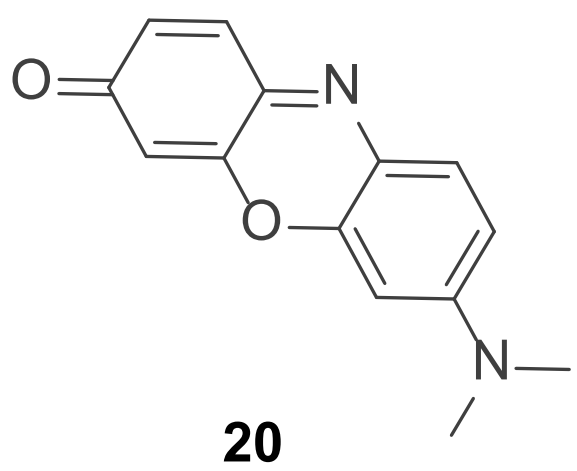

20

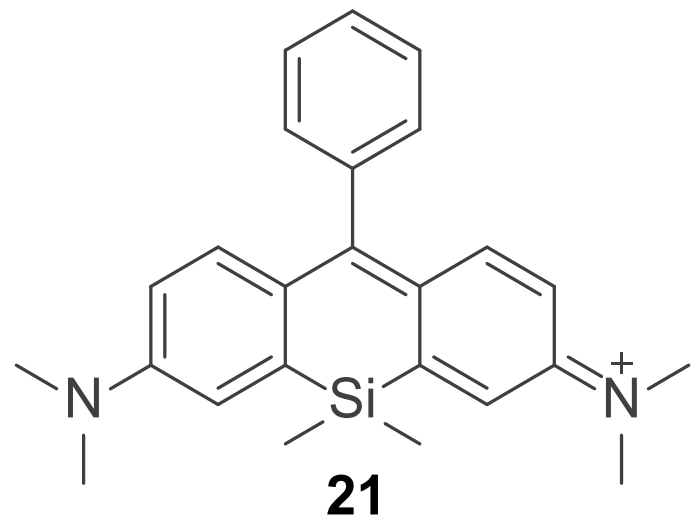

21

Supplementary Figure 1. Chemical structures of parent fluorophores **16-21**.

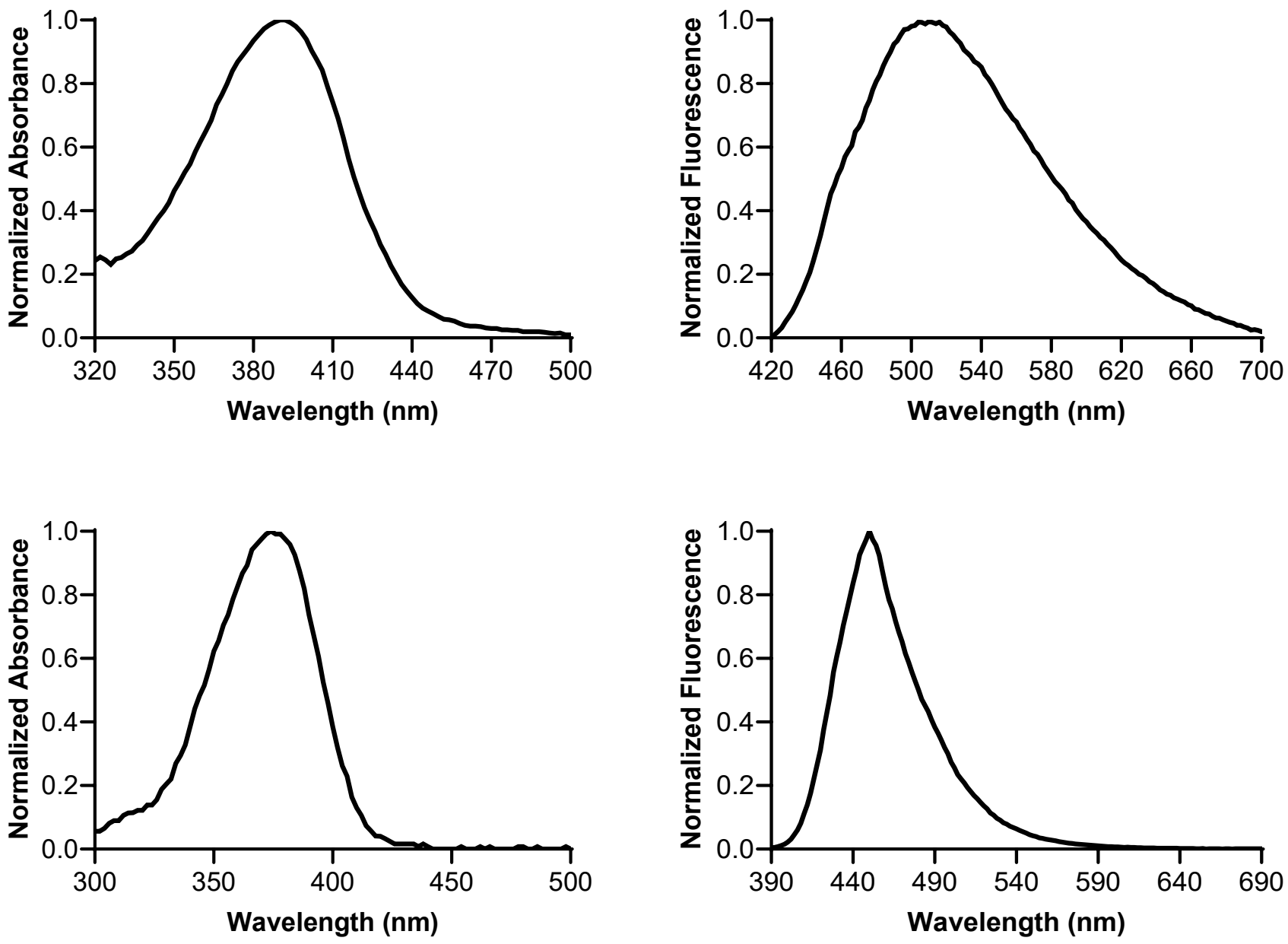

Supplementary Figure 2. Representative (from 3 independent experiments) normalized absorption and emission spectra of compounds **10** (30 μM, top) and **16** (30 μM, bottom) in EtOH. λ_{exc}: 390 nm (for **10**) and 360 nm (for **16**).

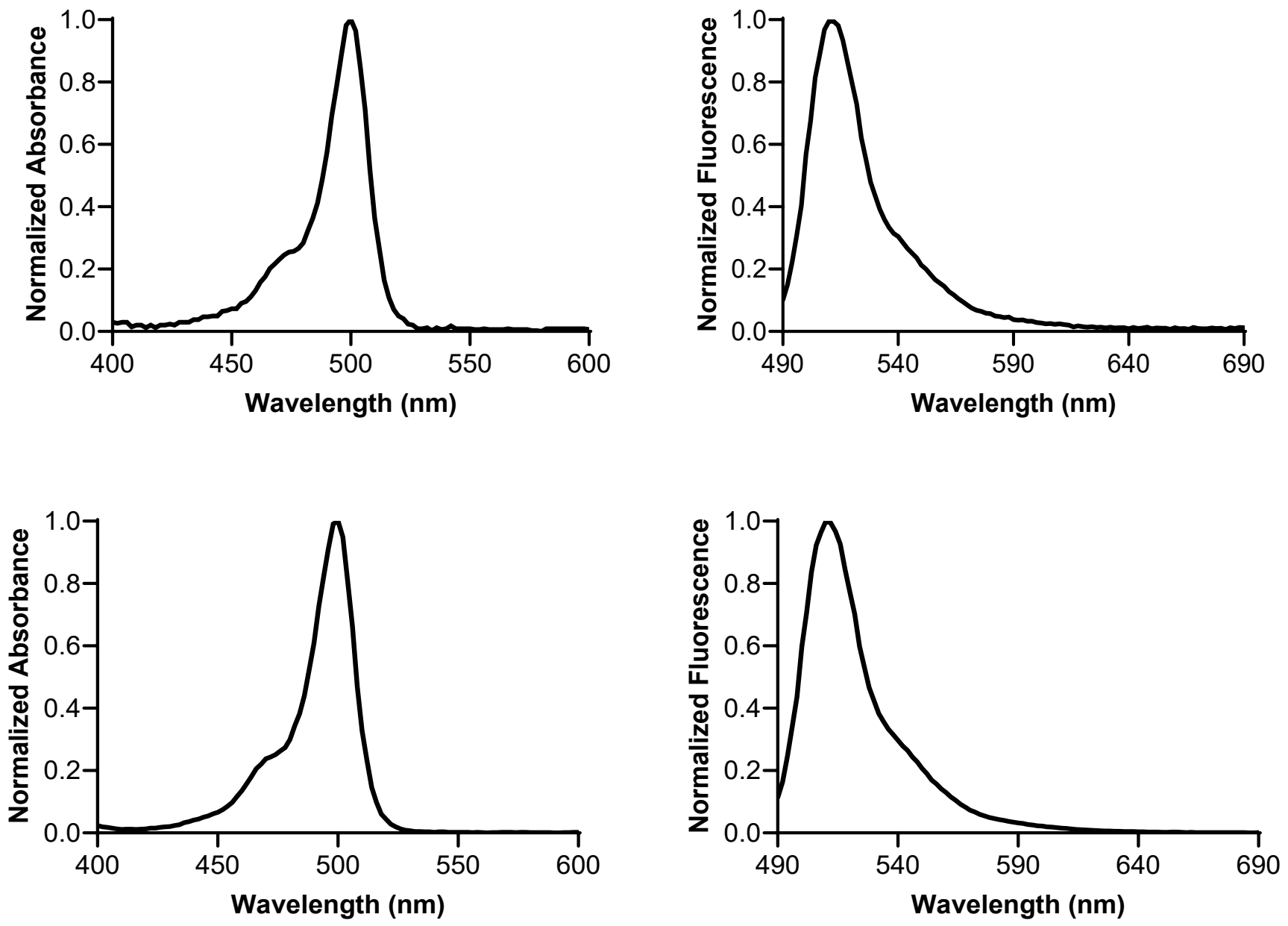

Supplementary Figure 3. Representative (from 3 independent experiments) normalized absorption and emission spectra of compounds **11** (10 µM, top) and **17** (10 µM, bottom) in EtOH. λ_{exc}: 460 nm.

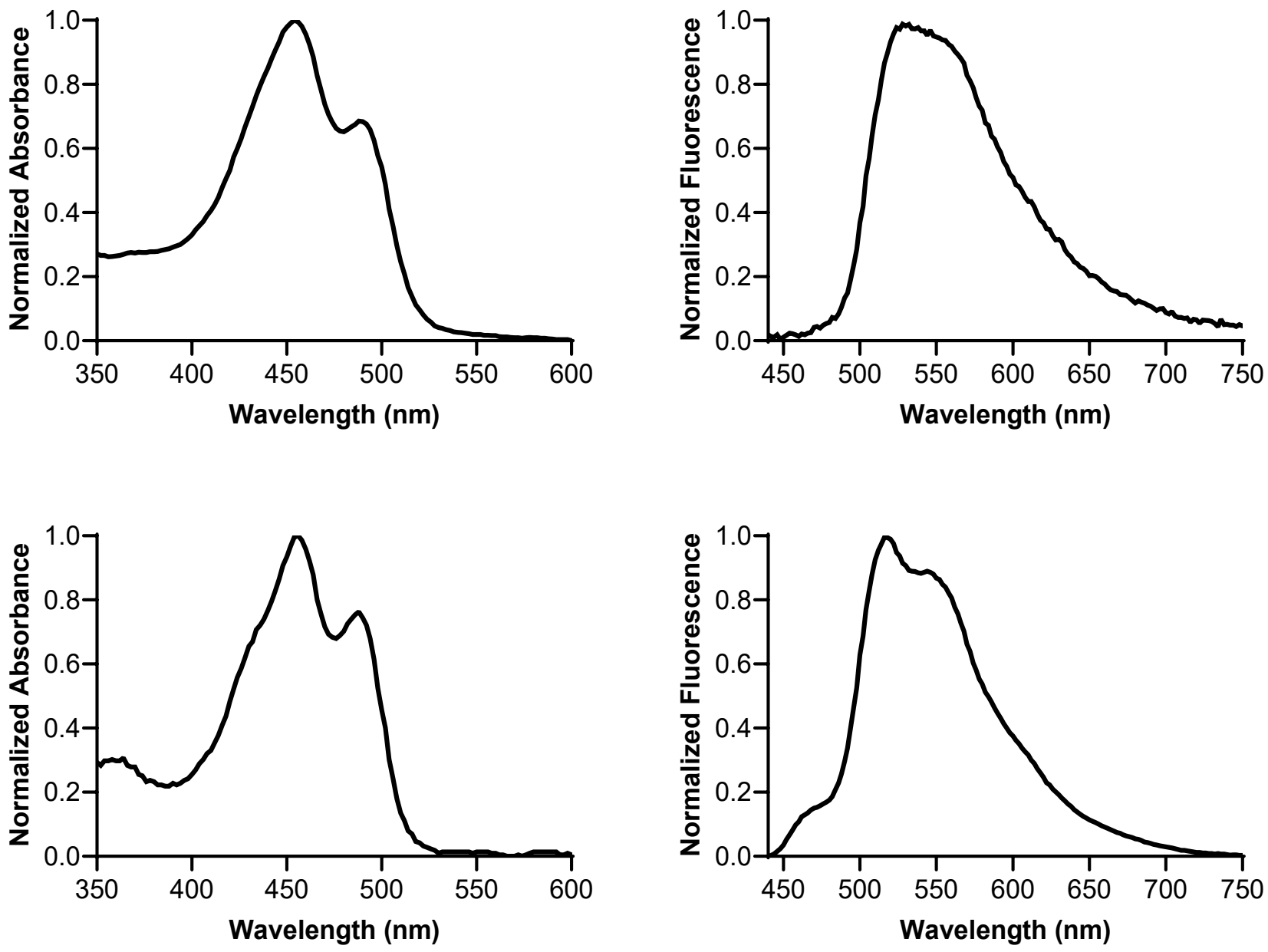

Supplementary Figure 4. Representative (from 3 independent experiments) normalized absorption and emission spectra of compounds **12** (50 μM, top) and **18** (10 μM, bottom) in EtOH. λ_{exc}: 410 nm.

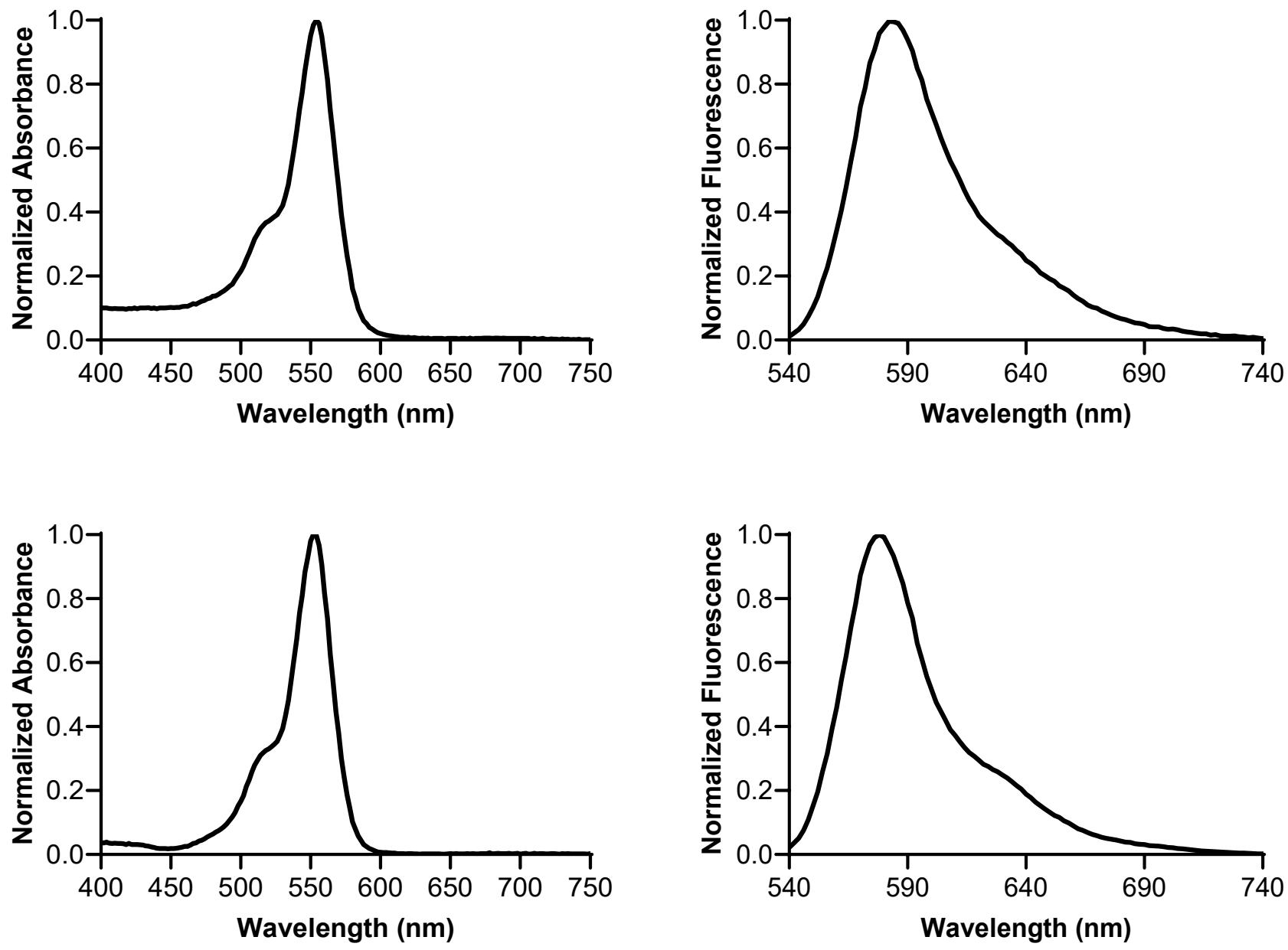

Supplementary Figure 5. Representative (from 3 independent experiments) normalized absorption and emission spectra of compounds **13** (10 μM, top) and **19** (10 μM, bottom) in EtOH. λ_{exc}: 510 nm.

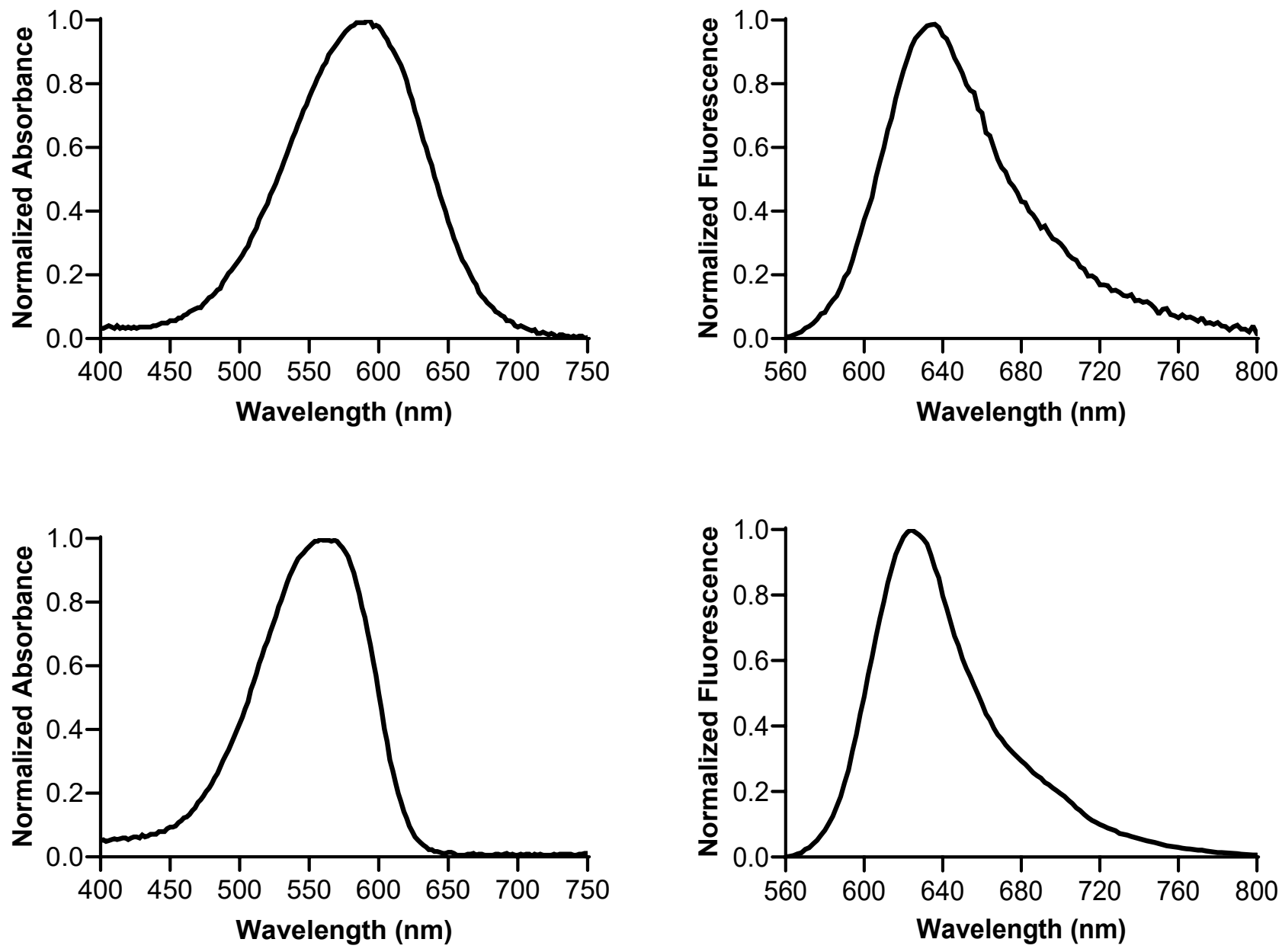

Supplementary Figure 6. Representative (from 3 independent experiments) normalized absorption and emission spectra of compounds **14** (10 µM, top) and **20** (10 µM, bottom) in EtOH. λ_{exc}: 530 nm.

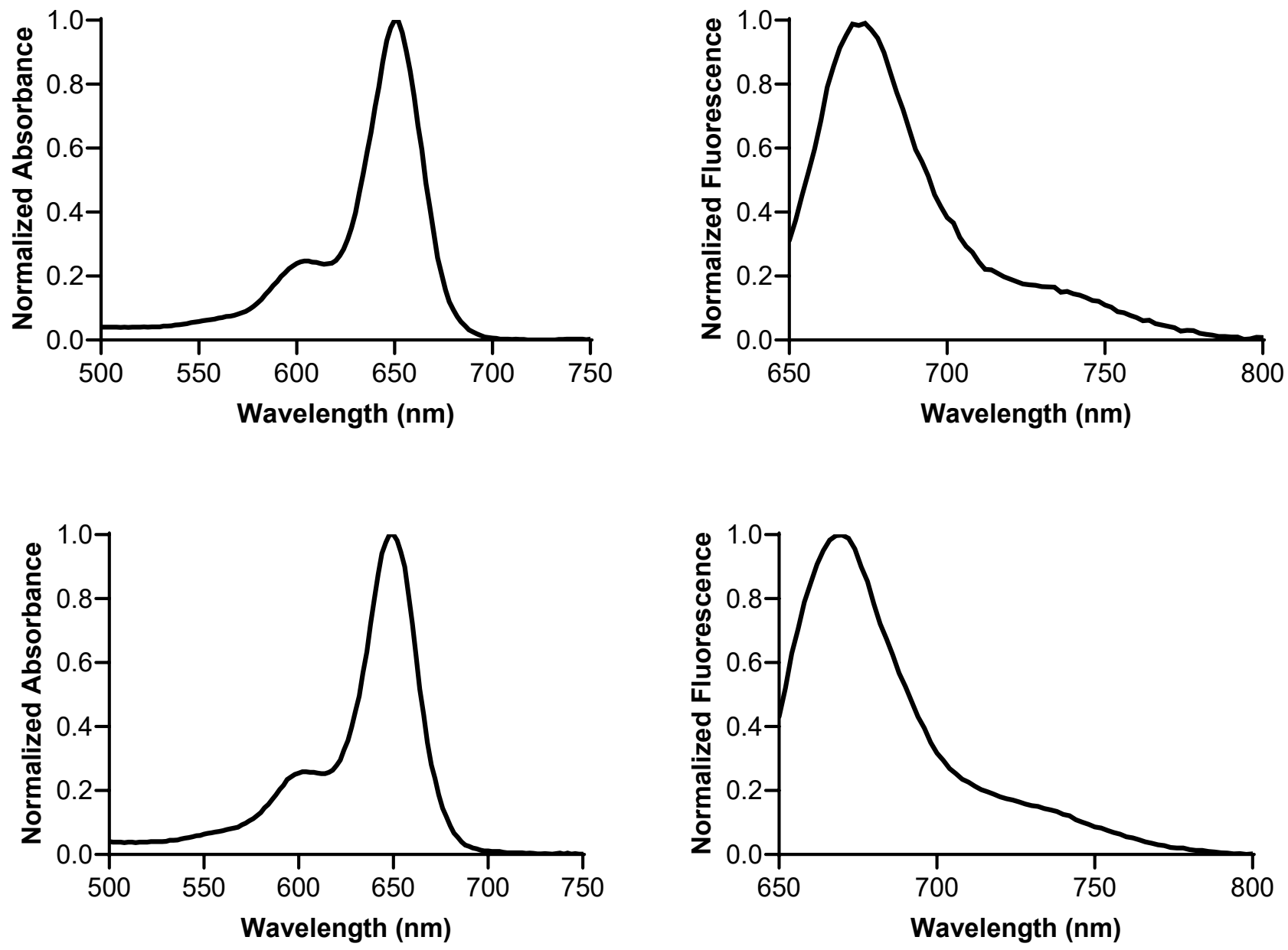

Supplementary Figure 7. Representative (from 3 independent experiments) normalized absorption and emission spectra of compounds **15** (10 μM, top) and **21** (10 μM, bottom) in EtOH. λ_{exc}: 620 nm.

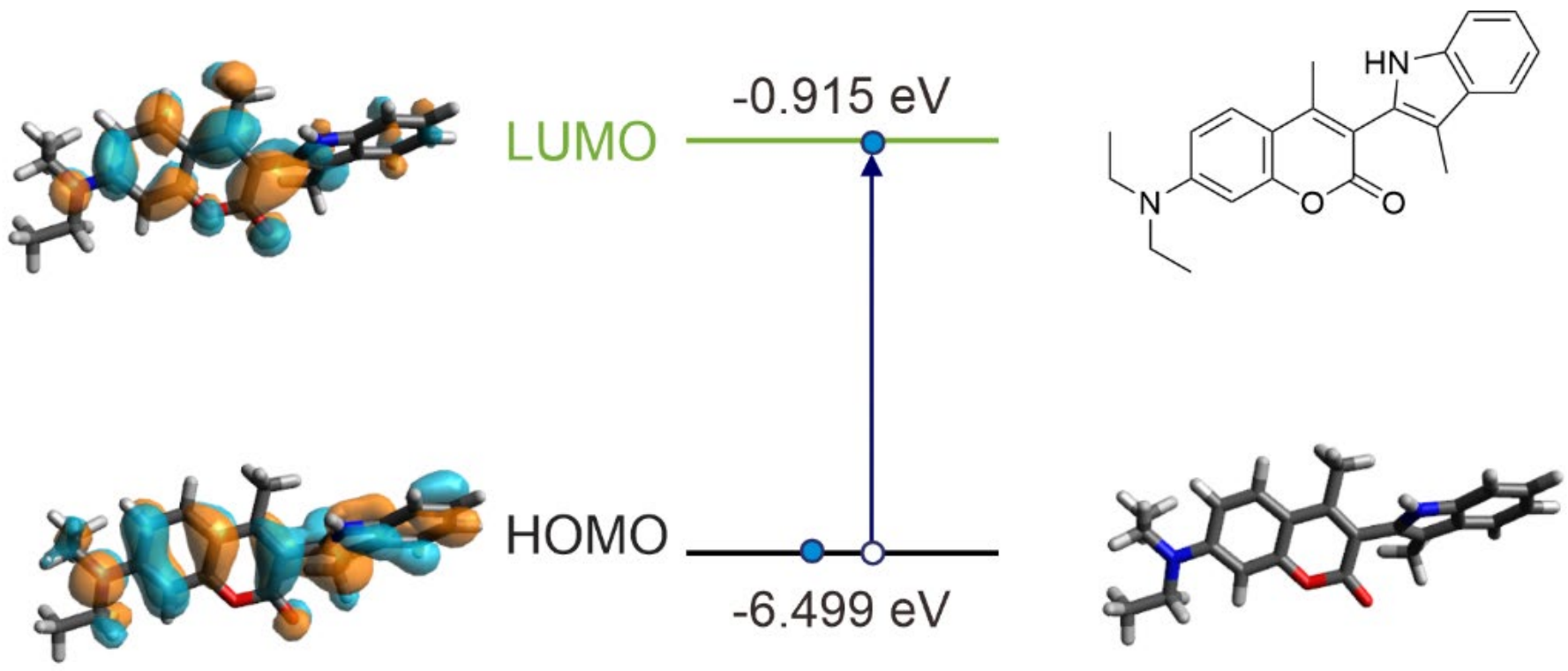

Supplementary Figure 8. Electron density and energy levels of HOMO and LUMO for compound coumarin-AA (**10**) in water, calculated at the optimized ground-state geometry using the M06-2X/def2-SVP level with SMD(water) solvation.

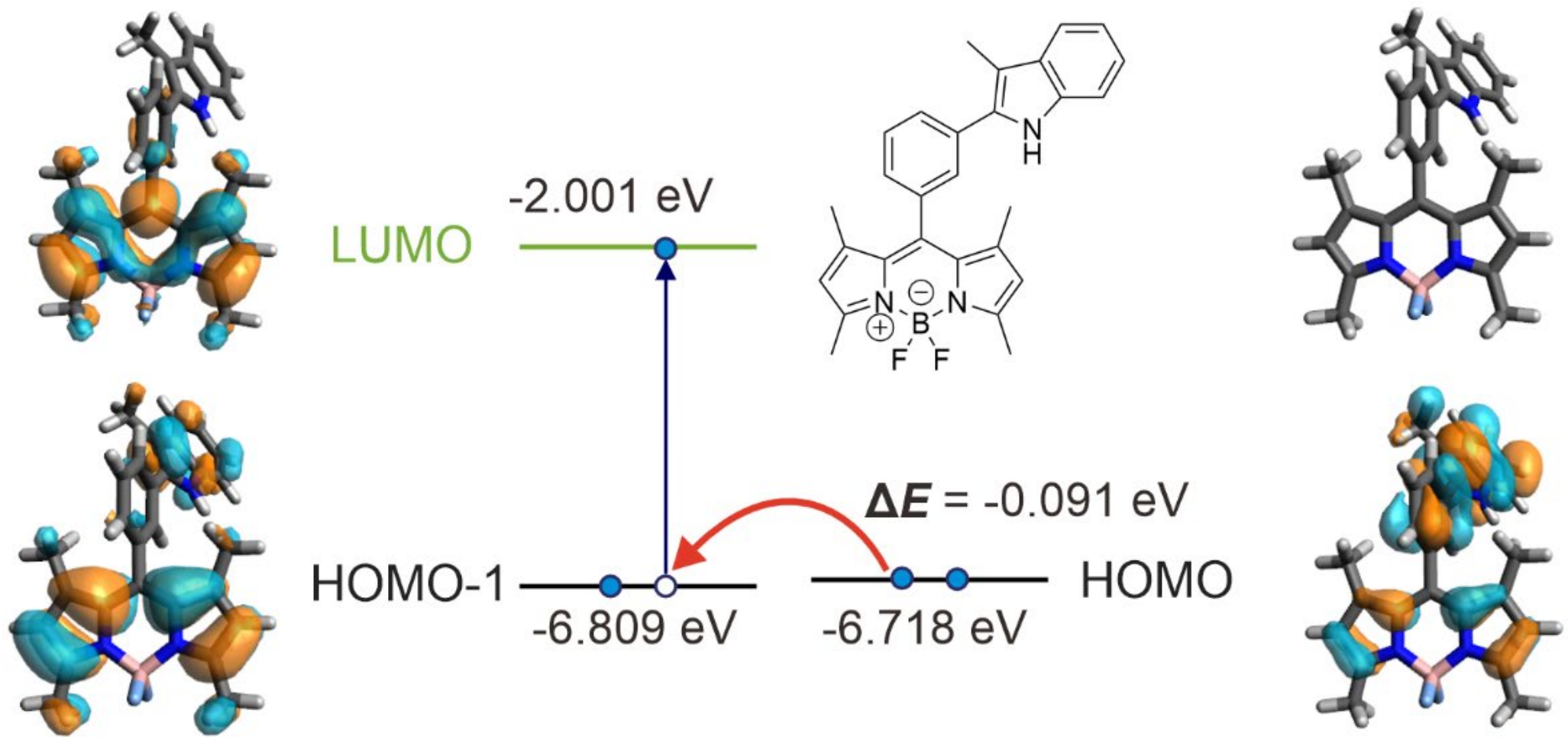

Supplementary Figure 9. Electron density and energy levels of HOMO-1, HOMO, and LUMO for compound BODIPY-AA (**11**) in water, calculated at the optimized ground-state geometry using the M06-2X/def2-SVP level with SMD(water) solvation. The inset highlights the energy gap (ΔE) between HOMO and HOMO-1.

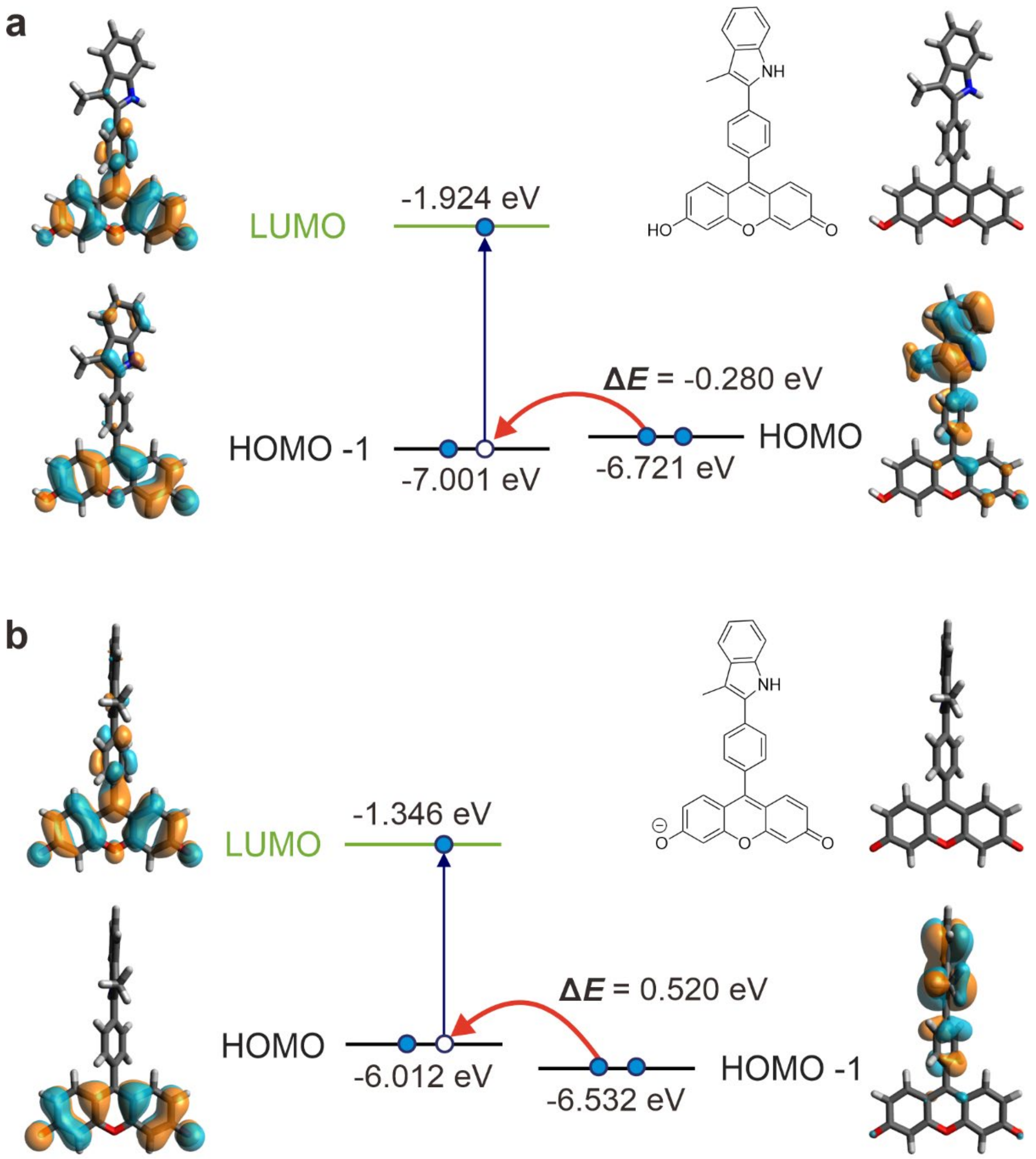

Supplementary Figure 10. Electron density and energy levels of HOMO-1, HOMO, and LUMO for the protonated and deprotonated forms of compound HPF-AA (**12**) in water, computed using the optimized ground-state geometry at the M06-2X/def2-SVP level for panel (a) or M06-2X/6-31G(d,p) for panel (b) with SMD(water) solvation. The inset highlights the energy gap (ΔE) between HOMO and HOMO-1. The top panel (a) shows compound HPF-AA (**12**) in a protonated form, and the bottom panel (b) shows compound HPF-AA (**12**) in a deprotonated form.

Supplementary Figure 11. Electron density and energy levels of HOMO-1, HOMO, and LUMO for compound O-rhodamine-AA (**13**) in water, calculated at the optimized ground-state geometry using the M06-2X/def2-SVP level with SMD(water) solvation. The inset highlights the energy gap (ΔE) between HOMO and HOMO-1.

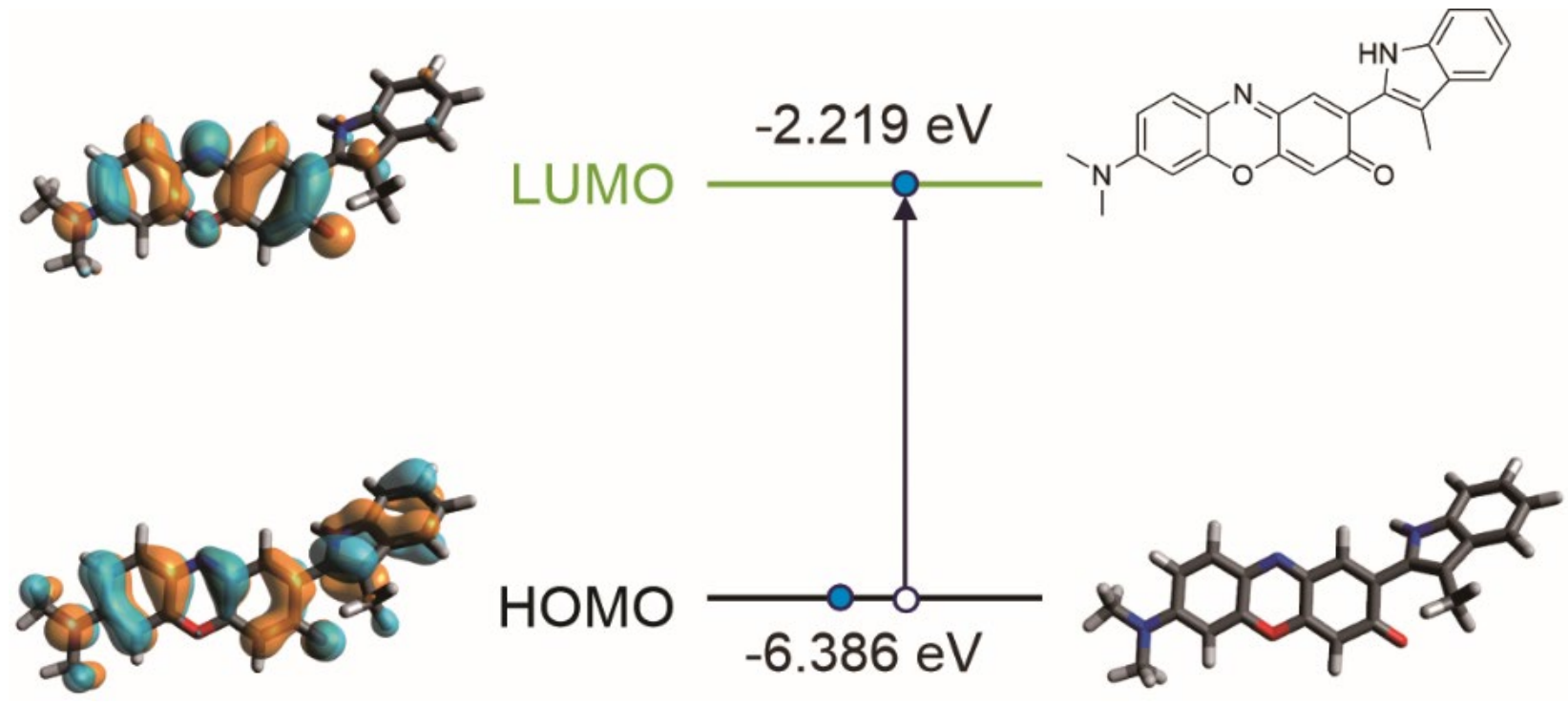

Supplementary Figure 12. Electron density and energy levels of HOMO and LUMO for compound oxazone-AA (**14**) in water, calculated at the optimized ground-state geometry using the M06-2X/def2-SVP level with SMD(water) solvation.

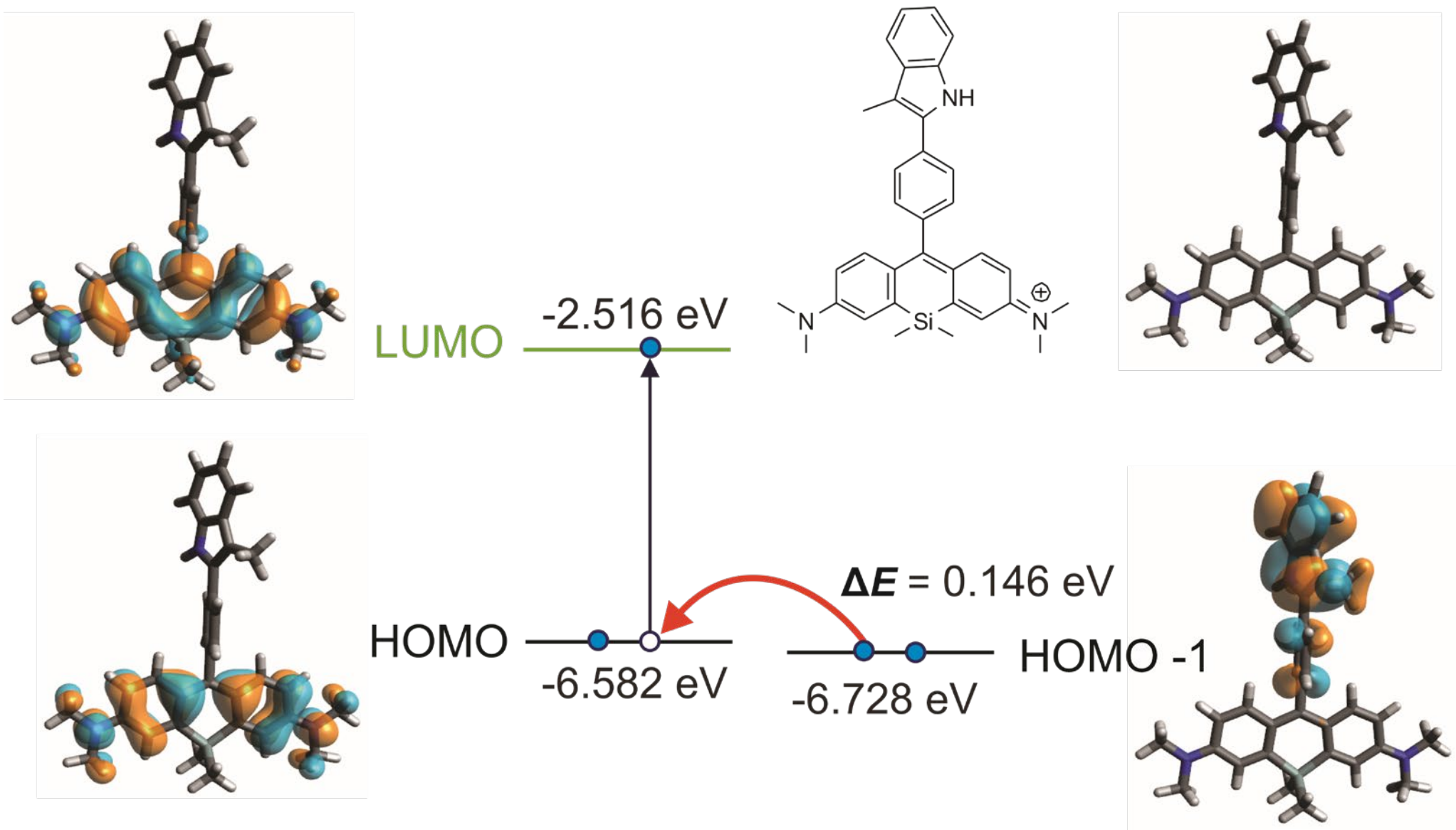

Supplementary Figure 13. Electron density and energy levels of HOMO-1, HOMO, and LUMO for compound Si-rhodamine-AA (**15**) in water, calculated at the optimized ground-state geometry using the M06-2X/def2-SVP level with SMD(water) solvation. The inset highlights the energy gap (ΔE) between HOMO and HOMO-1.

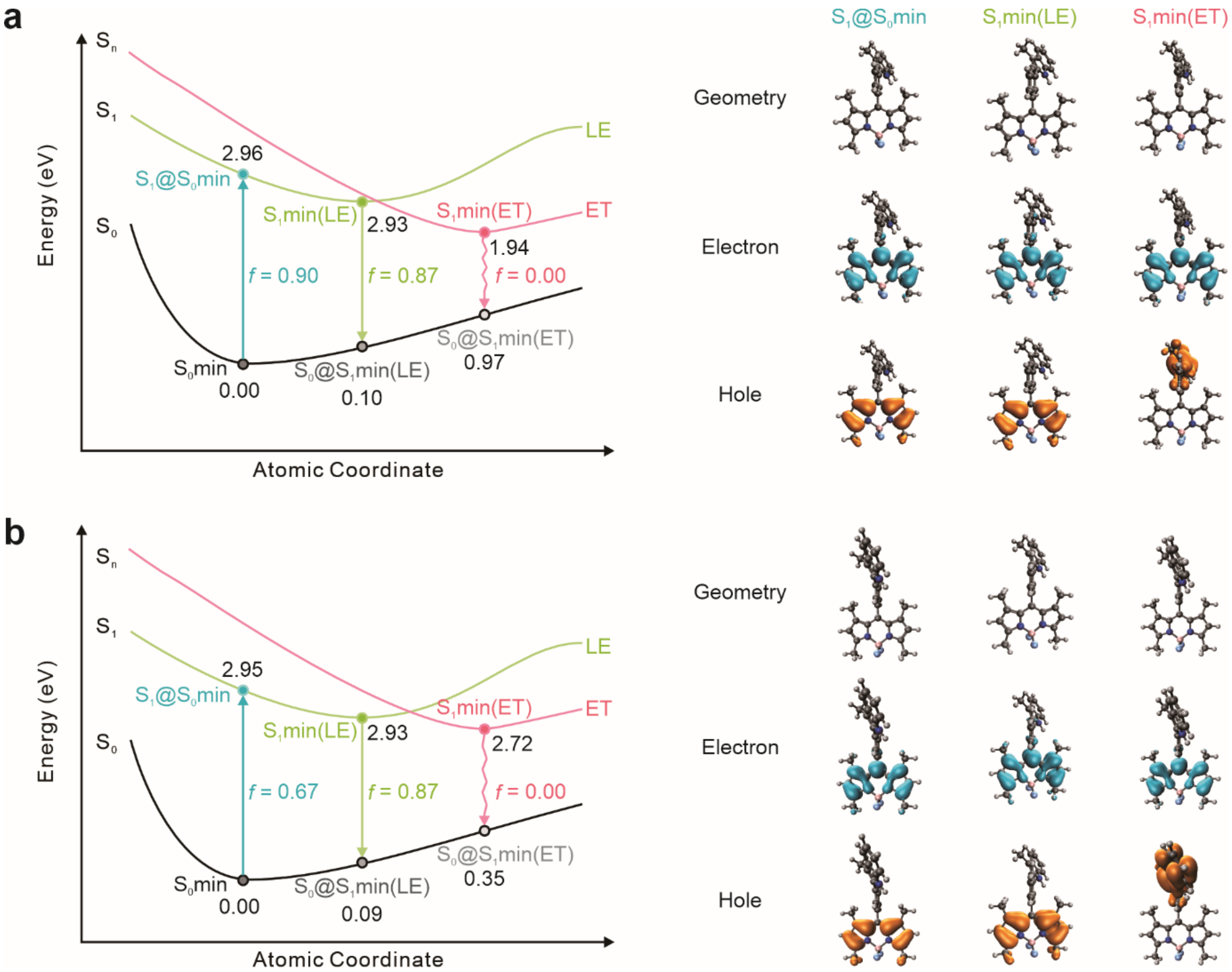

Supplementary Figure 14. Calculated excitation/de-excitation energy (including vertical oscillator strength *f*) of compound BODIPY-AA (**11**) in (a) water and (b) toluene. The right panel shows optimized molecular structures in representative states, along with the corresponding electron and hole distributions. LE: Locally Excited state; ET: Electron Transfer state. The driving energy from the LE (bright) state to the ET (dark) state decreases from water to toluene, indicating that PeT is suppressed in nonpolar solvents. The S_1 geometries were optimized at the M06-2X/def2-SVP level using the SMD solvation model within the linear-response (LR) formalism. Final single-point excited-state energies were calculated at the M06-2X/def2-TZVP level using the state-specific cLR formalism in conjunction with the SMD model in water (polar) or toluene (non-polar).

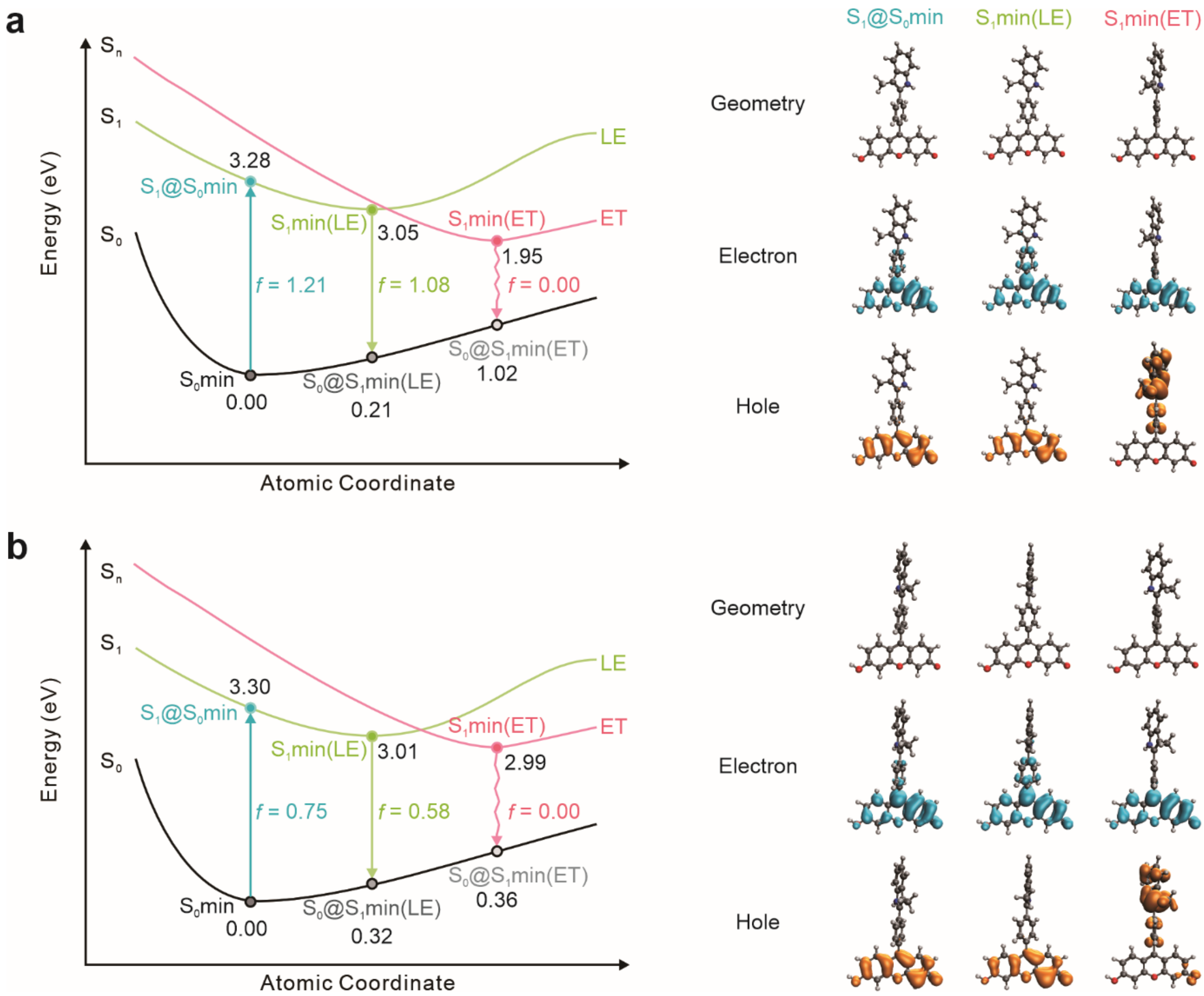

Supplementary Figure 15. Calculated excitation/de-excitation energy (including vertical oscillator strength f) of HPF-AA (**12**, protonated form) in (a) water and (b) toluene. The right panel shows optimized molecular structures in representative states, along with the corresponding electron and hole distributions. LE: Locally Excited state; ET: Electron Transfer state. The driving energy from the LE (bright) state to the ET (dark) state decreases from water to toluene, indicating that PeT is suppressed in nonpolar solvents. For clarity, the energy levels are not plotted to scale. The S_1 geometries were optimized at the M06-2X/def2-SVP level using the SMD solvation model within the linear-response (LR) formalism. Final single-point excited-state energies were calculated at the M06-2X/def2-TZVP level using the state-specific cLR formalism in conjunction with the SMD model in water (polar) or toluene (non-polar).

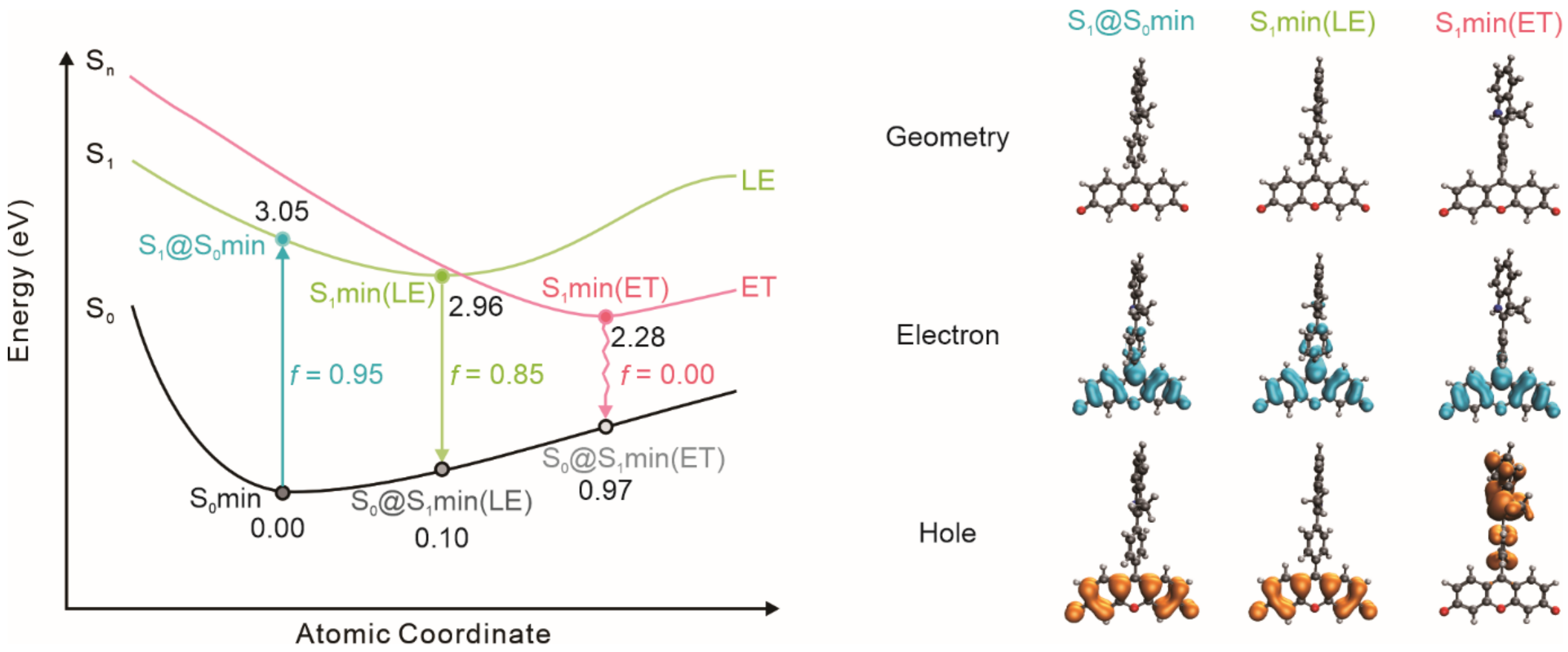

Supplementary Figure 16. Calculated excitation/de-excitation energy (including vertical oscillator strength f) of HPF-AA (**12**, deprotonated form) in water. The right panel shows optimized molecular structures in representative states, along with the corresponding electron and hole distributions. LE: Locally Excited state; ET: Electron Transfer state. For clarity, the energy levels are not plotted to scale. The S_1 geometries were optimized at the M06-2X/6-31G(d,p) level using the SMD solvation model within the linear-response (LR) formalism. Final single-point excited-state energies were calculated at the M06-2X/6-311+G(d,p) level using the state-specific cLR formalism in conjunction with the SMD model in water.

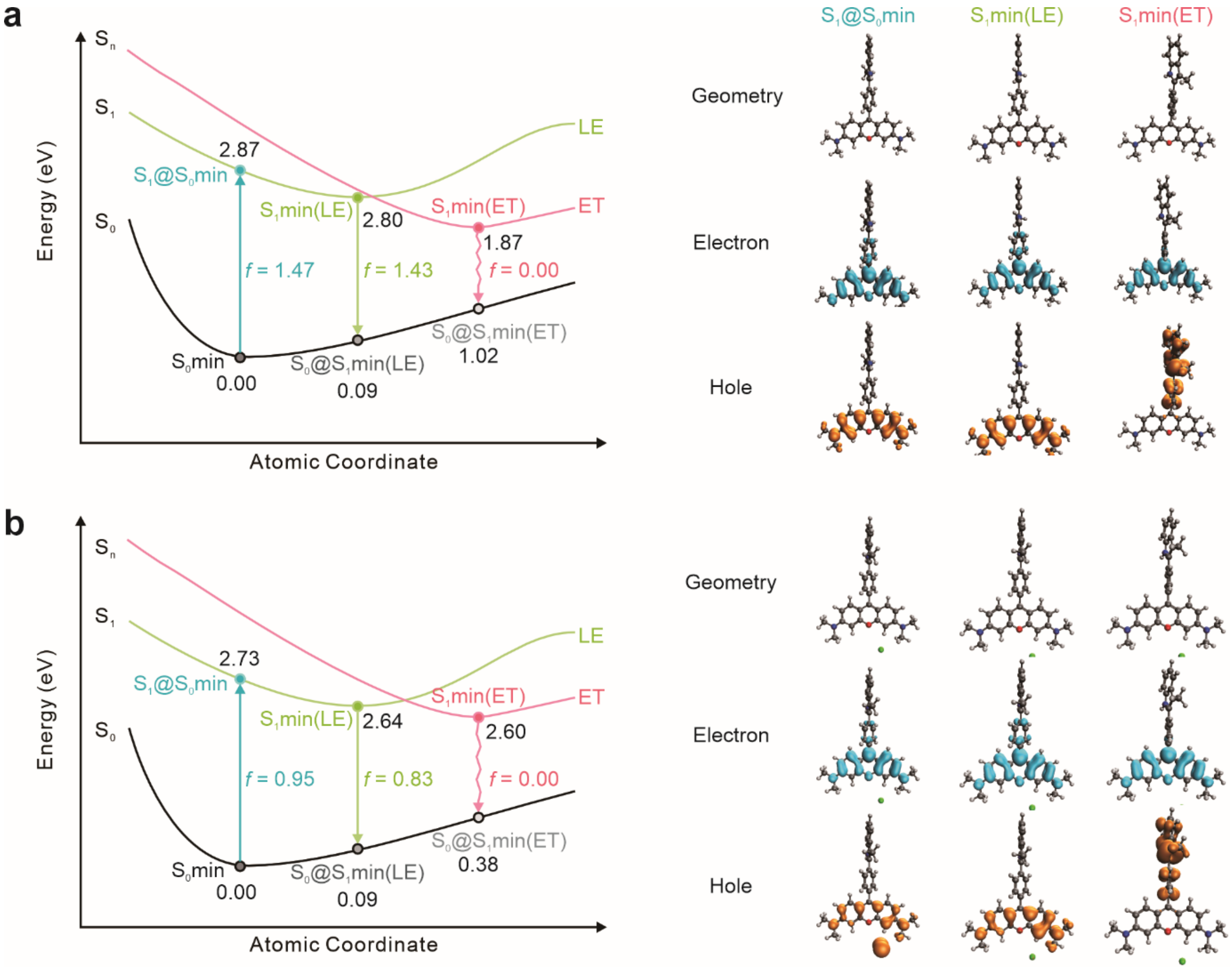

Supplementary Figure 17. Calculated excitation/de-excitation energy (including vertical oscillator strength *f*) of compound O-rhodamine-AA (**13**) in (a) water and (b) toluene. The right panel shows optimized molecular structures in representative states, along with the corresponding electron and hole distributions. LE: Locally Excited state; ET: Electron Transfer state. The driving energy from the LE (bright) state to the ET (dark) state decreases from water to toluene, indicating that PeT is suppressed in nonpolar solvents. For clarity, the energy levels are not plotted to scale. The S_1 geometries were optimized at the M06-2X/def2-SVP level using the SMD solvation model within the linear-response (LR) formalism. Final single-point excited-state energies were calculated at the M06-2X/def2-TZVP level using the state-specific cLR formalism in conjunction with the SMD model in water (polar) or toluene (non-polar).

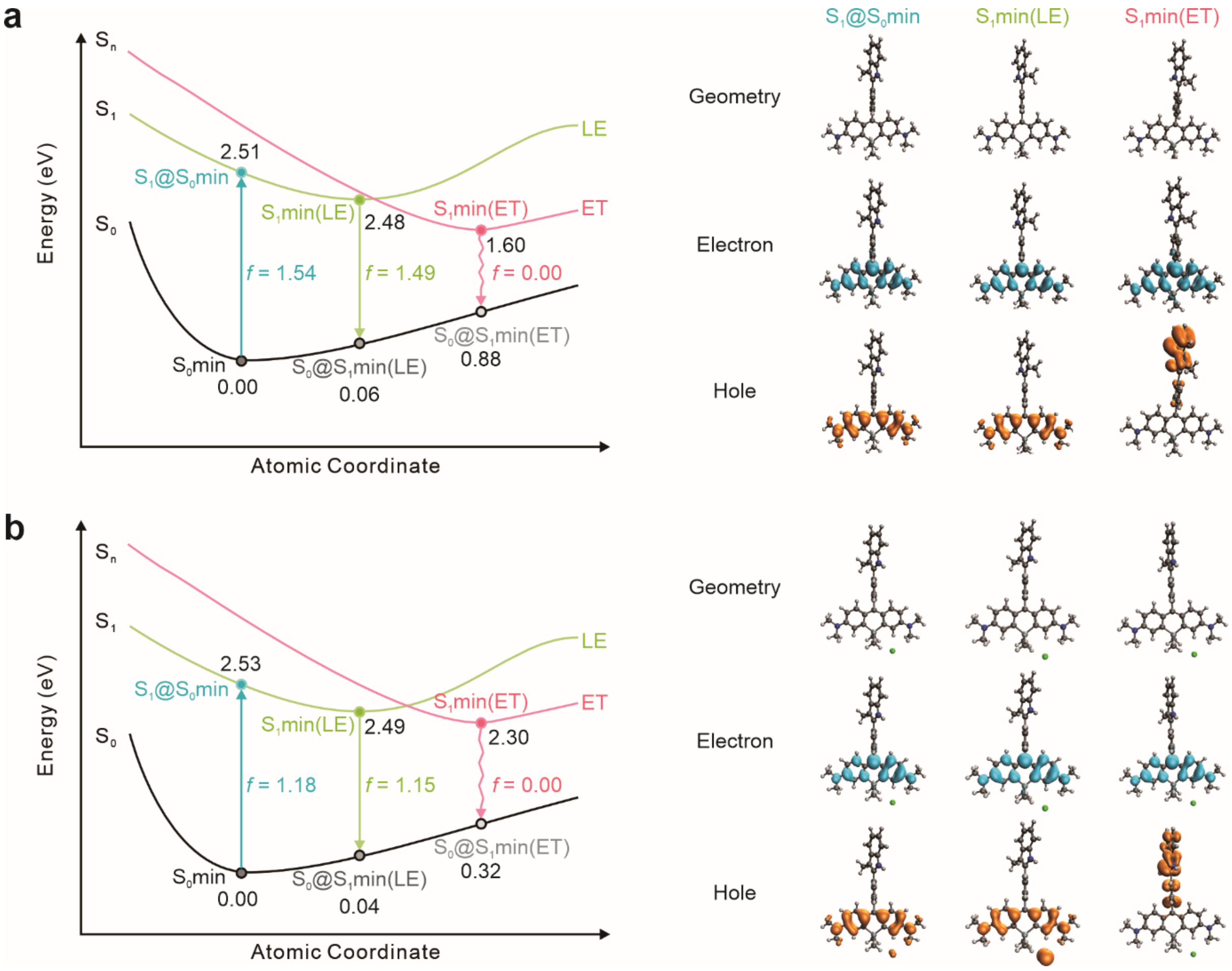

Supplementary Figure 18. Calculated excitation/de-excitation energy (including vertical oscillator strength *f*) of compound Si-rhodamine-AA (**15**) in (a) water and (b) toluene. The right panel shows optimized molecular structures in representative states, along with the corresponding electron and hole distributions. LE: Locally Excited state; ET: Electron Transfer state. The driving energy from the LE (bright) state to the ET (dark) state decreases from water to toluene, indicating that PeT is suppressed in nonpolar solvents. For clarity, the energy levels are not plotted to scale. The S_1 geometries were optimized at the M06-2X/def2-SVP level using the SMD solvation model within the linear-response (LR) formalism. Final single-point excited-state energies were calculated at the M06-2X/def2-TZVP level using the state-specific cLR formalism in conjunction with the SMD model in water (polar) or toluene (non-polar).

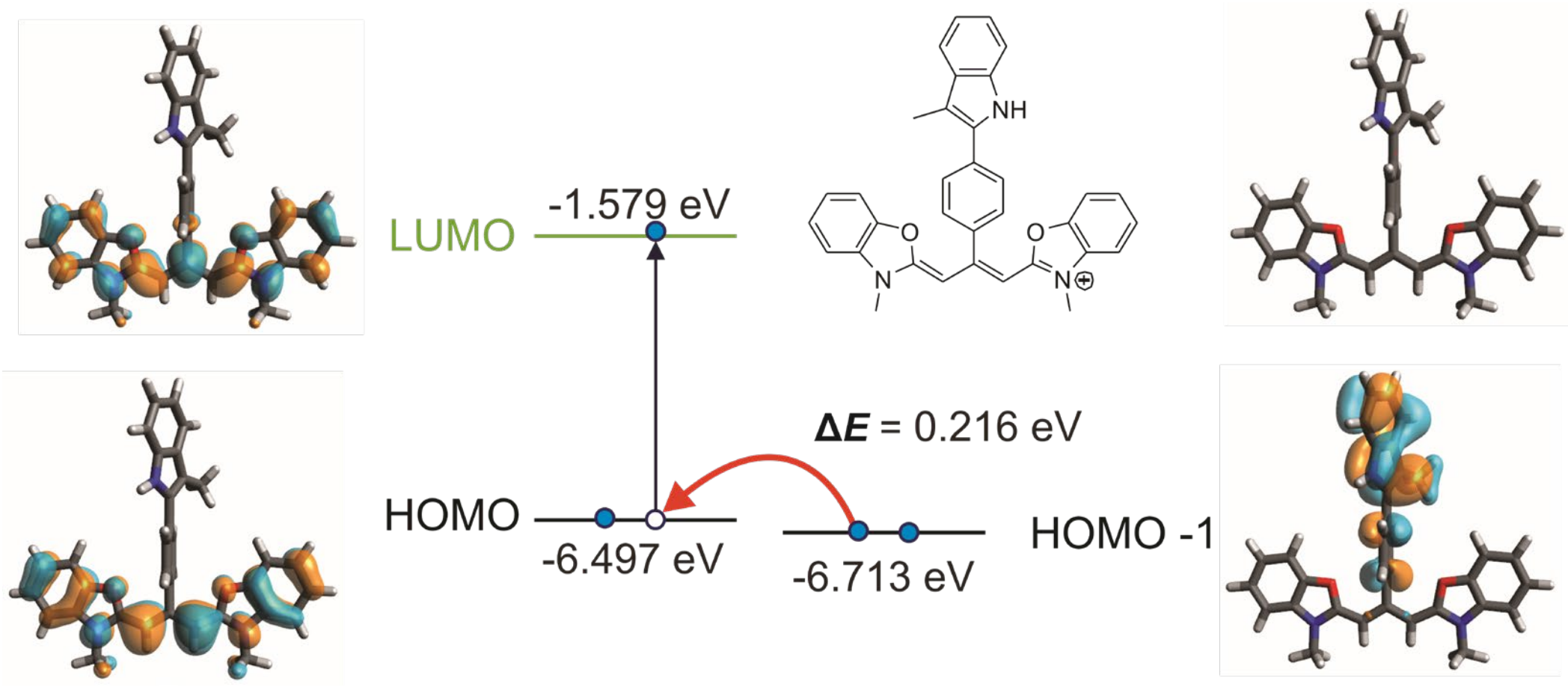

Supplementary Figure 19. Electron density and energy levels of HOMO-1, HOMO, and LUMO for phenyl-Cy3-AA in water, calculated at the optimized ground-state geometry using the M06-2X/def2-SVP level with SMD(water) solvation. The inset highlights the energy gap (Δ*E*) between HOMO and HOMO-1.

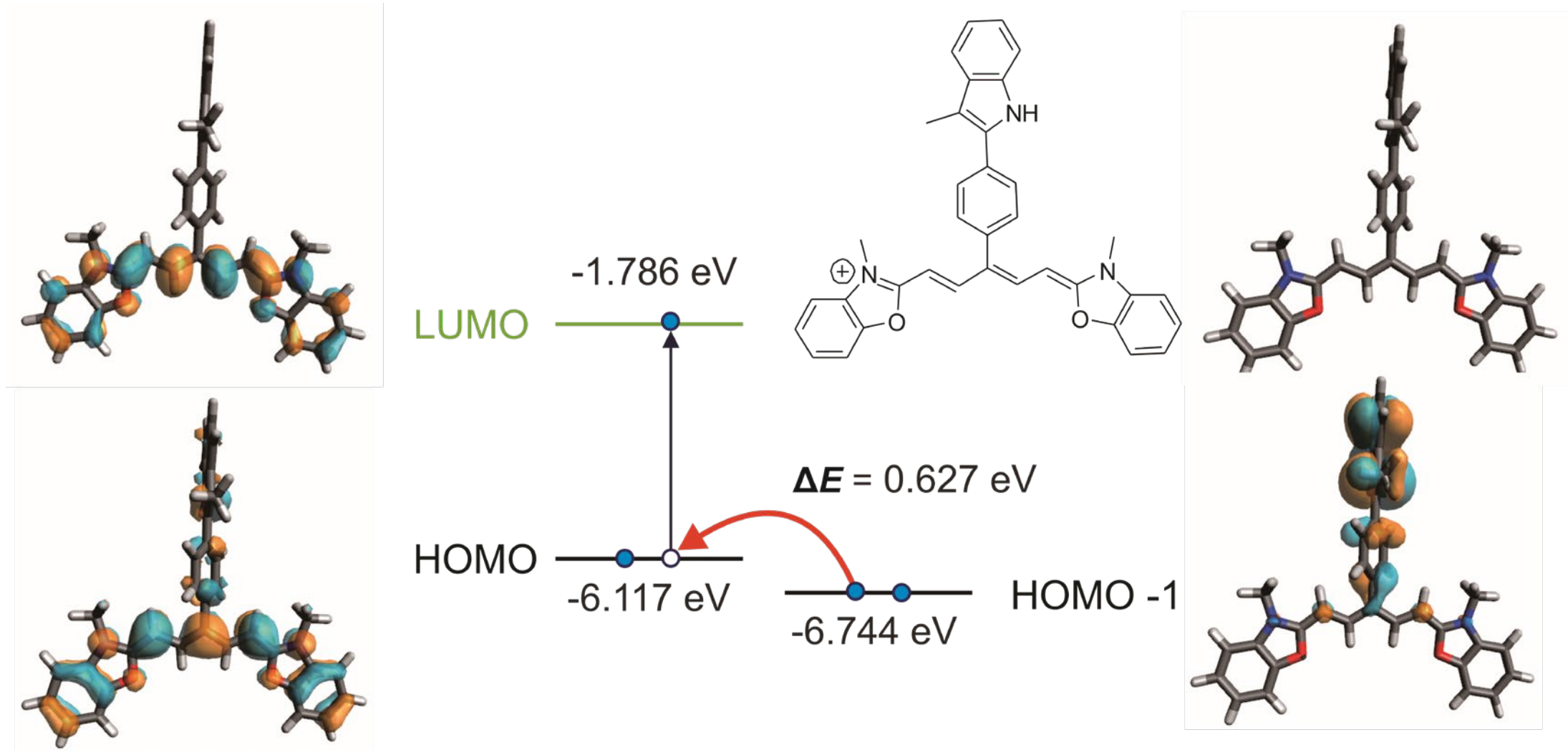

Supplementary Figure 20. Electron density and energy levels of HOMO-1, HOMO, and LUMO for phenyl-Cy5-AA in water, calculated at the optimized ground-state geometry using the M06-2X/def2-SVP level with SMD(water) solvation. The inset highlights the energy gap (Δ*E*) between HOMO and HOMO-1.

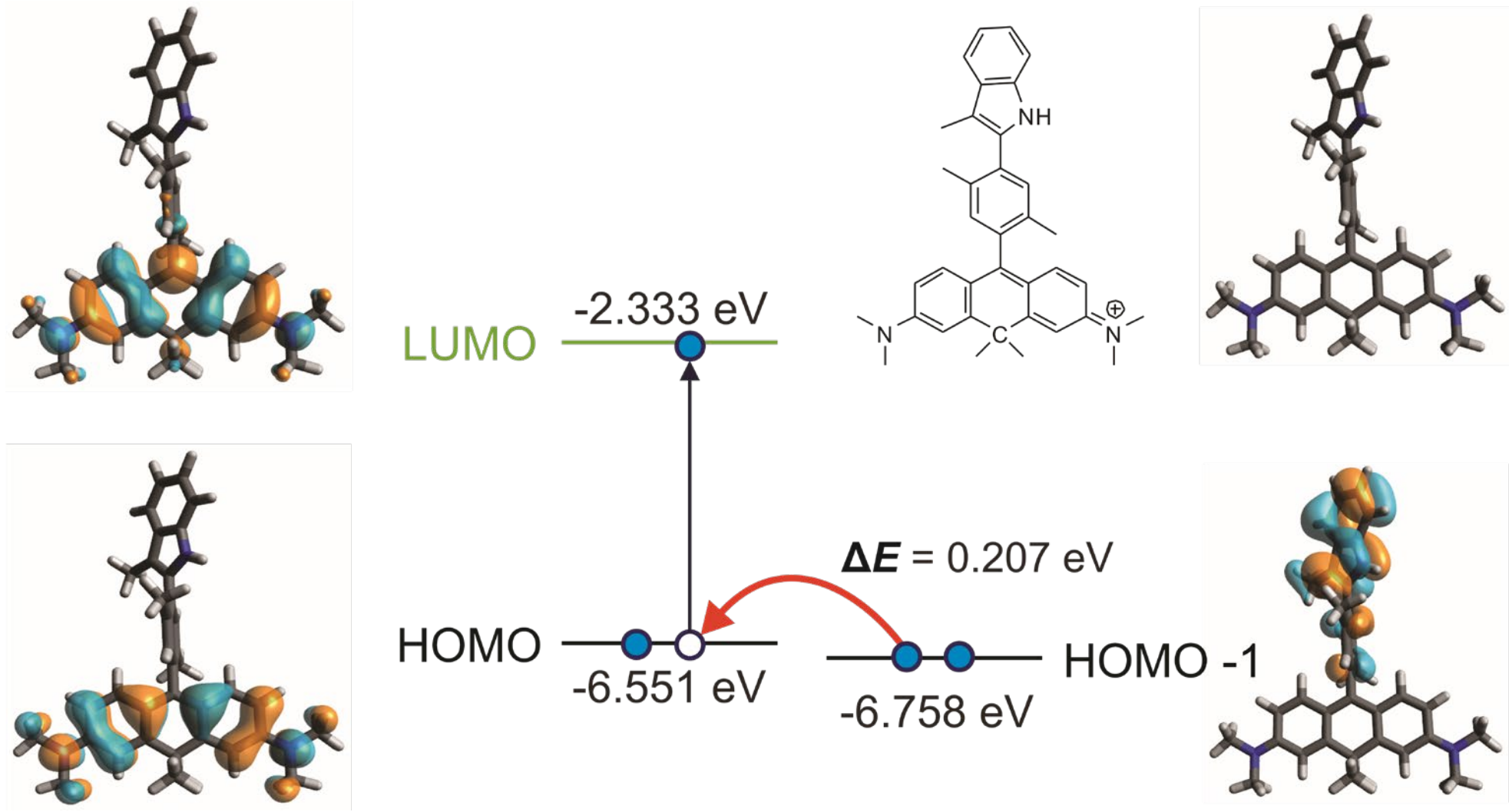

Supplementary Figure 21. Electron density and energy levels of HOMO-1, HOMO, and LUMO for carbopyronine-AA in water, calculated at the optimized ground-state geometry using the M06-2X/def2-SVP level with SMD(water) solvation. The inset highlights the energy gap (ΔE) between HOMO and HOMO-1.

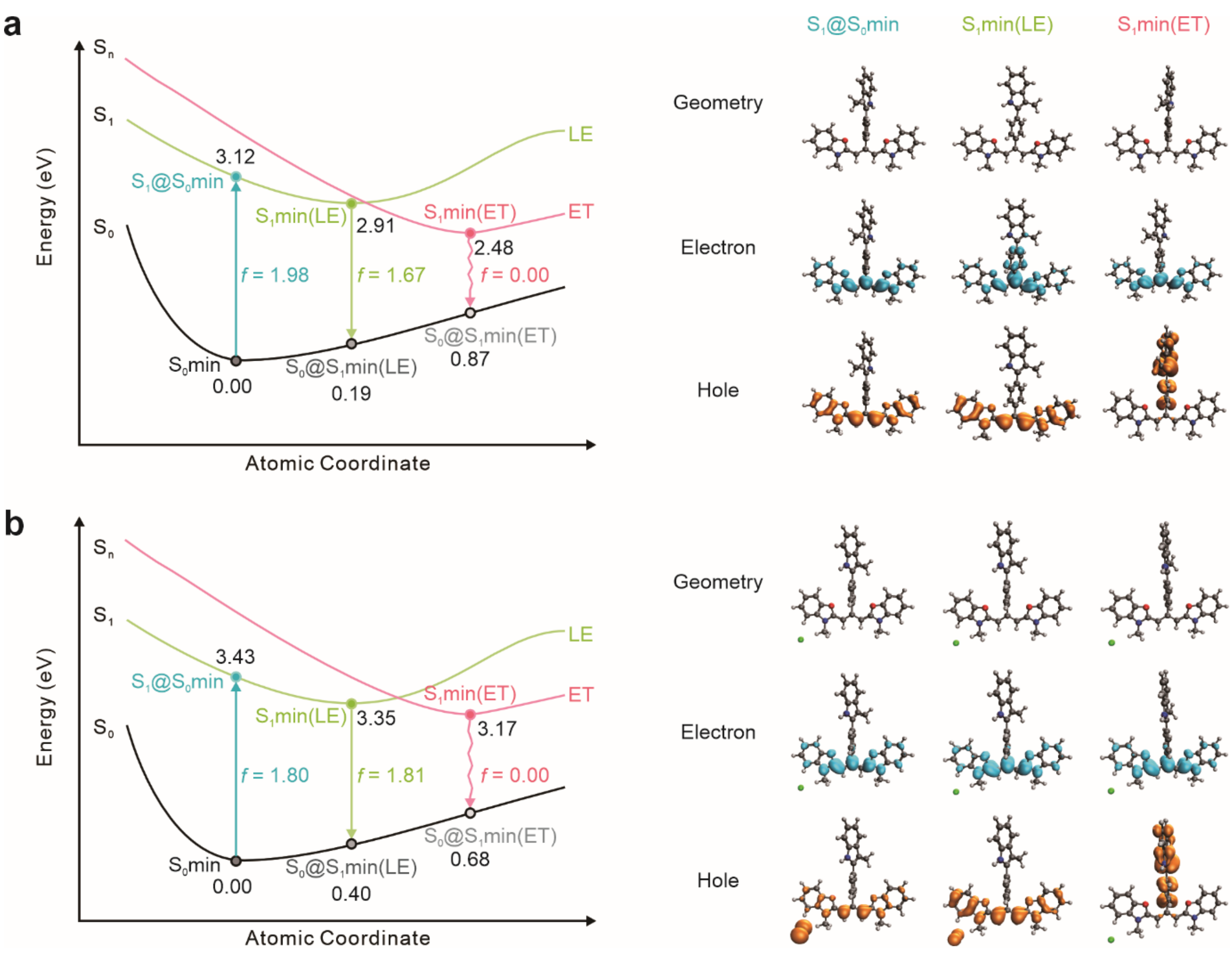

Supplementary Figure 22. Calculated excitation/de-excitation energy (including vertical oscillator strength *f*) of phenyl-Cy3-AA in (a) water and (b) toluene. The right panel shows optimized molecular structures in representative states, along with the corresponding electron and hole distributions. LE: Locally Excited state; ET: Electron Transfer state. The driving energy from the LE (bright) state to the ET (dark) state decreases from water to toluene, indicating that PeT is suppressed in nonpolar solvents. For clarity, the energy levels are not plotted to scale. The S_1 geometries were optimized at the M06-2X/def2-SVP level using the SMD solvation model within the linear-response (LR) formalism. Final single-point excited-state energies were calculated at the M06-2X/def2-TZVP level using the state-specific cLR formalism in conjunction with the SMD model in water (polar) or toluene (non-polar). During

geometry optimizations of both the LE and ET states, the meso-phenyl dihedral angles were constrained to 90°.

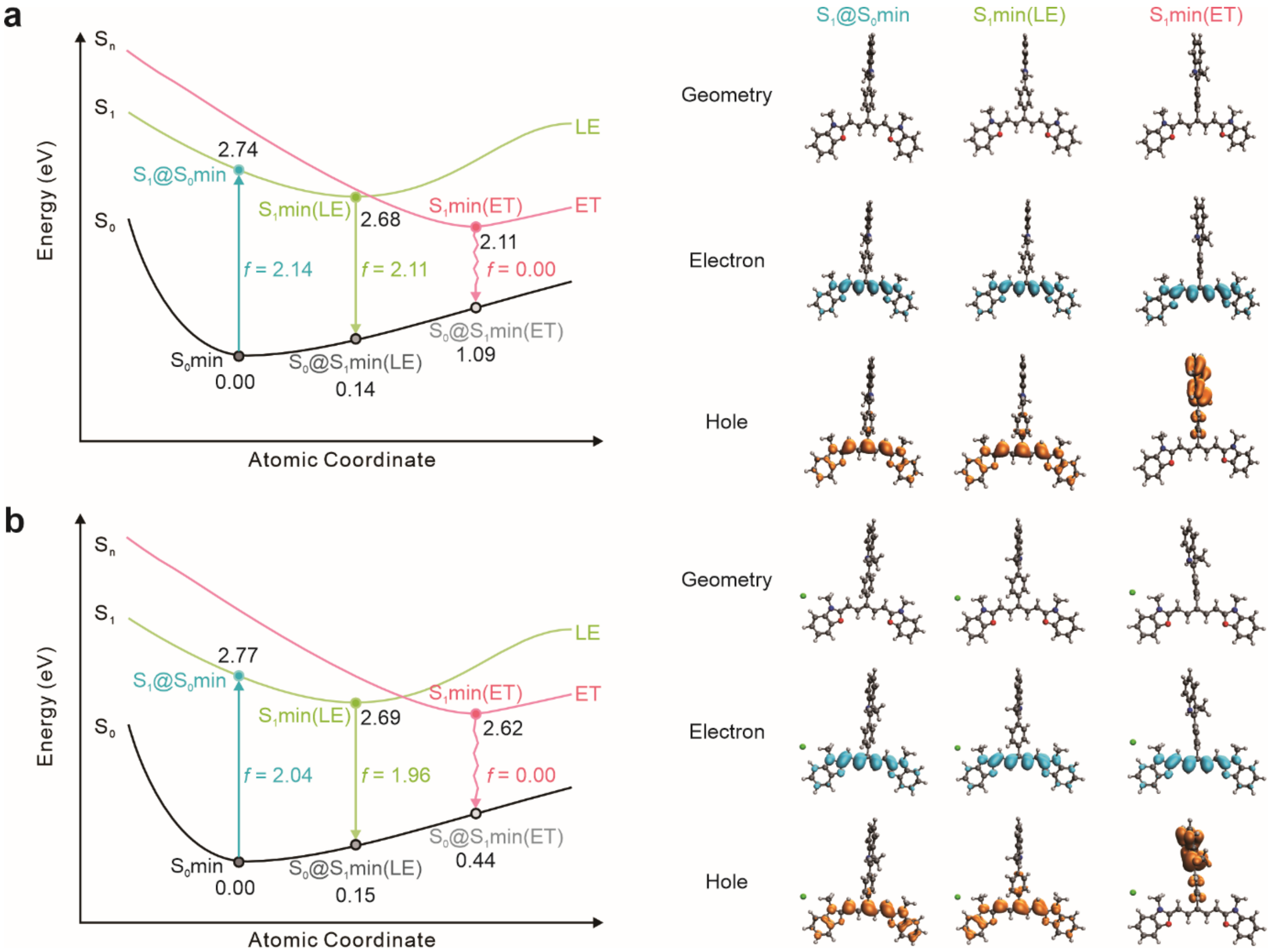

Supplementary Figure 23. Calculated excitation/de-excitation energy (including vertical oscillator strength *f*) of phenyl-Cy5-AA in (a) water and (b) toluene. The right panel shows optimized molecular structures in representative states, along with the corresponding electron and hole distributions. LE: Locally Excited state; ET: Electron Transfer state. The driving energy from the LE (bright) state to the ET (dark) state decreases from water to toluene, indicating that PeT is suppressed in nonpolar solvents. For clarity, the energy levels are not plotted to scale. The S_1 geometries were optimized at the M06-2X/def2-SVP level using the SMD solvation model within the linear-response (LR) formalism. Final single-point excited-state energies were calculated at the M06-2X/def2-TZVP level using the state-specific cLR formalism in conjunction with the SMD model in water (polar) or toluene (non-polar).

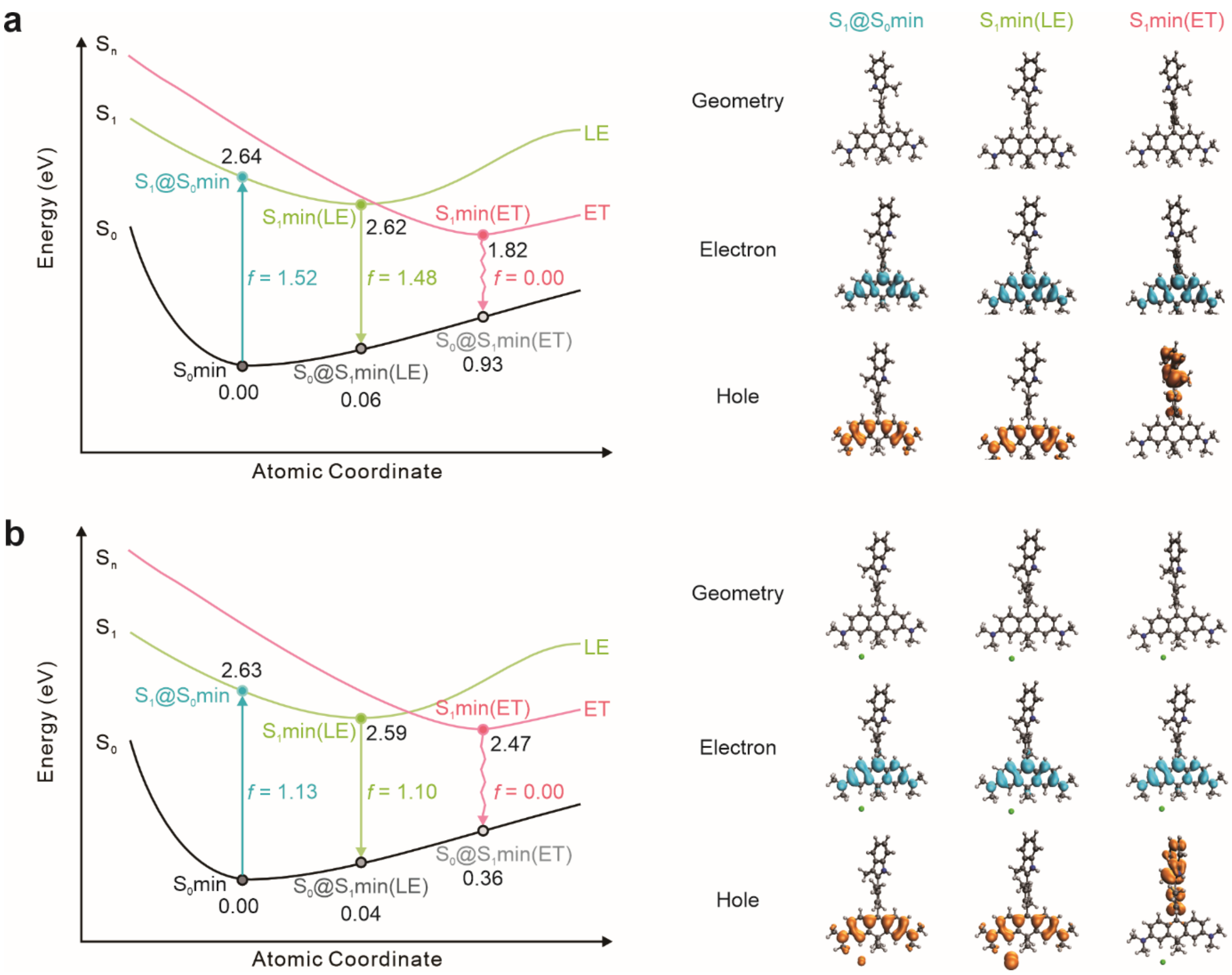

Supplementary Figure 24. Calculated excitation/de-excitation energy (including vertical oscillator strength *f*) of carbopyronine-AA in (a) water and (b) toluene. The right panel shows optimized molecular structures in representative states, along with the corresponding electron and hole distributions. LE: Locally Excited state; ET: Electron Transfer state. The driving energy from the LE (bright) state to the ET (dark) state decreases from water to toluene, indicating that PeT is suppressed in nonpolar solvents. For clarity, the energy levels are not plotted to scale. The S_1 geometries were optimized at the M06-2X/def2-SVP level using the SMD solvation model within the linear-response (LR) formalism. Final single-point excited-state energies were calculated at the M06-2X/def2-TZVP level using the state-specific cLR formalism in conjunction with the SMD model in water (polar) or toluene (non-polar).

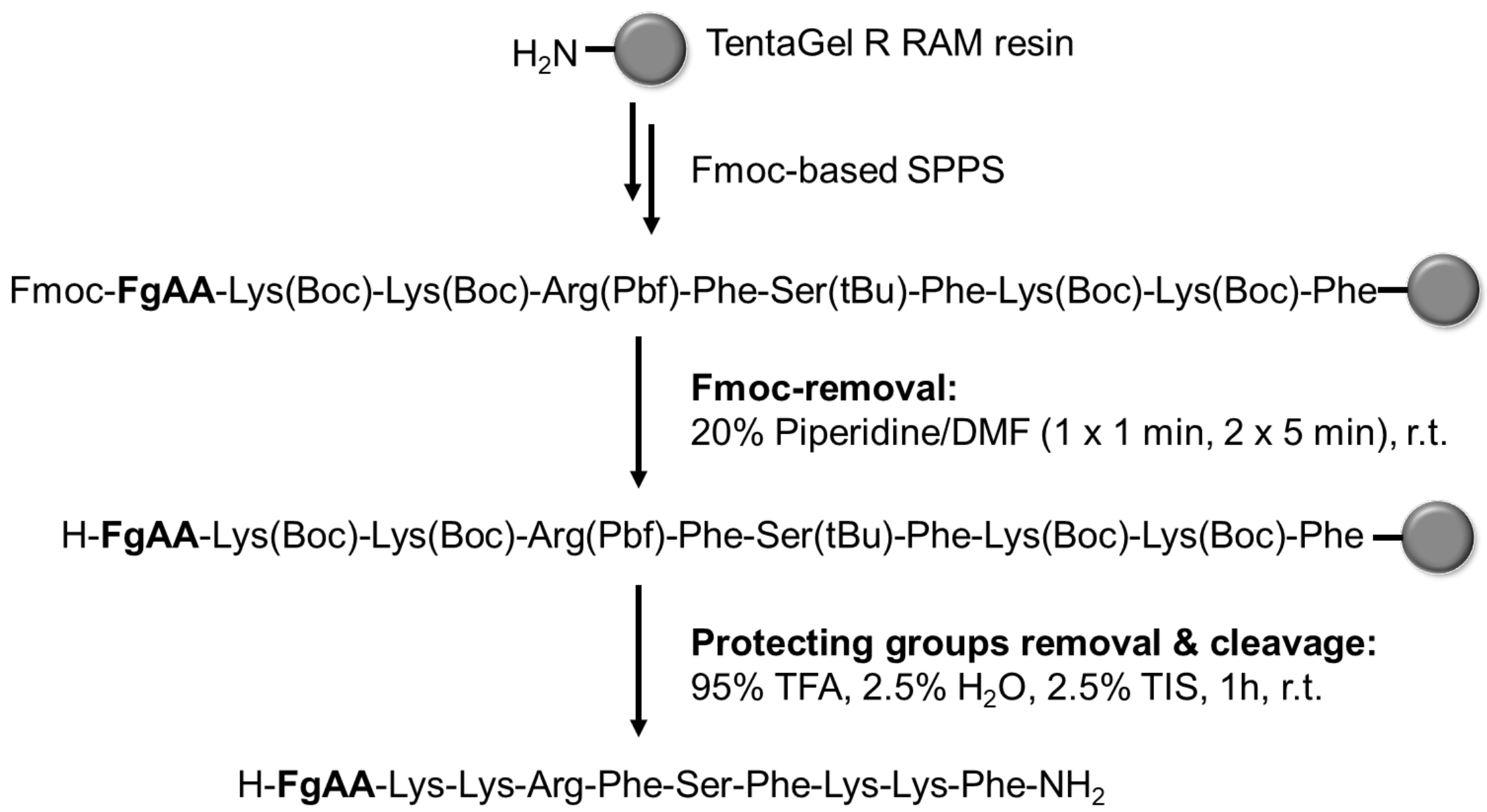

Supplementary Figure 25. Synthetic scheme for the preparation of fluorogenic peptides **22**-**24**. FgAA: HPF-AA (for peptide **22**), O-rhodamine-AA (for peptide **23**), Si-rhodamine-AA (for peptide **24**).

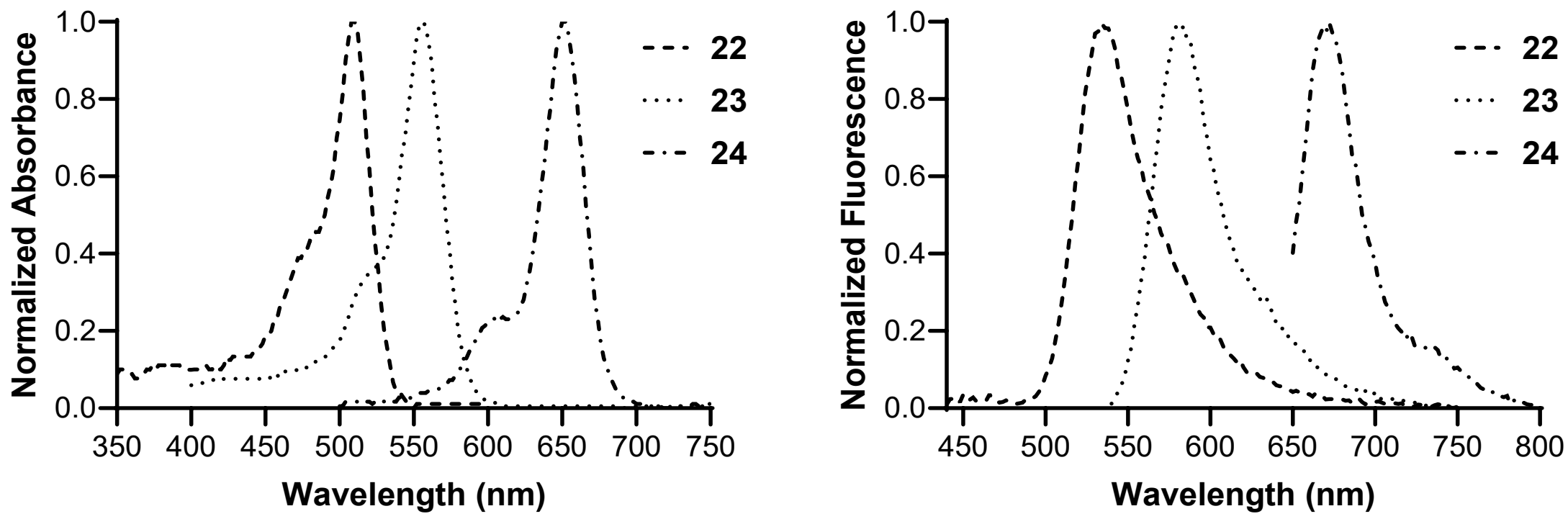

Supplementary Figure 26. Representative (from 3 independent experiments) normalized absorption (left) and emission (right) spectra of peptides **22** (25 μM), **23** (25 μM), and **24** (18 μM) in EtOH. λ_{exc}: 410 nm (for **22**), 510 nm (for **23**) and 620 nm (for **24**).

Peptide **22**

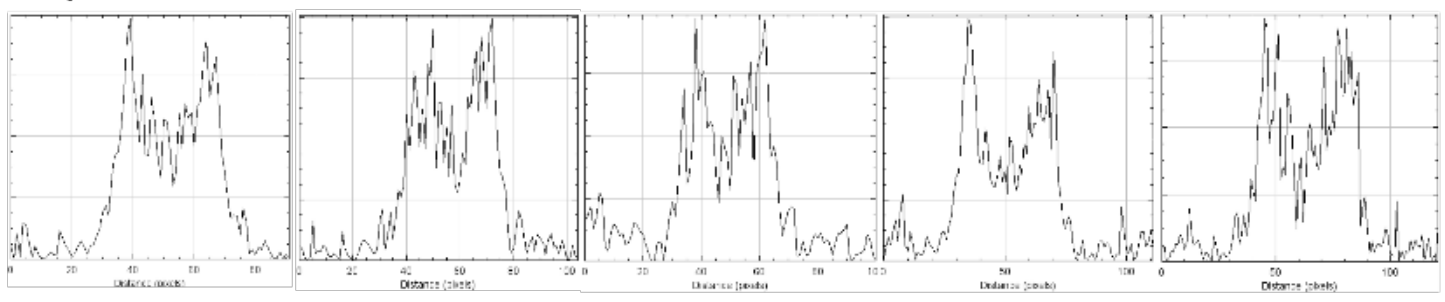

	Average background	Average signal	SNR
Replicate 1	3.5	78	22
Replicate 2	4.5	81	18
Replicate 3	5.1	76	15
Replicate 4	4.3	88	20
Replicate 5	4.4	70	16

Peptide **23**

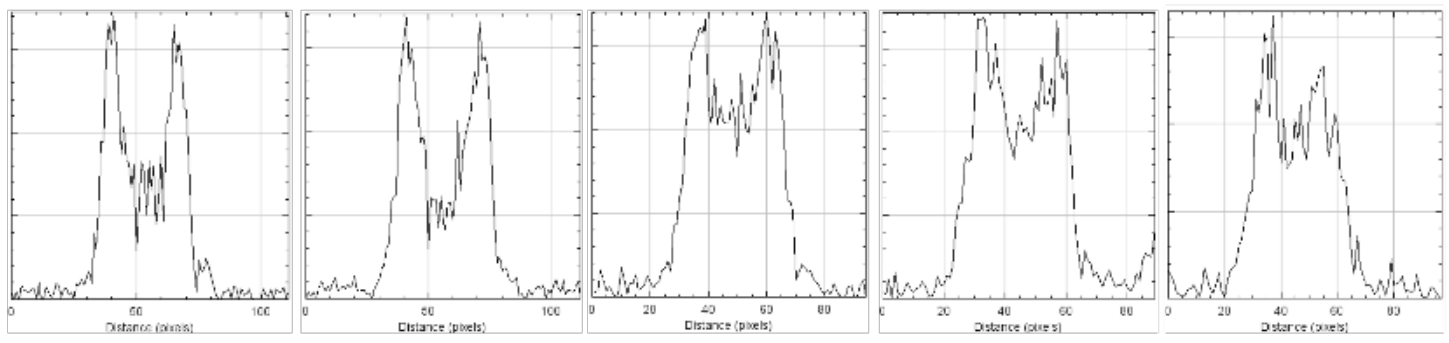

	Average background	Average signal	SNR
Replicate 1	4.8	152	32
Replicate 2	3.7	126	34
Replicate 3	3.9	160	41
Replicate 4	3.5	111	32
Replicate 5	5.9	142	24

Peptide **24**

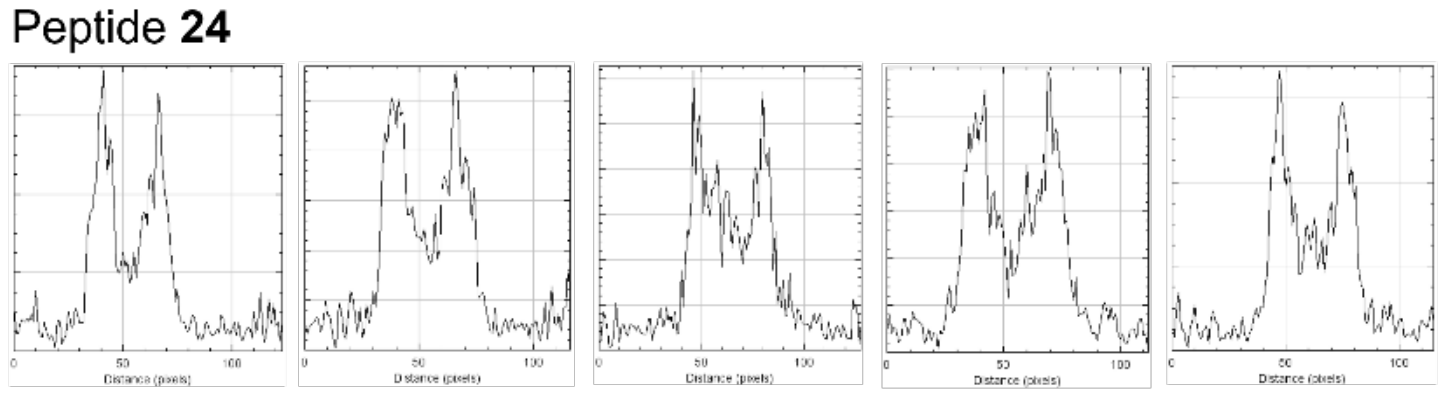

	Average background	Average signal	SNR
Replicate 1	1.1	71	64
Replicate 2	1.3	76	58
Replicate 3	1.3	77	59
Replicate 4	1.6	85	53
Replicate 5	1.4	80	57

Supplementary Figure 27. Plot profiles (from 5 independent replicates) of *E. coli* bacterial cells upon incubation with peptides **22**-**24** (representative fluorescence microscopy images shown in Figure 3) and calculation of the signal-to-noise (SNR) values.

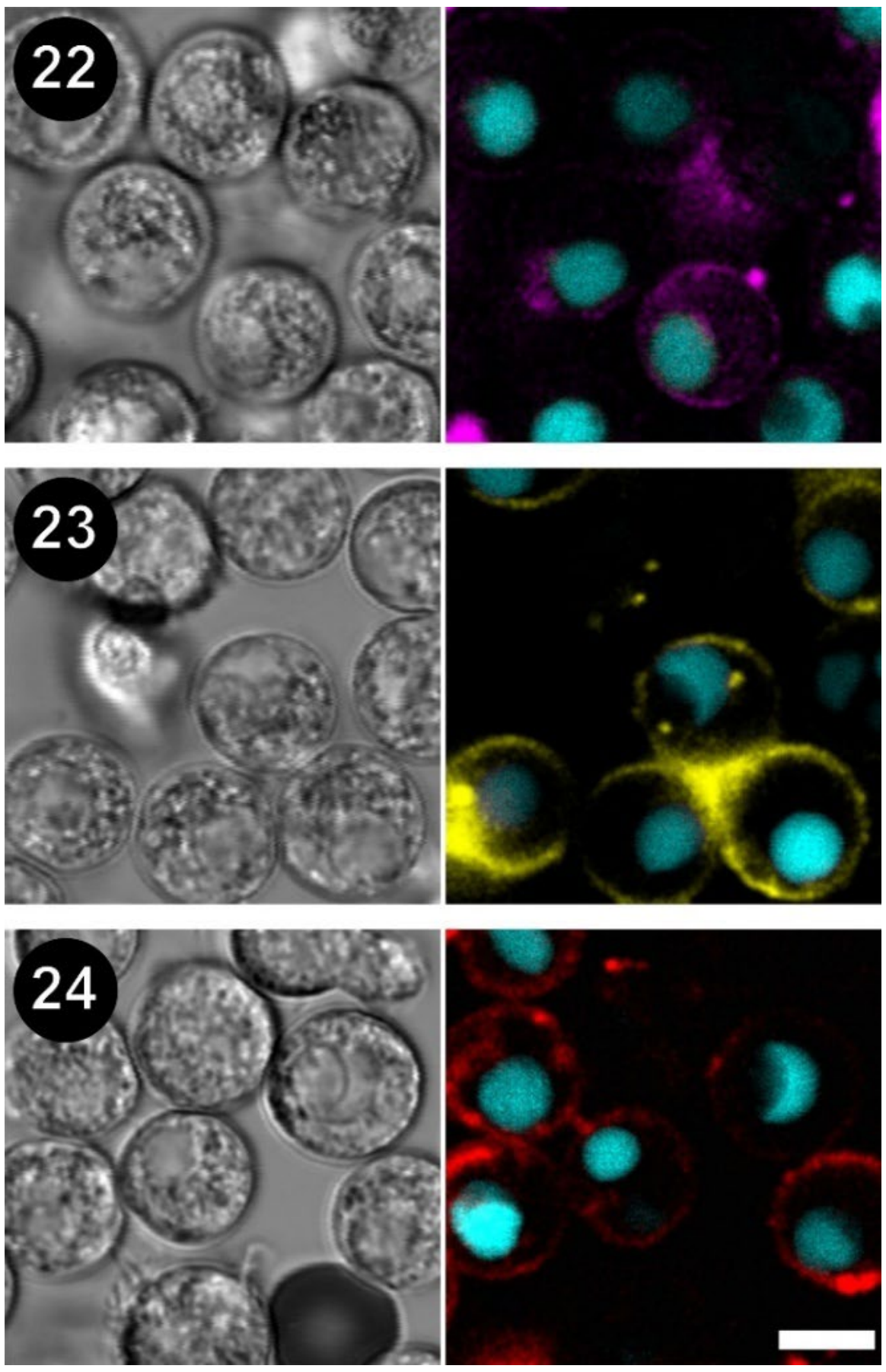

Supplementary Figure 28. Representative wash-free microscopy images (from 3 independent experiments) of apoptotic human neutrophils after incubation with fluorogenic peptides. Apoptotic human neutrophils were treated with Hoechst (1: 5,000; from 10 mg mL^{-1} stock; 15 min, cyan) followed by peptides **22** (25 μM, magenta), **23** (1 μM, yellow), and **24** (1 μM, red) for 15 min. Cells were imaged without any washing steps using a Leica Stellaris FALCON microscope (20× objective). Representative brightfield (left panels) and fluorescence (right panels) microscopy images are shown from three independent experiments. Excitation lasers: 405 nm (Hoechst), 452 nm (**22**), 554 nm (**23**), 650 nm (**24**). Scale bar: 10 μm.

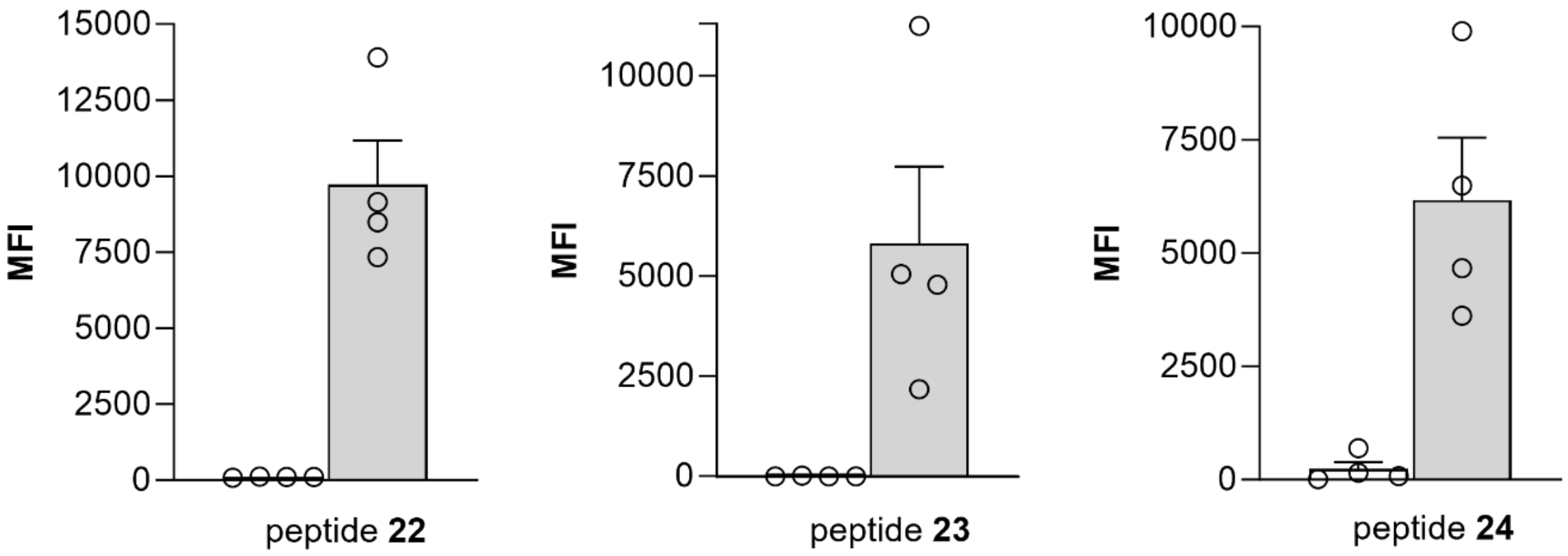

Supplementary Figure 29. Flow cytometry quantification of turn-on emission of peptides 22-24. Median Fluorescence Intensity (MFI) values of peptides **22** (25 µM), **23** (1 µM) and **24** (1 µM) in the absence (white bars) or presence of *E. coli* cells (grey bars) in PBS for 30 min (n=4, independent experiments).

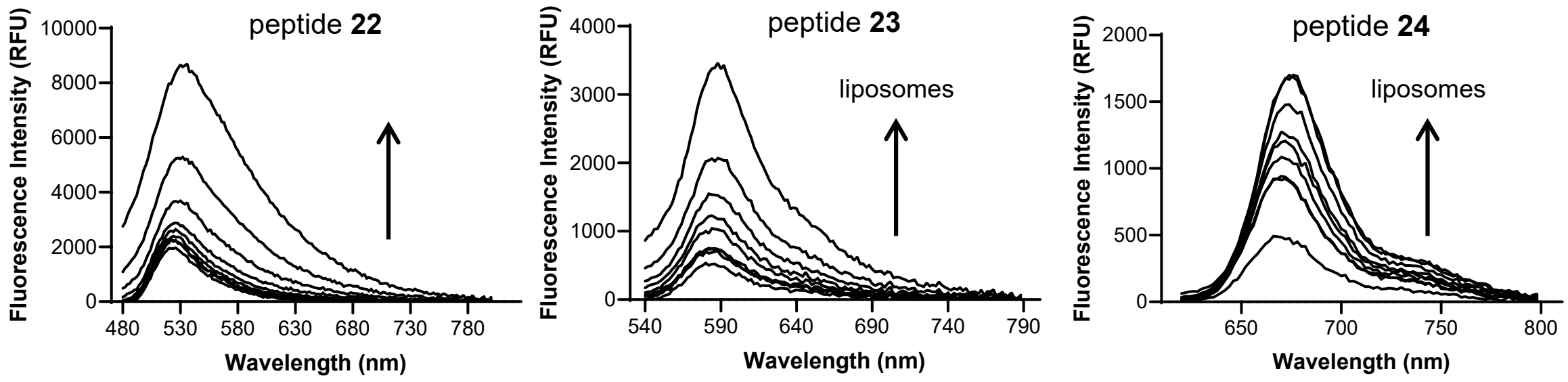

Supplementary Figure 30. Representative (from 3 independent experiments) fluorescence spectra of peptides **22** (10 μM), **23** (2.5 μM) and **24** (2.5 μM) in phosphatidylcholine liposome suspensions with increasing lipid content. λ_{exc}: 450 nm (**22**), 510 nm (**23**) and 590 nm (**24**).

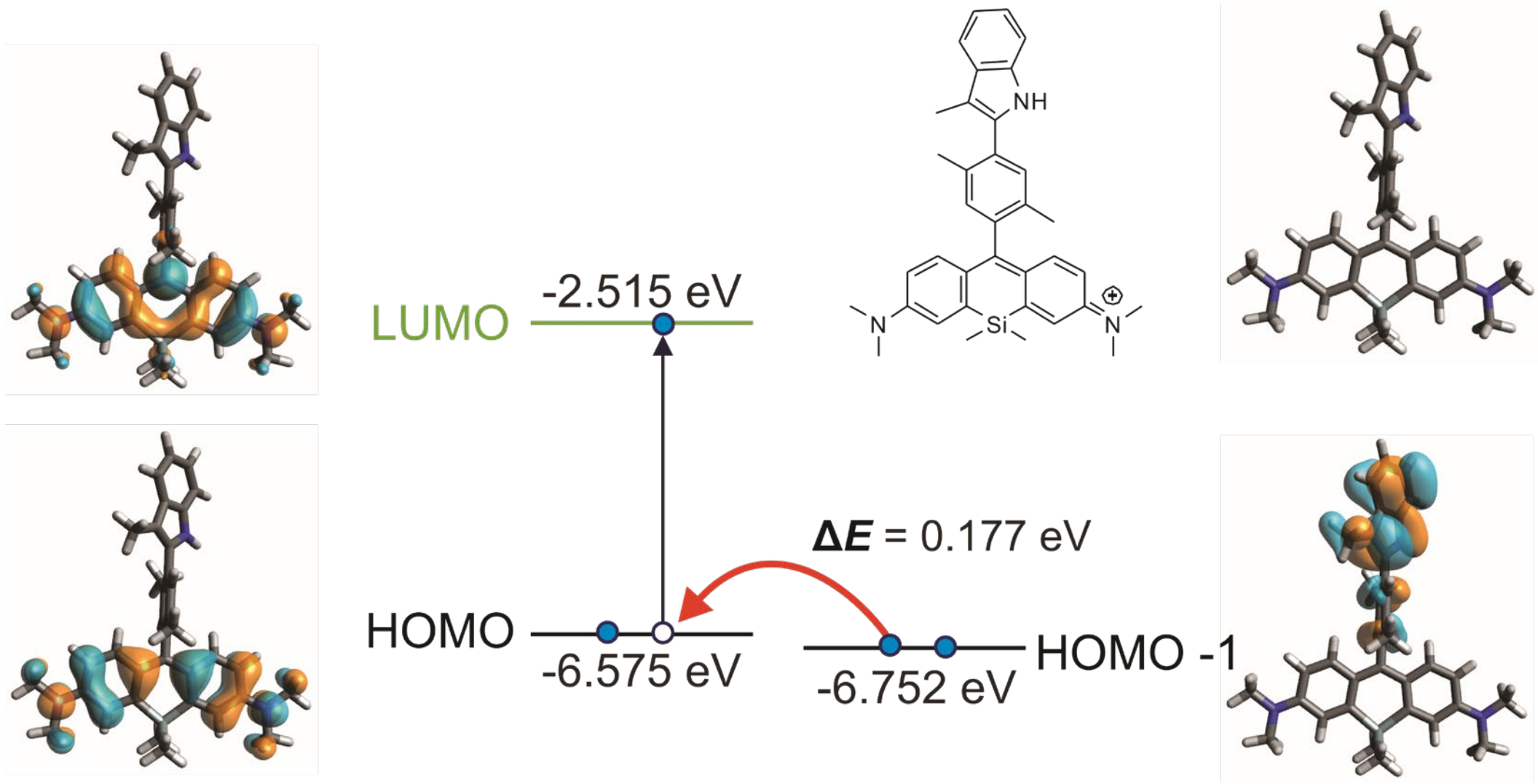

Supplementary Figure 31. Electron density and energy levels of HOMO-1, HOMO, and LUMO for compound Si-rhodamine-AA (**28**) in water, calculated at the optimized ground-state geometry using the M06-2X/def2-SVP level with SMD(water) solvation. The inset highlights the energy gap (ΔE) between HOMO and HOMO-1.

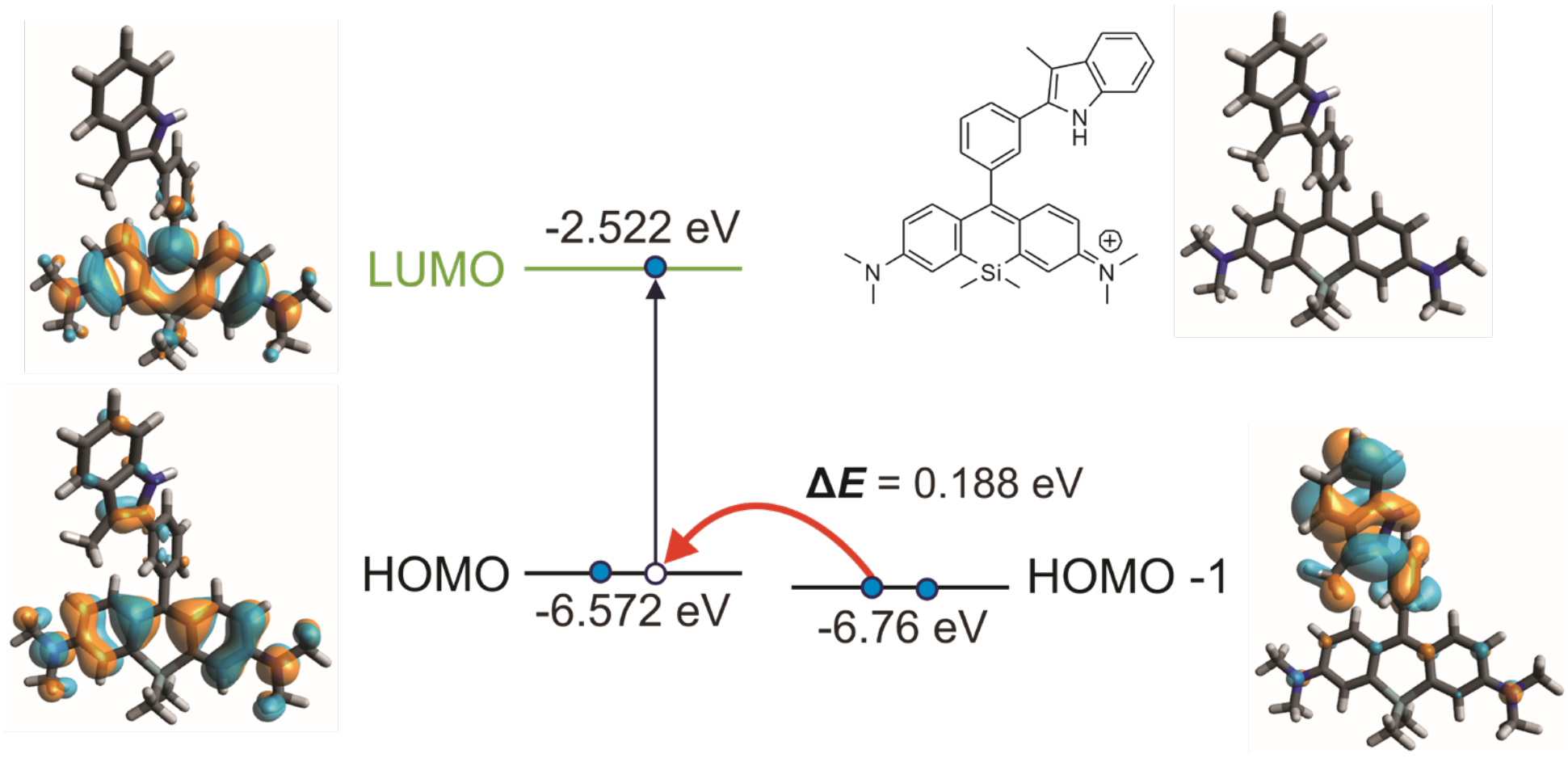

Supplementary Figure 32. Electron density and energy levels of HOMO-1, HOMO, and LUMO for compound Si-rhodamine-AA (**29**) in water, calculated at the optimized ground-state geometry using the M06-2X/def2-SVP level with SMD(water) solvation. The inset highlights the energy gap (ΔE) between HOMO and HOMO-1.

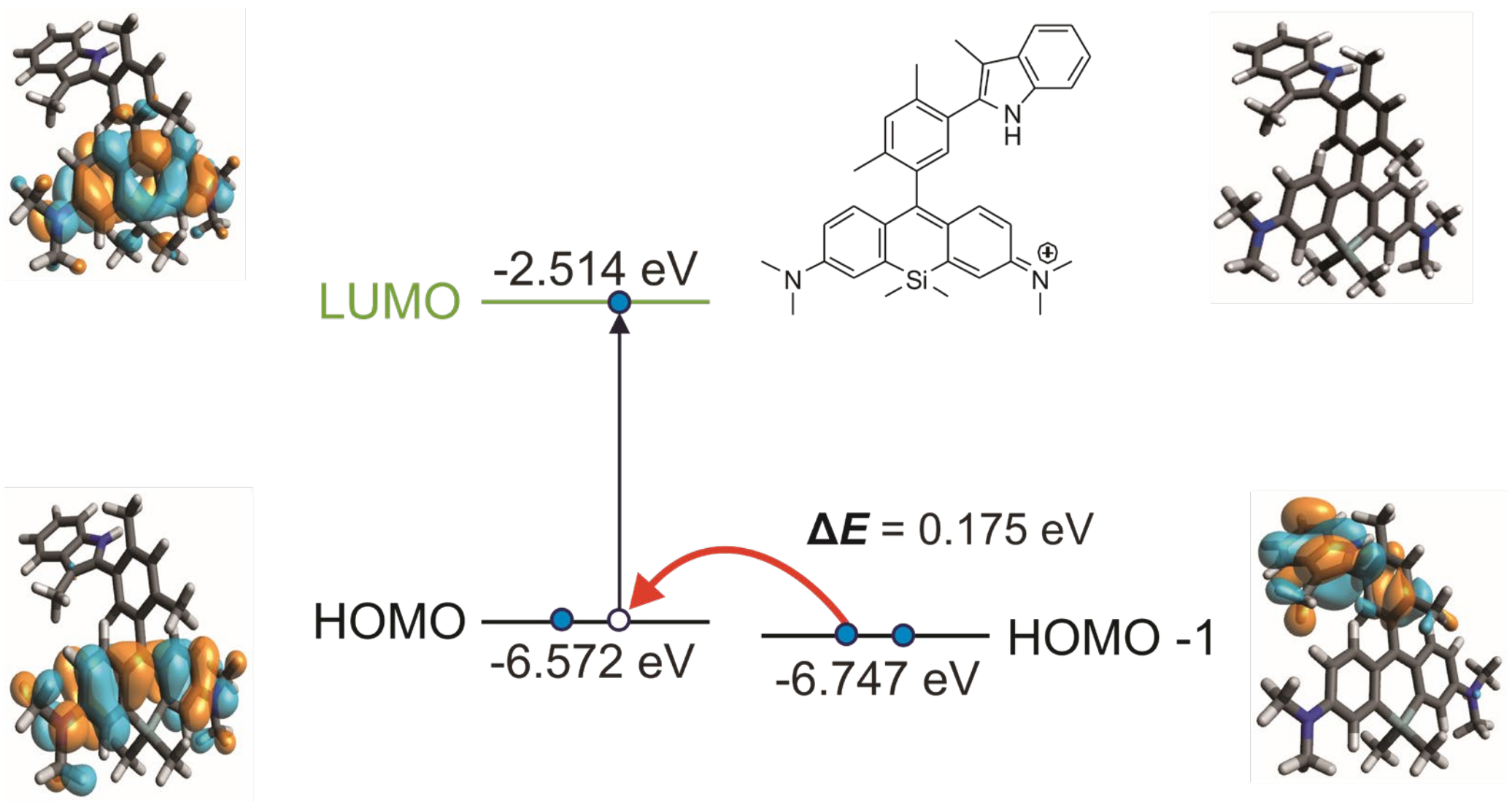

Supplementary Figure 33. Electron density and energy levels of HOMO-1, HOMO, and LUMO for compound Si-rhodamine-AA (**30**) in water, calculated at the optimized ground-state geometry using the M06-2X/def2-SVP level with SMD(water) solvation. The inset highlights the energy gap (ΔE) between HOMO and HOMO-1.

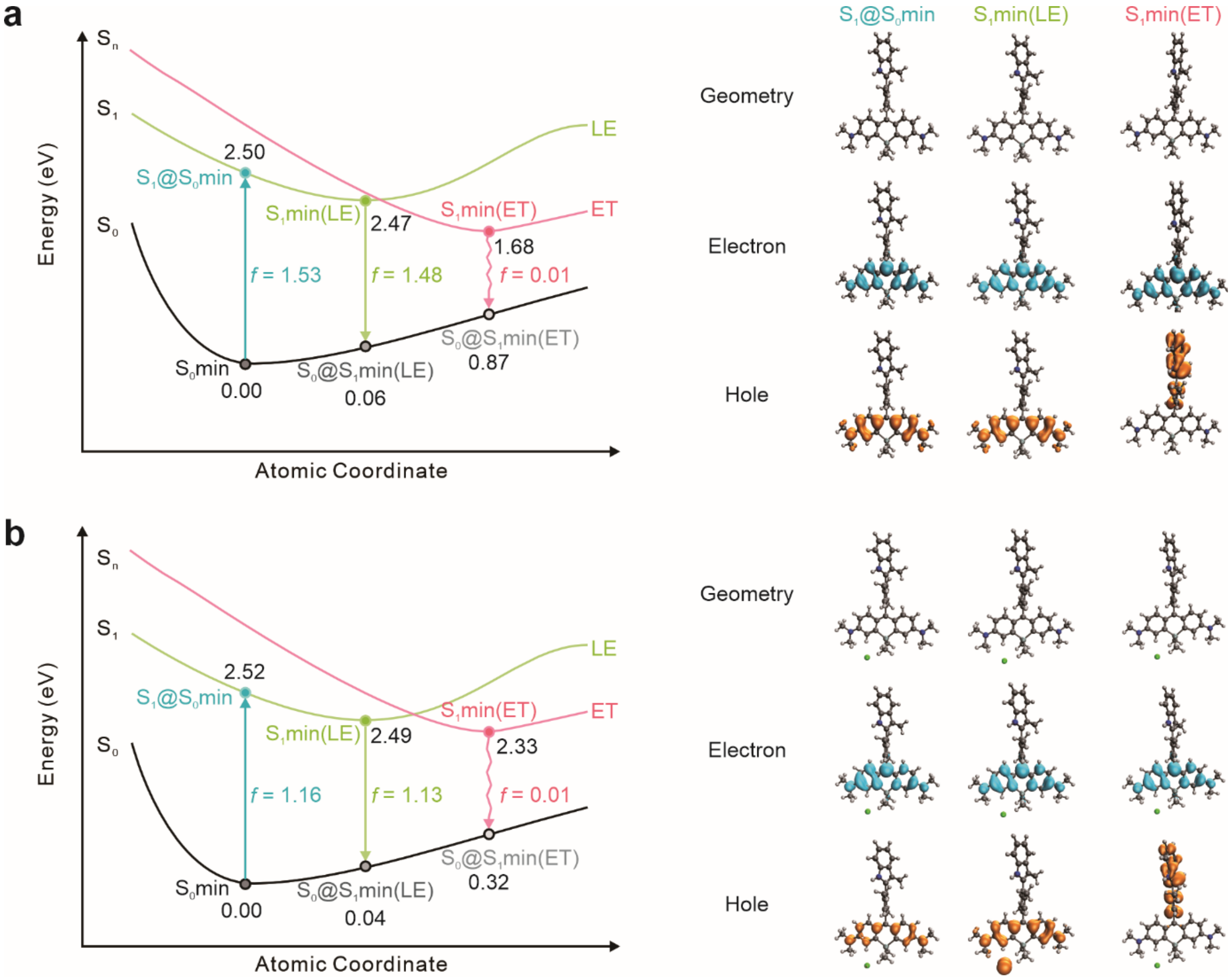

Supplementary Figure 34. Calculated excitation/de-excitation energy (including vertical oscillator strength f) of compound Si-rhodamine-AA (**28**) in (a) water and (b) toluene. The right panel shows optimized molecular structures in representative states, along with the corresponding electron and hole distributions. LE: Locally Excited state; ET: Electron Transfer state. The driving energy from the LE (bright) state to the ET (dark) state decreases from water to toluene, indicating that PeT is suppressed in nonpolar solvents. For clarity, the energy levels are not plotted to scale. The S_1 geometries were optimized at the M06-2X/def2-SVP level using the SMD solvation model within the linear-response (LR) formalism. Final single-point excited-state energies were calculated at the M06-2X/def2-TZVP level using the state-specific cLR formalism in conjunction with the SMD model in water (polar) or toluene (non-polar).

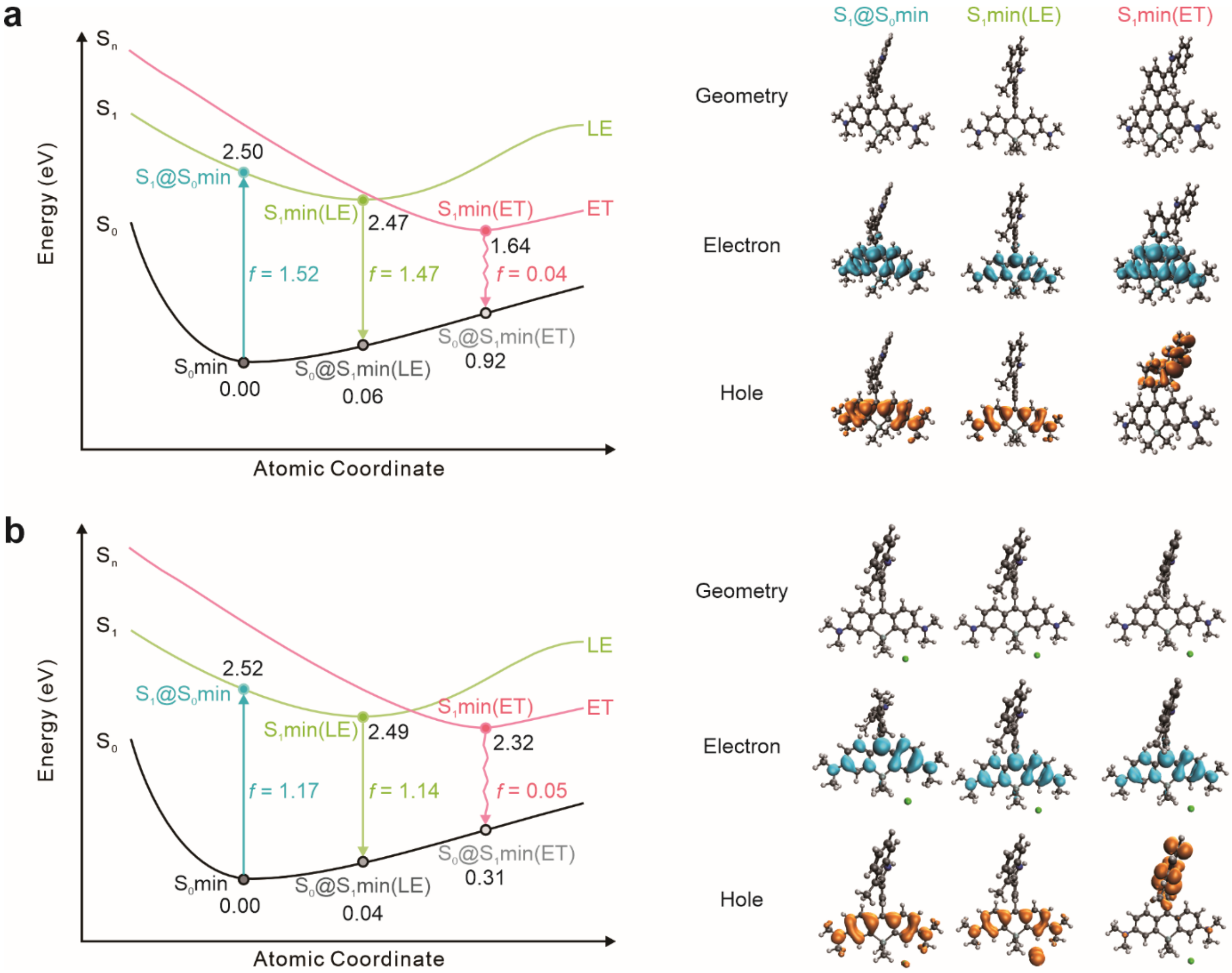

Supplementary Figure 35. Calculated excitation/de-excitation energy (including vertical oscillator strength *f*) of compound Si-rhodamine-AA (**29**) in (a) water and (b) toluene. The right panel shows optimized molecular structures in representative states, along with the corresponding electron and hole distributions. LE: Locally Excited state; ET: Electron Transfer state. The driving energy from the LE (bright) state to the ET (dark) state decreases from water to toluene, indicating that PeT is suppressed in nonpolar solvents. For clarity, the energy levels are not plotted to scale. The S_1 geometries were optimized at the M06-2X/def2-SVP level using the SMD solvation model within the linear-response (LR) formalism. Final single-point excited-state energies were calculated at the M06-2X/def2-TZVP level using the state-specific cLR formalism in conjunction with the SMD model in water (polar) or toluene (non-polar).

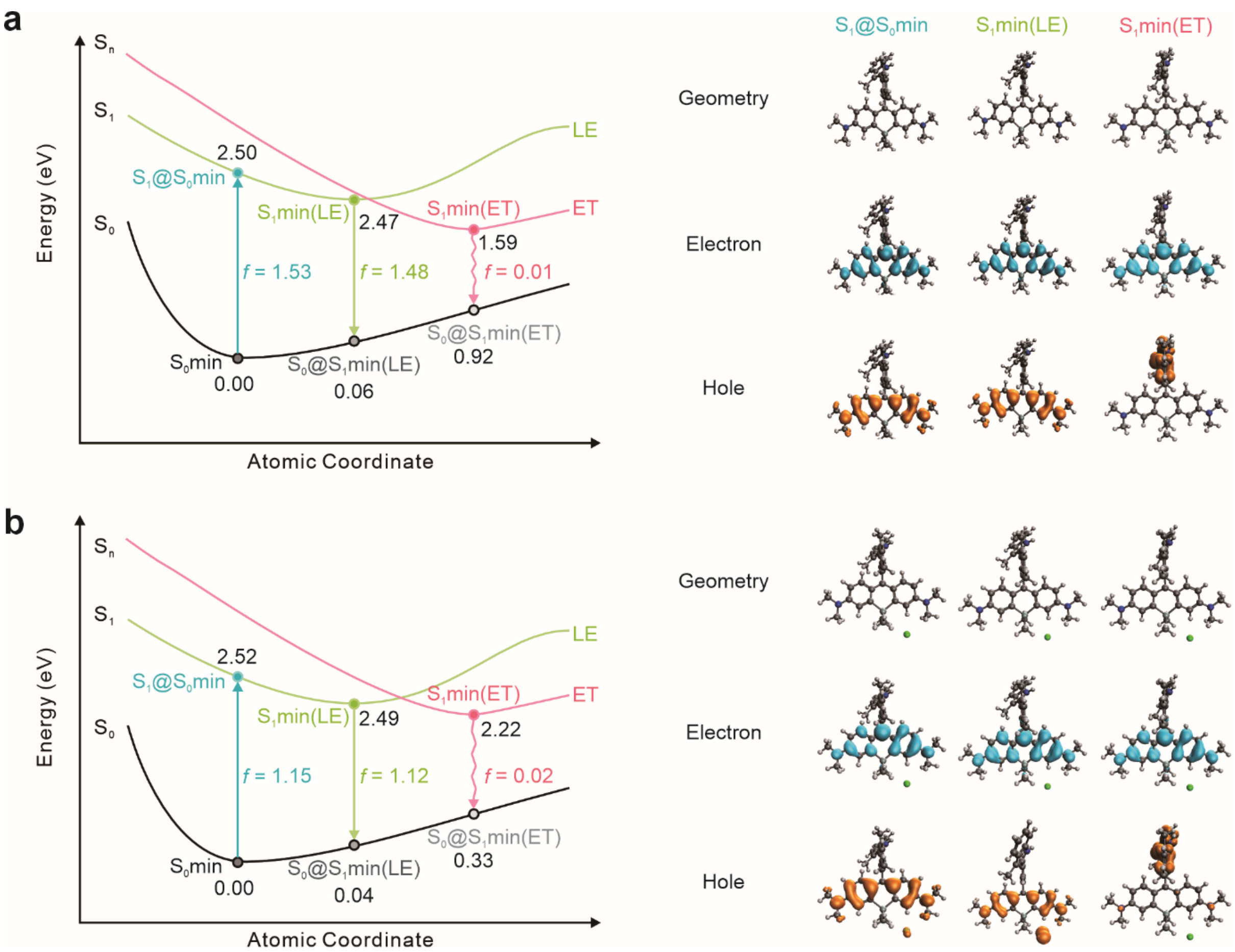

Supplementary Figure 36. Calculated excitation/de-excitation energy (including vertical oscillator strength *f*) of compound Si-rhodamine-AA (**30**) in (a) water and (b) toluene. The right panel shows optimized molecular structures in representative states, along with the corresponding electron and hole distributions. LE: Locally Excited state; ET: Electron Transfer state. The driving energy from the LE (bright) state to the ET (dark) state decreases from water to toluene, indicating that PeT is suppressed in nonpolar solvents. For clarity, the energy levels are not plotted to scale. The S_1 geometries were optimized at the M06-2X/def2-SVP level using the SMD solvation model within the linear-response (LR) formalism. Final single-point excited-state energies were calculated at the M06-2X/def2-TZVP level using the state-specific cLR formalism in conjunction with the SMD model in water (polar) or toluene (non-polar).

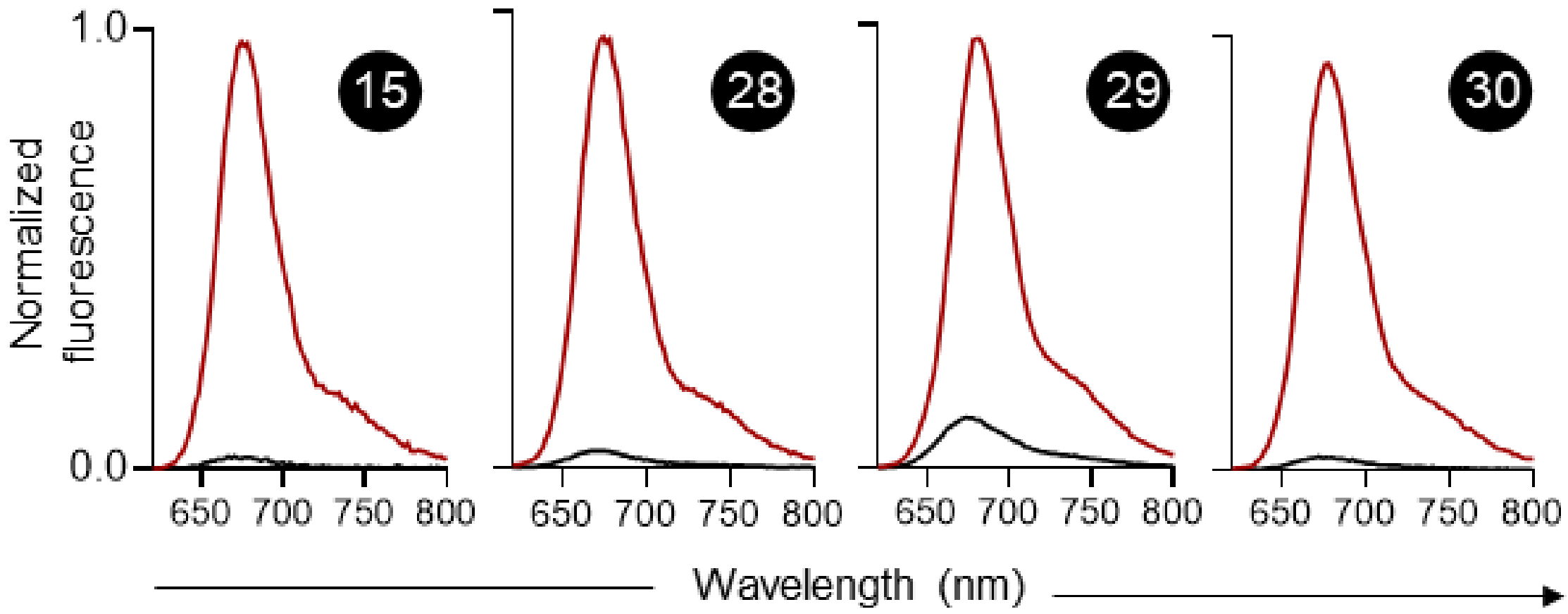

Supplementary Figure 37. Representative (from 3 independent experiments) fluorescence spectra of FgAAs **15**, **28**, **29**, and **30** (2.5-20 μM) in glycerol:water (red lines) and in water (black lines). λ_{exc}: 590 nm.

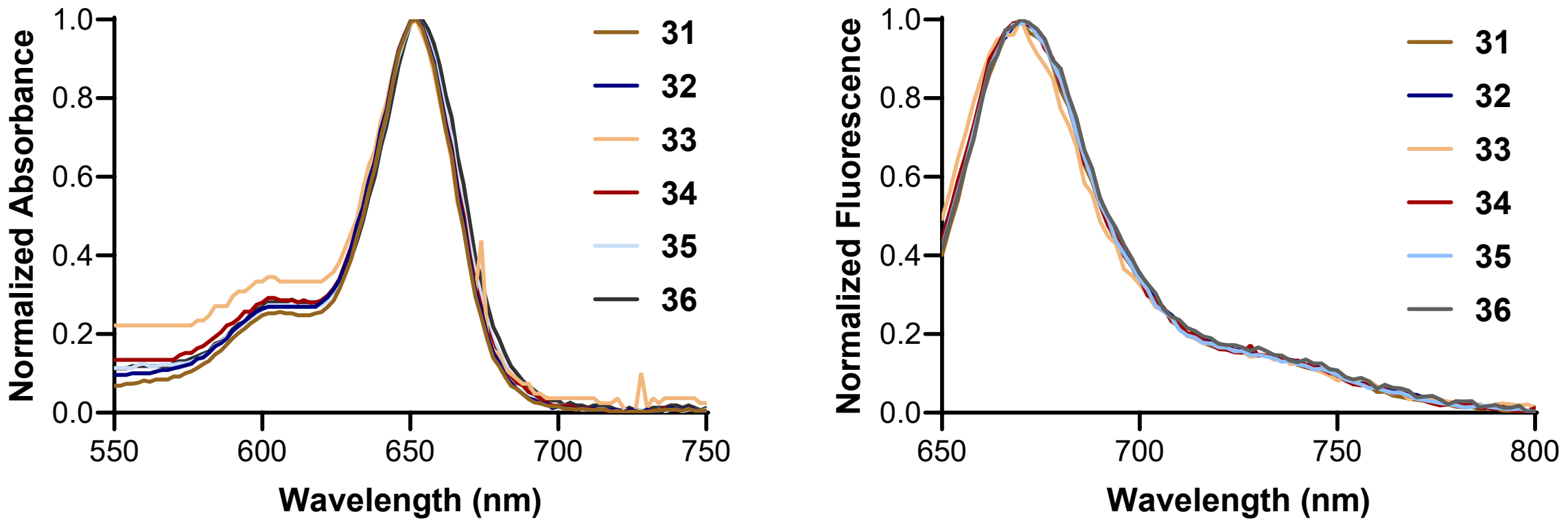

Supplementary Figure 38. Representative (from 3 independent experiments) normalized absorption (left) and emission (right) spectra of peptides **31-36** (all at 50 μM) in EtOH. λ_{exc}: 620 nm.

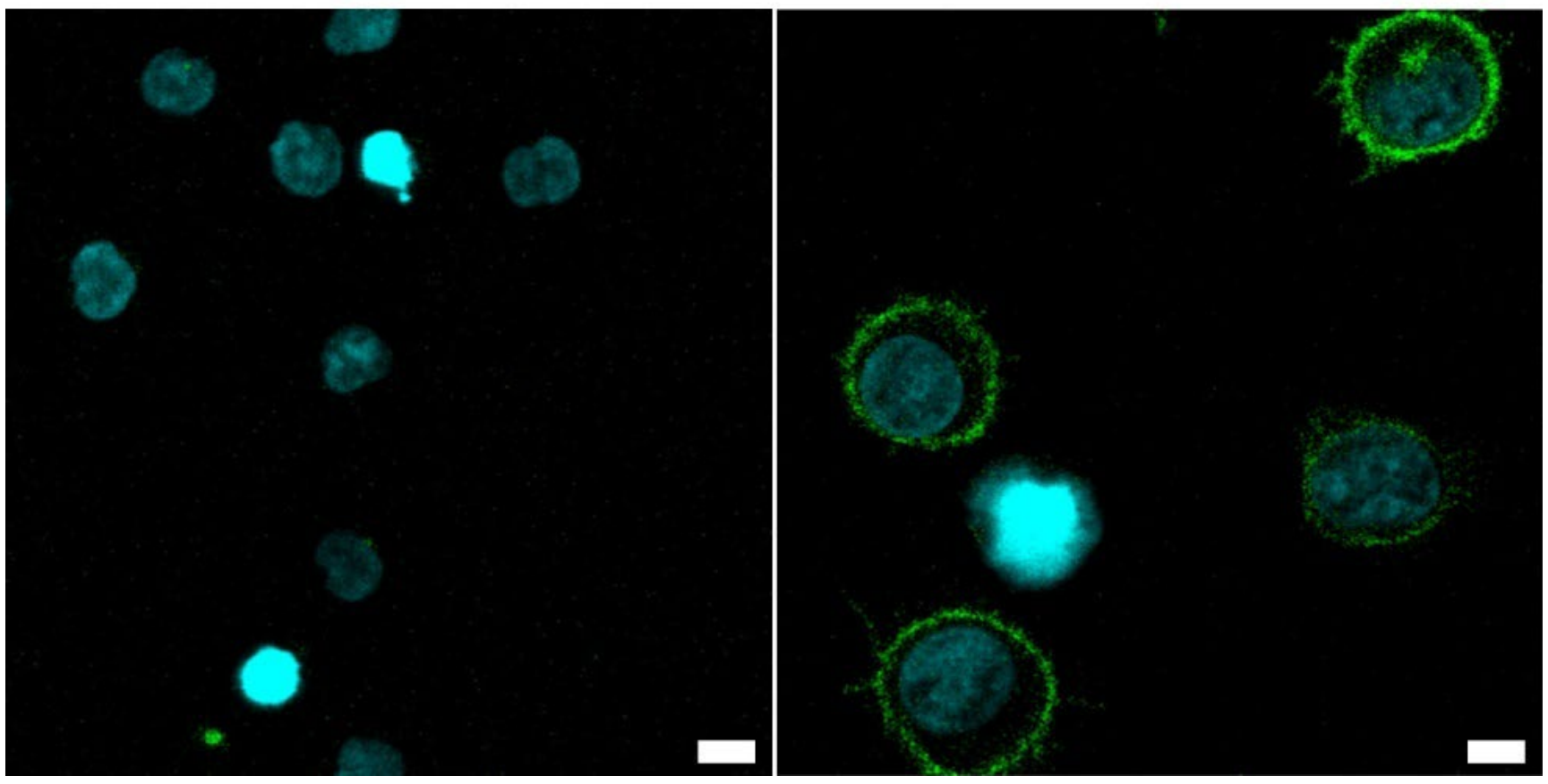

Supplementary Figure 39. Human activated T cells express detectable levels of PD-1 receptors. Representative (from 3 independent experiments) fluorescence microscopy images of non-activated and activated human T cells after incubation with Hoechst (1:5000, 15 min, cyan) and anti-PD-1 antibody (1:100, 20 min on ice, green). Cells were washed before fluorescence microscopy. Brightfield and fluorescence microscopy images were captured on the Leica Stellaris FALCON microscope (20× objective), and representative fields are shown from three independent experiments. Scale bar: 5 µm.

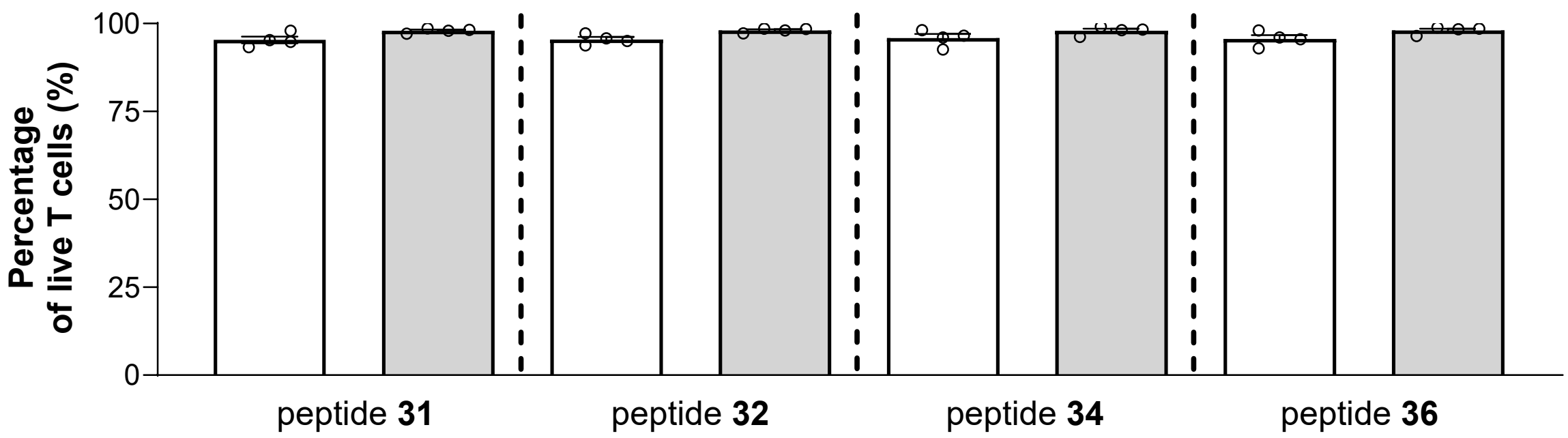

Supplementary Figure 40. Cytotoxicity assays with fluorogenic peptides. Non-activated human T cells (white bars) and activated human T cells (grey bars) were treated with fluorogenic peptides (**31**, **32**, **34**, and **36**; all at 5 μM) for 30 min. The cytotoxicity was measured by flow cytometry using the live-dead stain Zombie Violet (1:100). In all cases, the percentage of live cells was higher than 98%. Values are presented as means ± SEM (n=4, independent experiments).

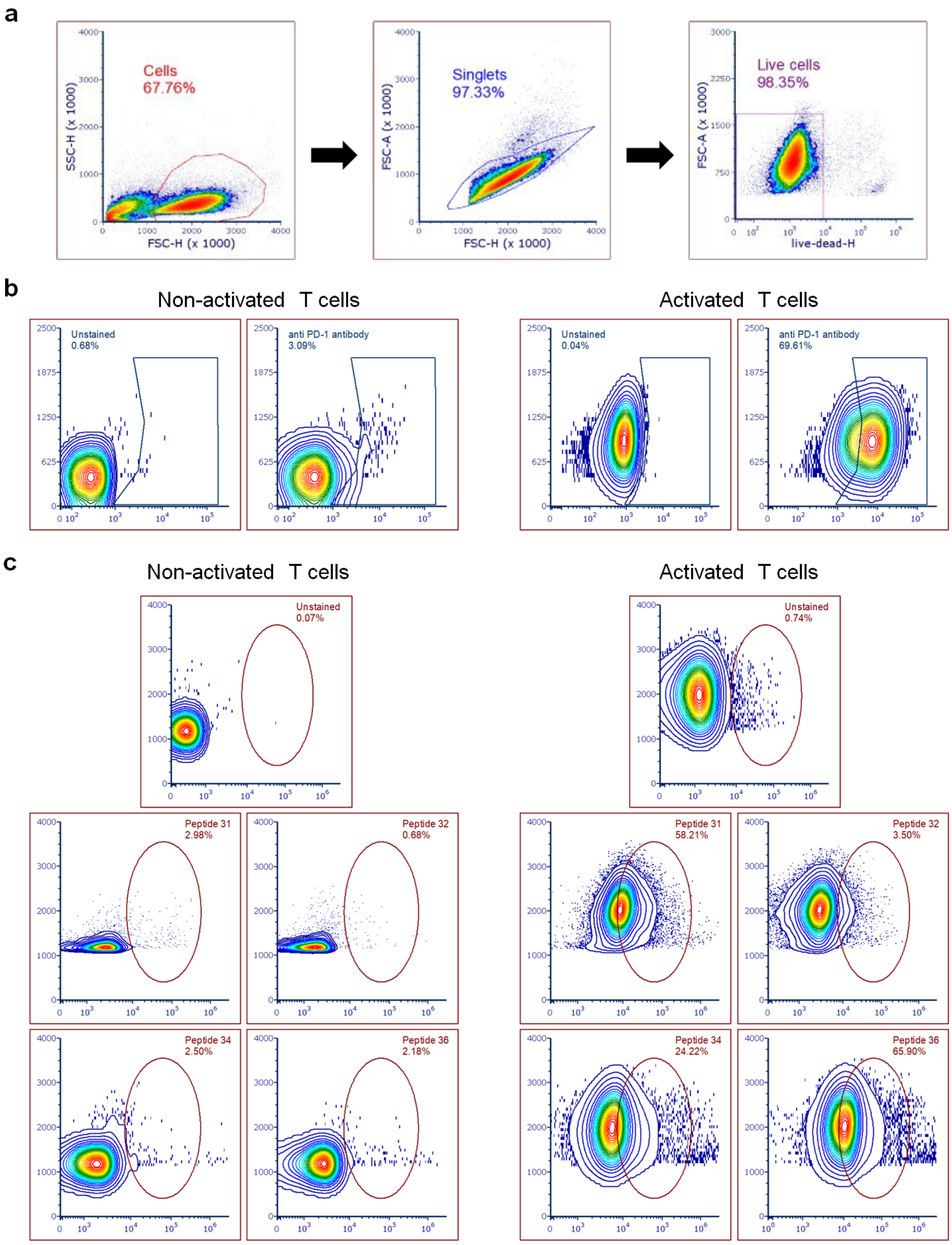

Supplementary Figure 41. Gating strategy for T cell labeling with PD-1-binding peptides. a) Stepwise gating strategy. Cells were gated first based on the forward and side scattering, eliminating all debris. Next, singlets were gated based on forward scatter area versus height. Live cells were selected from the singlets based on live-dead staining (Zombie Violet, 1:100). b, c) Contour plots showing staining of anti PD-

1 antibody (b) and peptides **31**, **32**, **34** and **36** (c) with PD-1 negative T cells and PD-1 positive T cells gated on unstained samples. Quantification was performed as percentages of cells with positive PD-1 signals. Data was acquired on FCS Express 7 (De Novo Software).

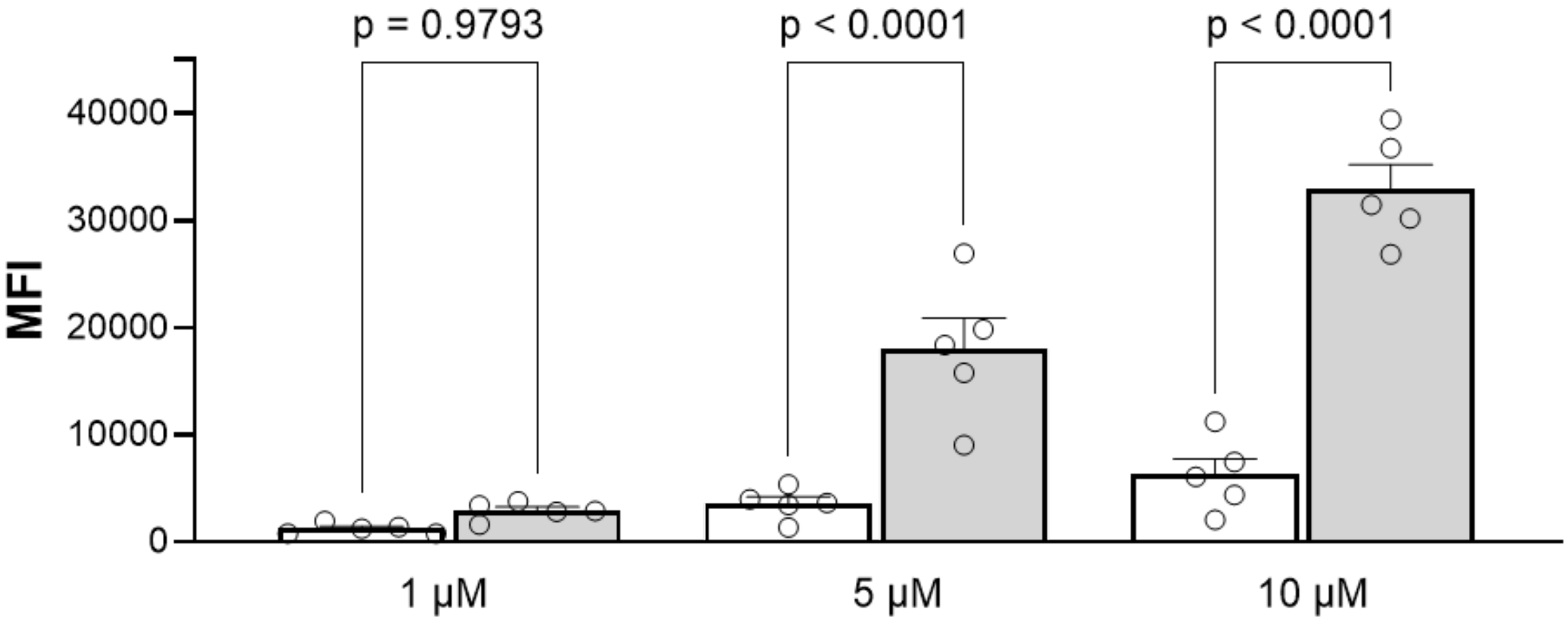

Supplementary Figure 42. Fluorescence emission of peptide 36 upon incubation with T cells at different concentrations. Median Fluorescence Intensity (MFI) values of non-activated T cells (white bars) and activated T cells (grey bars) after incubation with peptide **36** at different concentrations. Data were acquired on a NovoCyte flow cytometer and presented as means ± SEM (n=5, independent experiments). P values were obtained from one-way ANOVA using multiple comparisons.

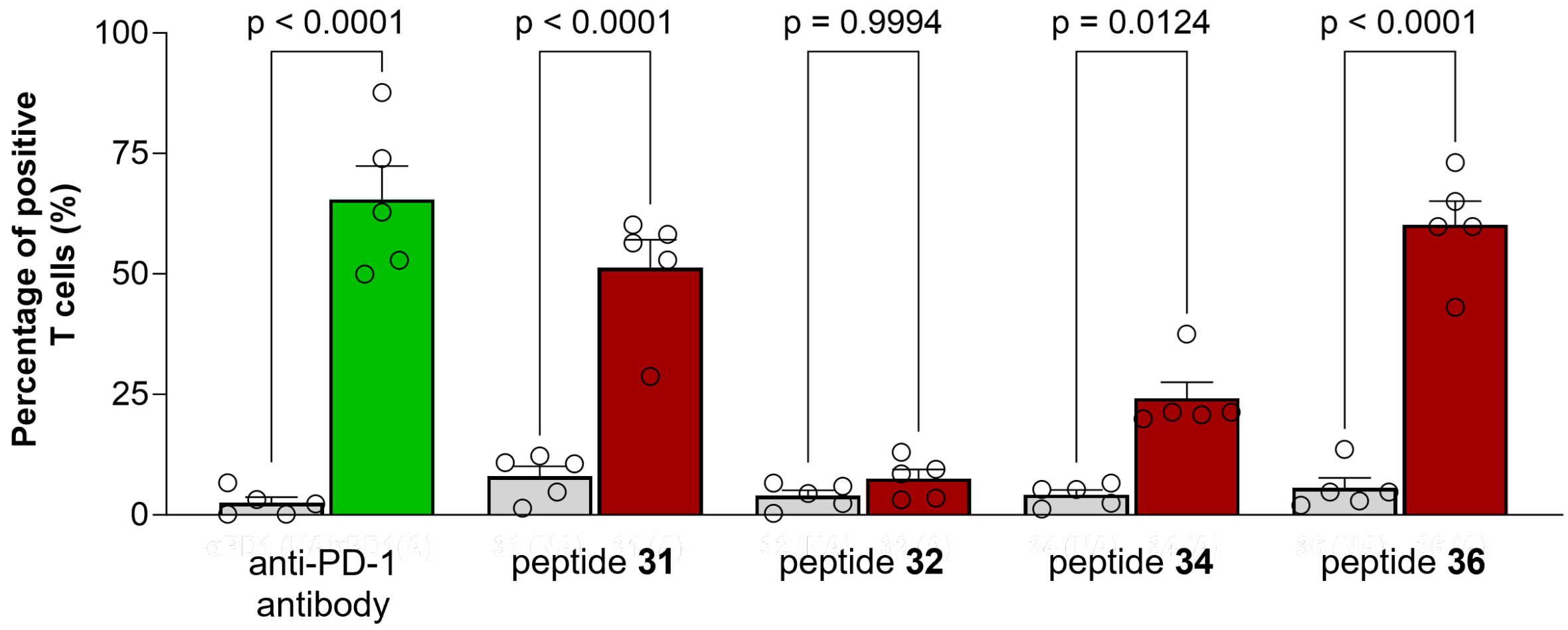

Supplementary Figure 43. Fluorogenic PD-1-binding peptides for T cell labeling. Percentages of PD-1-positive cells in non-activated T cells (grey bars) and activated T cells (green and red bars for anti-PD-1 and fluorogenic peptides, respectively) after labeling with anti-PD-1 antibody (20 min on ice) or with peptides **31**, **32**, **34** and **36** (5 µM, 30 min). Data were acquired on a NovoCyte flow cytometer and presented as means ± SEM (n=5, independent experiments). P values were obtained from one-way ANOVA using multiple comparisons.

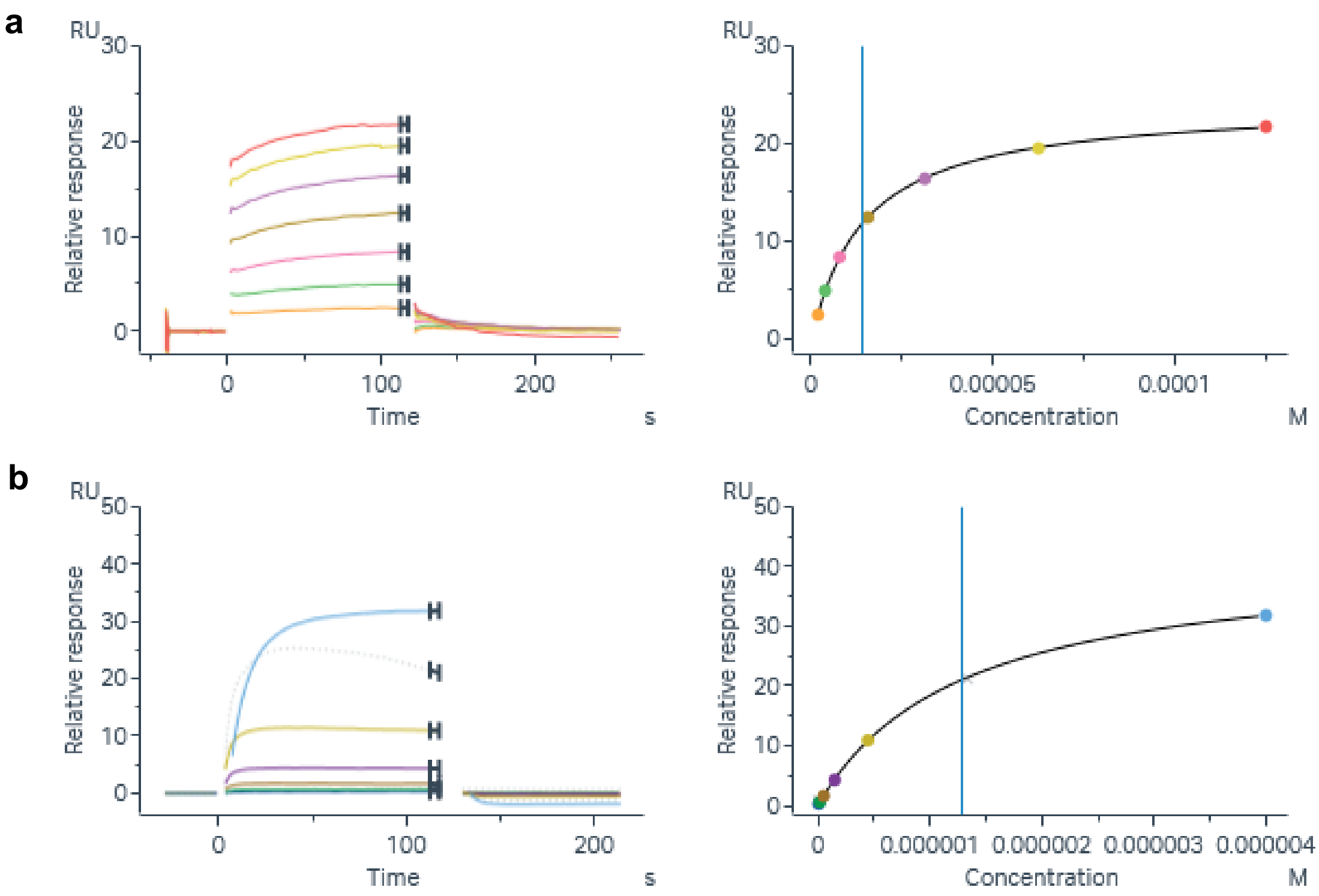

Supplementary Figure 44. Surface Plasmon Resonance binding assays. a) Sensorgram (left) and steady-state plot (right) of the binding between peptide **36** (concentration range: 2 µM to 125 µM) and immobilized PD-1 protein. The equilibrium dissociation constant (K_D) of peptide **36** was determined as 14 µM. b) Sensorgram (left) and steady-state plot (right) of the binding between the native ligand for PD-1 (PD-L1) and immobilized PD-1 protein. PD-L1 exhibited a K_D in the low micromolar range (1.3 µM).

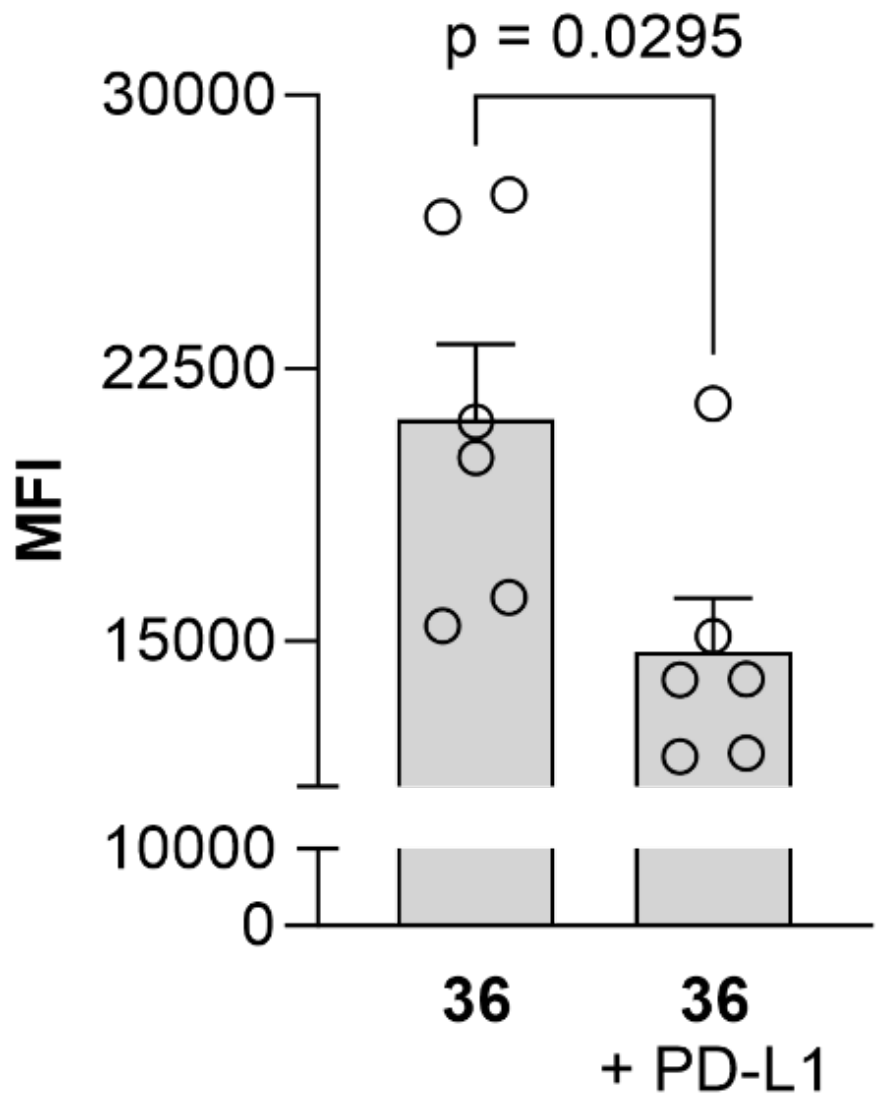

Supplementary Figure 45. Competition experiments with PD-L1. Activated T cells were incubated with PD-L1 (5 uM) for 20 min on ice prior to incubation with peptide **36** (5 μM, 30 min). Data were acquired on a NovoCyte flow cytometer and presented as means ± SEM (n=6, independent experiments). P values were obtained from unpaired t-test analysis.

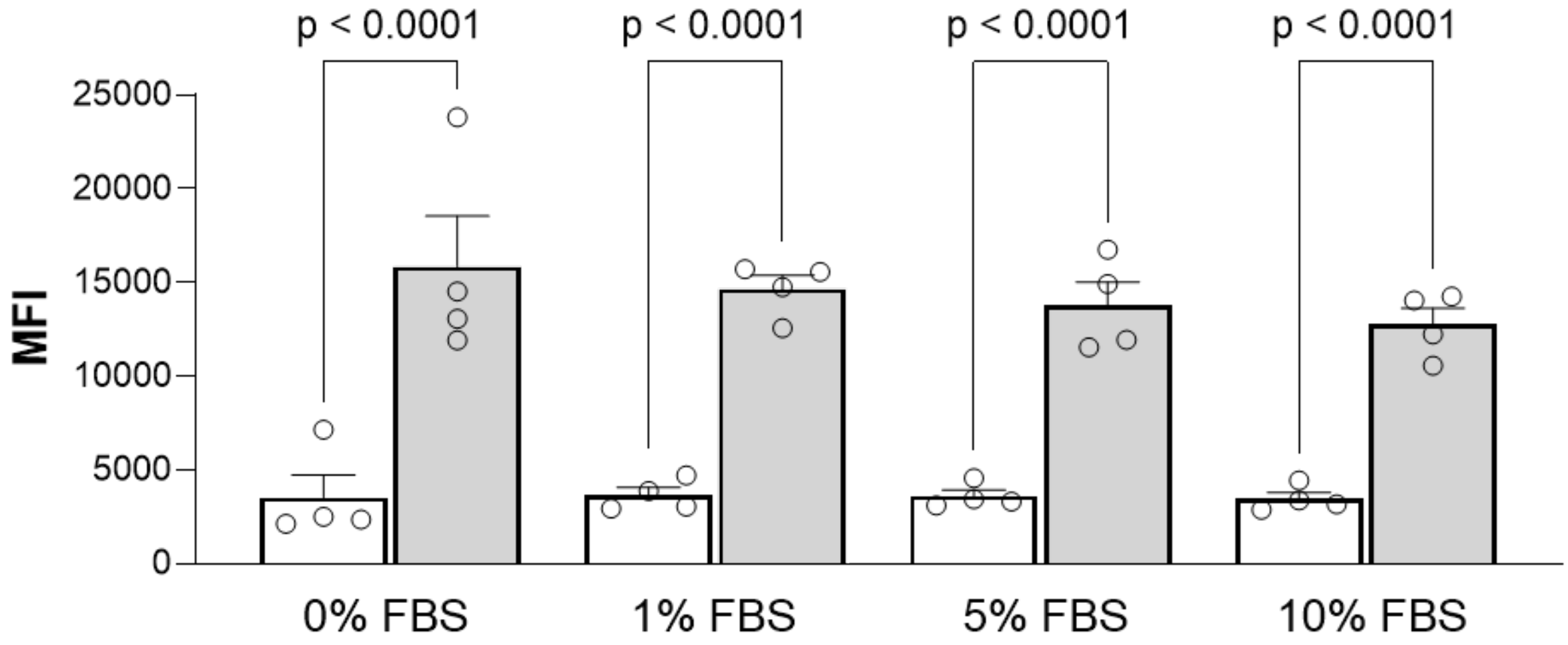

Supplementary Figure 46. Fluorescence analysis in media supplemented with serum. Median Fluorescence Intensity (MFI) values of non-activated T cells (white bars) and activated T cells (grey bars) after incubation with peptide **36** (5 µM, 30 min) in DMEM media with different percentages of FBS. Data were acquired on a NovoCyte flow cytometer and presented as means ± SEM (n=4, independent experiments). P values were obtained from one-way ANOVA using multiple comparisons.

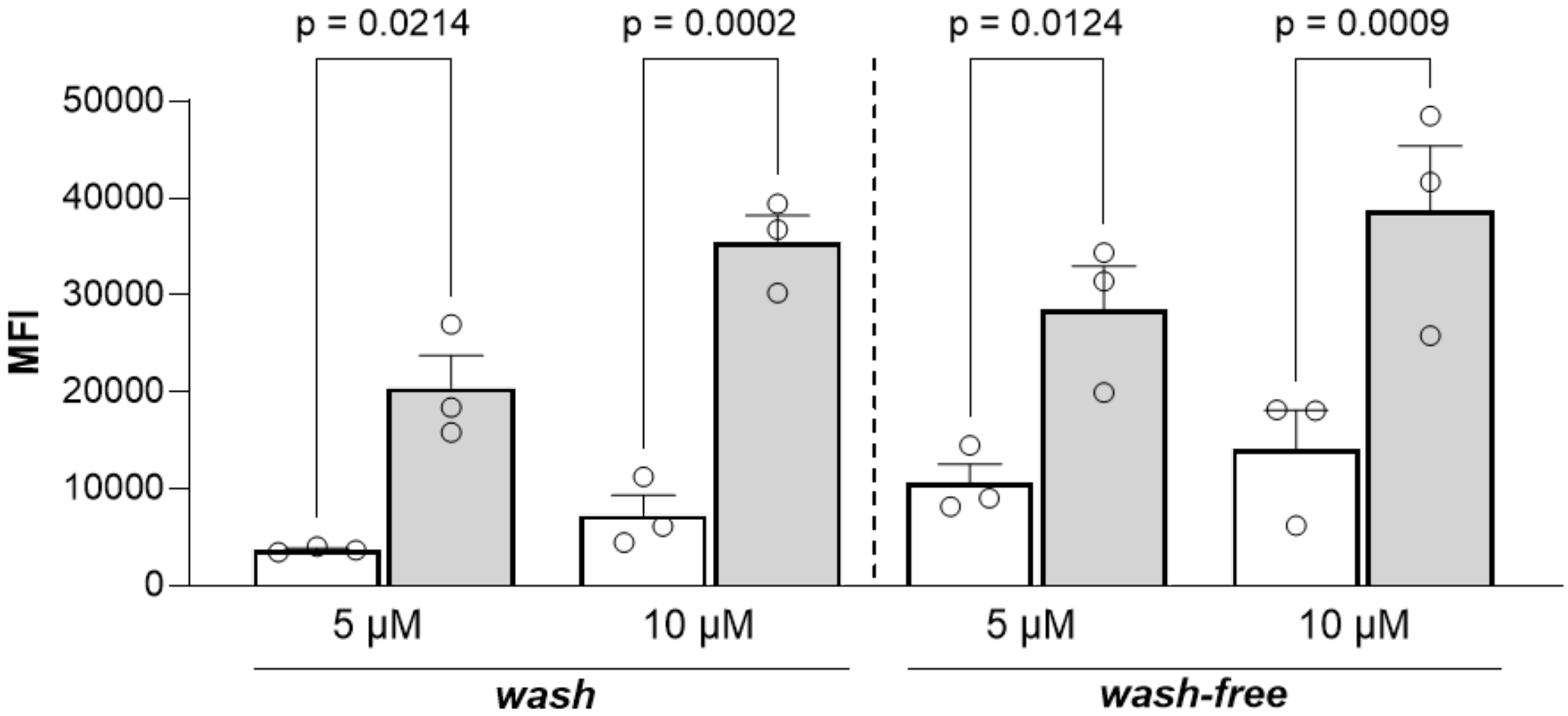

Supplementary Figure 47. Flow cytometry analysis of T cells under wash or wash-free conditions. Median Fluorescence Intensity (MFI) in non-activated T cells (white bars) and activated T cells (grey bars) after labeling with peptide **36** at different concentrations. Flow cytometry analysis was performed imaging after washing once with PBS (left) or under wash-free conditions (right). Data were acquired on a NovoCyte flow cytometer and presented as means ± SEM (n=3, independent experiments). P values were obtained from one-way ANOVA using multiple comparisons.

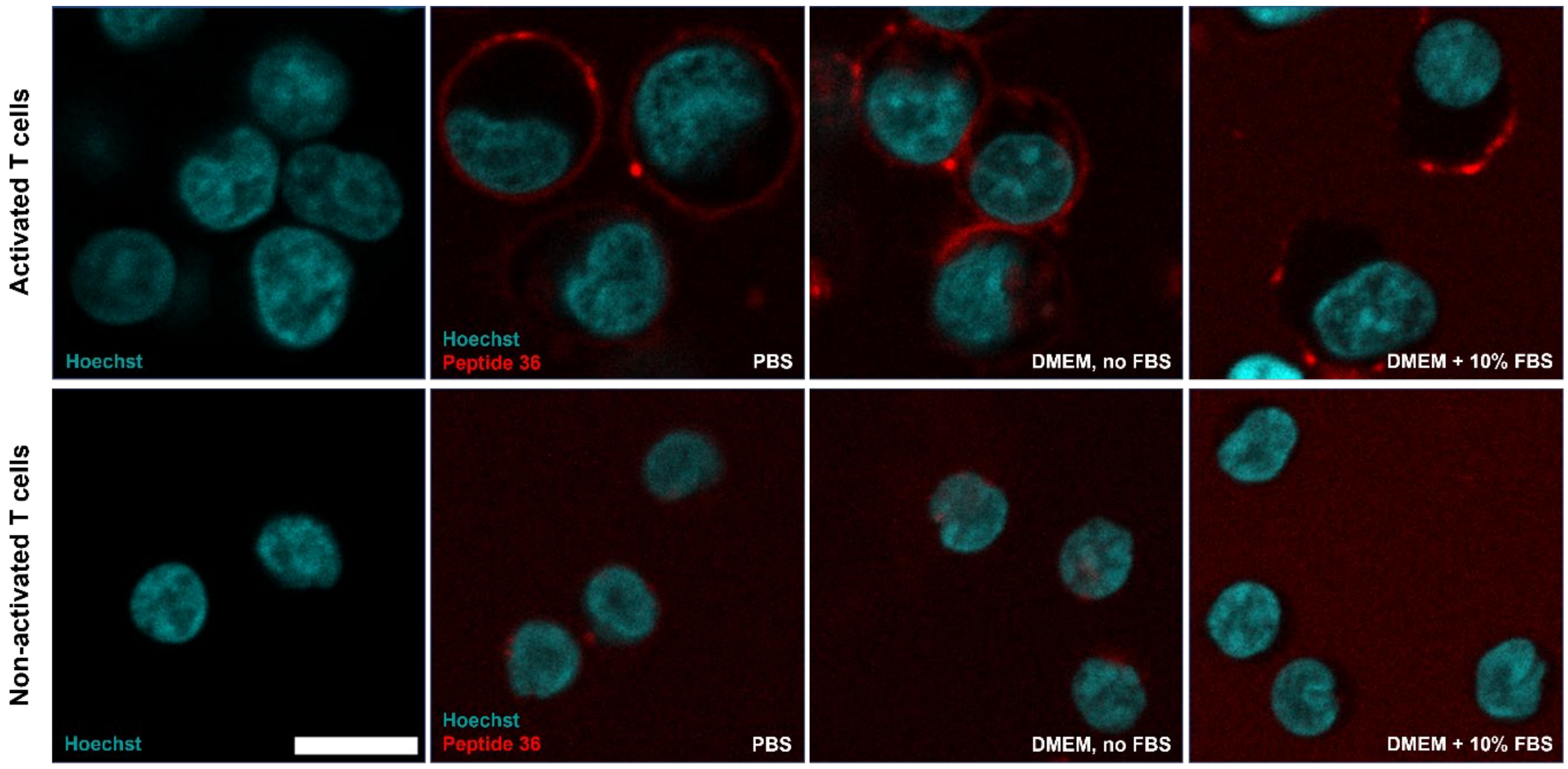

Supplementary Figure 48. Representative (from 3 independent experiments) fluorescence microscopy images of activated T cells and non-activated T cells after addition of peptide **36** (5 µM, 30 min, red) in different media and under wash-free conditions. Cells were counterstained with Hoechst 33342 (cyan). $\lambda_{exc/em}$: 405 nm/430-492 nm (Hoechst 33342), 650 nm/661-750 nm (peptide **36**)). Scale bar: 10 µm.

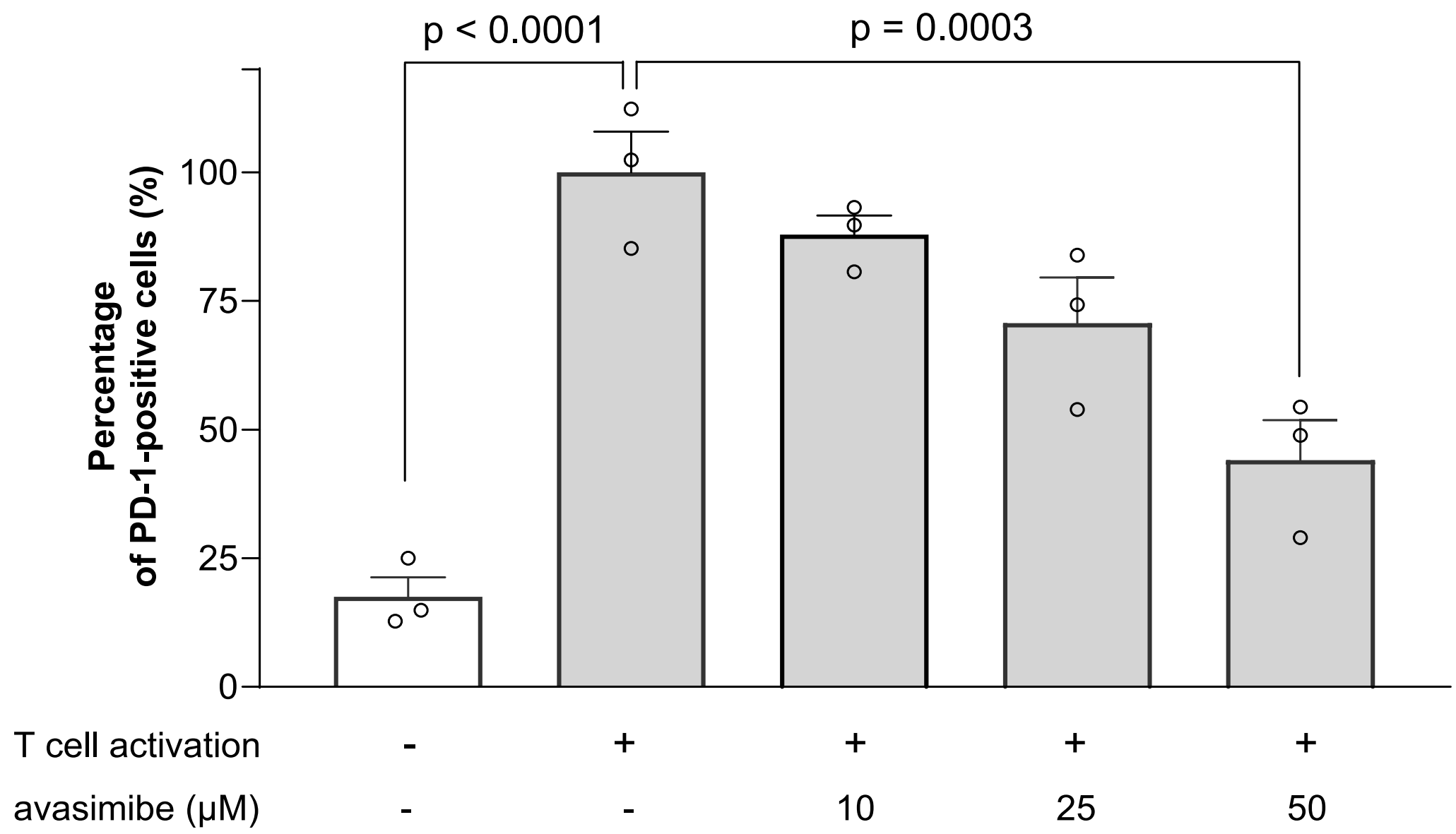

Supplementary Figure 49. Anti-PD-1 antibody staining after treatment with avasimibe. Non-activated T cells (white bar) and activated T cells (grey bars) were incubated with different concentrations of avasimibe for 24 h before harvesting. PD-1 expression was measured using an anti-PD-1 antibody (1:100). Data presented as means ± SEM (n=3, independent experiments). P values were obtained from one-way ANOVA.

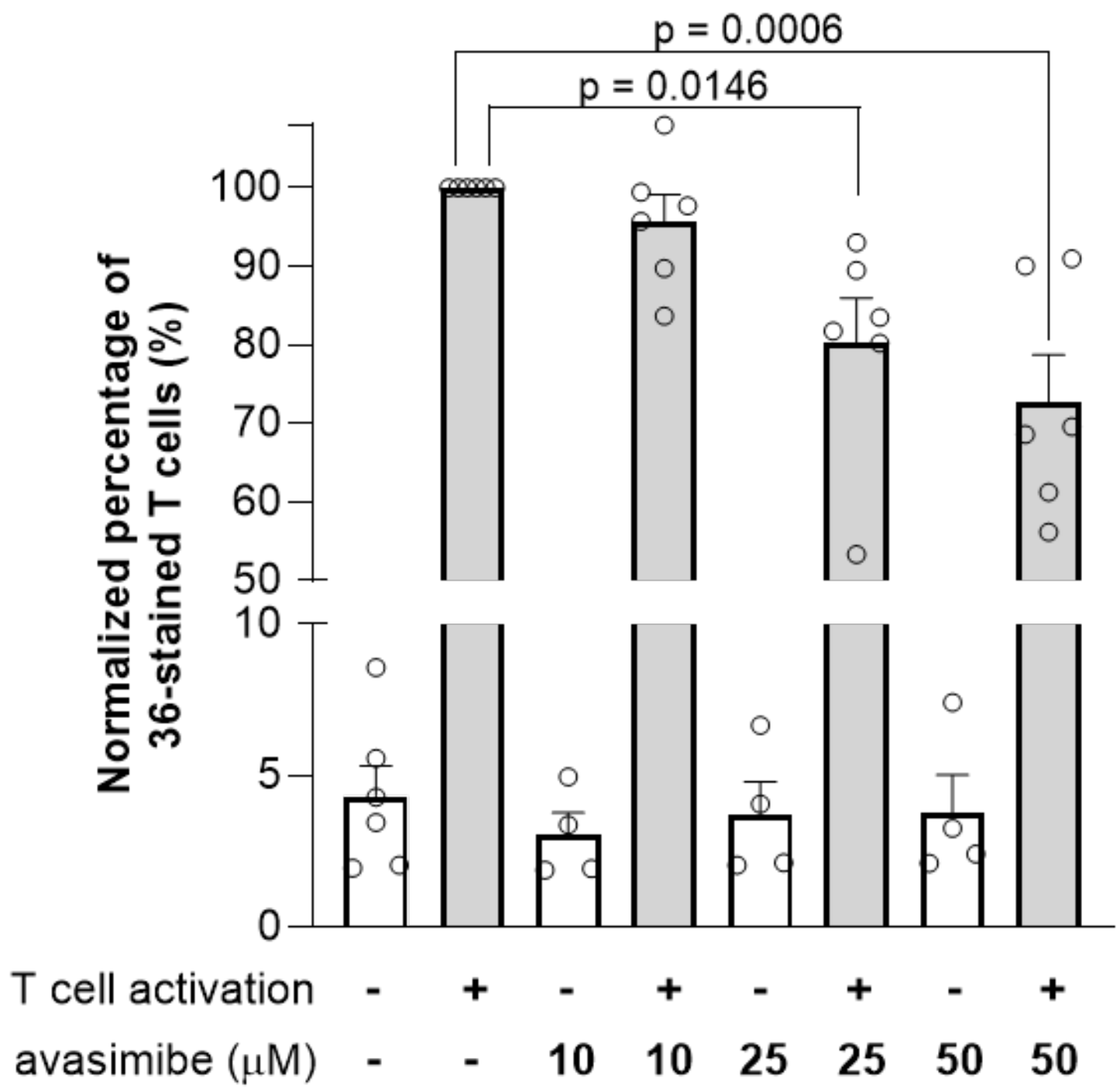

Supplementary Figure 50. Peptide 36 labeling of T cells after treatment with avasimibe. Normalized percentages of labeled T cells after treatment with different concentrations of avasimibe (0-50 μM, 24 h) and subsequent incubation with peptide **36** (5 μM, 30 min). Data presented as means ± SEM (n=4, independent experiments). P values were obtained from one-way ANOVA.

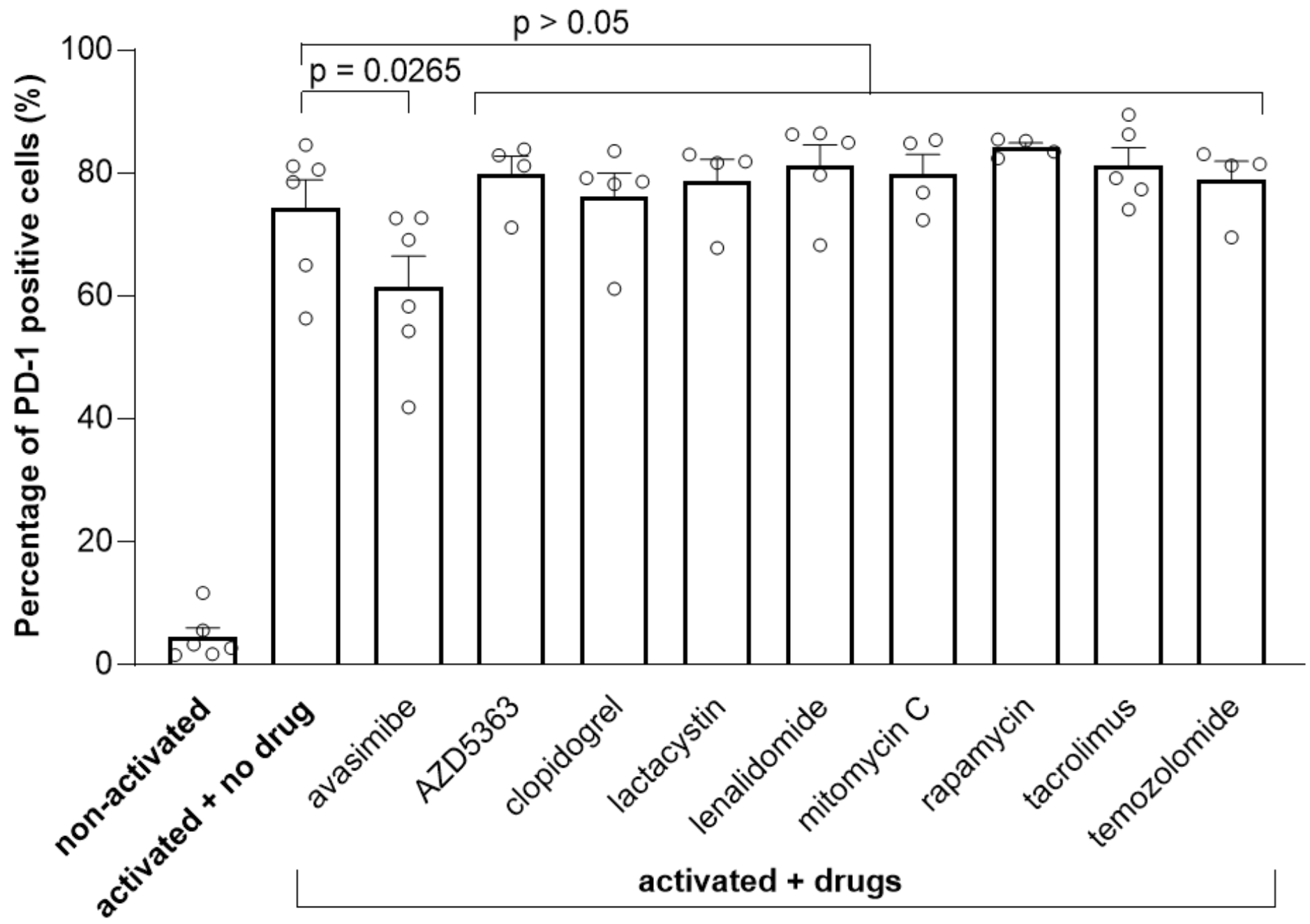

Supplementary Figure 51. Drug screening to identify modulators of PD-1 expression in human activated T cells. Percentages of labeled T cells after incubation with immune-targeting drugs (experimental conditions in Supplementary Table 2) and peptide **36** (5 μM, 30 min). Data presented as means ± SEM (n=5, independent experiments). P values were obtained from one-way ANOVA.

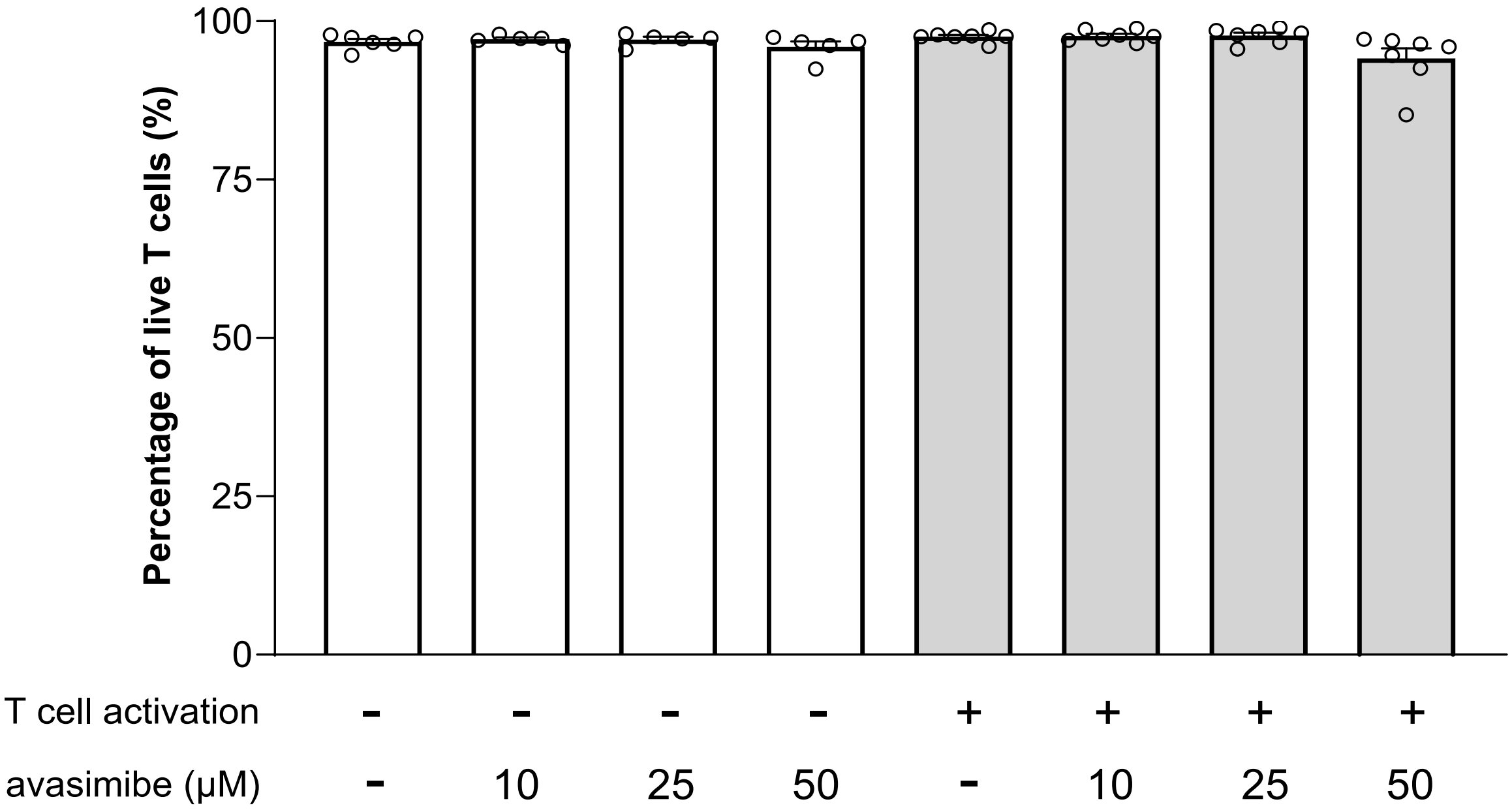

Supplementary Figure 52. Cytotoxicity assays of T cells after incubation with avasimibe. Non-activated human T cells (white bars) and activated human T cells (grey bars) were treated with avasimibe at the indicated concentrations for 24 h before harvesting. Cytotoxicity was measured by flow cytometry using the live-dead stain Zombie Violet (1:100). In all cases, the percentage of live cells was higher than 95%. Values are presented as means ± SEM (n=5, independent experiments).

Supplementary Table 1. Stability conditions tested for amino acids **10**-**15** and **28**. Reaction conditions: amino acid (20-40 µM, r.t.). [a] 1 h, [b] 10 eq DTT, [c] 5 min. Purity determined by HPLC (UV detection: 300 nm).

**conditions**	**amino acids**						
	**10**	**11**	**12**	**13**	**14**	**15**	**28**
50% ACN, 50% H_2O	≥99%	≥99%	≥99%	≥99%	≥99%	≥99%	≥99%
95% TFA, 2.5% TIS, 2.5% H_2O[a]	≥99%	<5%	≥99%	≥99%	≥99%	92%	≥99%
95% TFA, 5% TIS, DTT[a,b]	≥99%	<5%	98%	≥99%	≥99%	≥99%	≥99%
20% piperidine/DMF[c]	≥99%	≥99%	≥99%	94%	84%	93%	85%

Supplementary Table 2. Drugs tested on human T cells using peptide **36**.

**drugs**	**concentration**	**incubation time at 37°C**
avasimibe	25 μM	24 h
AZD5363	1 μM	4 h
clopidogrel	20 μM	24 h
lactacystin	1 μM	4 h
lenalidomide	1 μM	24 h
mitomycin c	1 μM	4 h
rapamycin	0.3 μM	4 h
tacrolimus	12.5 μM	24 h
temozolomide	1 μM	4 h

3. Supplementary Movie Legends

Supplementary Movie 1. Time-lapse fluorescence confocal microscopy images of *E. coli* after incubation with peptide **22** (25 μM, magenta). Peptide **22** was added to the cells, and fluorescence images were immediately recorded. Excitation laser: 452 nm. The movie was compressed (jpeg) at 10 fps.

Supplementary Movie 2. Time-lapse fluorescence confocal microscopy images of *E. coli* after incubation with peptide **23** (1 μM, yellow). Peptide **23** was added to the cells, and fluorescence images were immediately recorded. Excitation laser: 554 nm. The movie was compressed (jpeg) at 10 fps.

Supplementary Movie 3. Time-lapse fluorescence confocal microscopy images of *E. coli* after incubation with peptide **24** (1 μM, red). Peptide **24** was added to the cells, and fluorescence images were immediately recorded. Excitation laser: 650 nm. The movie was compressed (jpeg) at 10 fps.

4. Experimental Isotopic Patterns from MALDI-TOF-TOF Mass Spectra

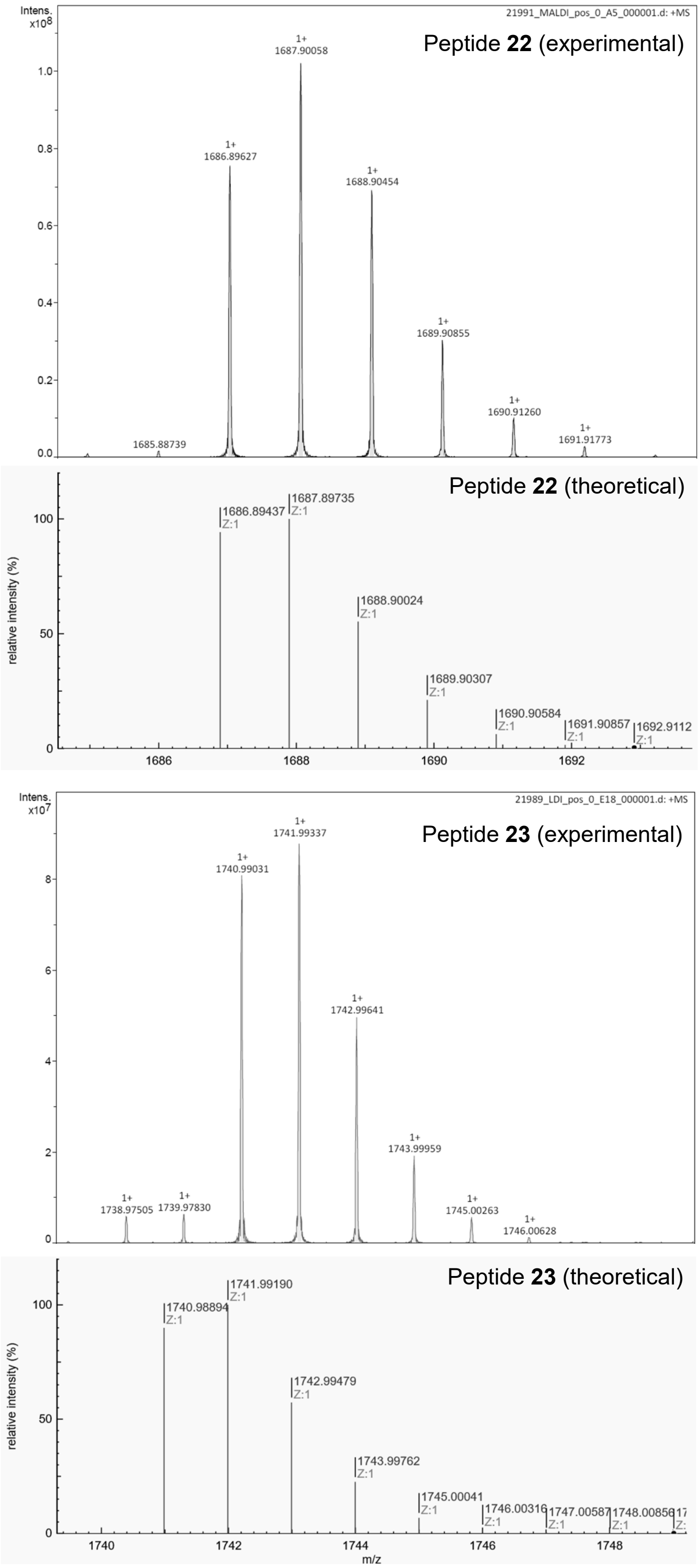

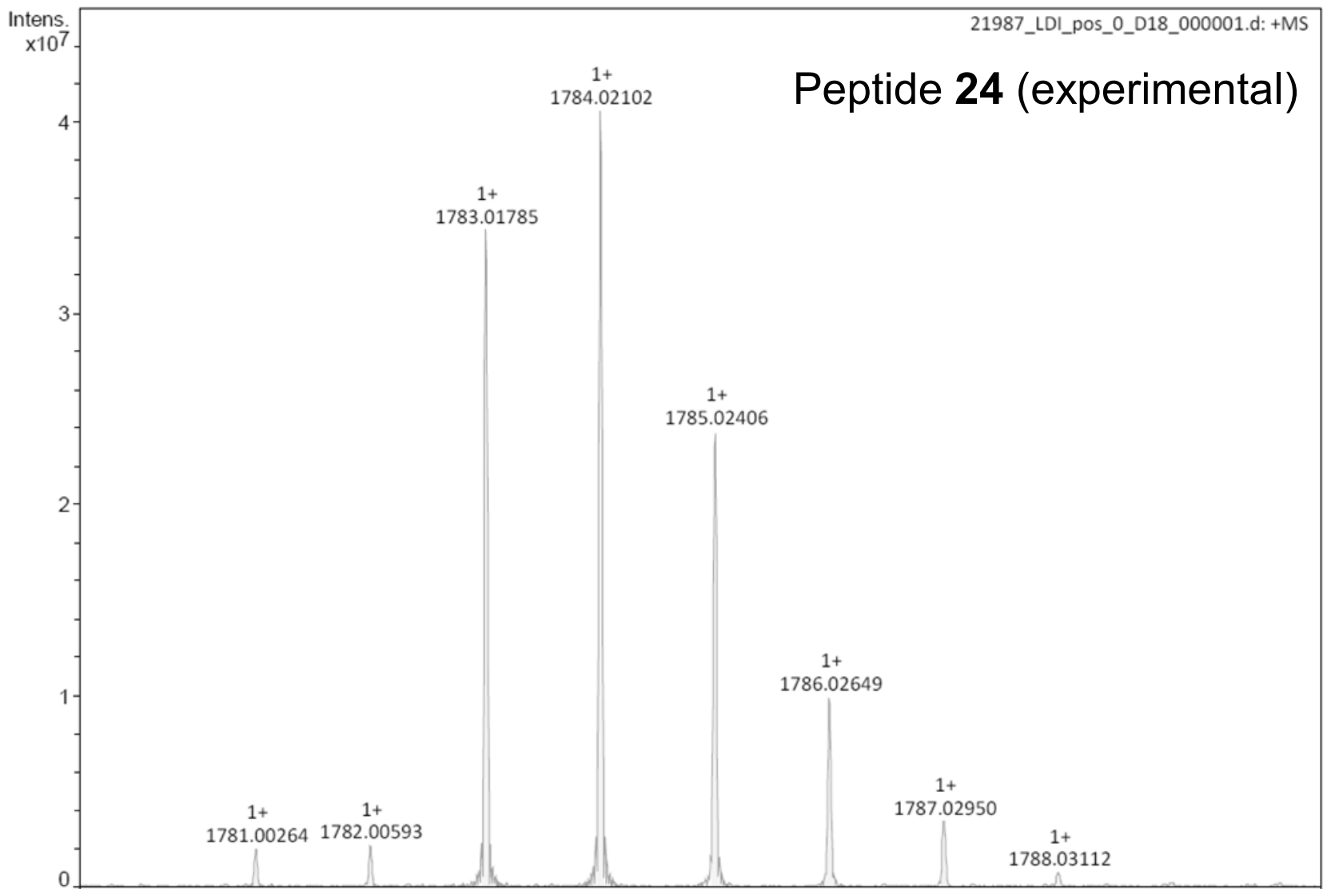
Intens.
x10^7
21987_LDI_pos_0_D18_000001.d: +MS
Peptide 24 (experimental)
1+
1781.00264
1+
1782.00593
1+
1783.01785
1+
1784.02102
1+
1785.02406
1+
1786.02649
1+
1787.02950
1+
1788.03112

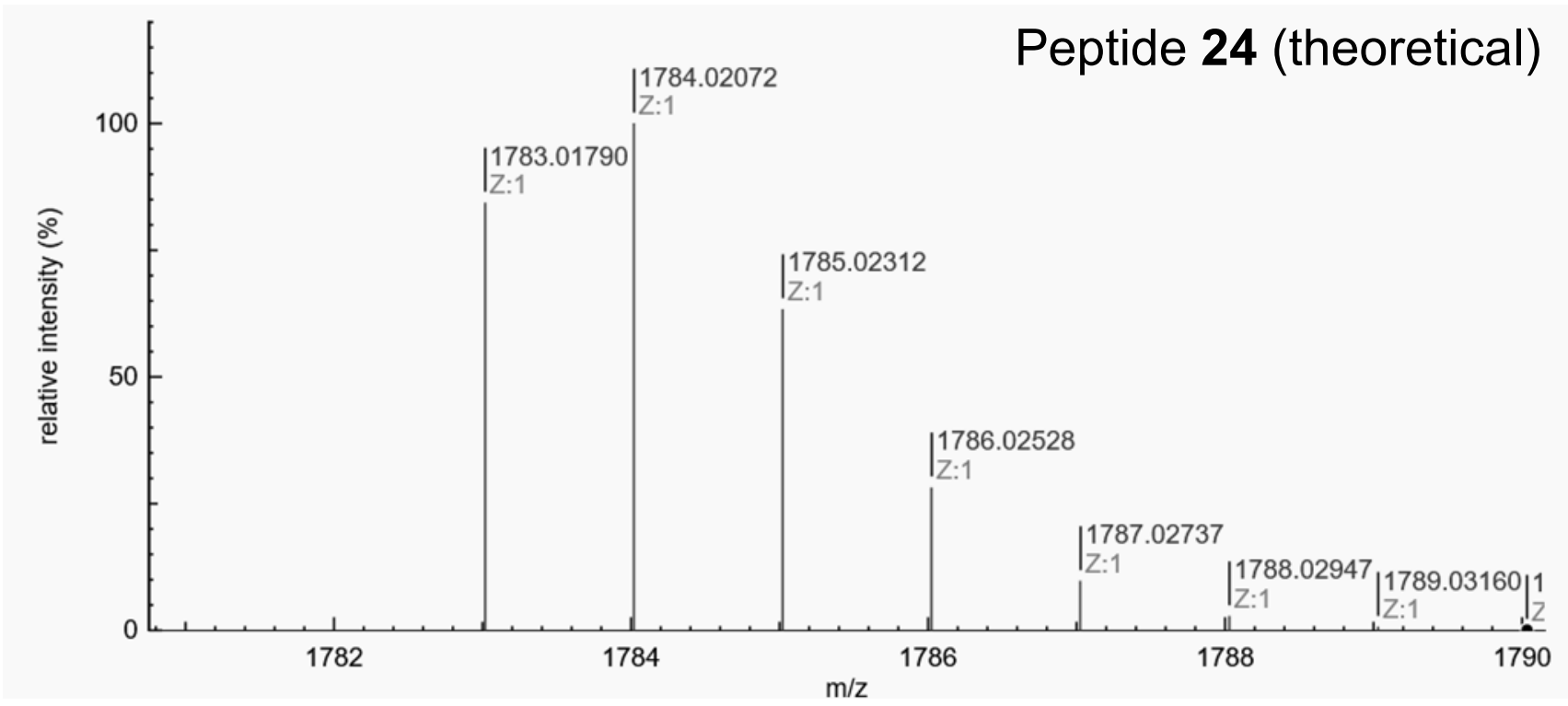
Peptide 24 (theoretical)
relative intensity (%)
1783.01790
Z:1
1784.02072
Z:1
1785.02312
Z:1
1786.02528
Z:1
1787.02737
Z:1
1788.02947
Z:1
1789.03160
Z:1
m/z

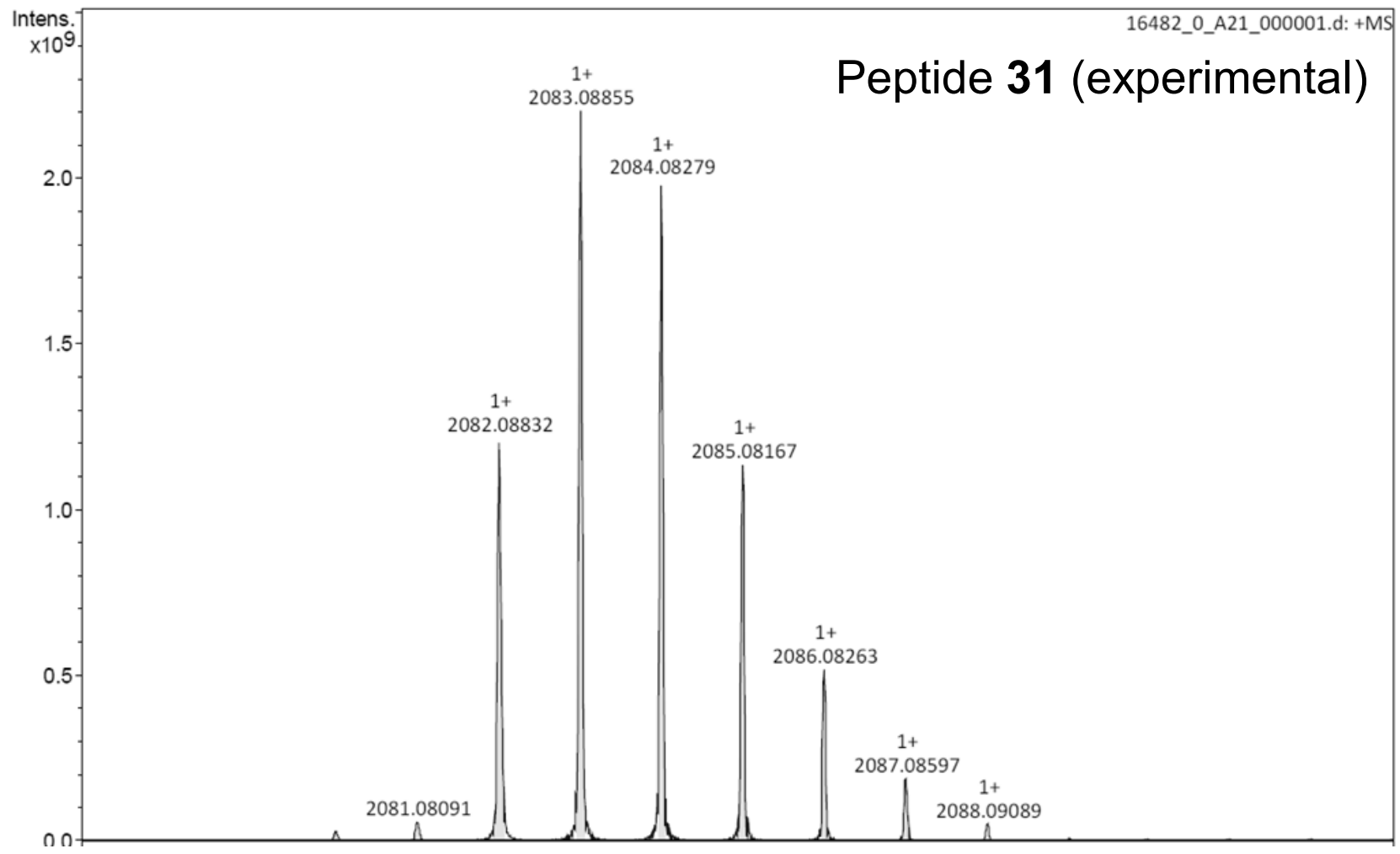
Intens.
x10^9
16482_0_A21_000001.d: +MS
Peptide 31 (experimental)
2081.08091
1+
2082.08832
1+
2083.08855
1+
2084.08279
1+
2085.08167
1+
2086.08263
1+
2087.08597
1+
2088.09089

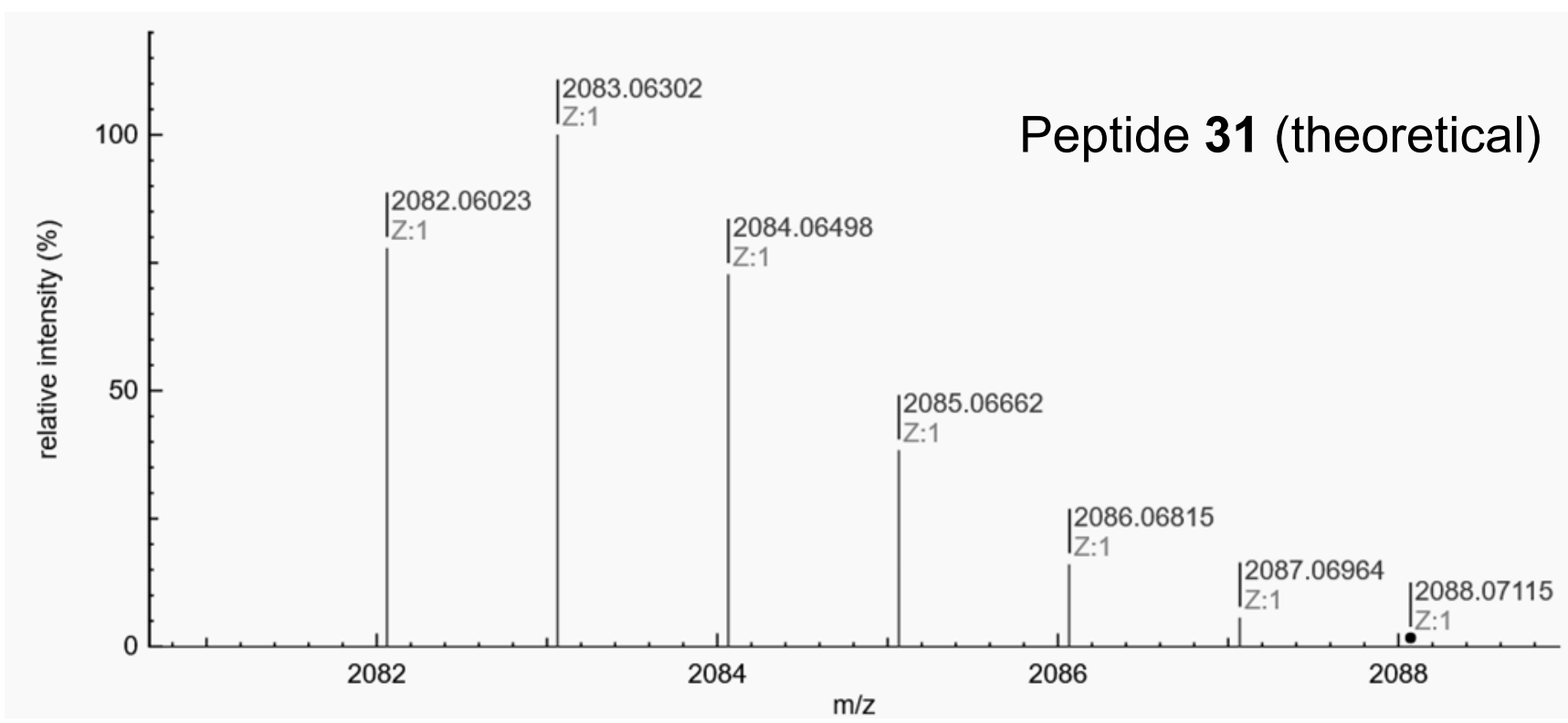
Peptide 31 (theoretical)
relative intensity (%)
2082.06023
Z:1
2083.06302
Z:1
2084.06498
Z:1
2085.06662
Z:1
2086.06815
Z:1
2087.06964
Z:1
2088.07115
Z:1
m/z

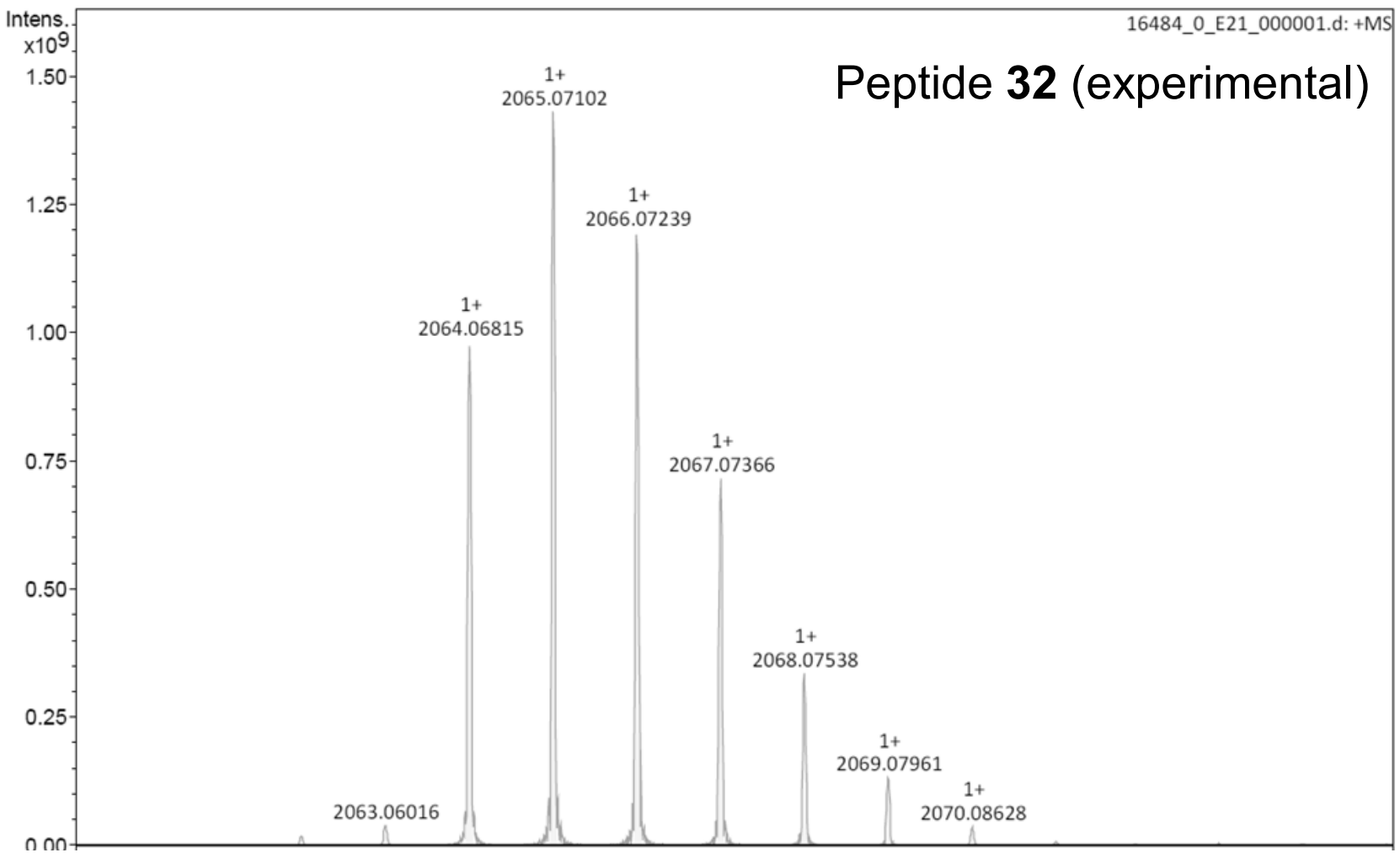
Intens.
x10^9
16484_0_E21_000001.d: +MS
Peptide 32 (experimental)
1+ 2065.07102
1+ 2066.07239
1+ 2064.06815
1+ 2067.07366
1+ 2068.07538
1+ 2069.07961
1+ 2070.08628
2063.06016

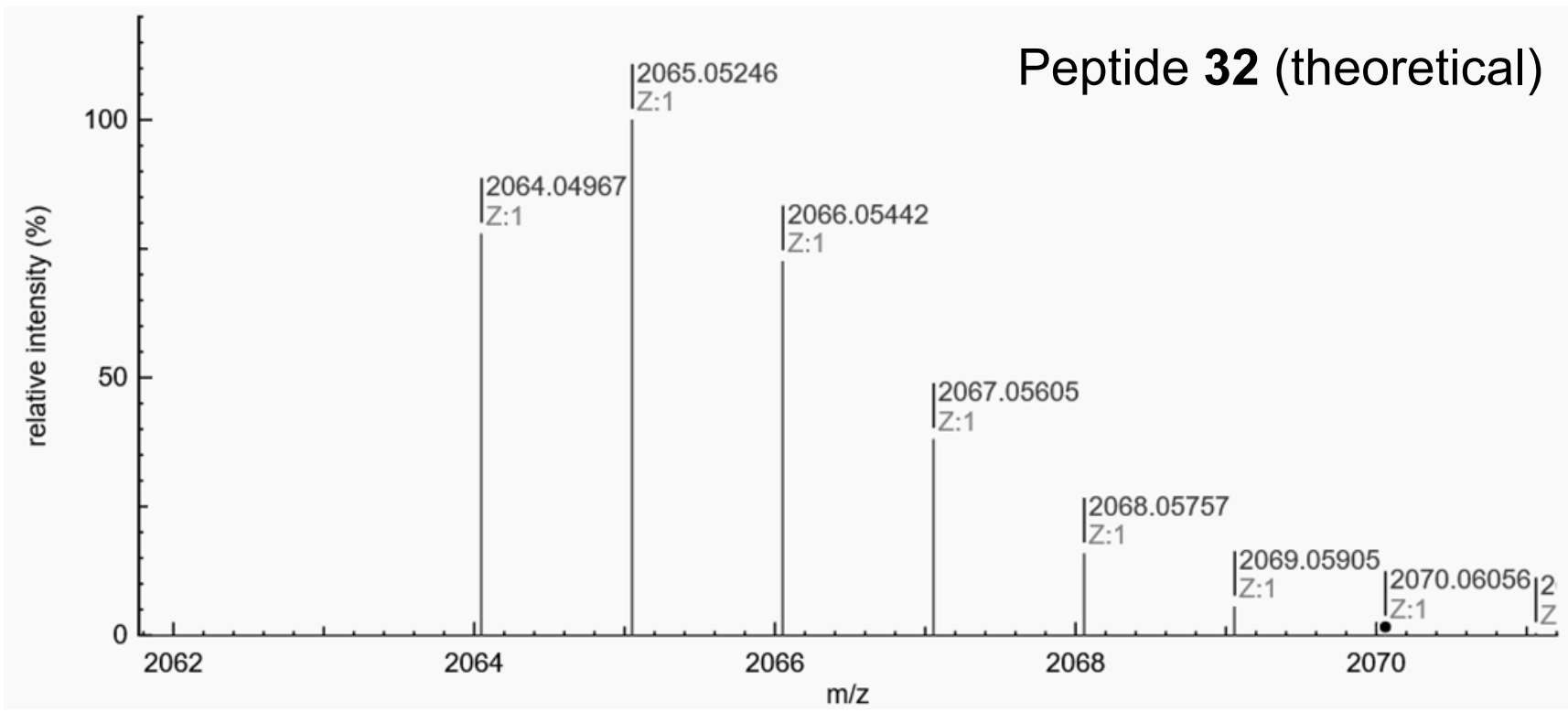
Peptide 32 (theoretical)
relative intensity (%)
2065.05246 Z:1
2064.04967 Z:1
2066.05442 Z:1
2067.05605 Z:1
2068.05757 Z:1
2069.05905 Z:1
2070.06056 Z:1
m/z

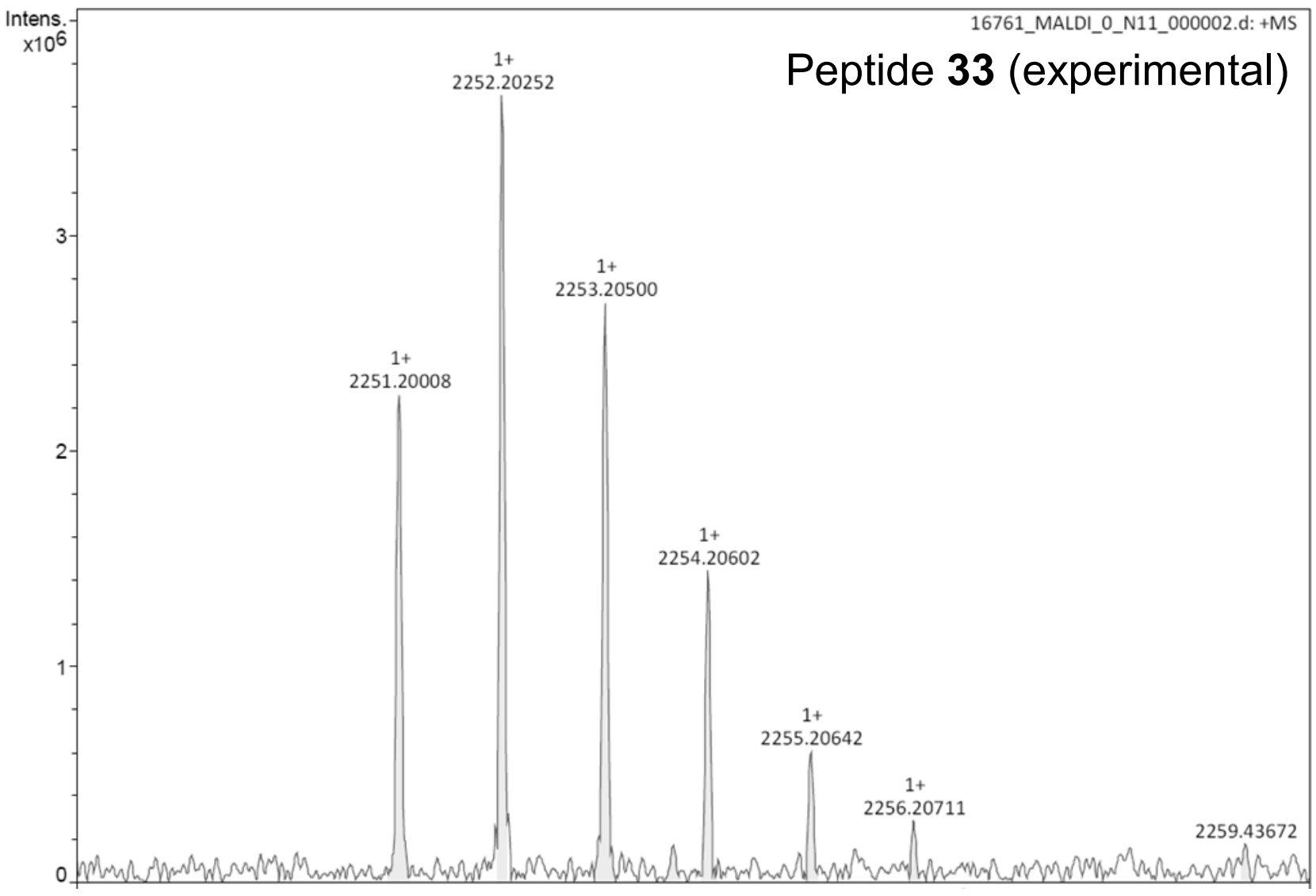
Intens.
x10^6
16761_MALDI_0_N11_000002.d: +MS
Peptide 33 (experimental)
1+ 2252.20252
1+ 2253.20500
1+ 2251.20008
1+ 2254.20602
1+ 2255.20642
1+ 2256.20711
2259.43672

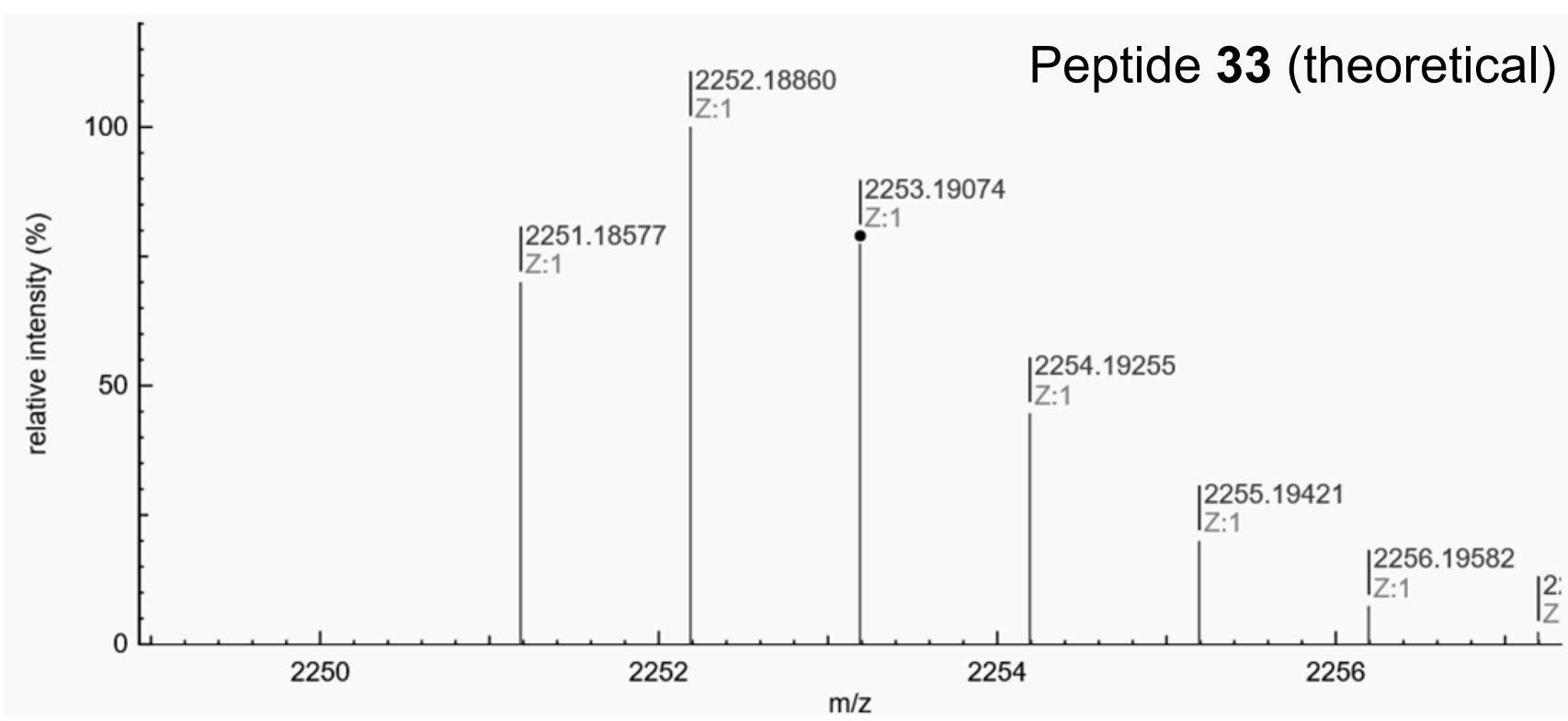
Peptide 33 (theoretical)
relative intensity (%)
2252.18860 Z:1
2253.19074 Z:1
2251.18577 Z:1
2254.19255 Z:1
2255.19421 Z:1
2256.19582 Z:1
m/z

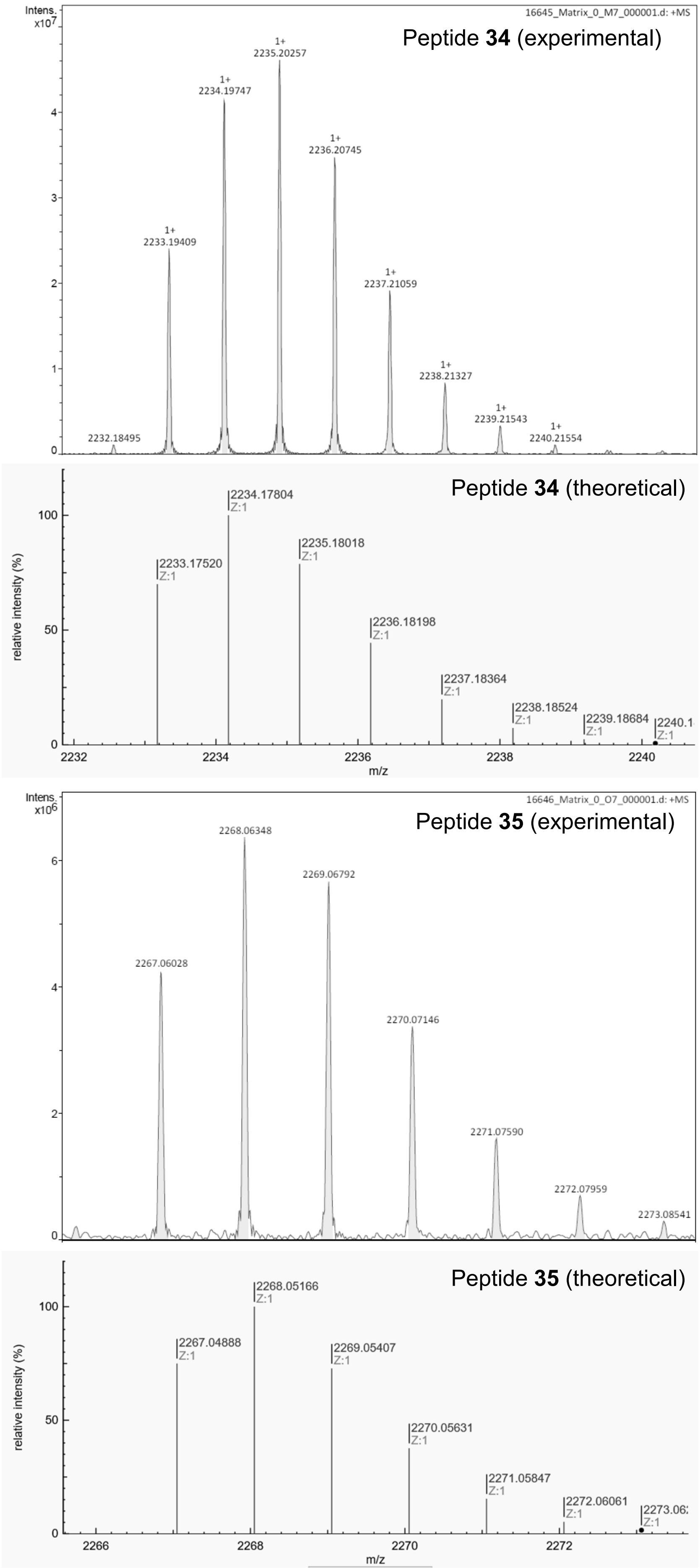
Peptide 34 (experimental)
16645_Matrix_0_M7_000001.d: +MS
Intens. x10^7
2232.18495
1+ 2233.19409
1+ 2234.19747
1+ 2235.20257
1+ 2236.20745
1+ 2237.21059
1+ 2238.21327
1+ 2239.21543
1+ 2240.21554
Peptide 34 (theoretical)
relative intensity (%)
2233.17520 Z:1
2234.17804 Z:1
2235.18018 Z:1
2236.18198 Z:1
2237.18364 Z:1
2238.18524 Z:1
2239.18684 Z:1
m/z
Peptide 35 (experimental)
16646_Matrix_0_O7_000001.d: +MS
Intens. x10^6
2267.06028
2268.06348
2269.06792
2270.07146
2271.07590
2272.07959
2273.08541
Peptide 35 (theoretical)
relative intensity (%)
2267.04888 Z:1
2268.05166 Z:1
2269.05407 Z:1
2270.05631 Z:1
2271.05847 Z:1
2272.06061 Z:1
m/z

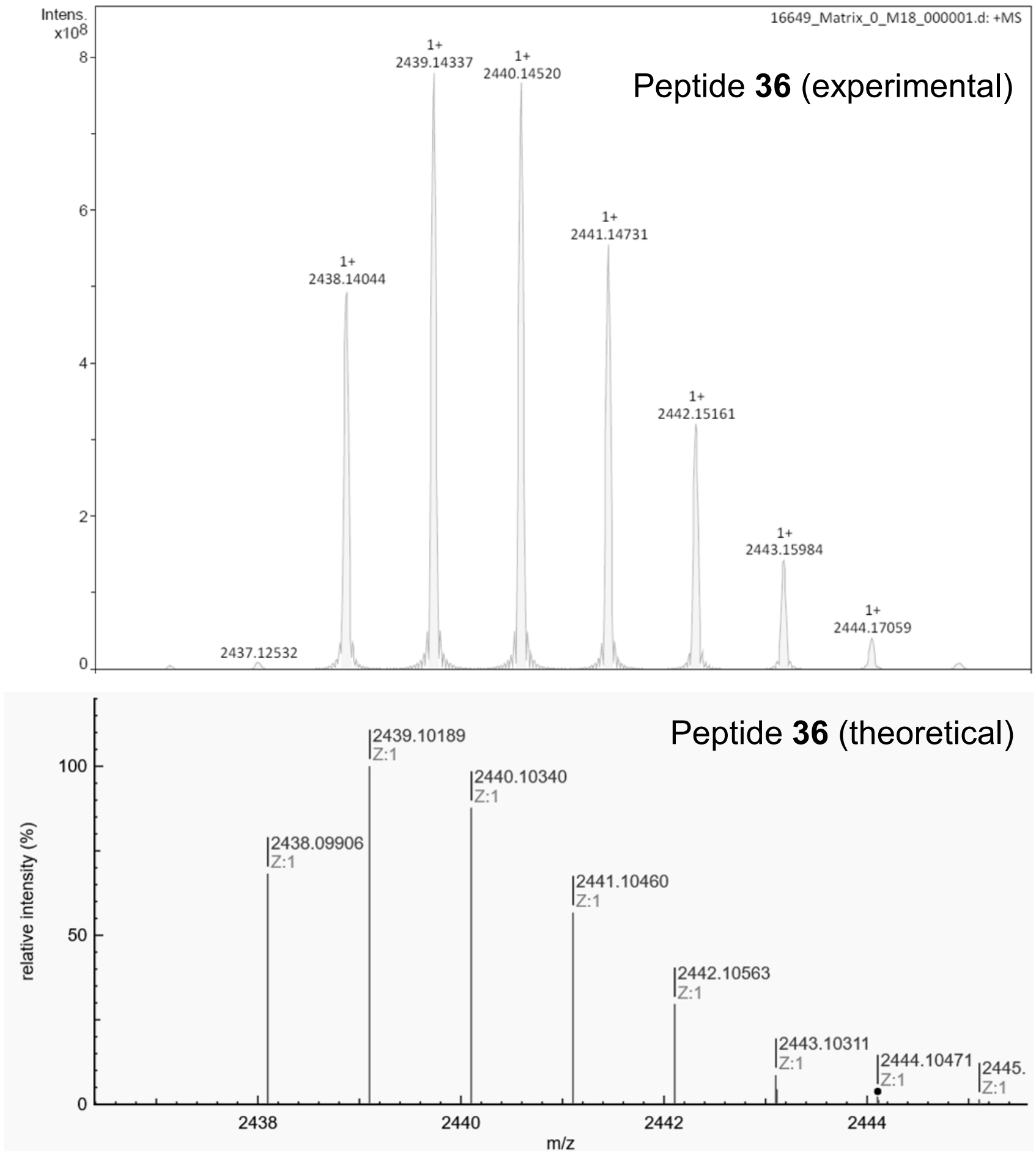

Intens.
x10^8
16649_Matrix_0_M18_000001.d: +MS
Peptide 36 (experimental)
1+
2439.14337
1+
2440.14520
1+
2441.14731
1+
2438.14044
1+
2442.15161
1+
2443.15984
1+
2444.17059
2437.12532
Peptide 36 (theoretical)
2439.10189
Z:1
2440.10340
Z:1
2438.09906
Z:1
2441.10460
Z:1
2442.10563
Z:1
2443.10311
Z:1
2444.10471
Z:1
2445.
Z:1
relative intensity (%)
m/z

5. NMR Spectra

Compound 1 (MeOD)

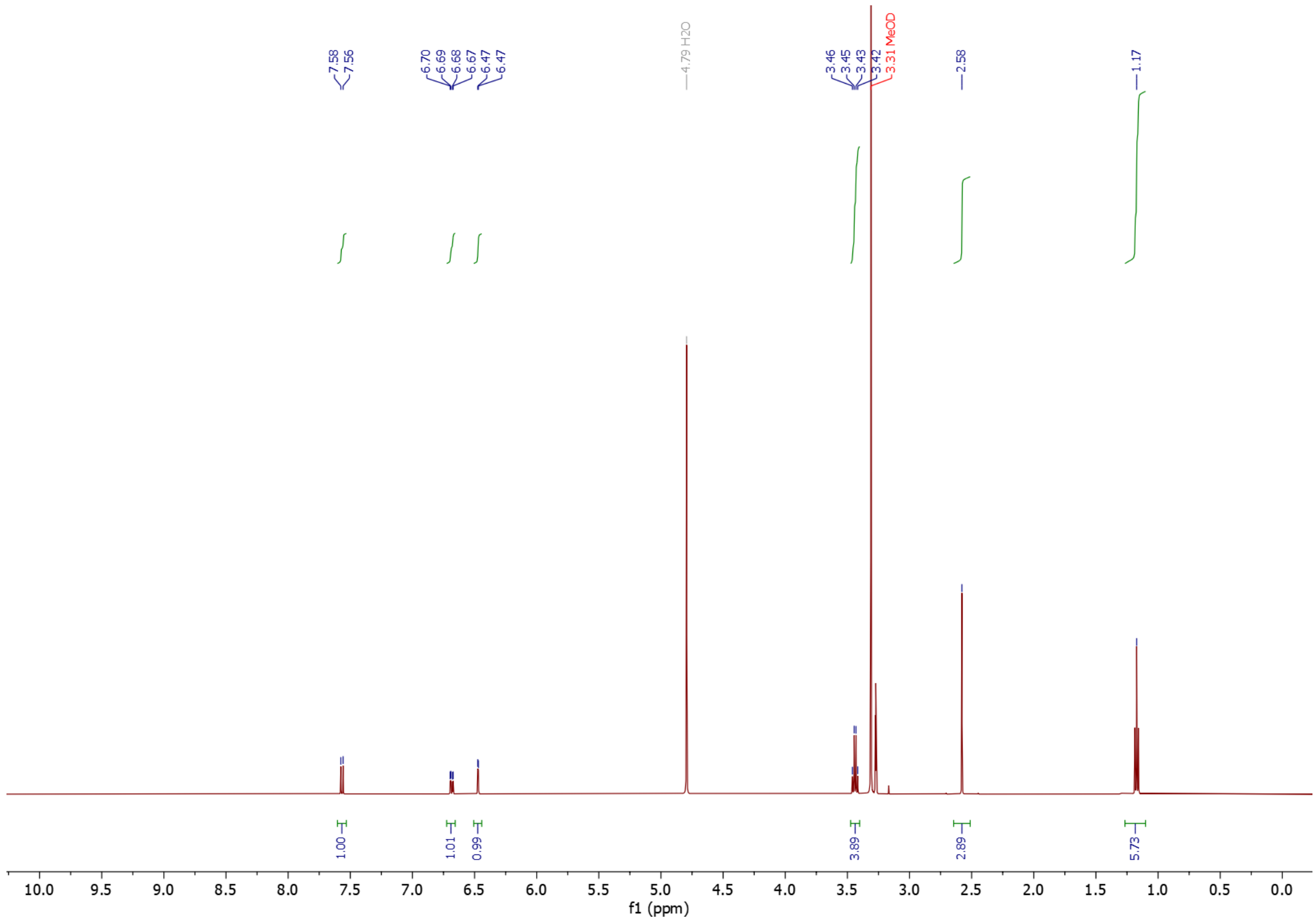

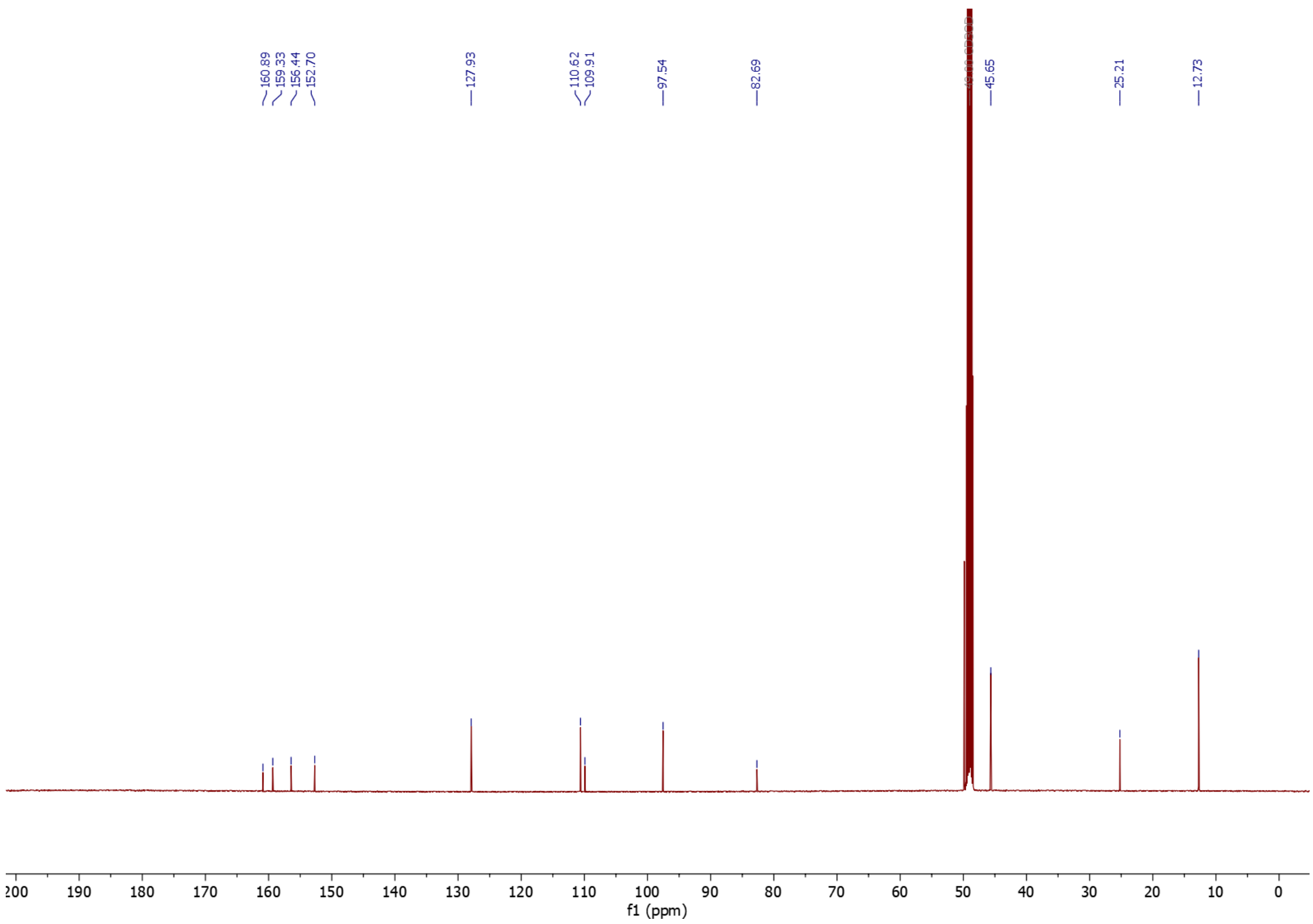

Compound 3 (DMSO-d_6)

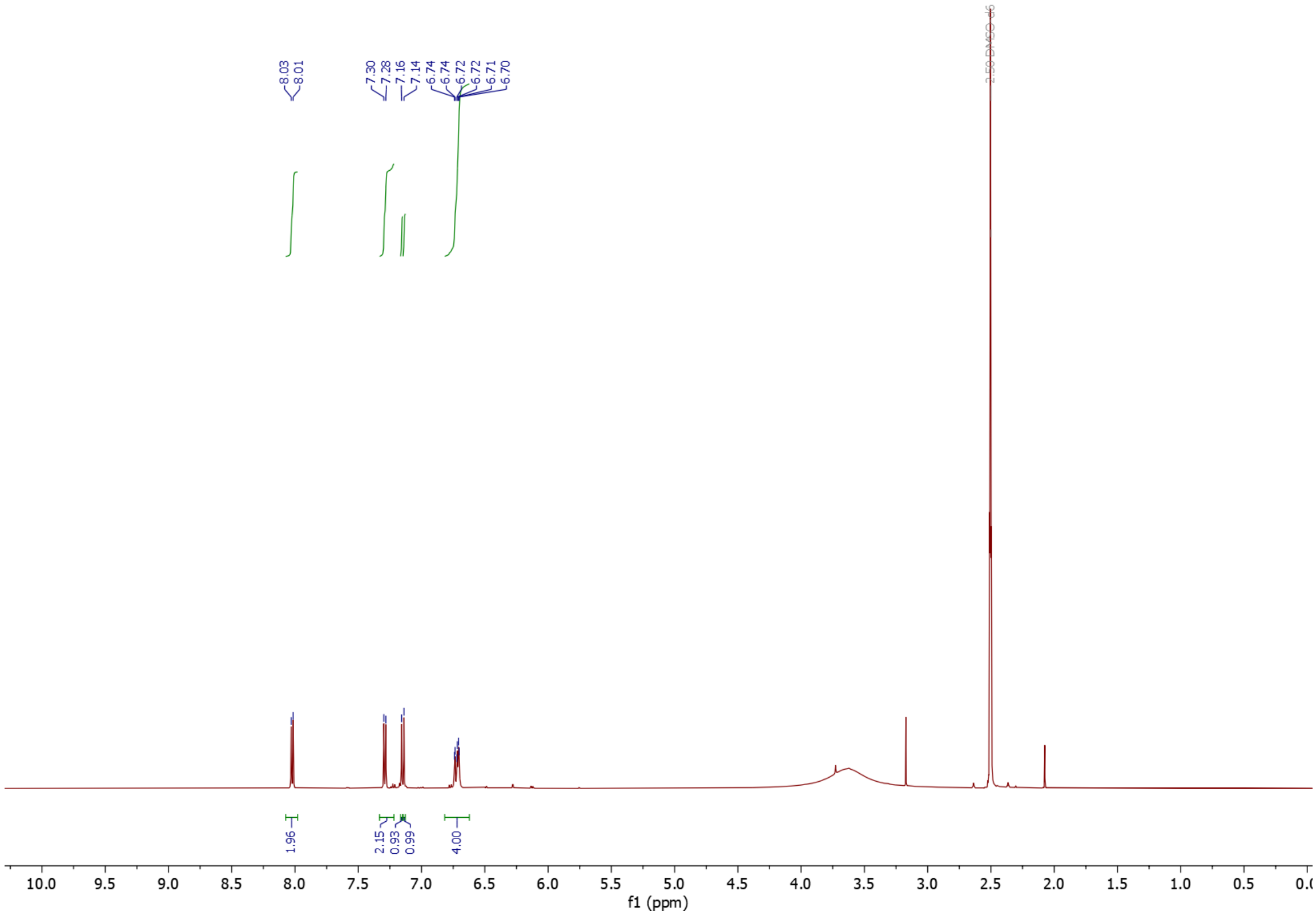

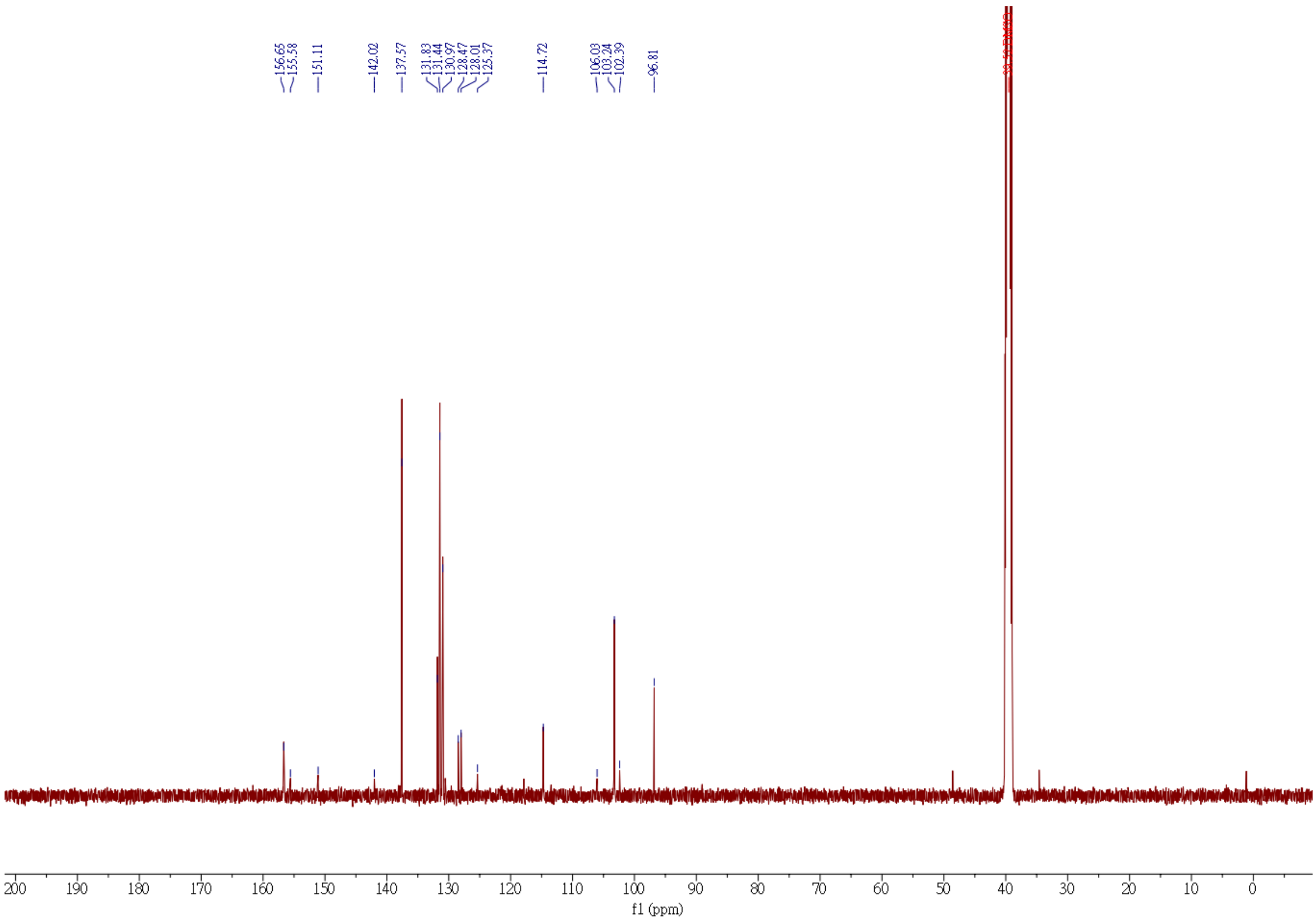

4,4'-Methylenebis(3-bromo-*N*,*N*-dimethylaniline) ($CDCl_3$)

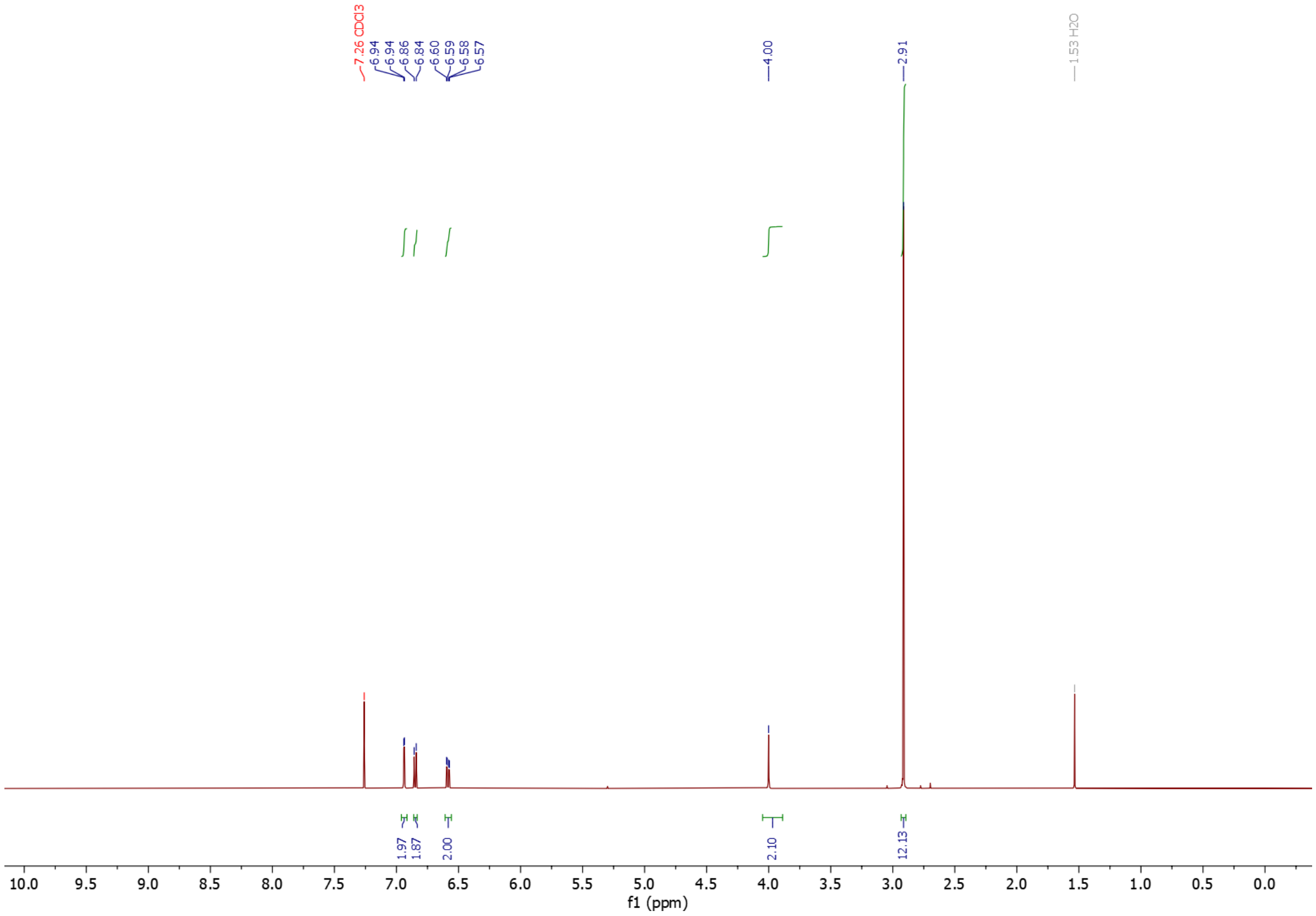

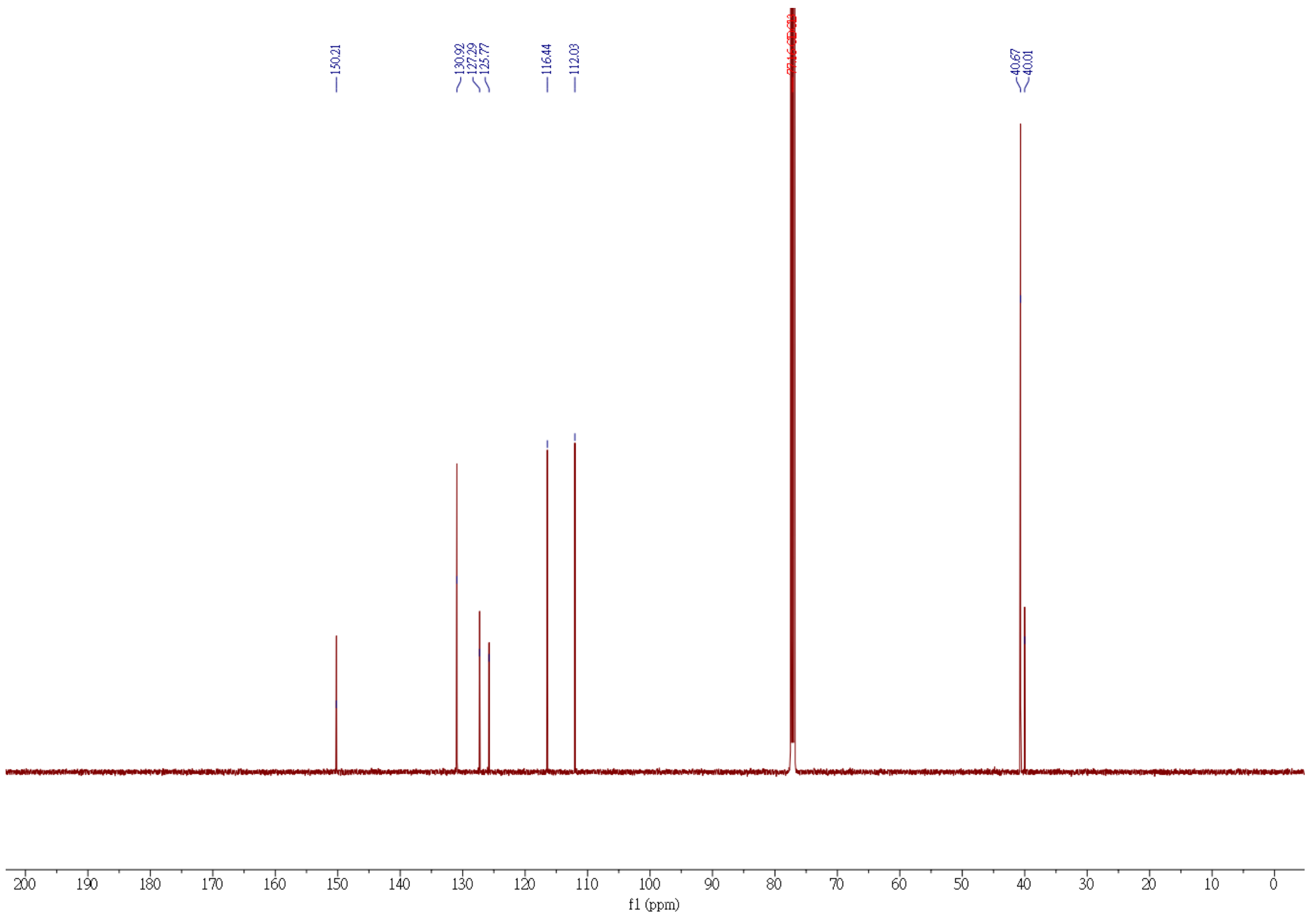

Compound 5 ($CDCl_3$)

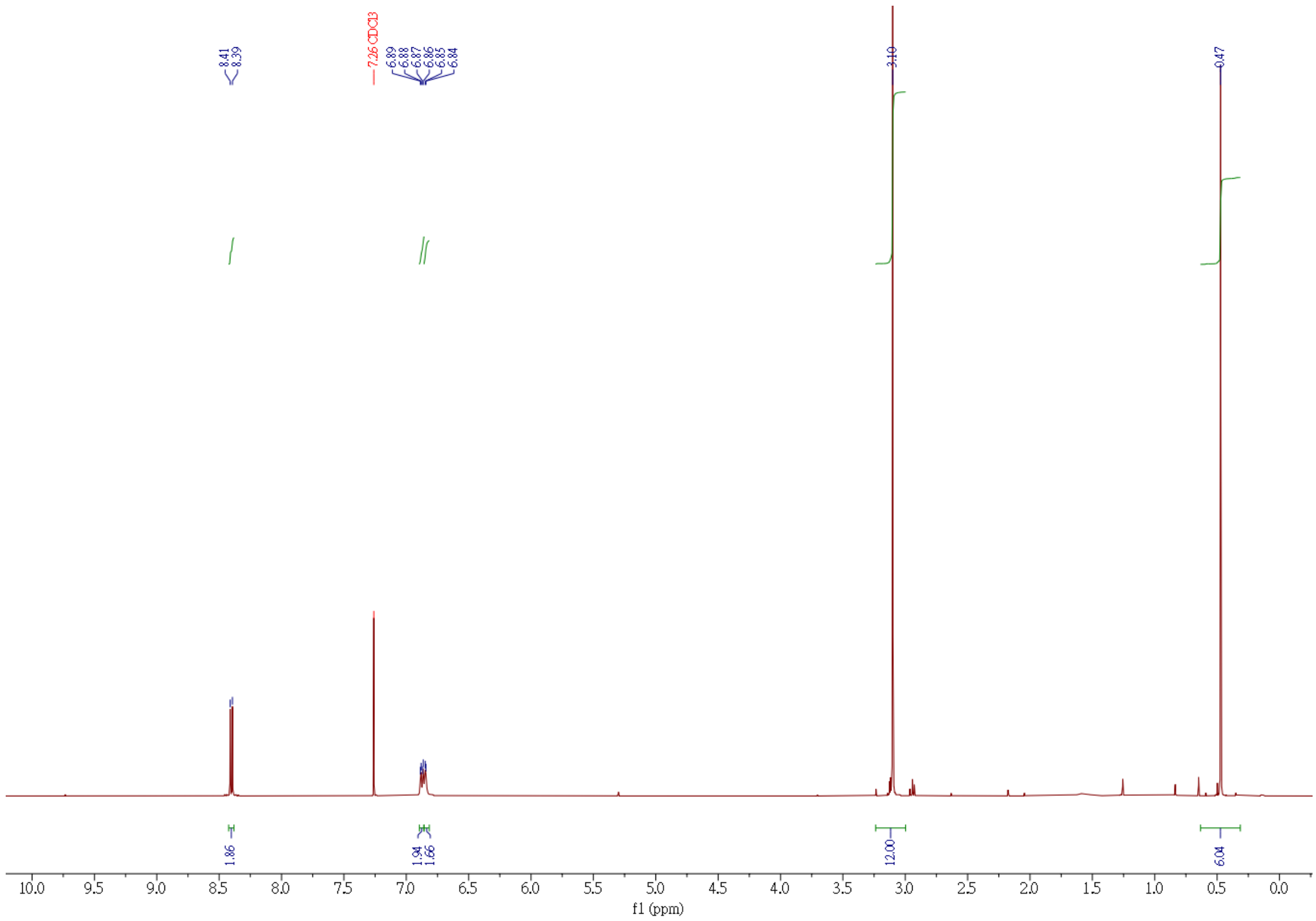

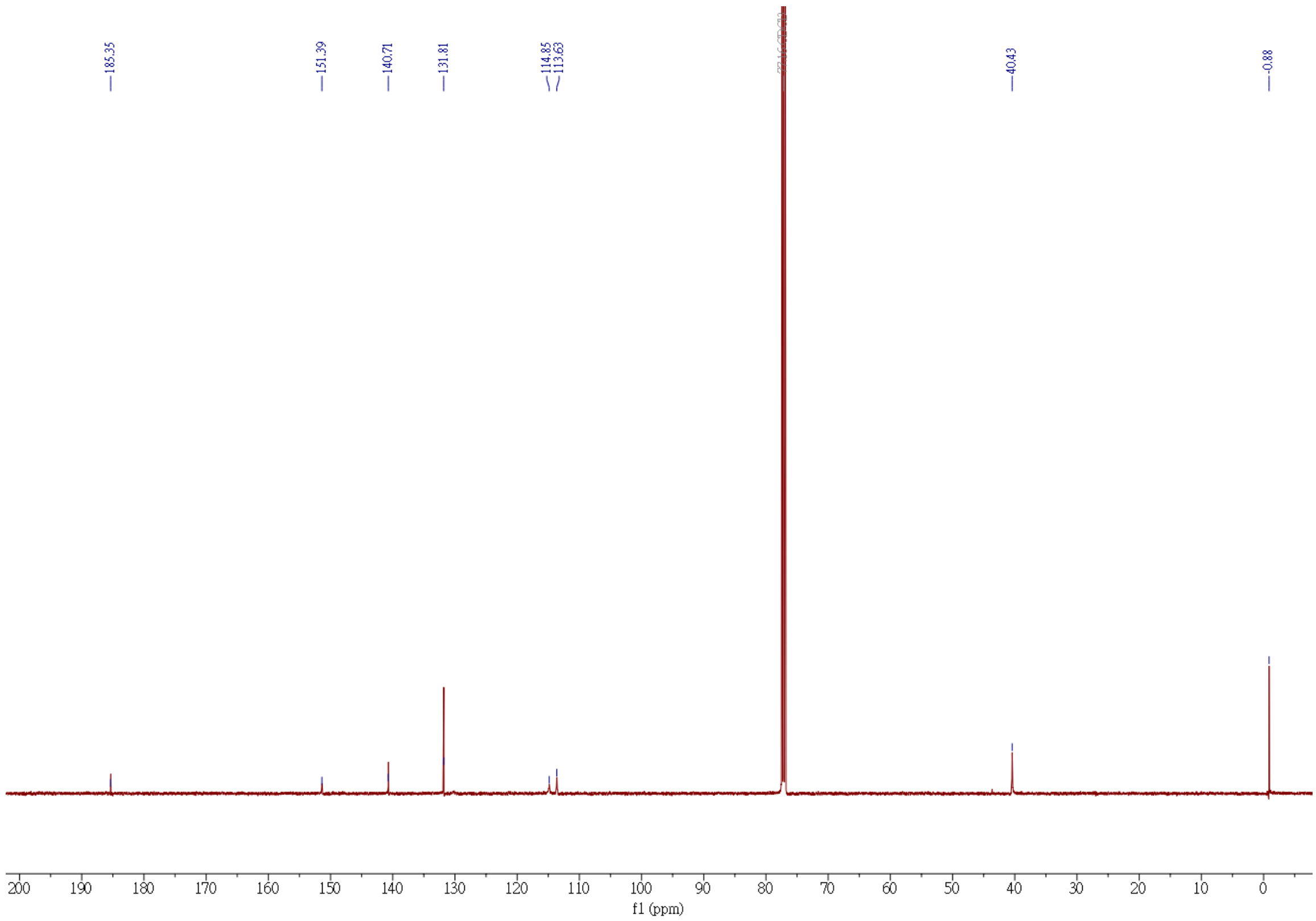

Compound 6 (CD_3OD)

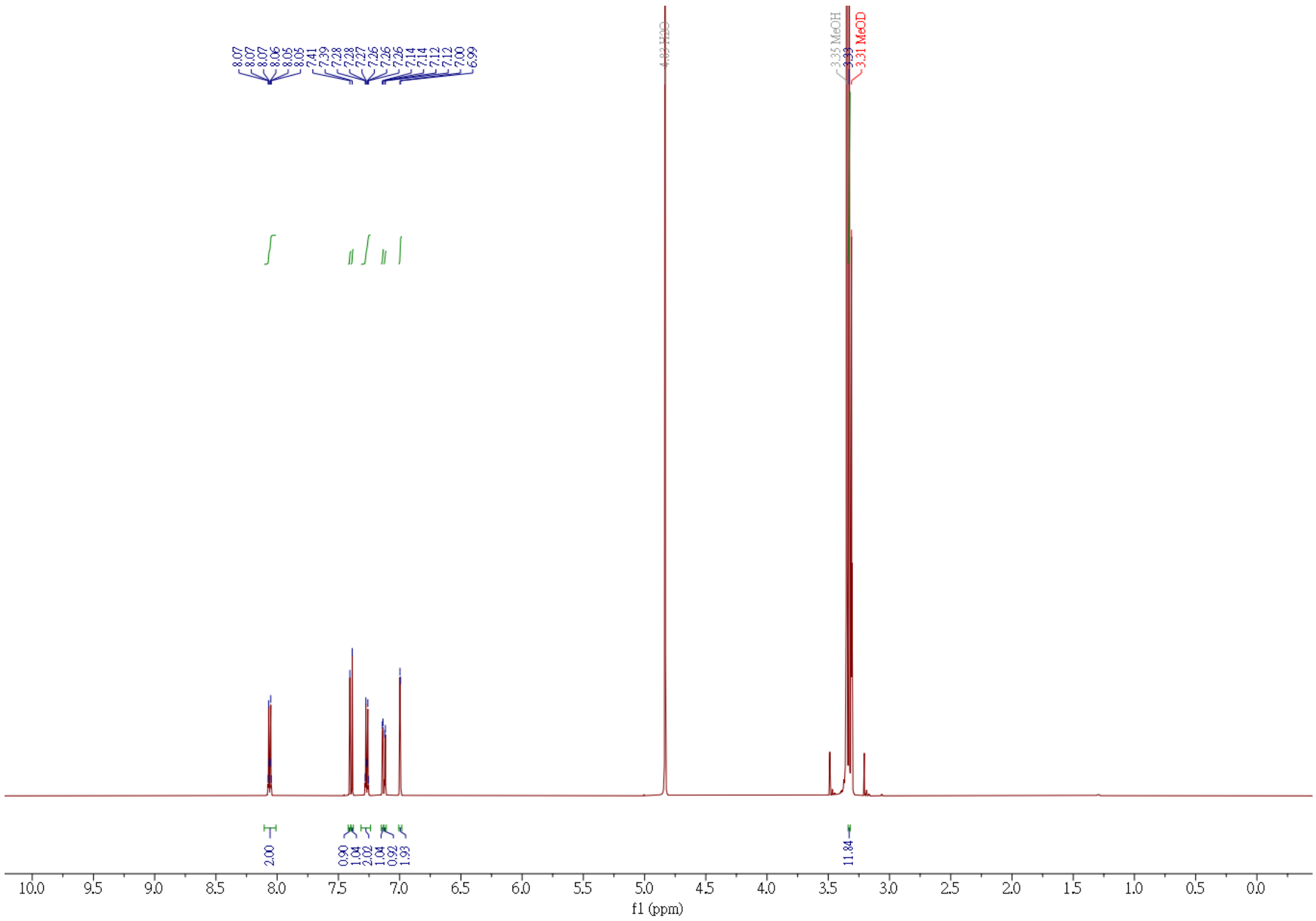

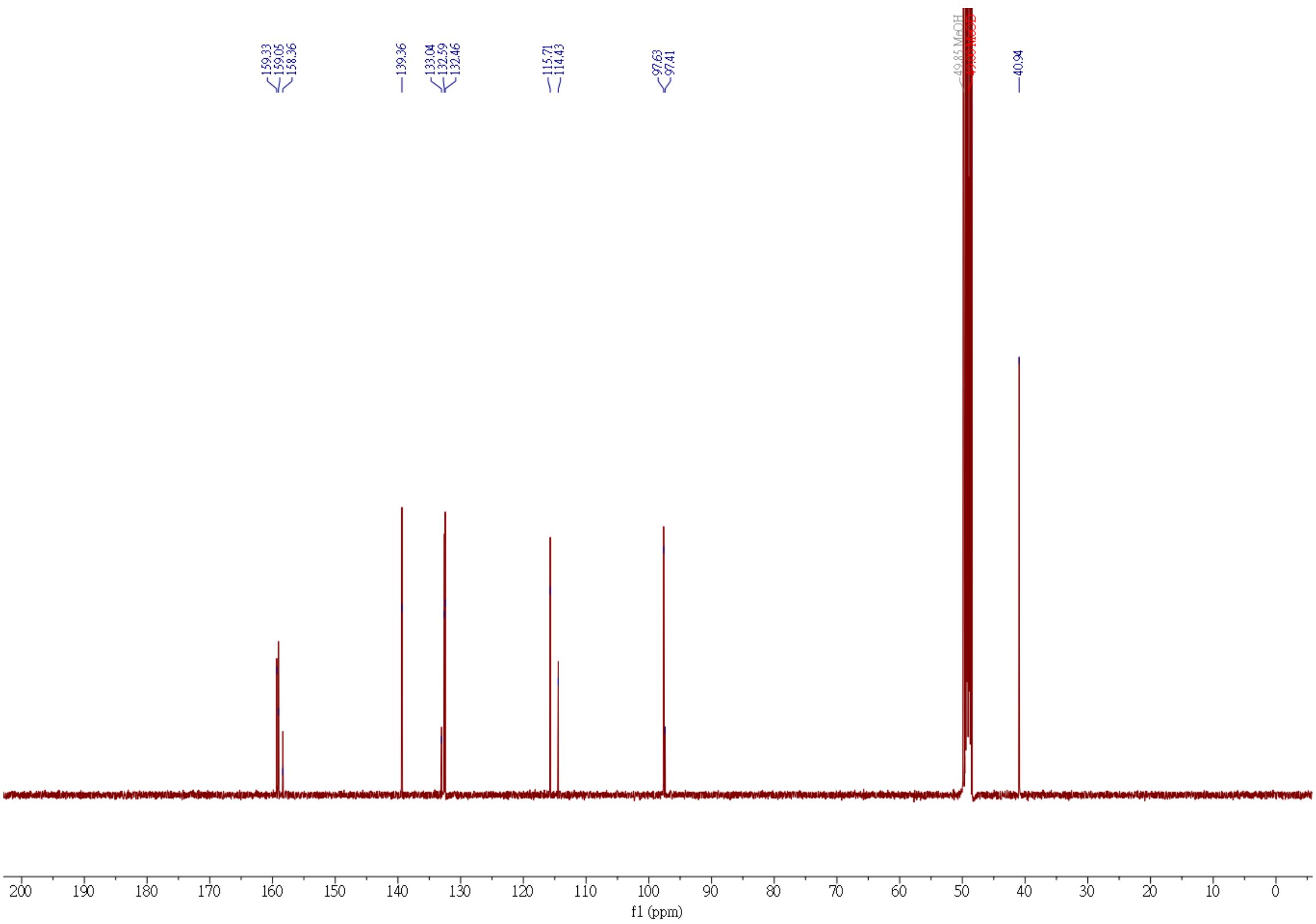

Compound 7 ($CDCl_3$)

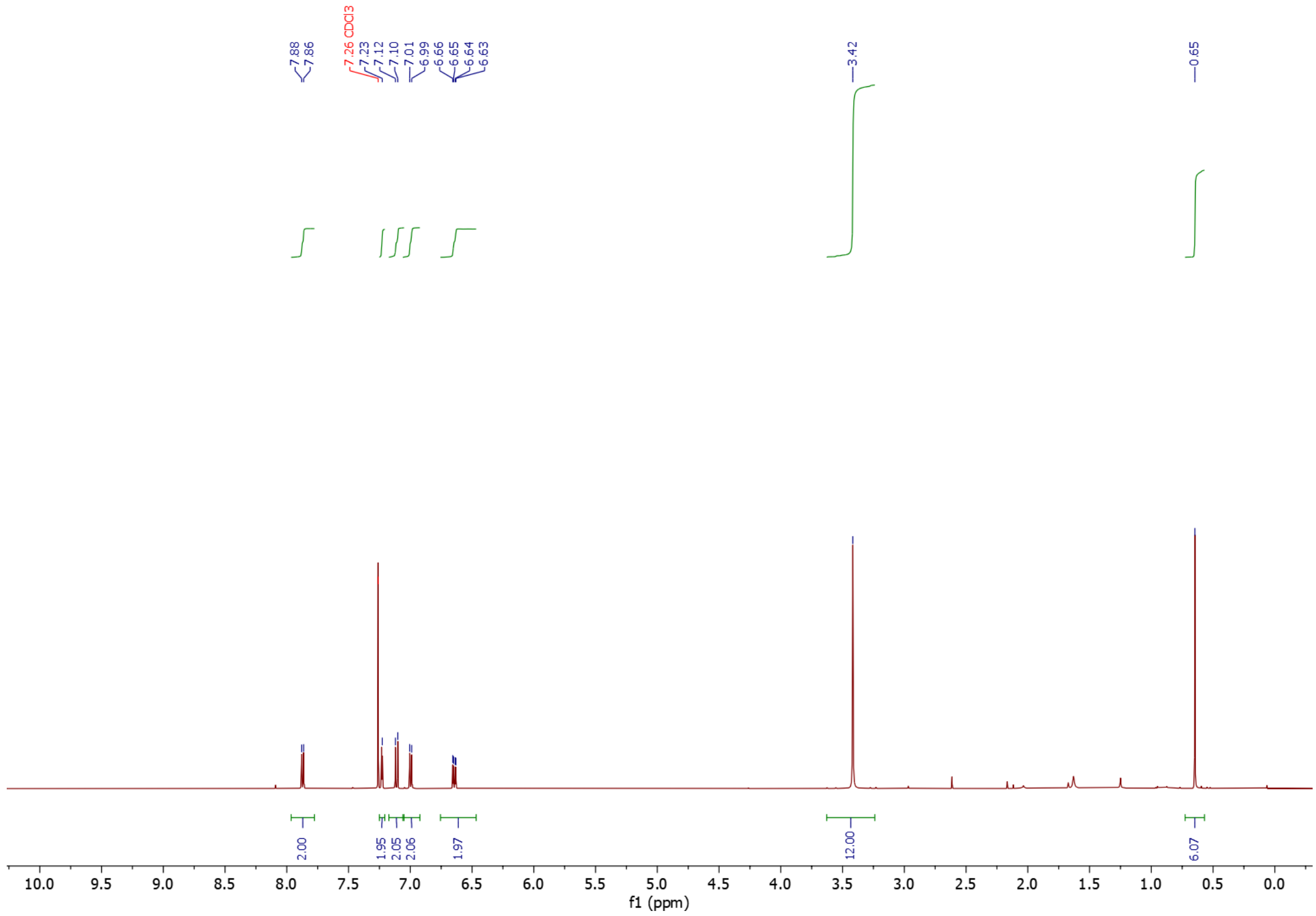

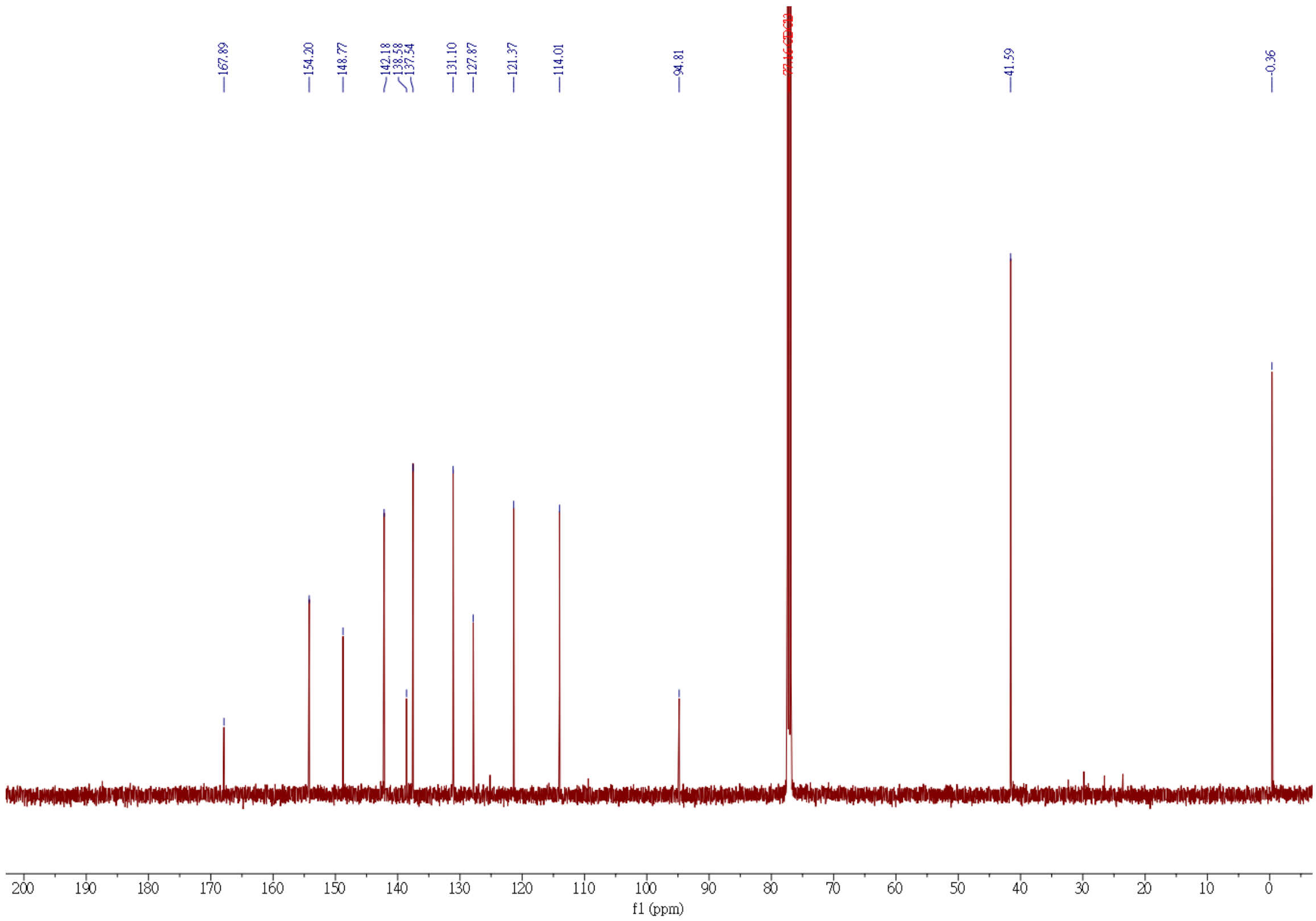

Compound 8 ($CDCl_3$)

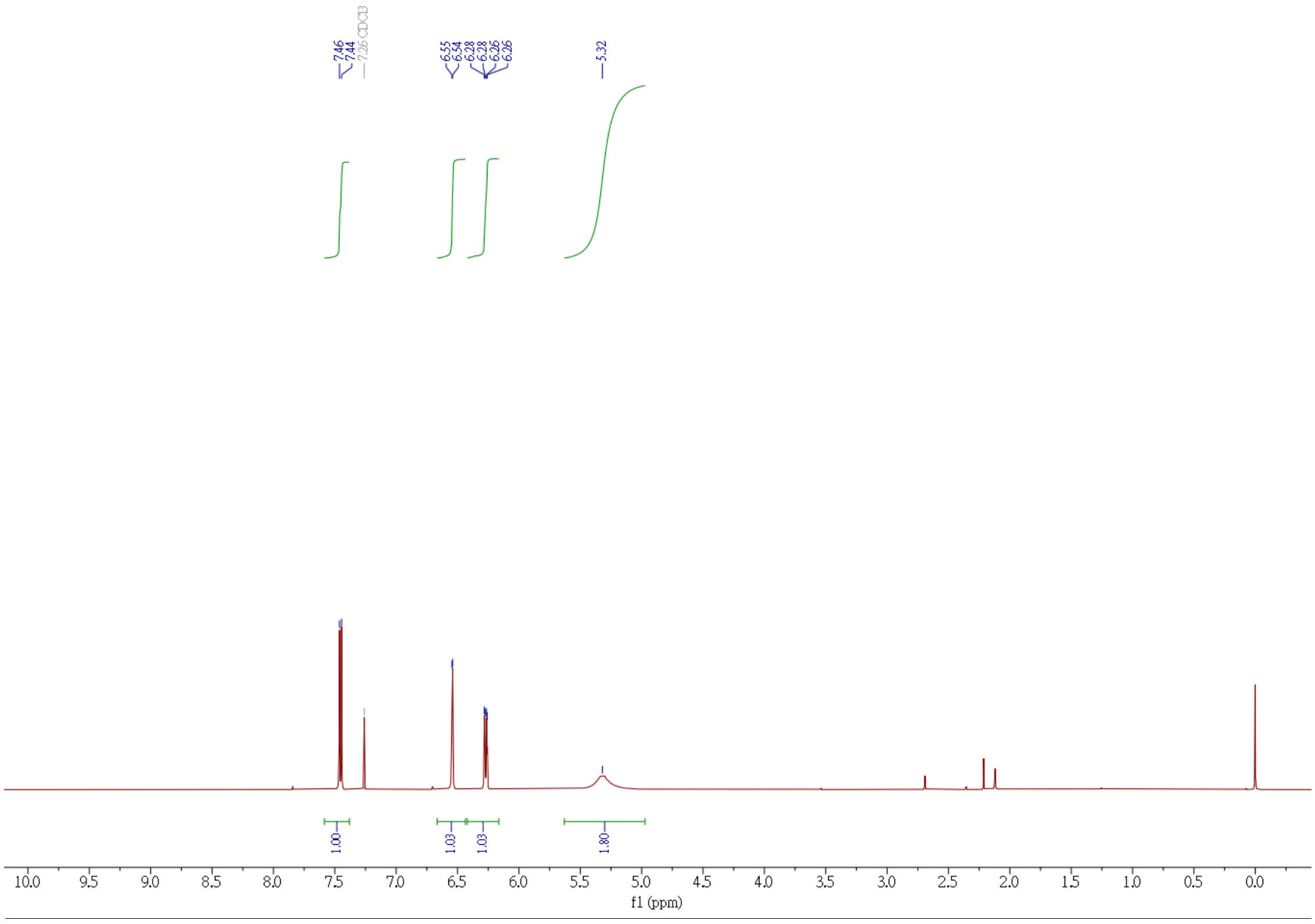

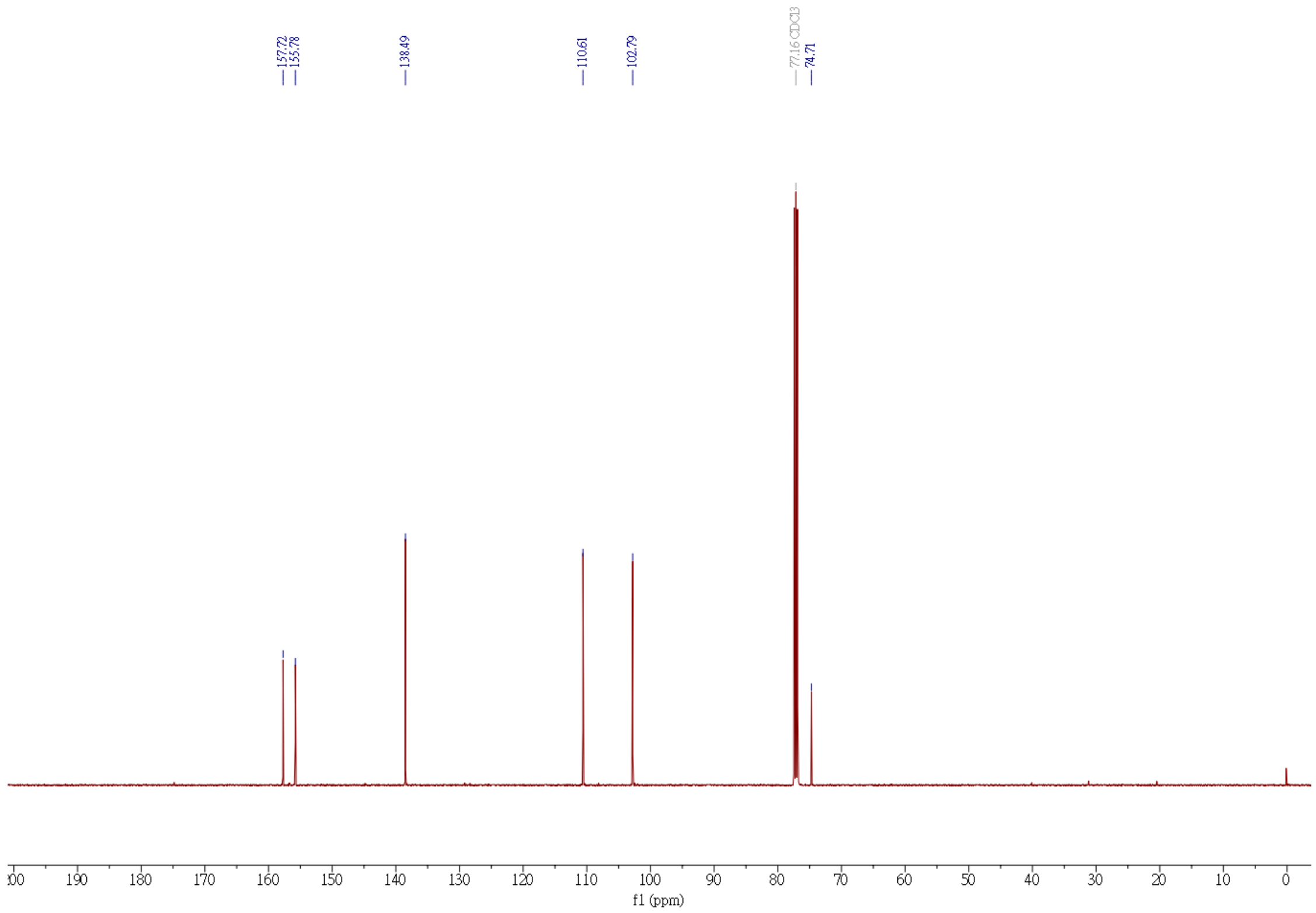

Compound 9 ($CDCl_3$)

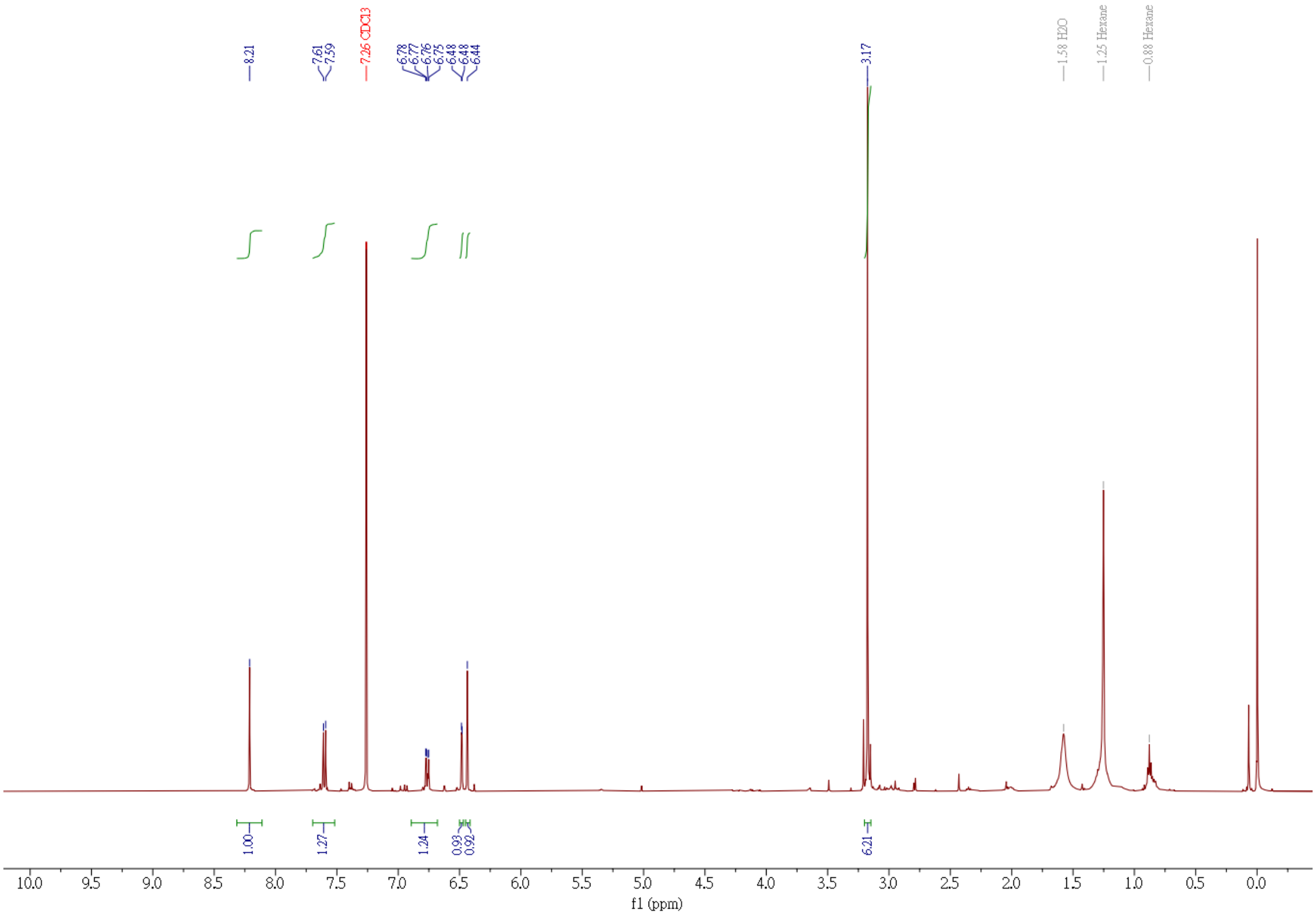

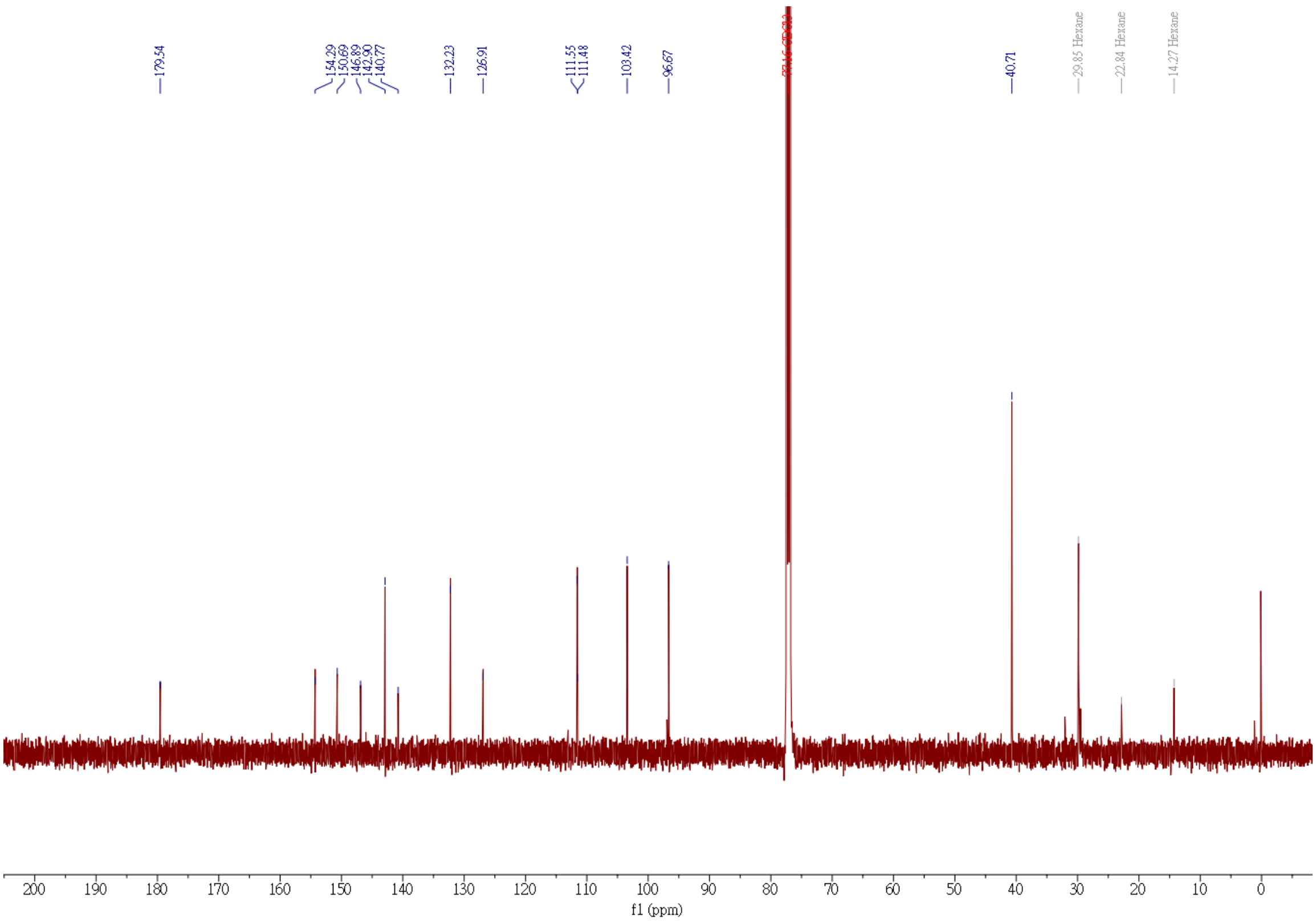

Compound 10 ($CDCl_3$)

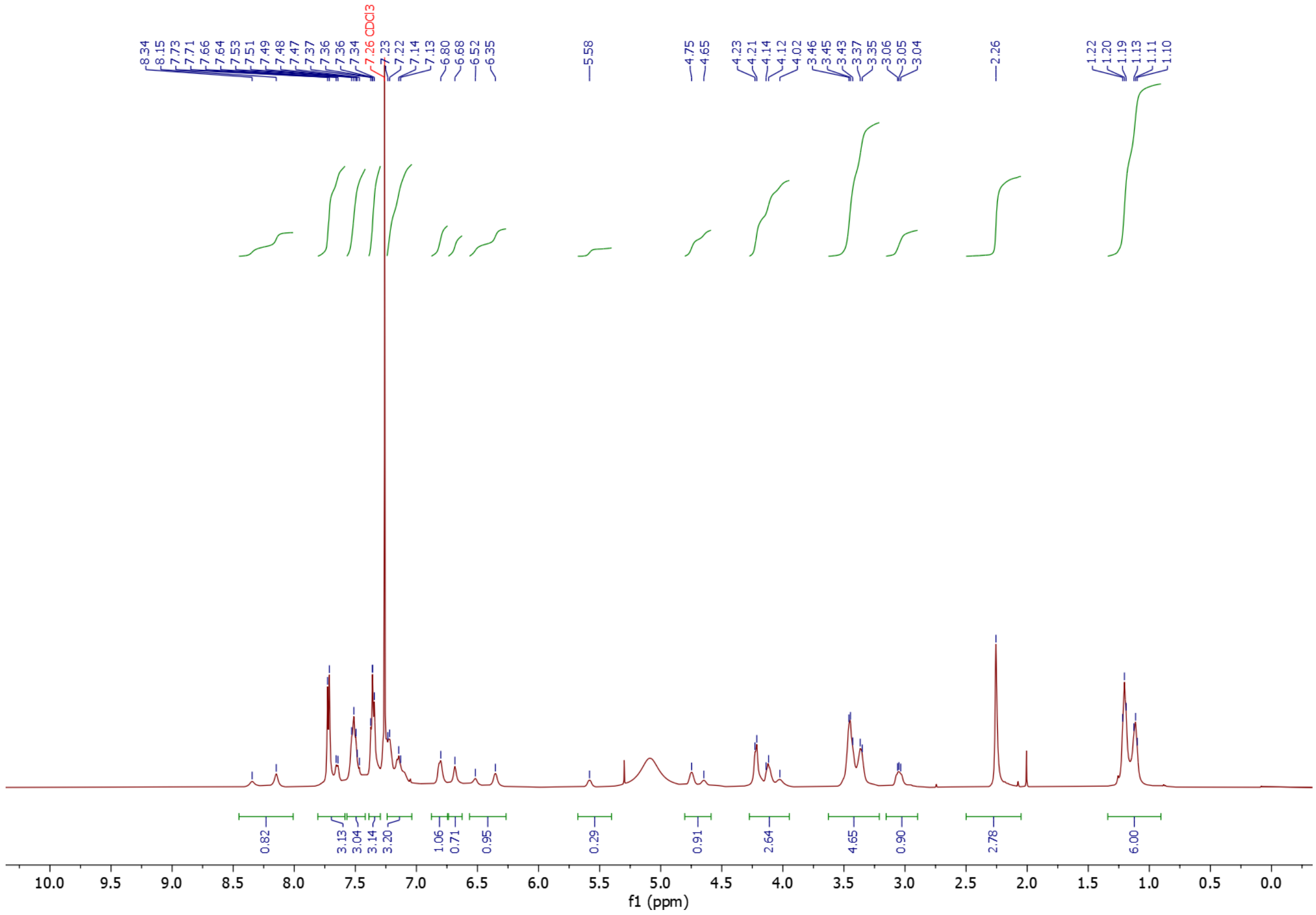

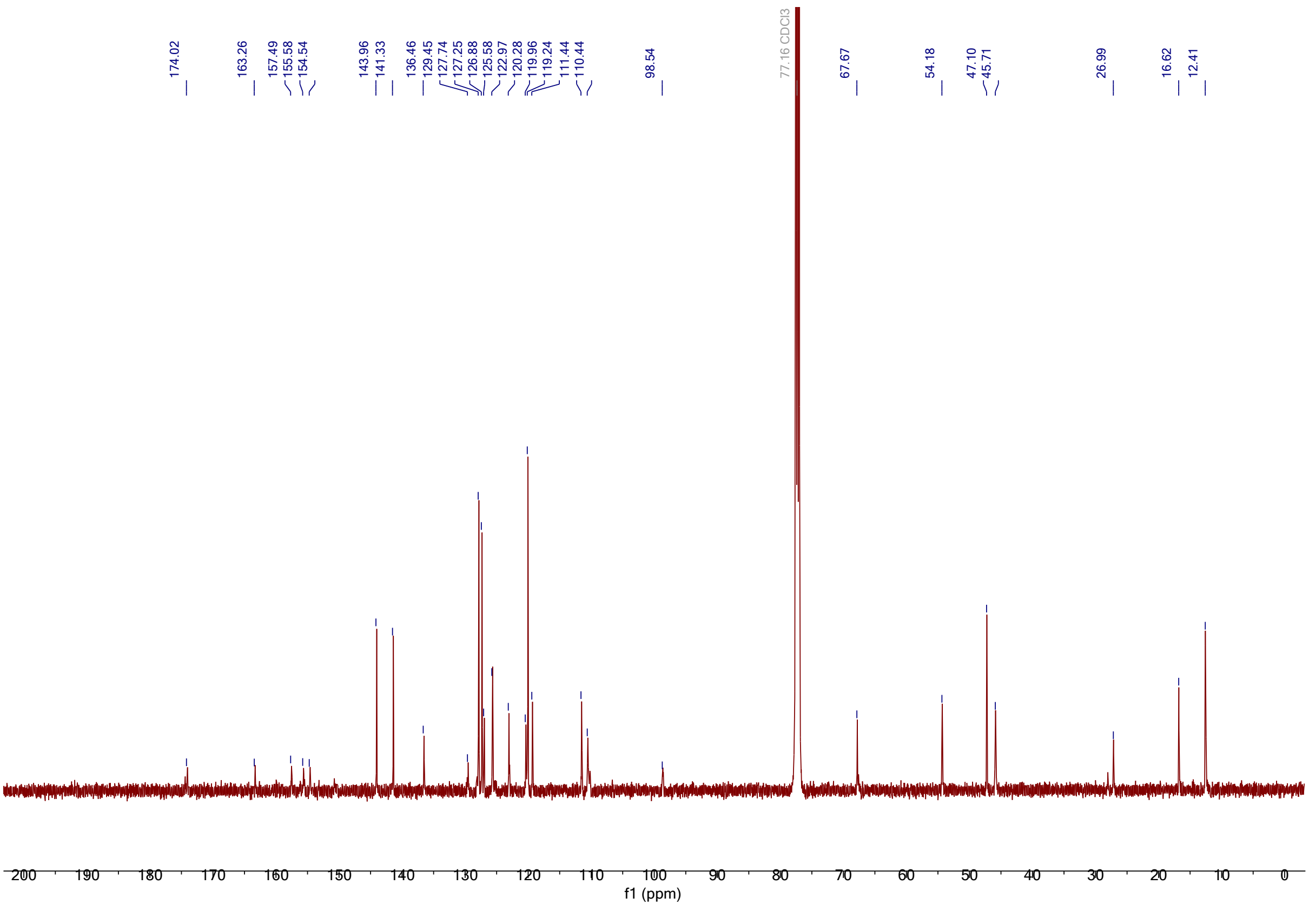

Compound 12 (DMSO-d_6)

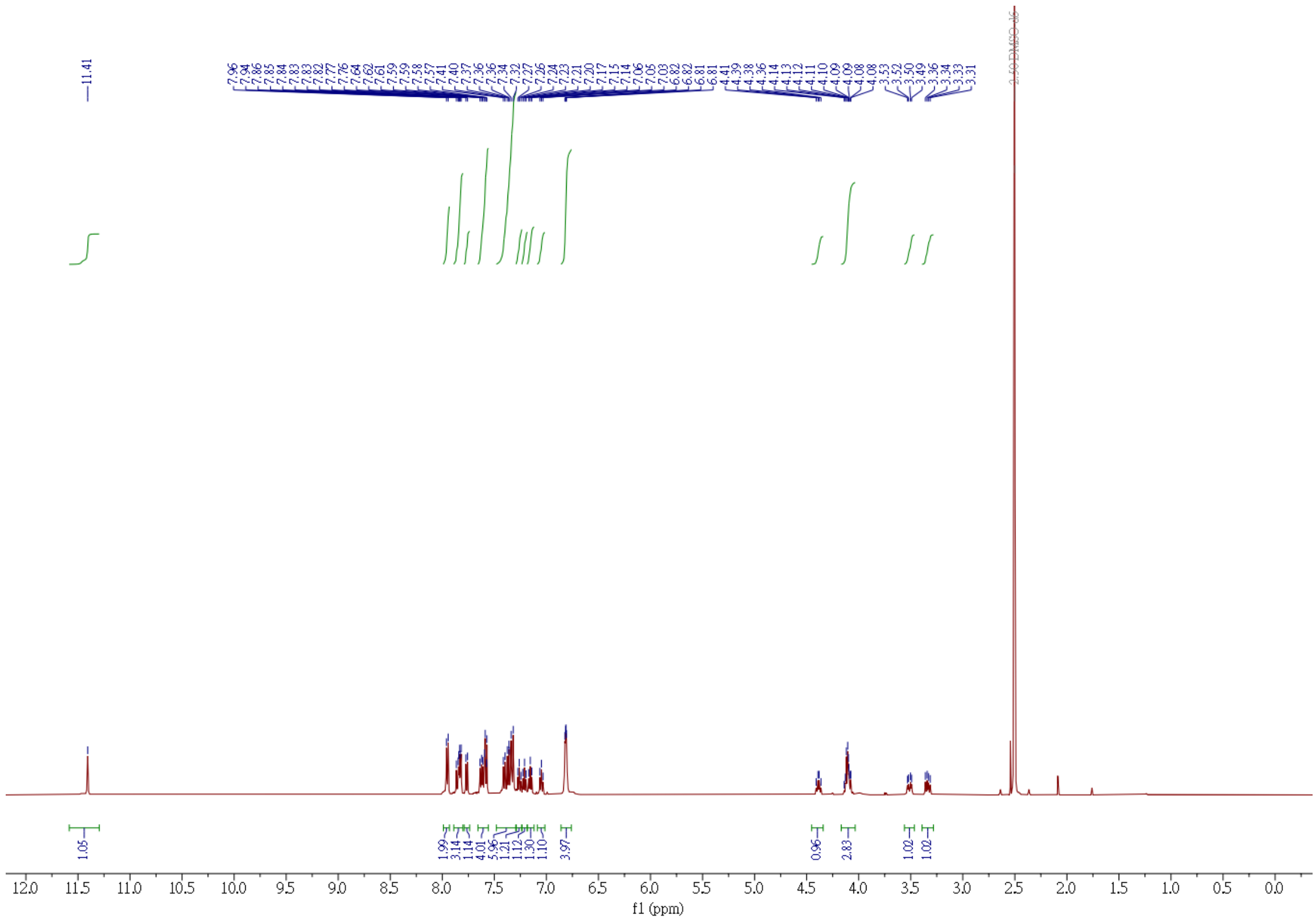

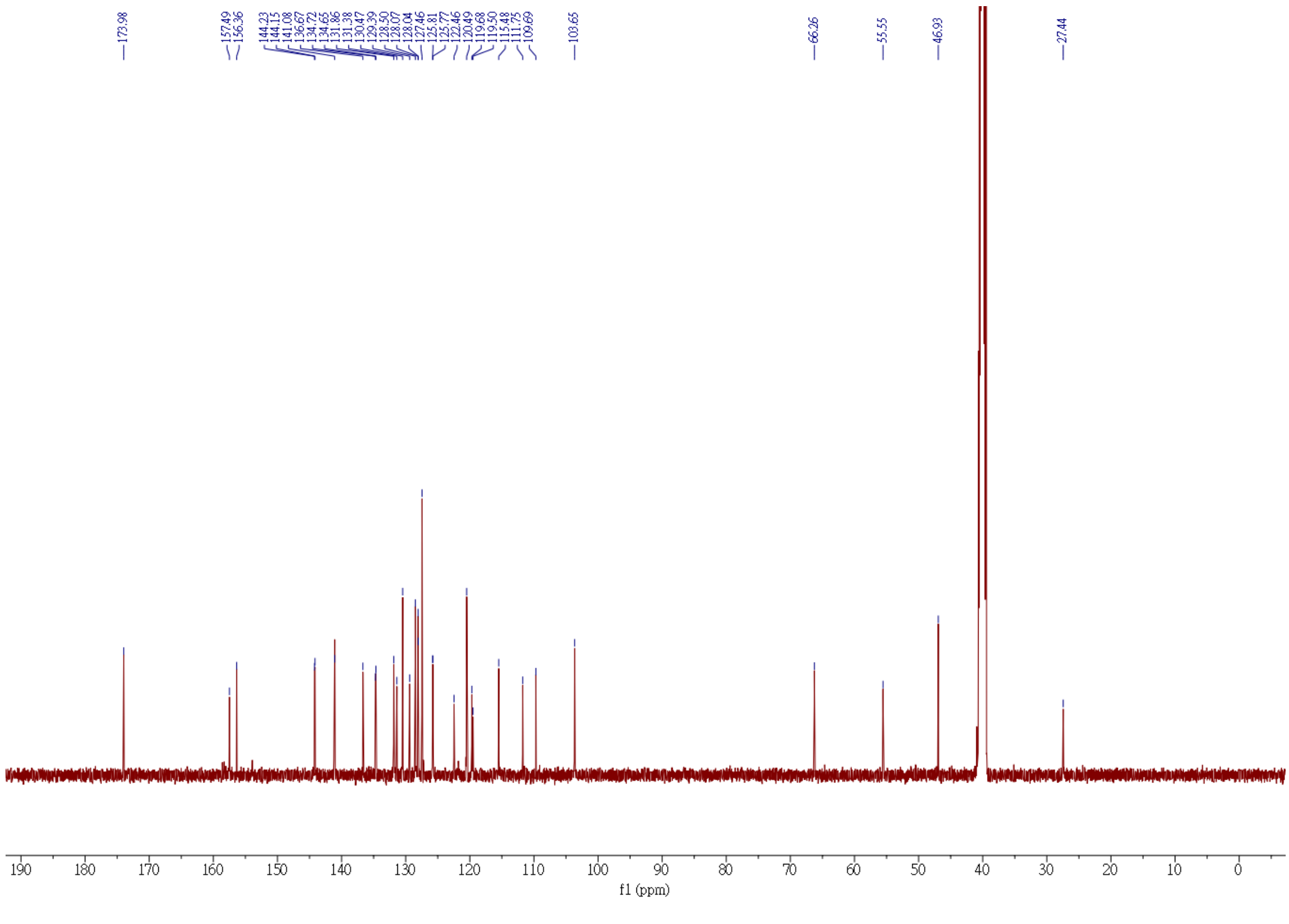

Compound 13 (CD_3OD)

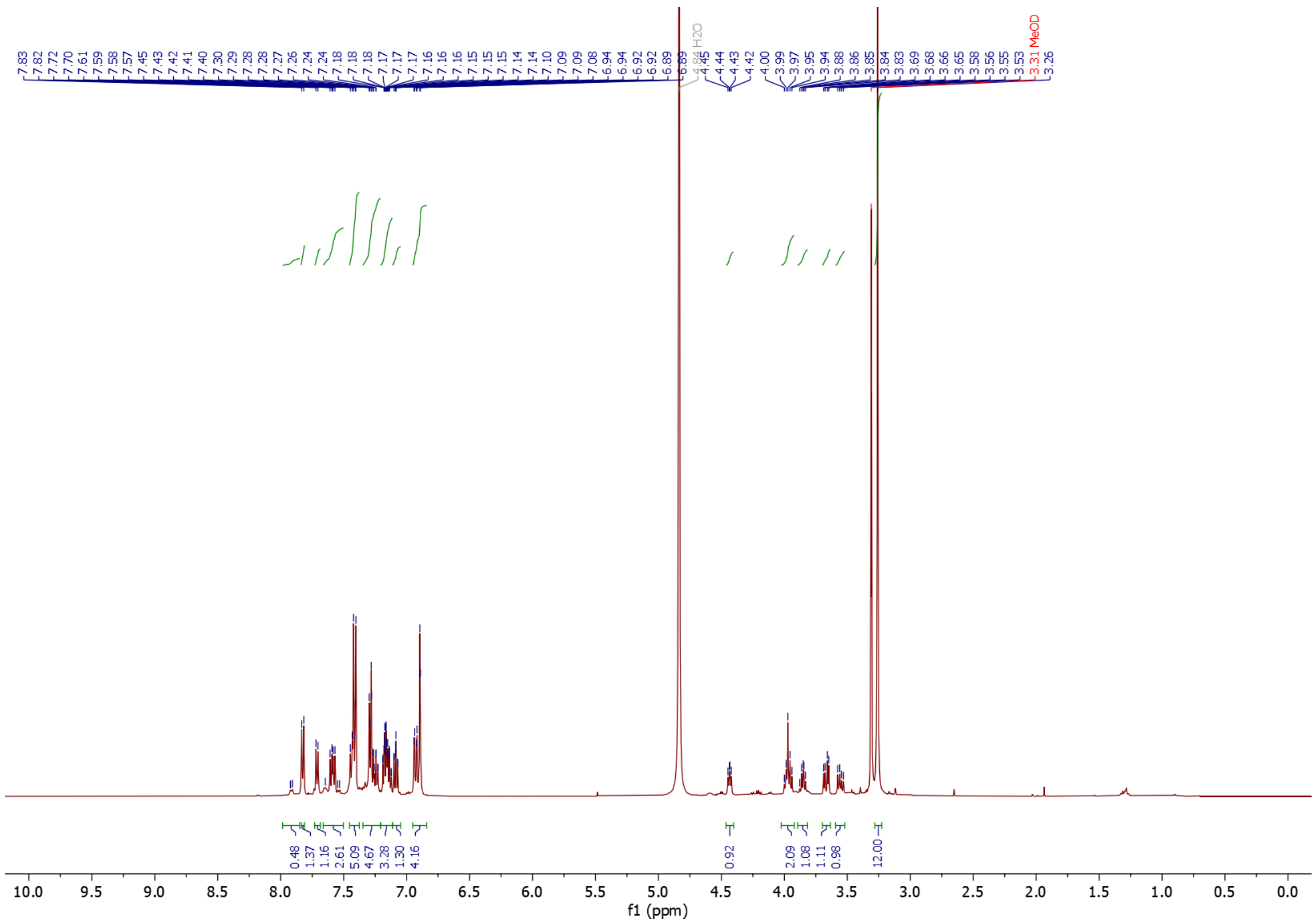

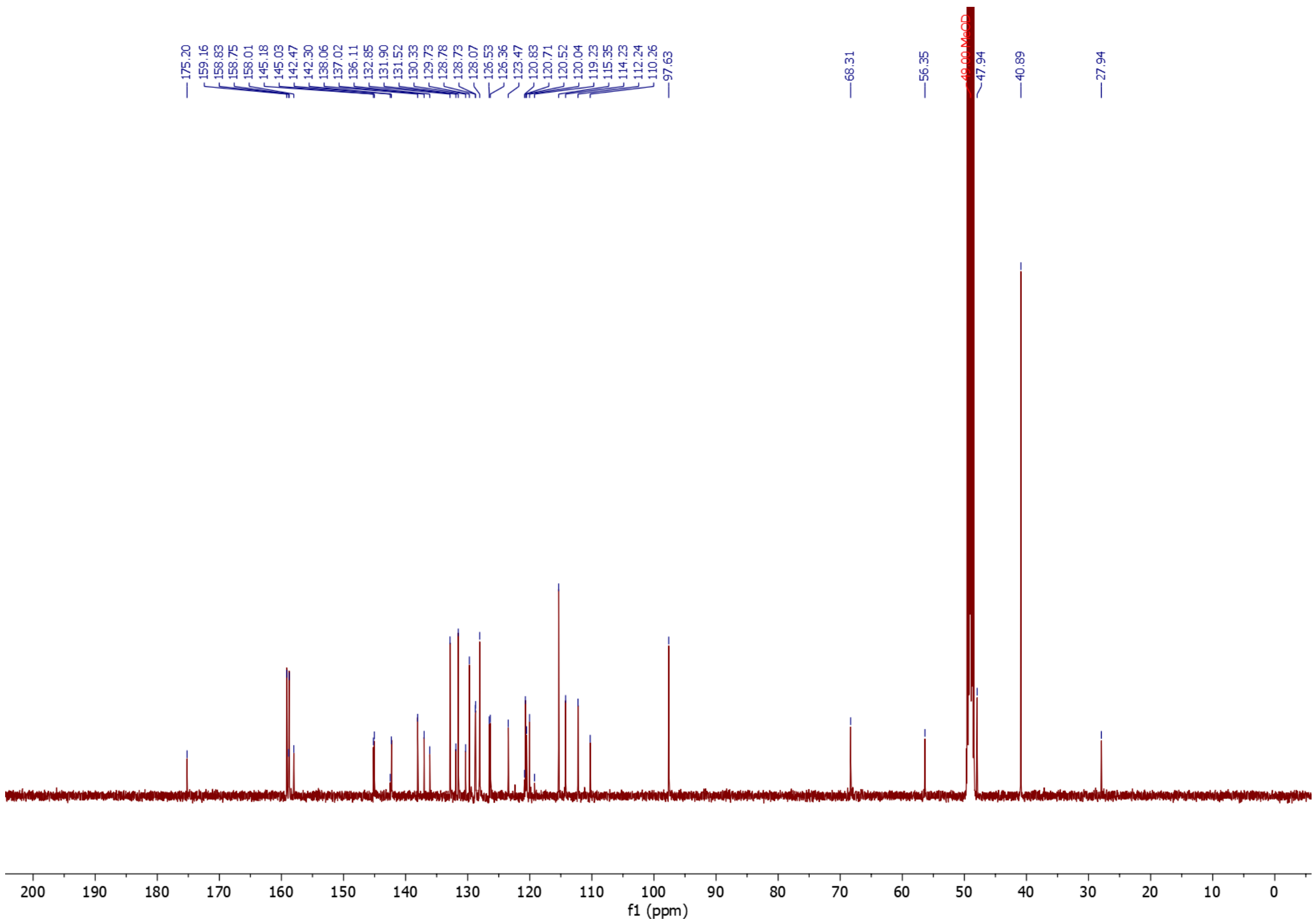

Compound 14 (^{1}H (DMSO-d_6) and ^{13}C (CD_3OD))

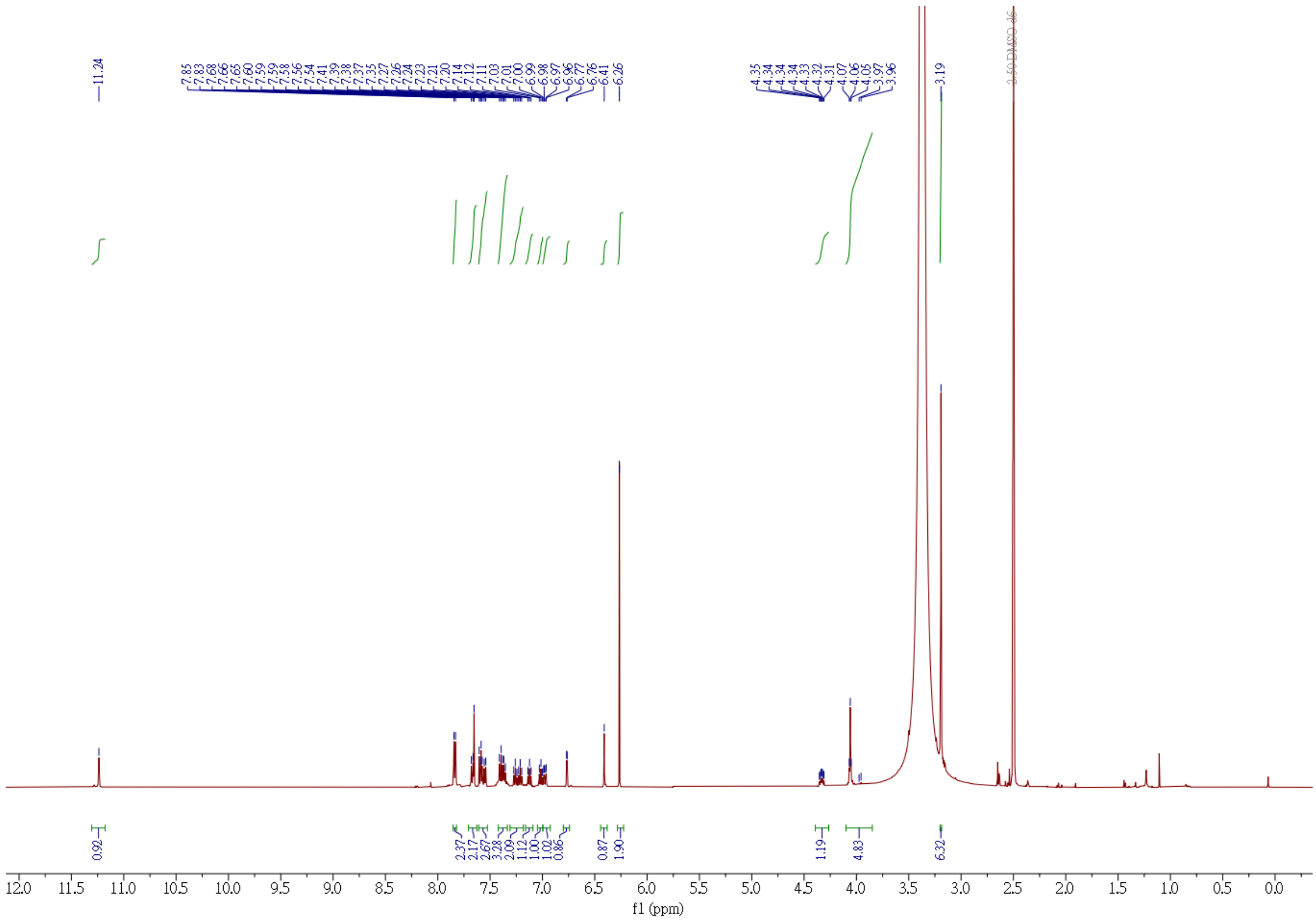

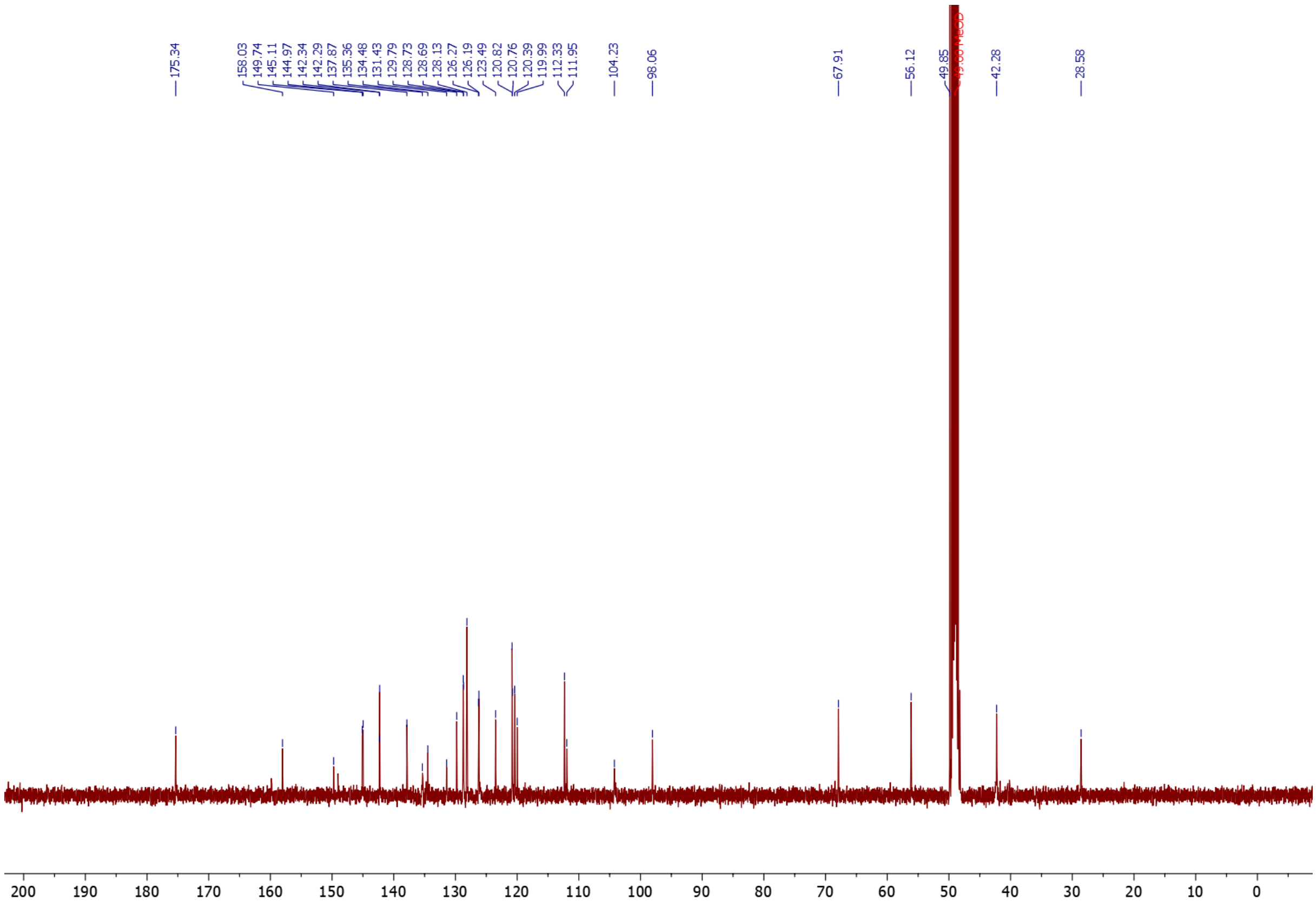

Compound 15 (CDCl$_3$)

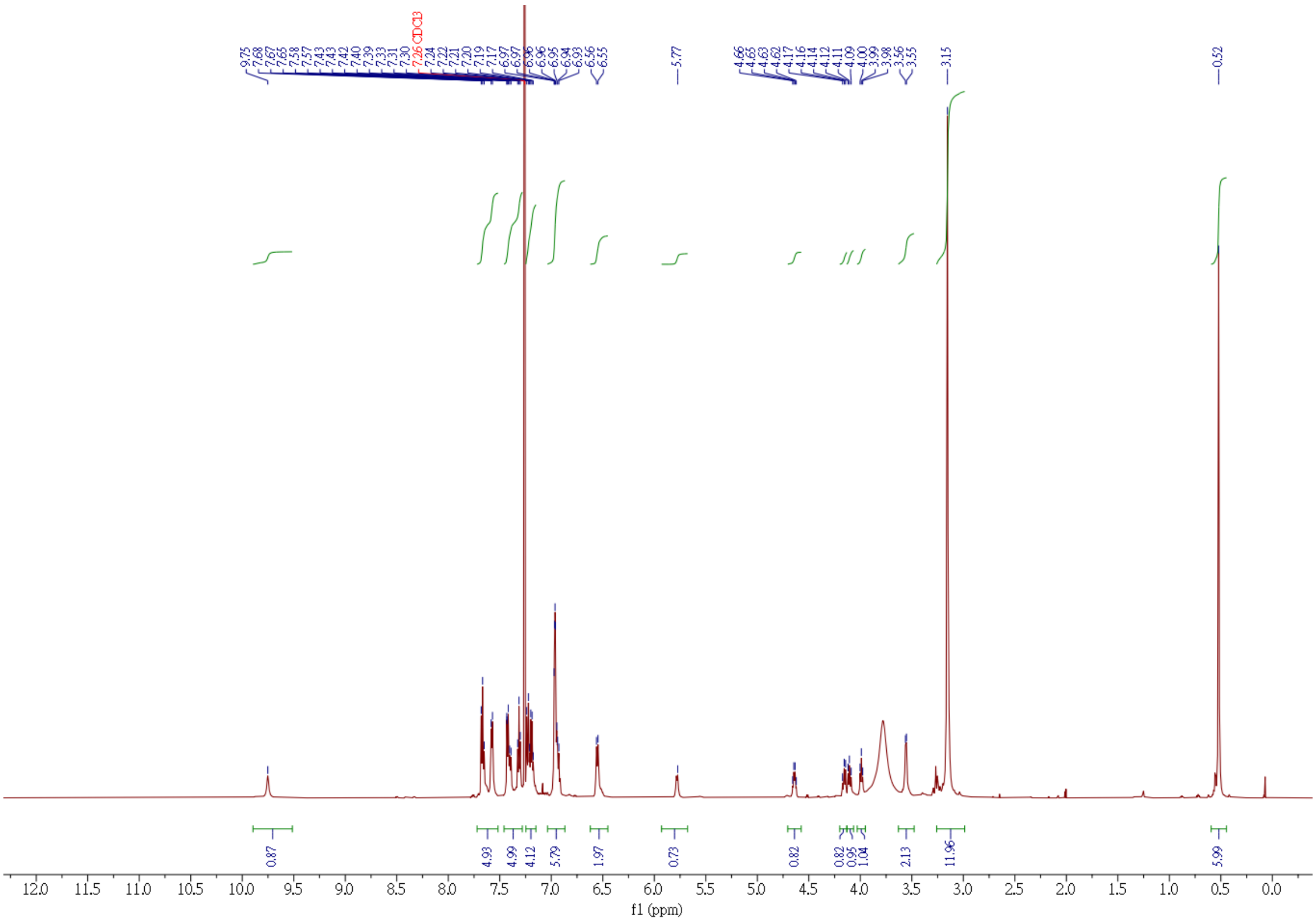

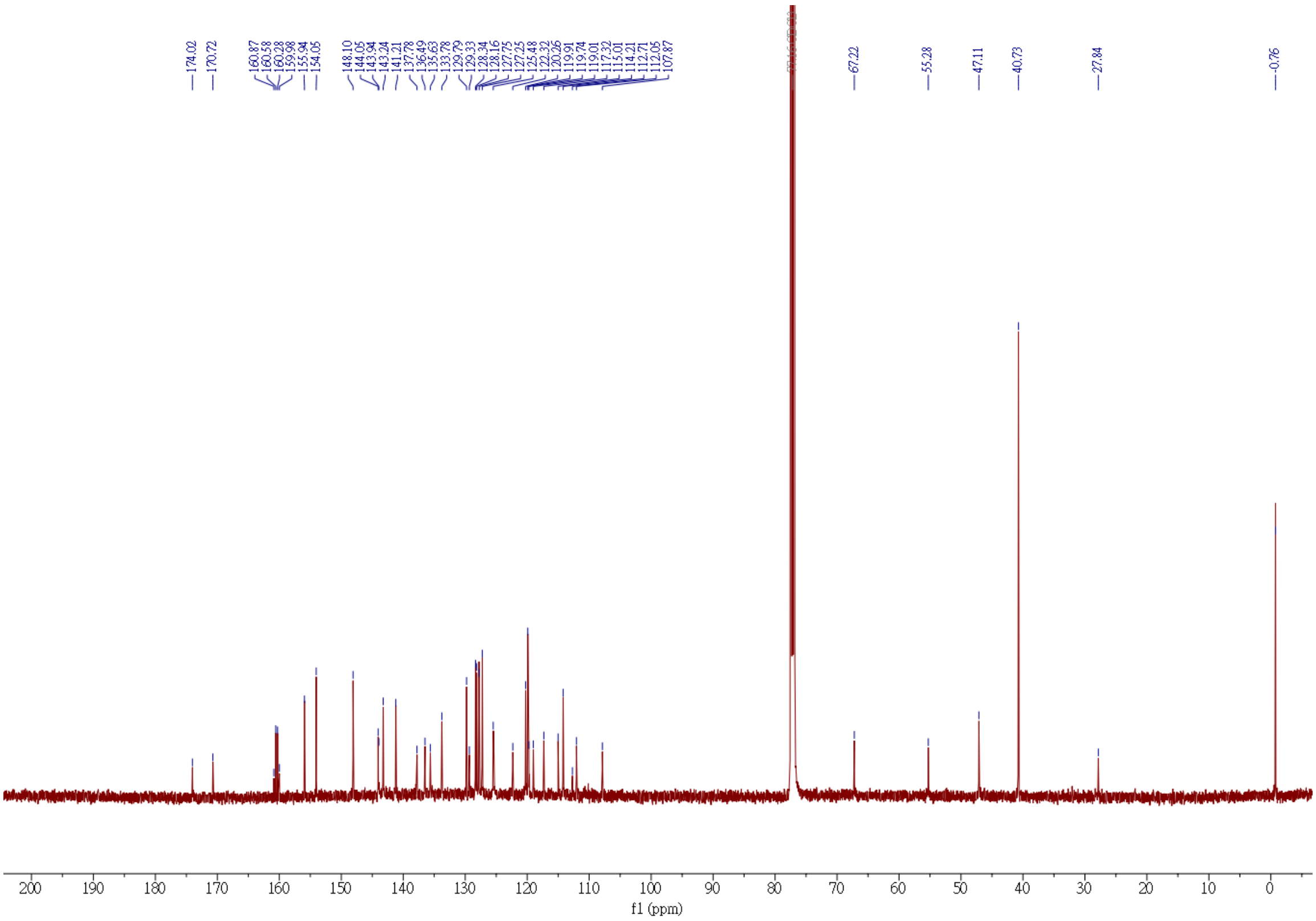

Compound 18 (CD_3OD)

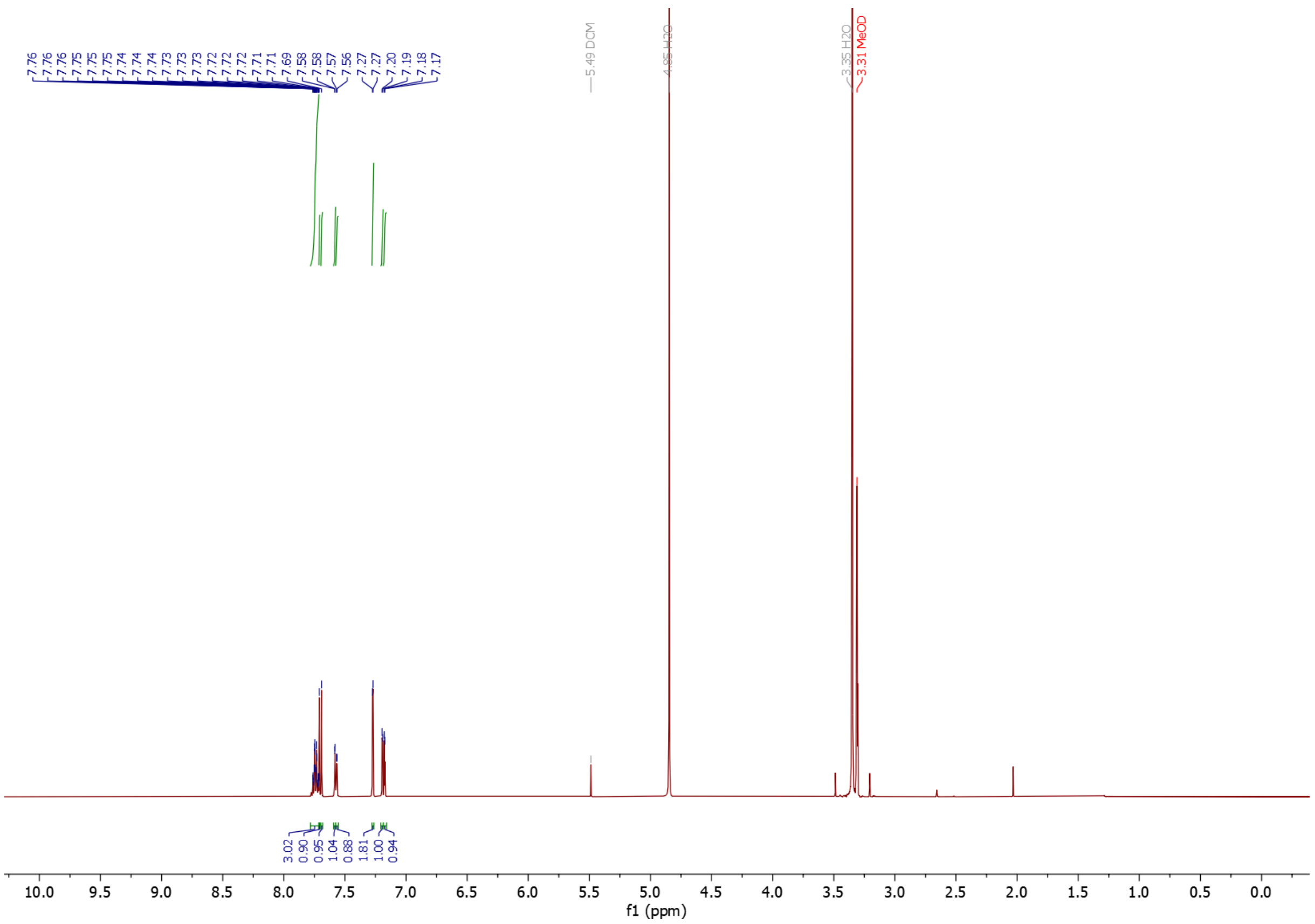

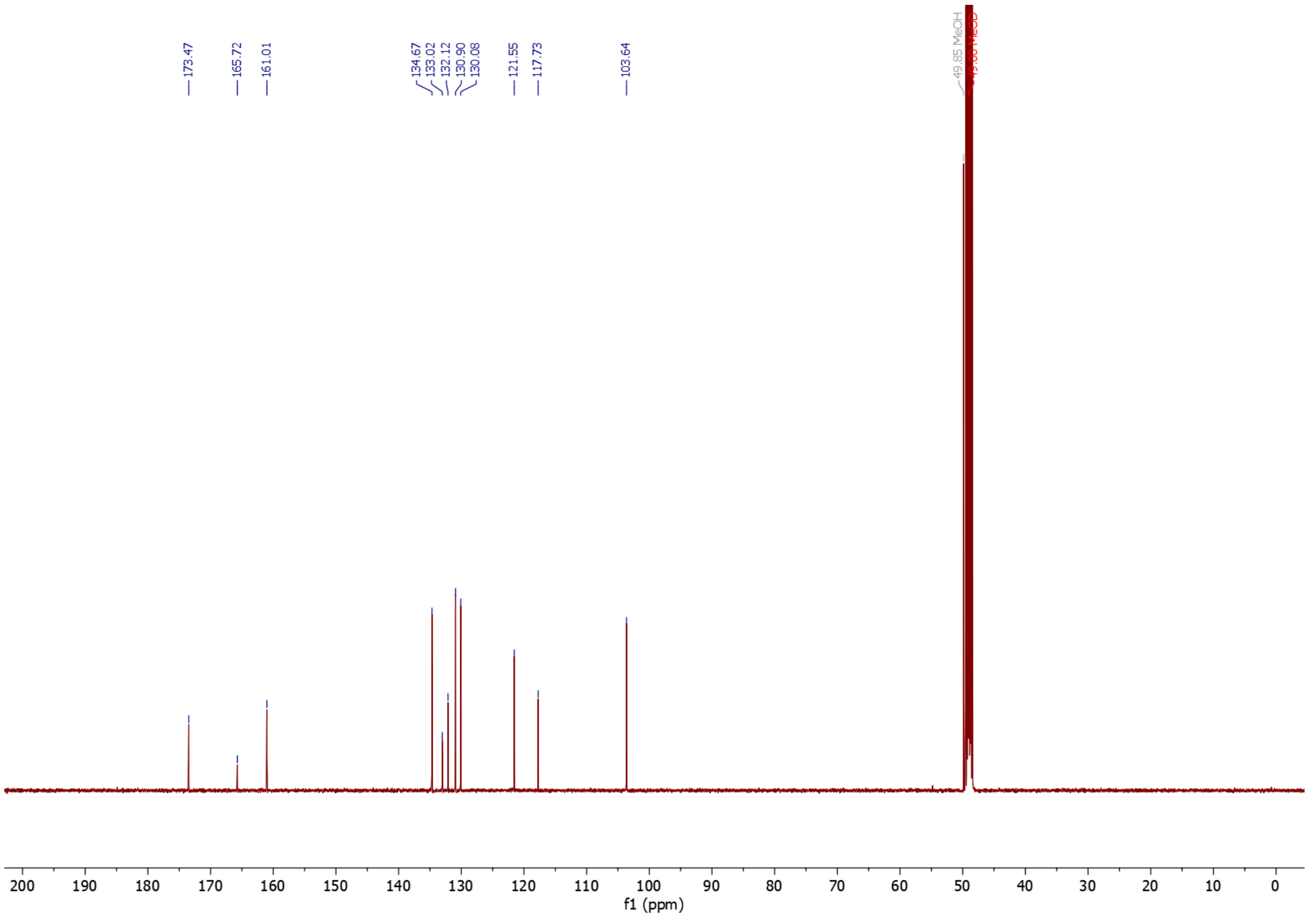

Compound 19 (CD_3OD)

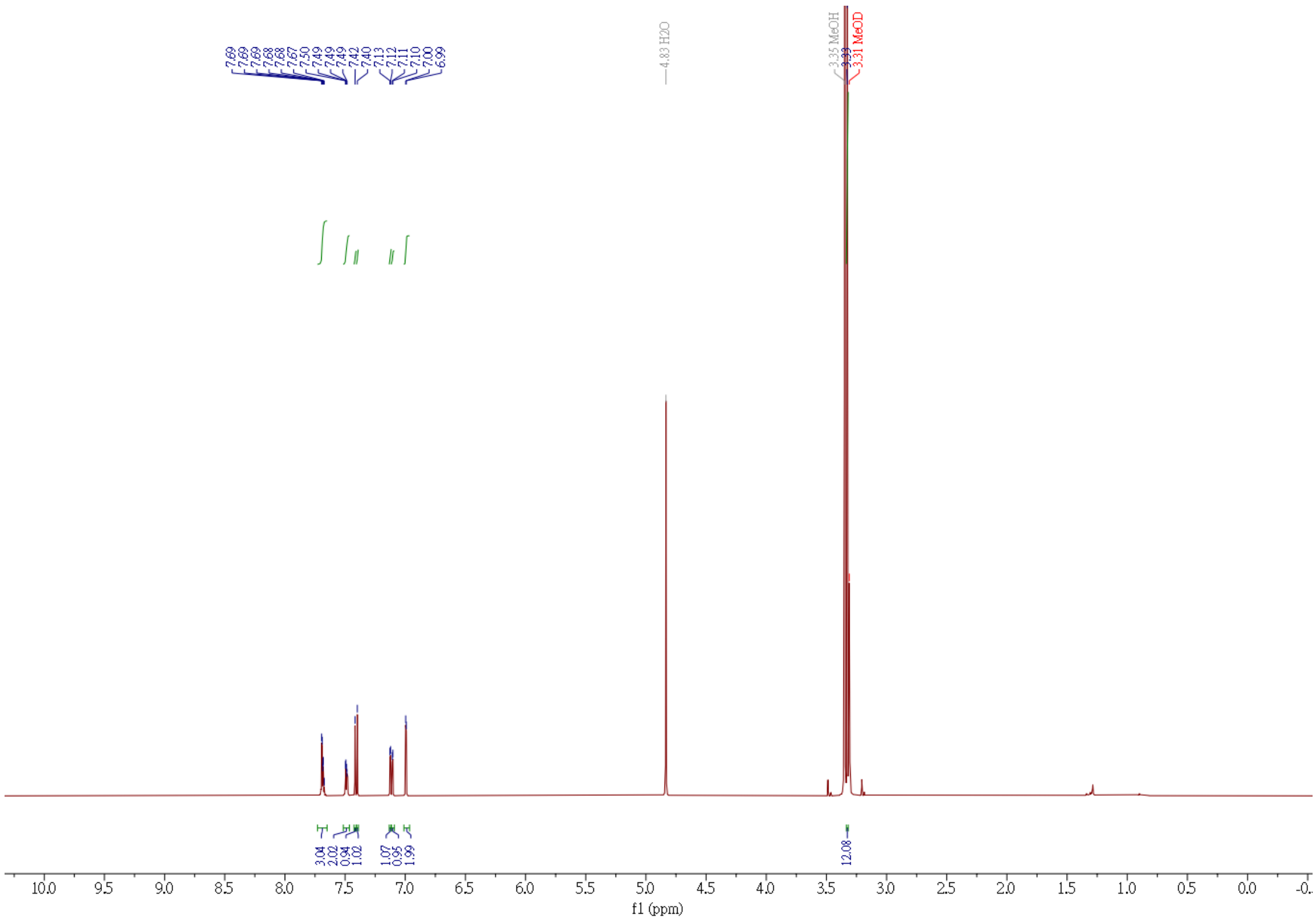

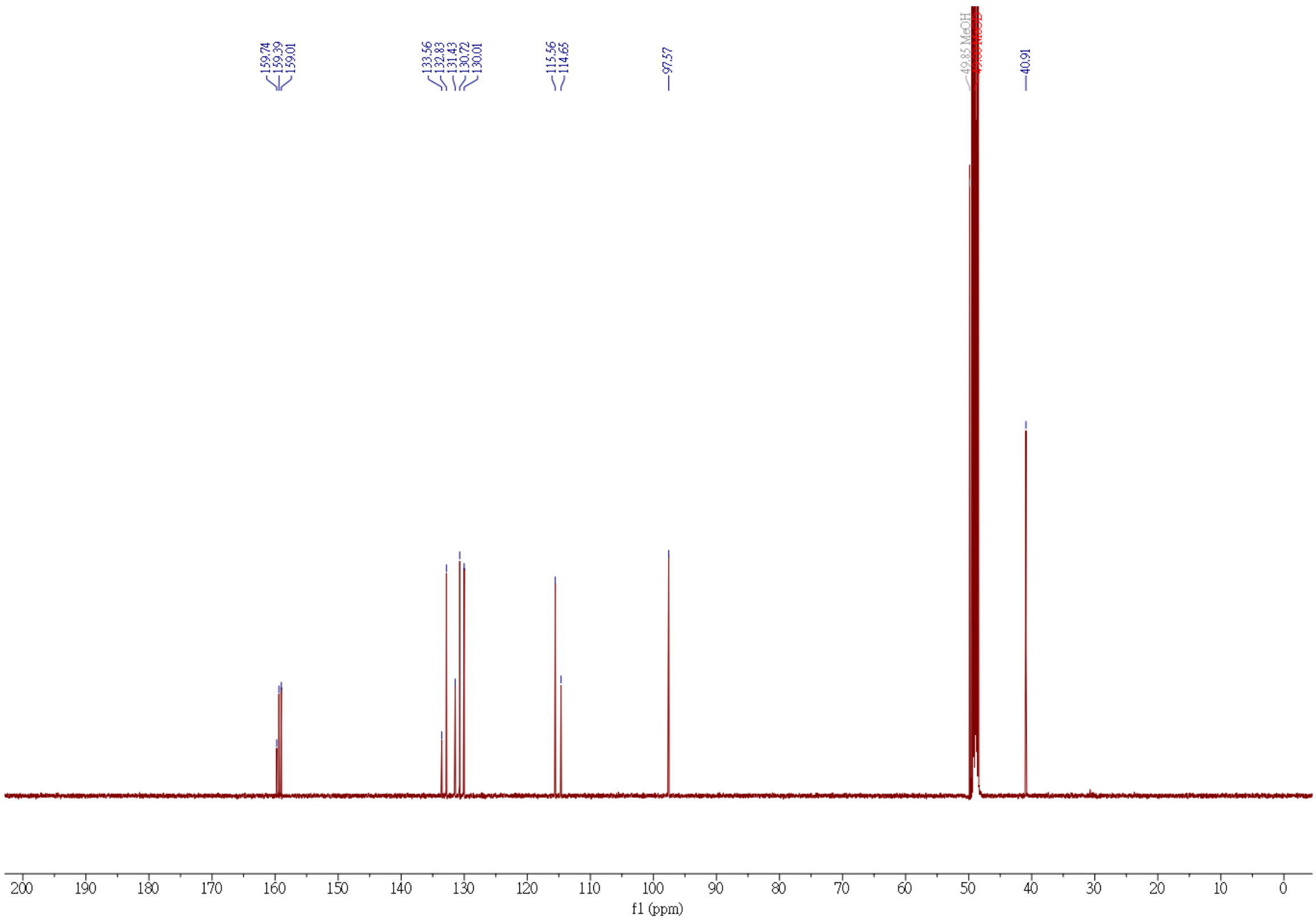

Compound 20 (CD_3OD)

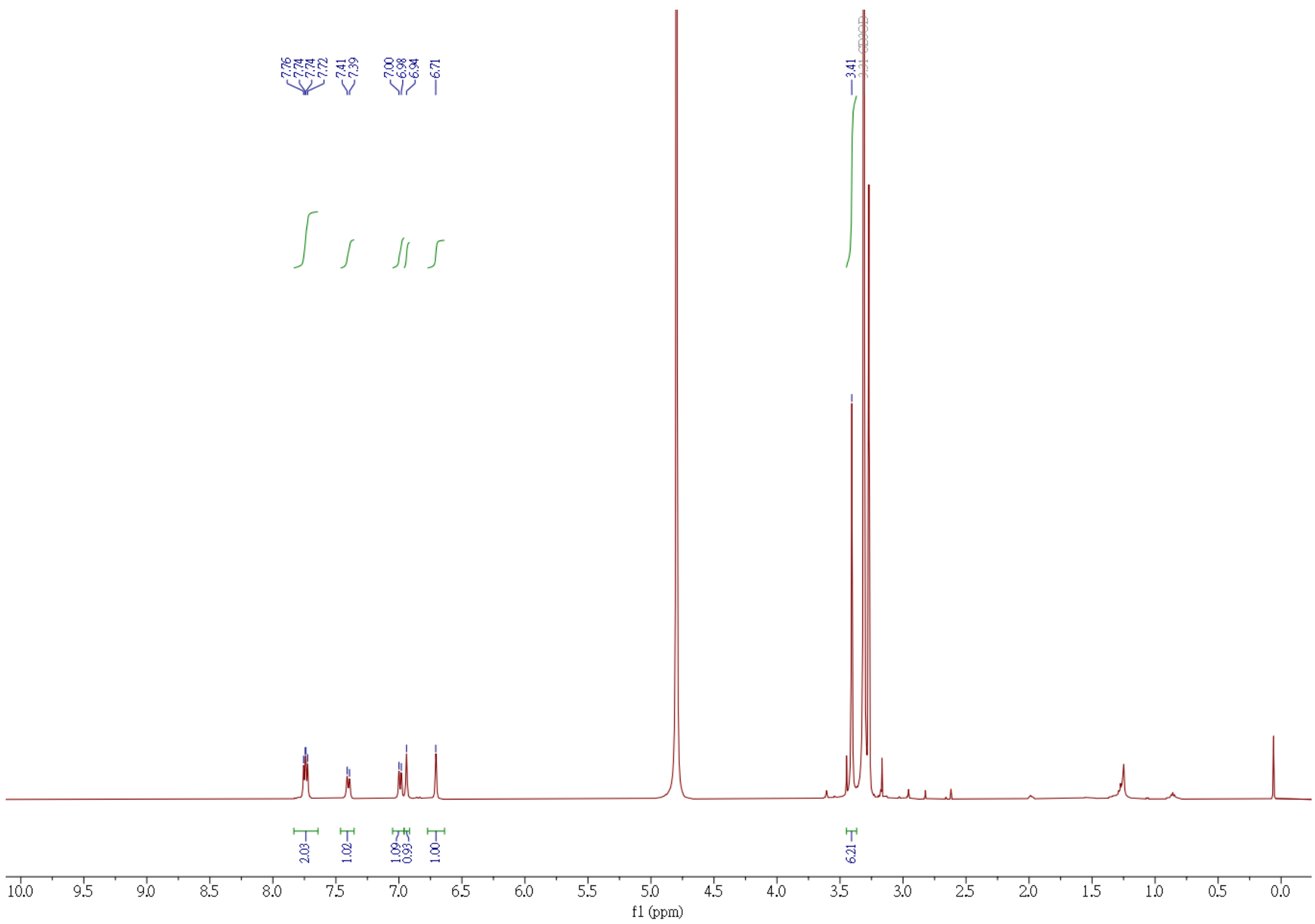

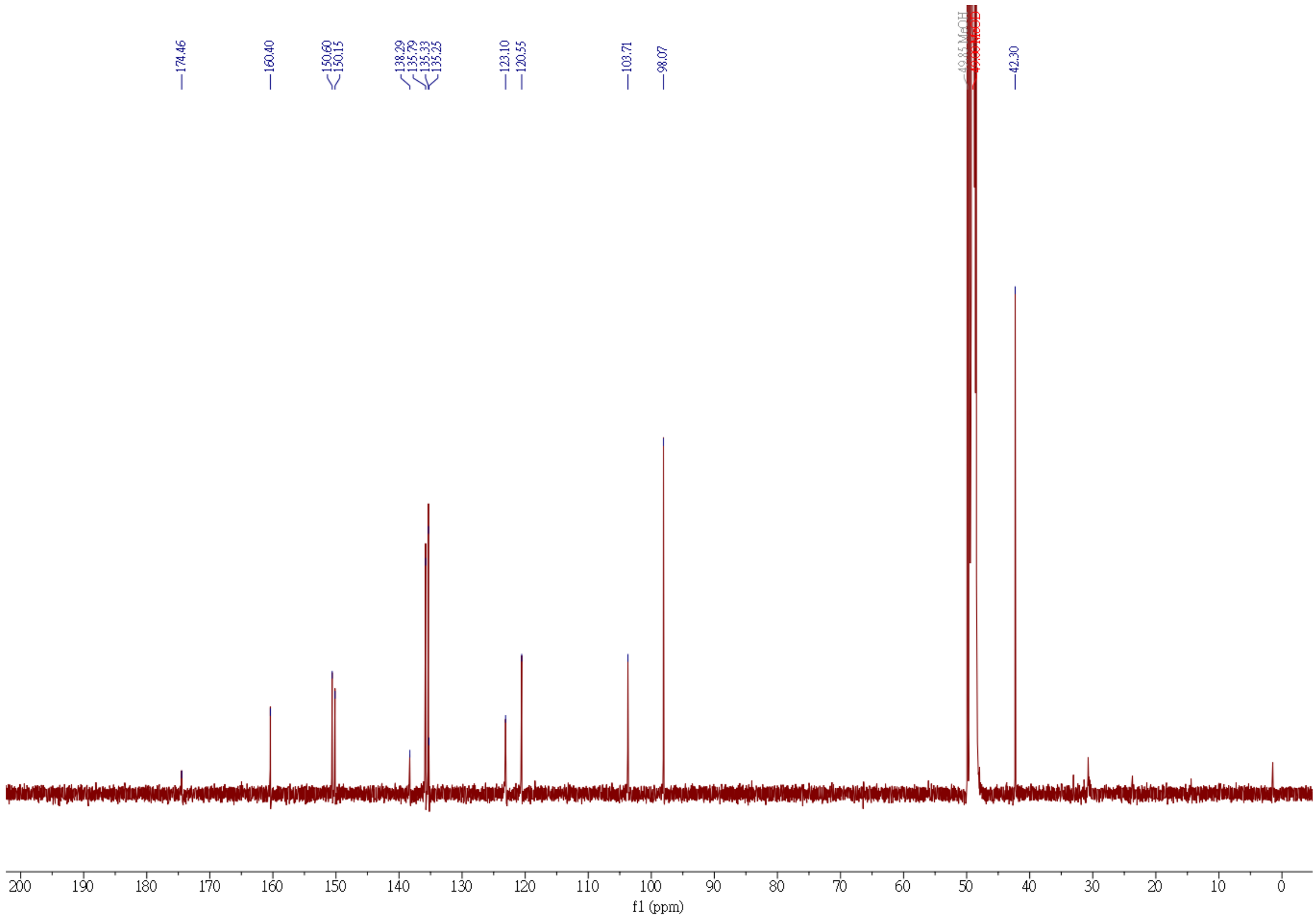

Compound 21 ($CDCl_3$)

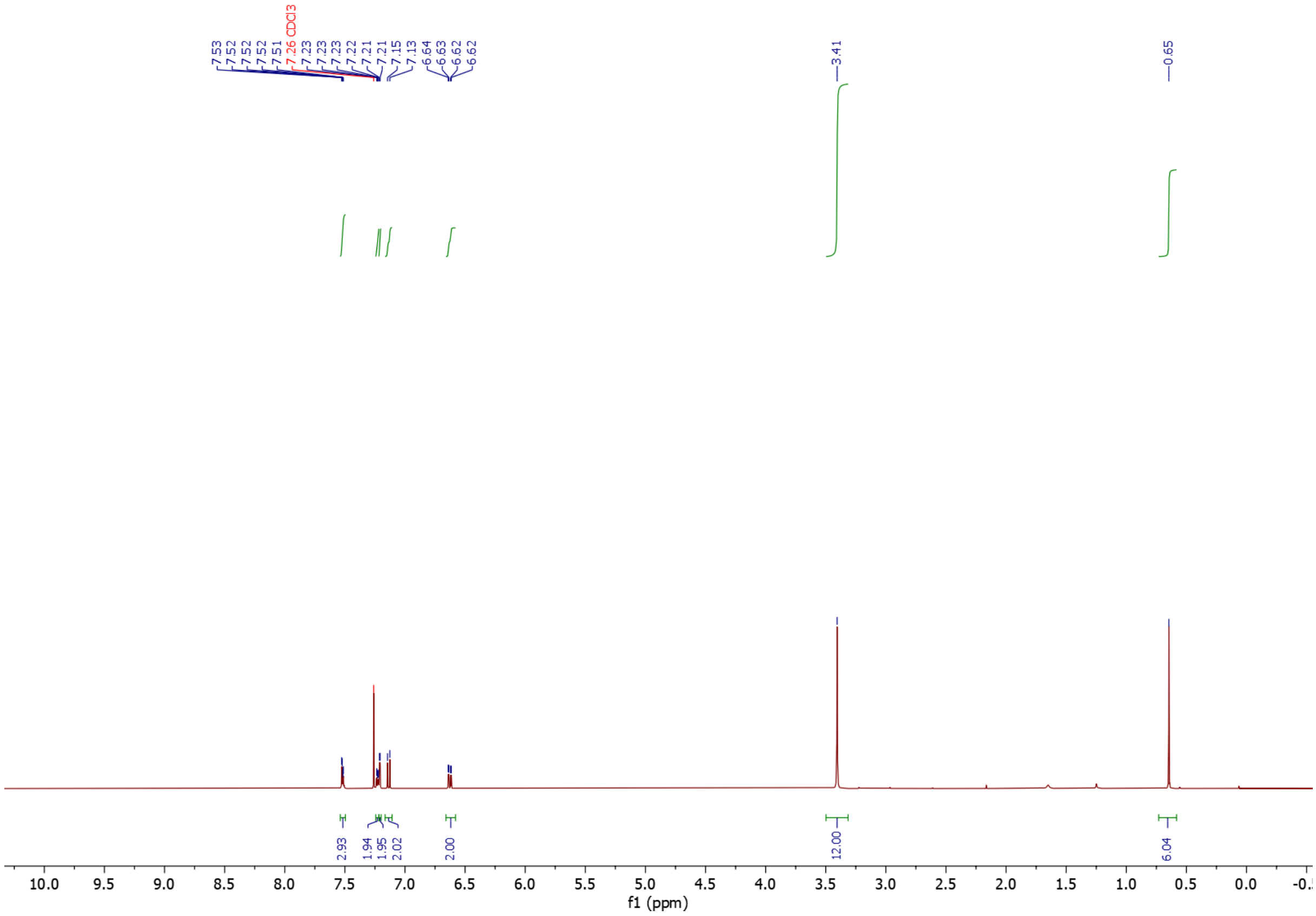

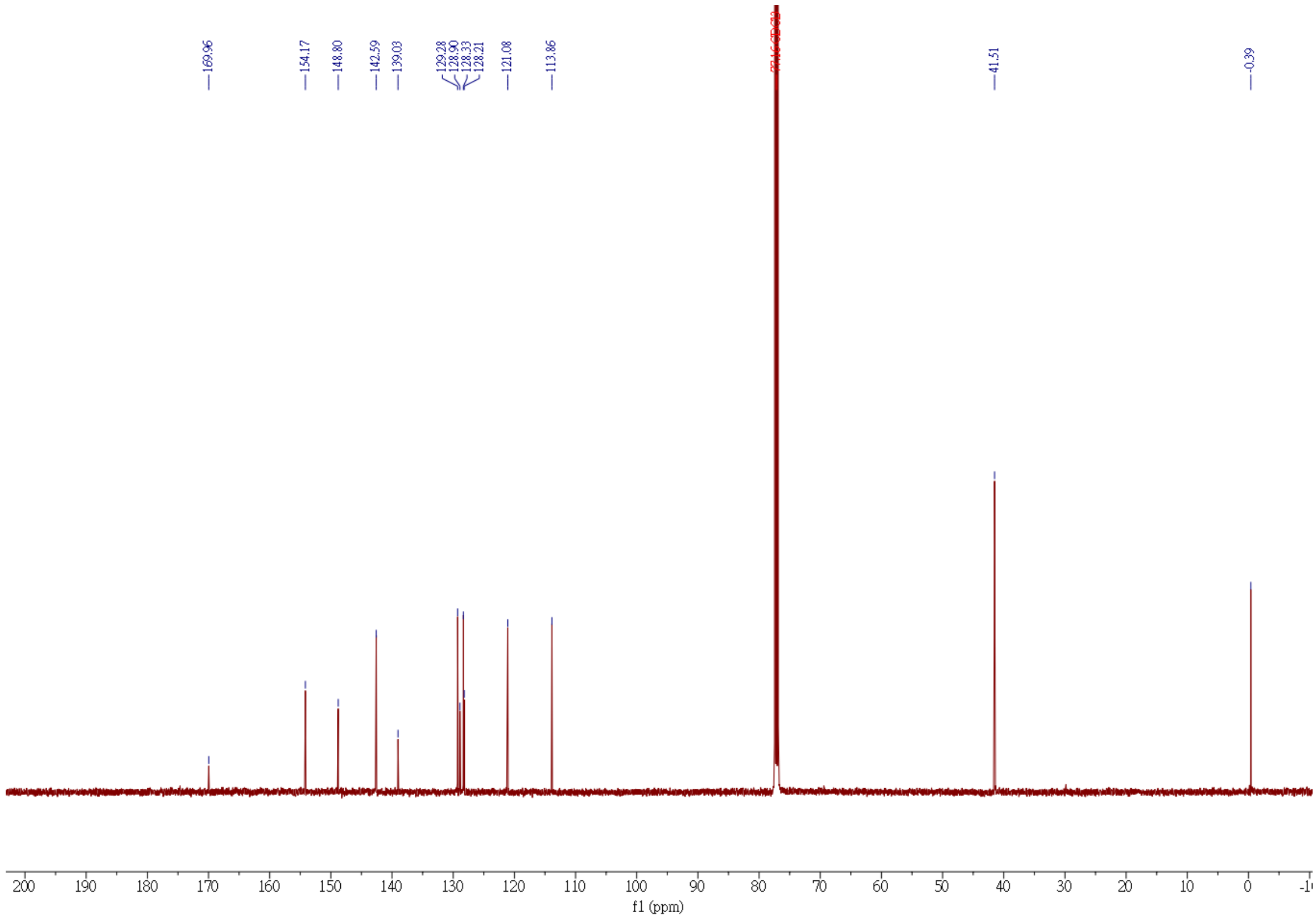

Compound 25 ($CDCl_3$)

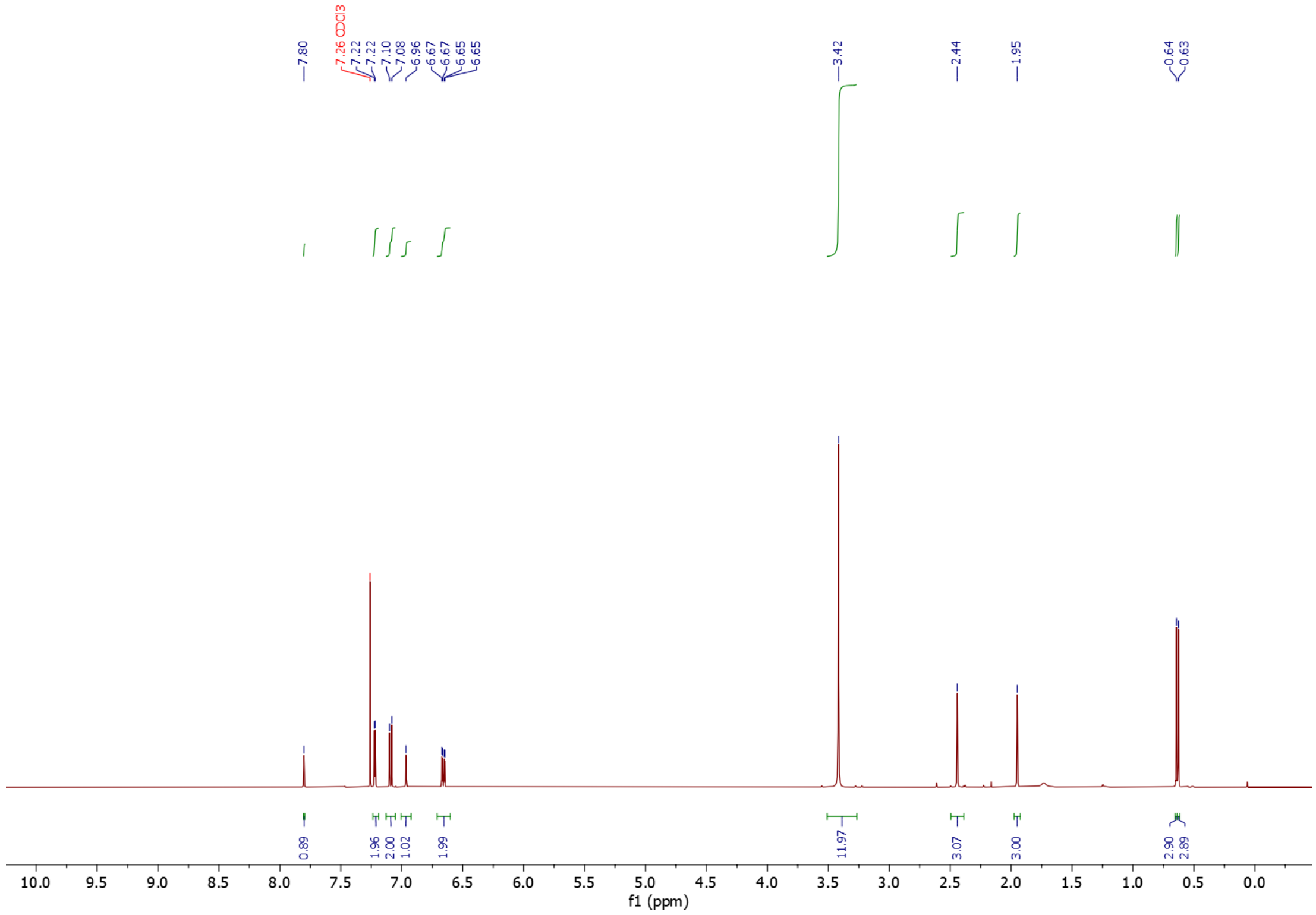

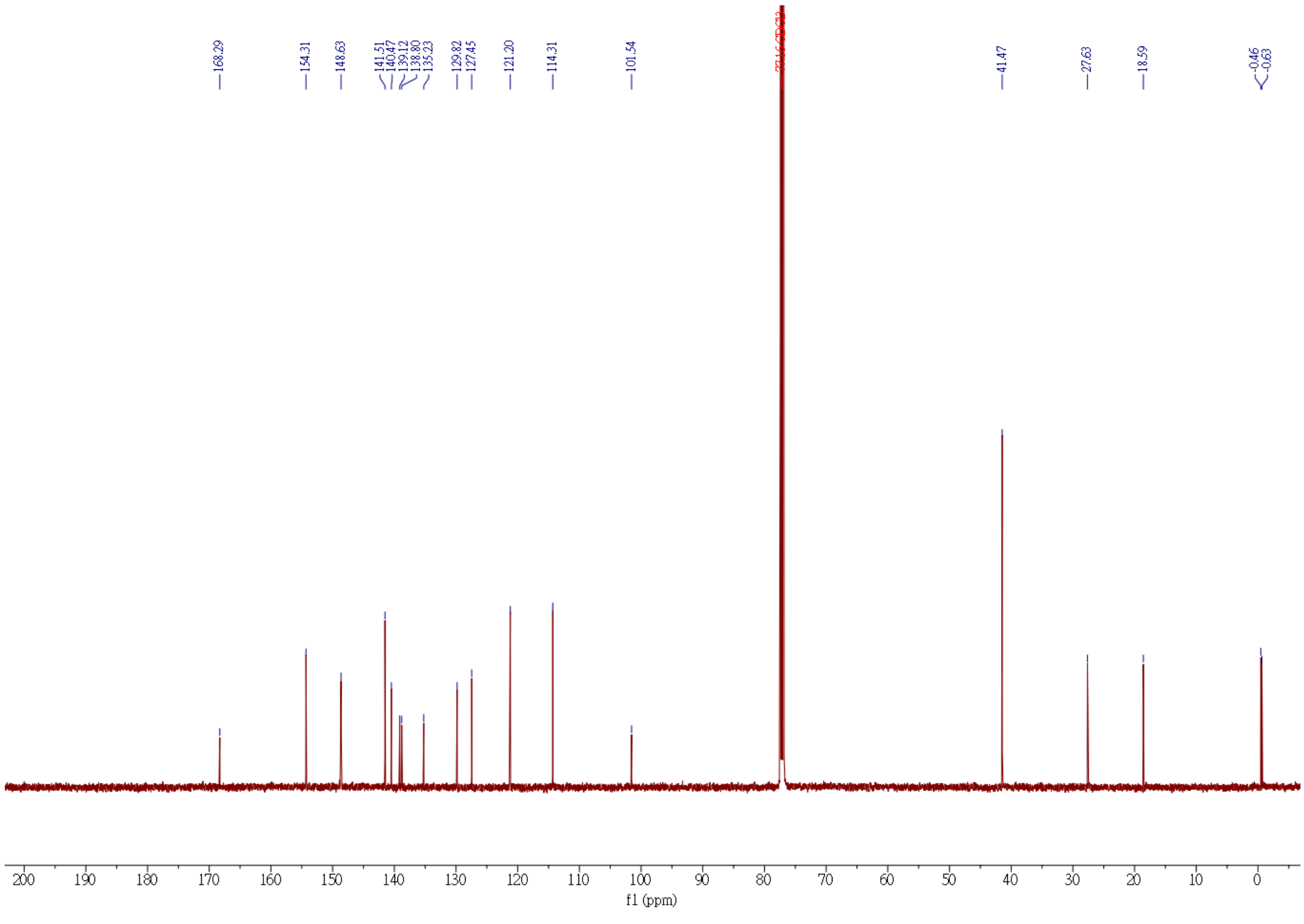

Compound 26 ($CDCl_3$)

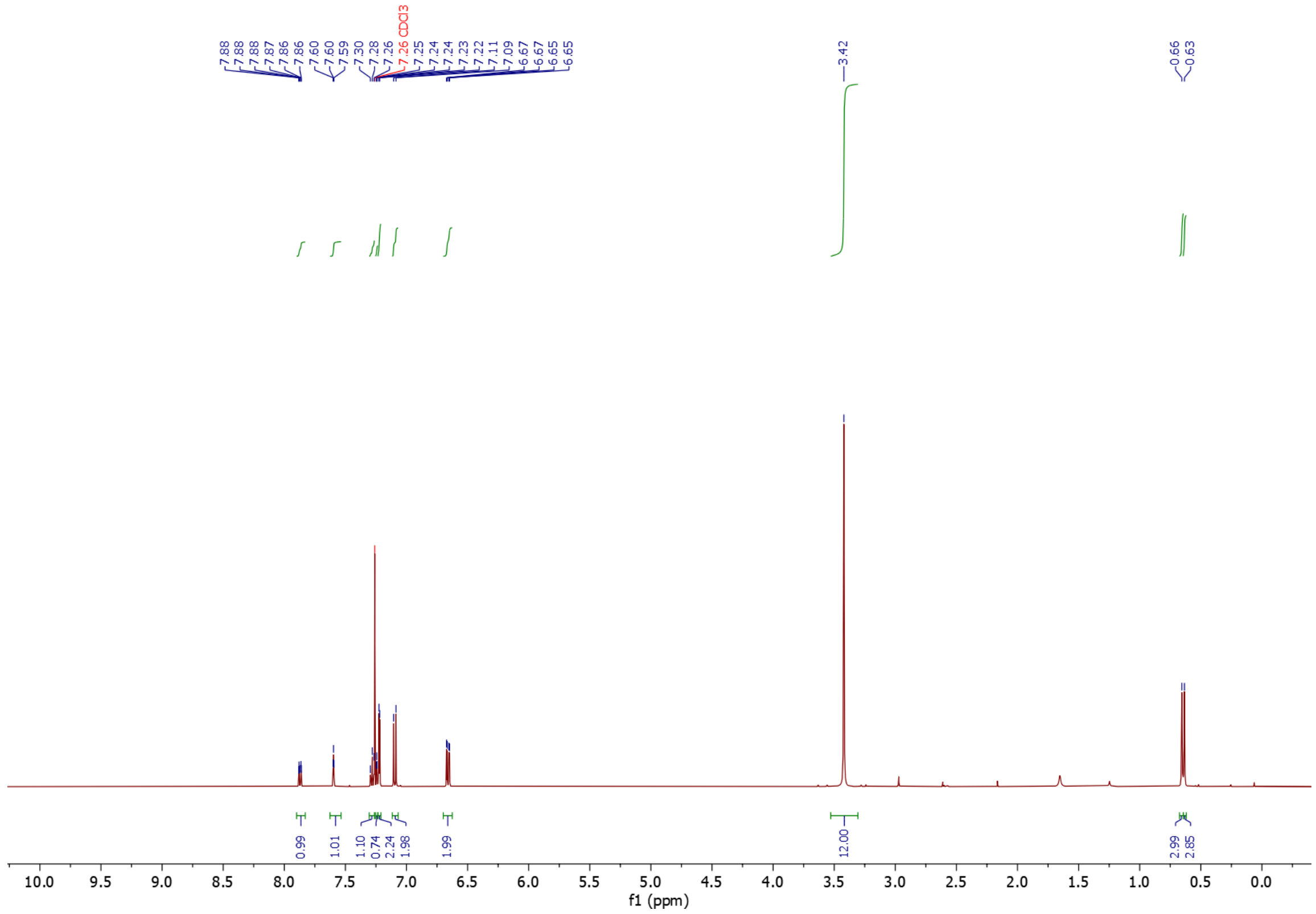

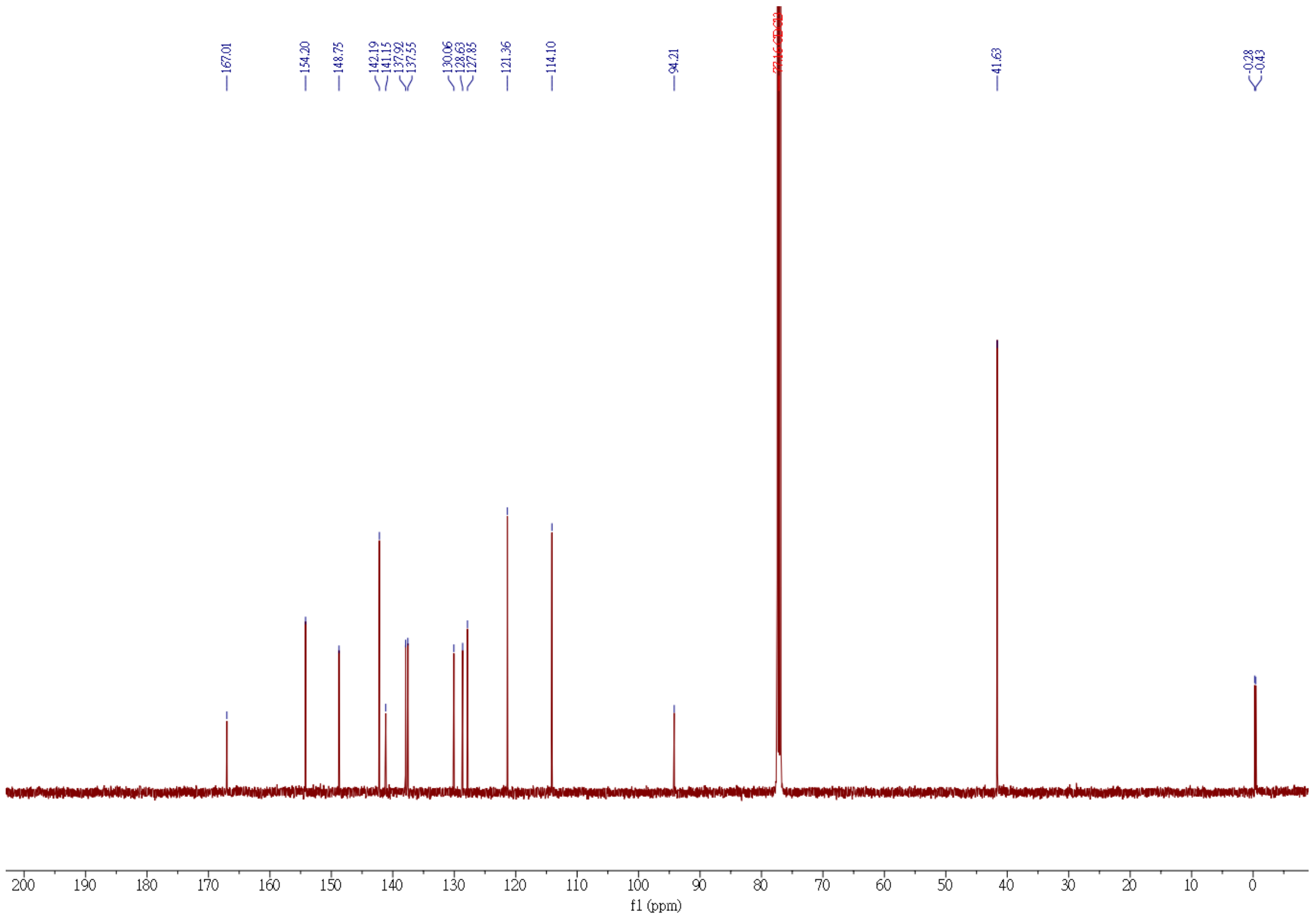

Compound 27 ($CDCl_3$)

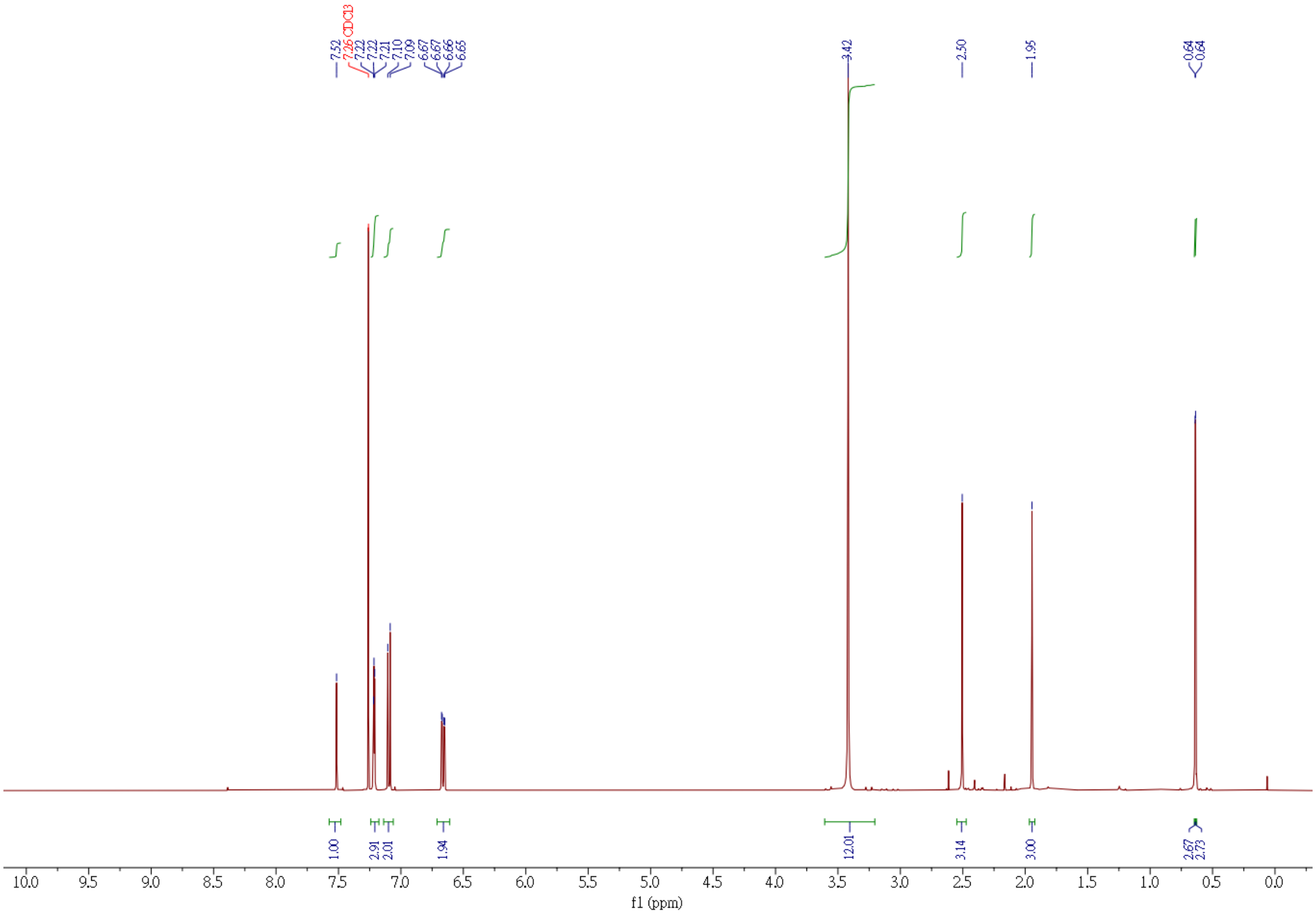

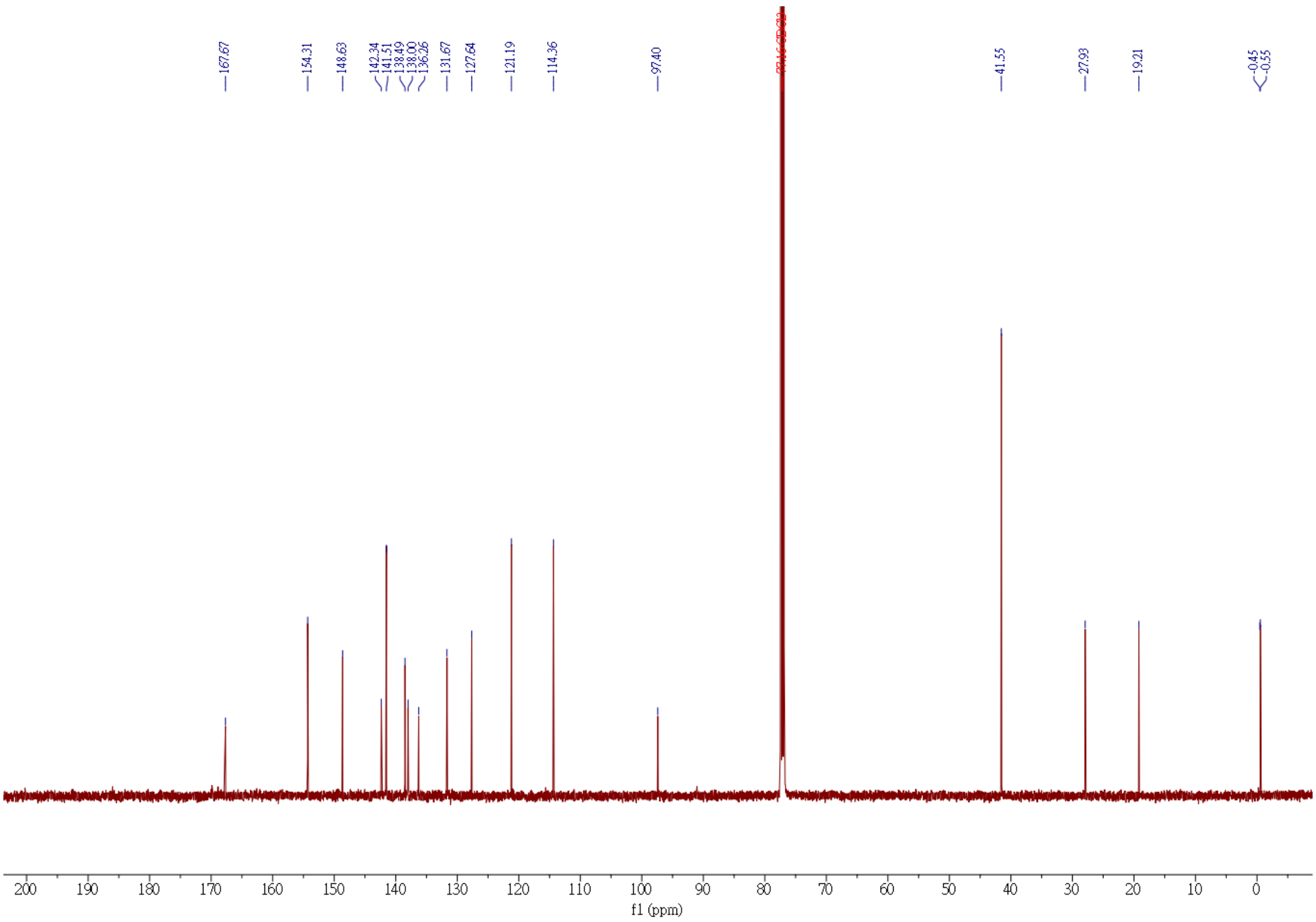

Compound 28 ($CDCl_3$)

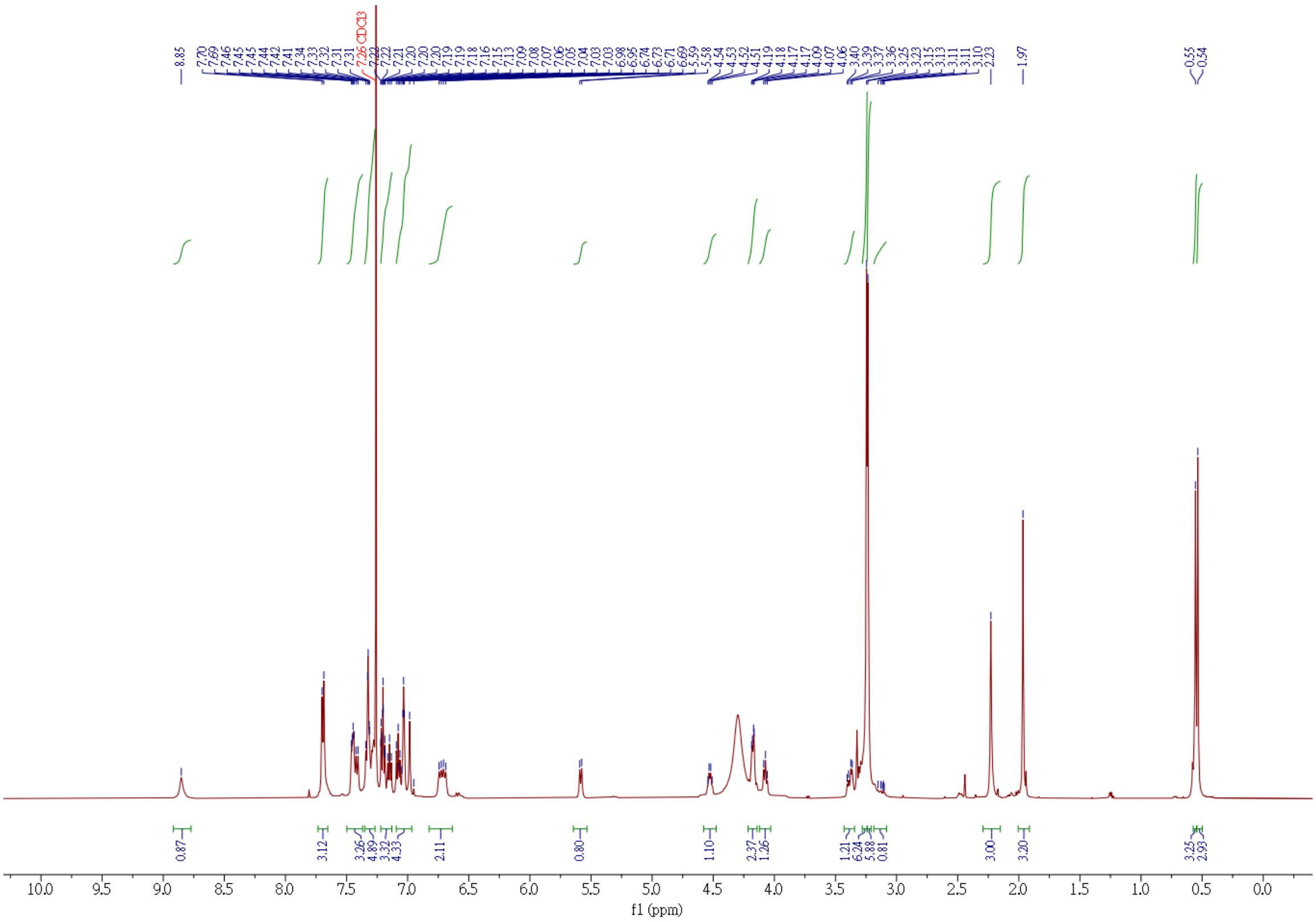

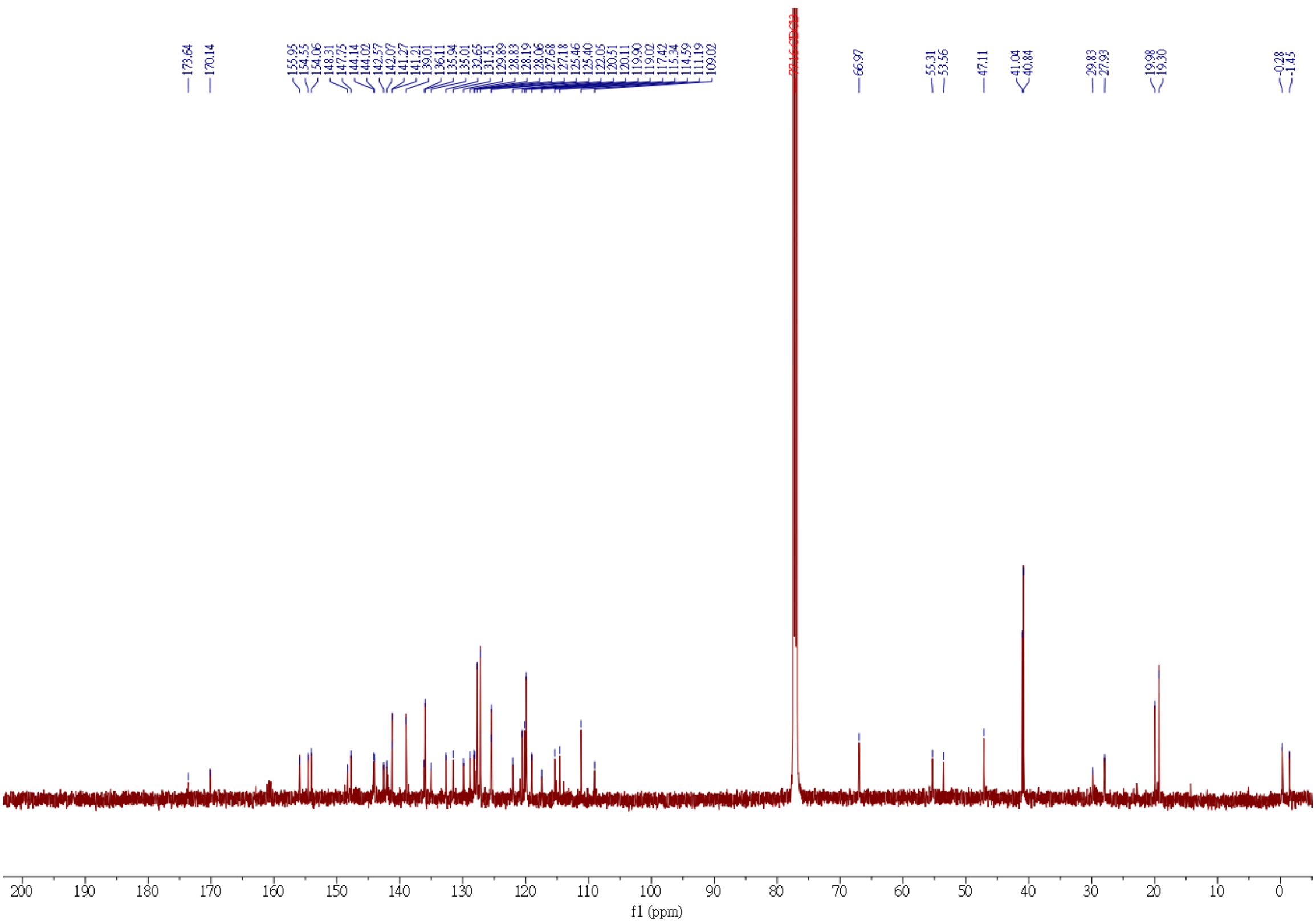

Compound 29 ($CDCl_3$)

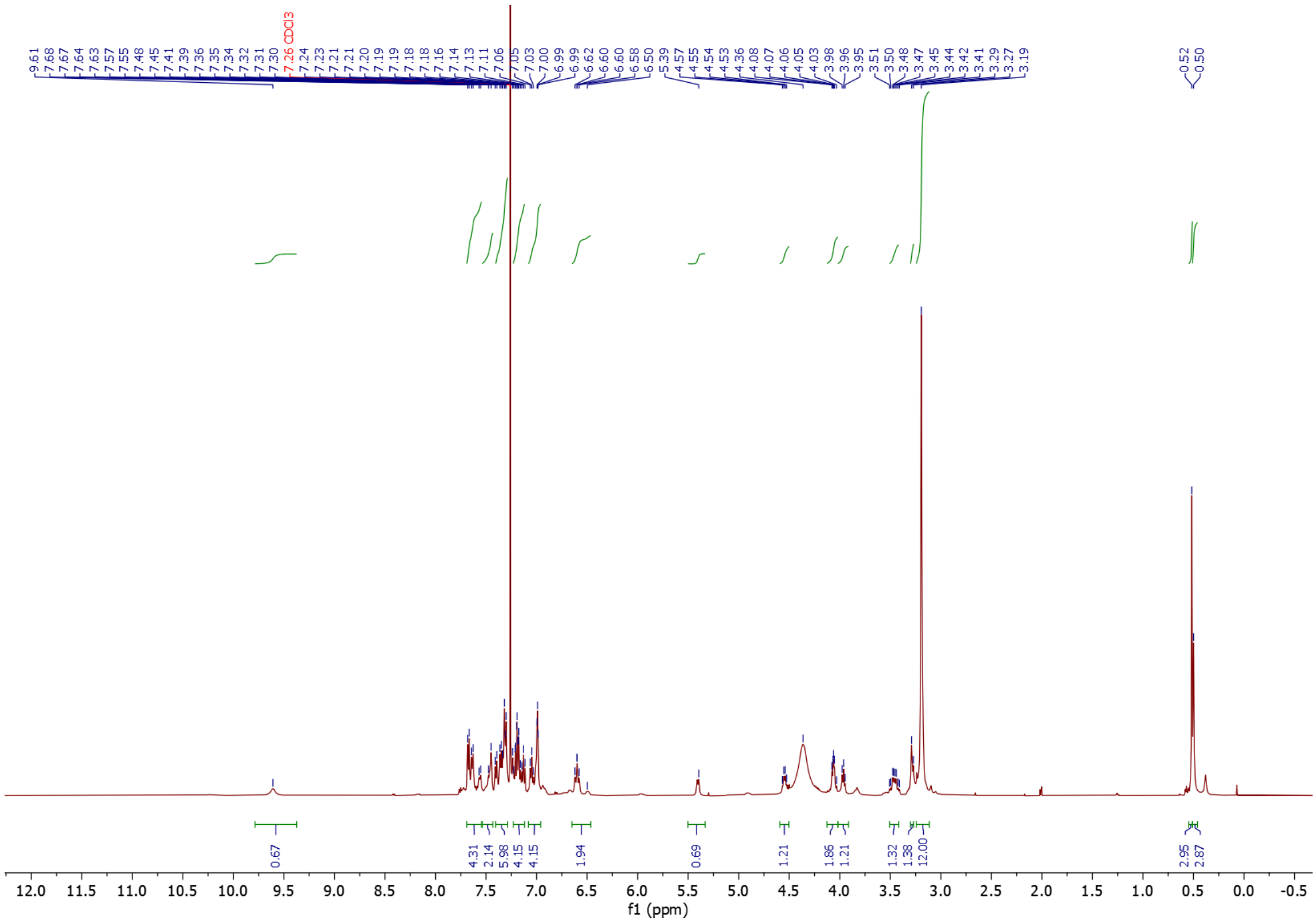

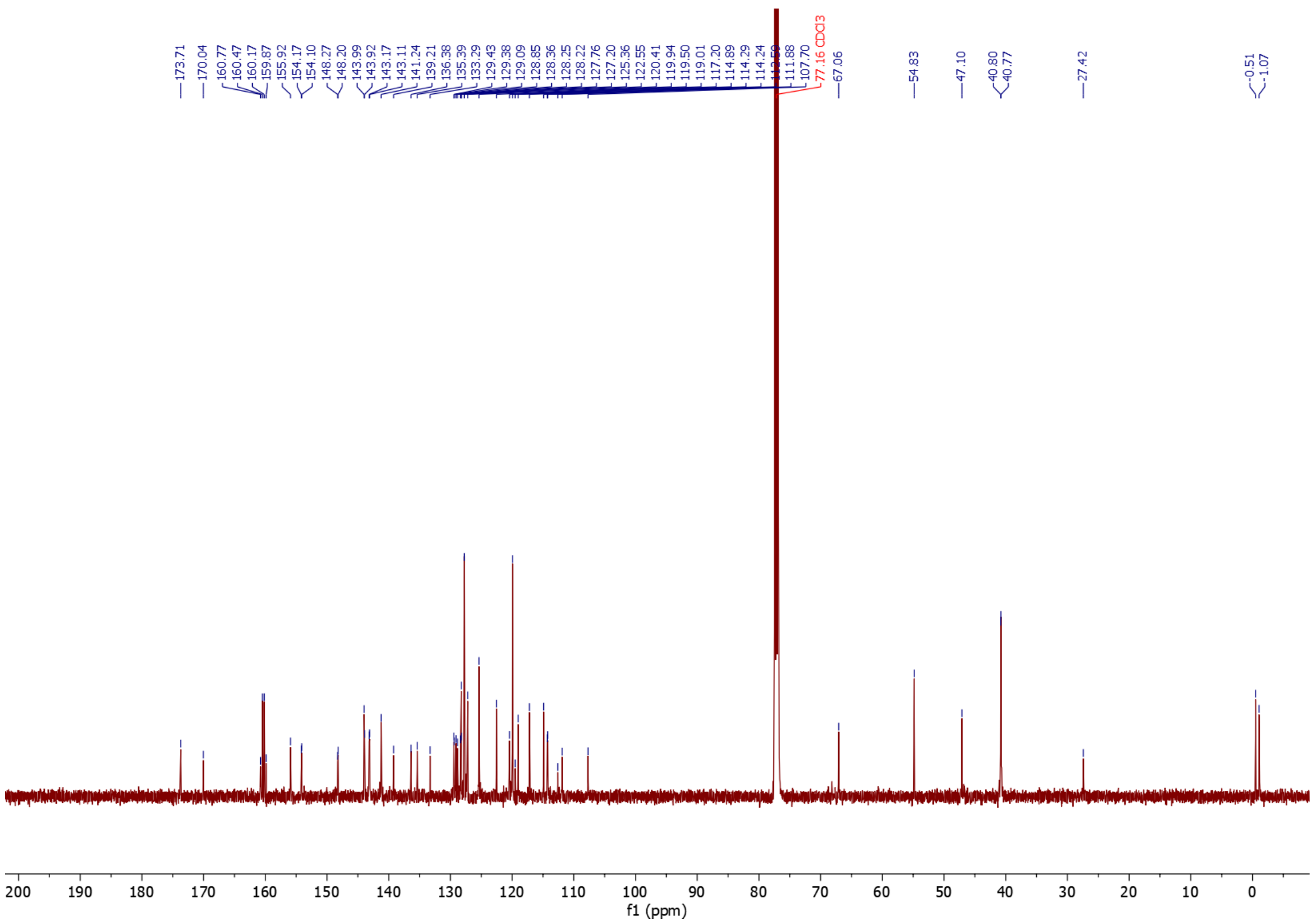

Compound 30 ($CDCl_3$)

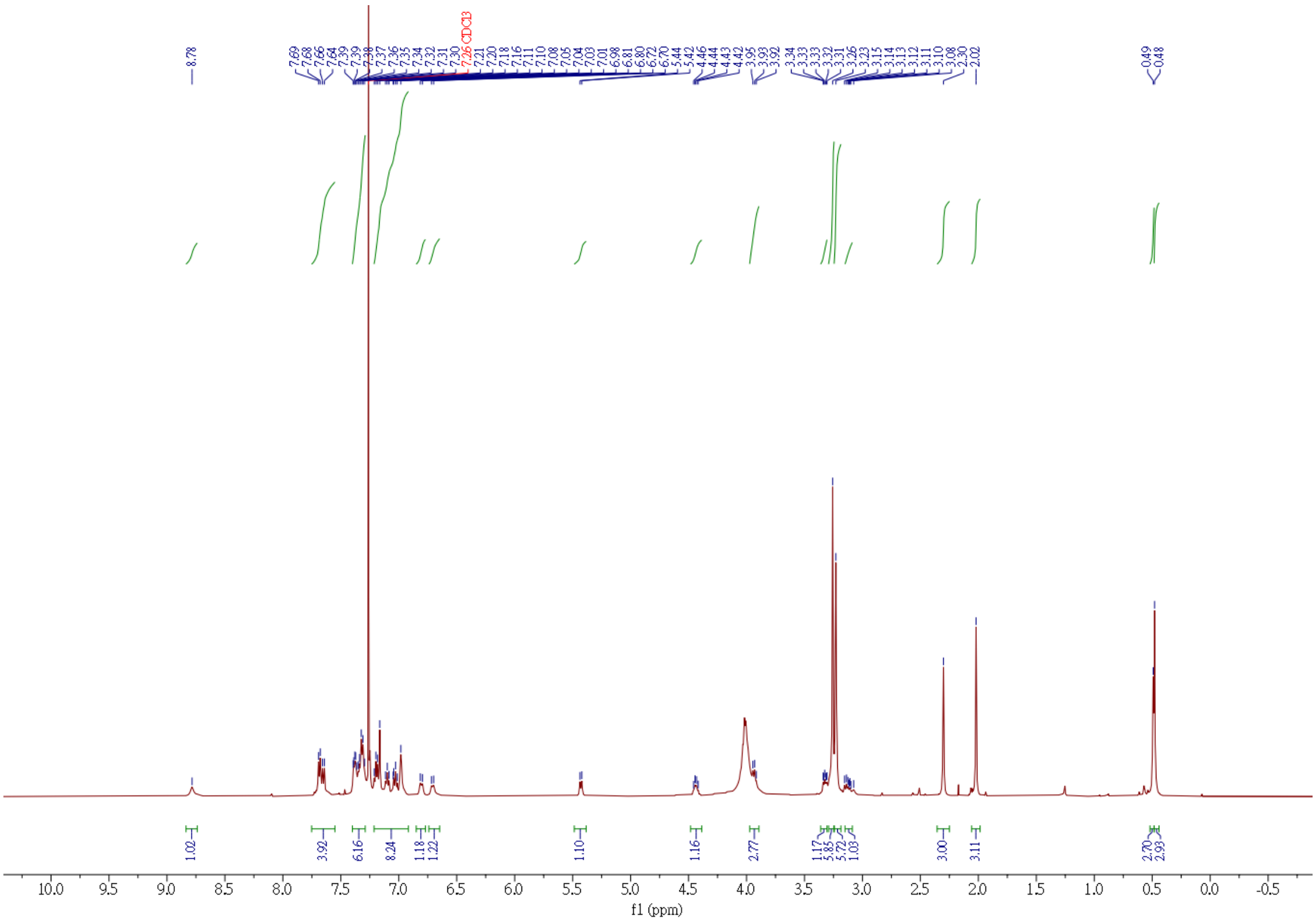

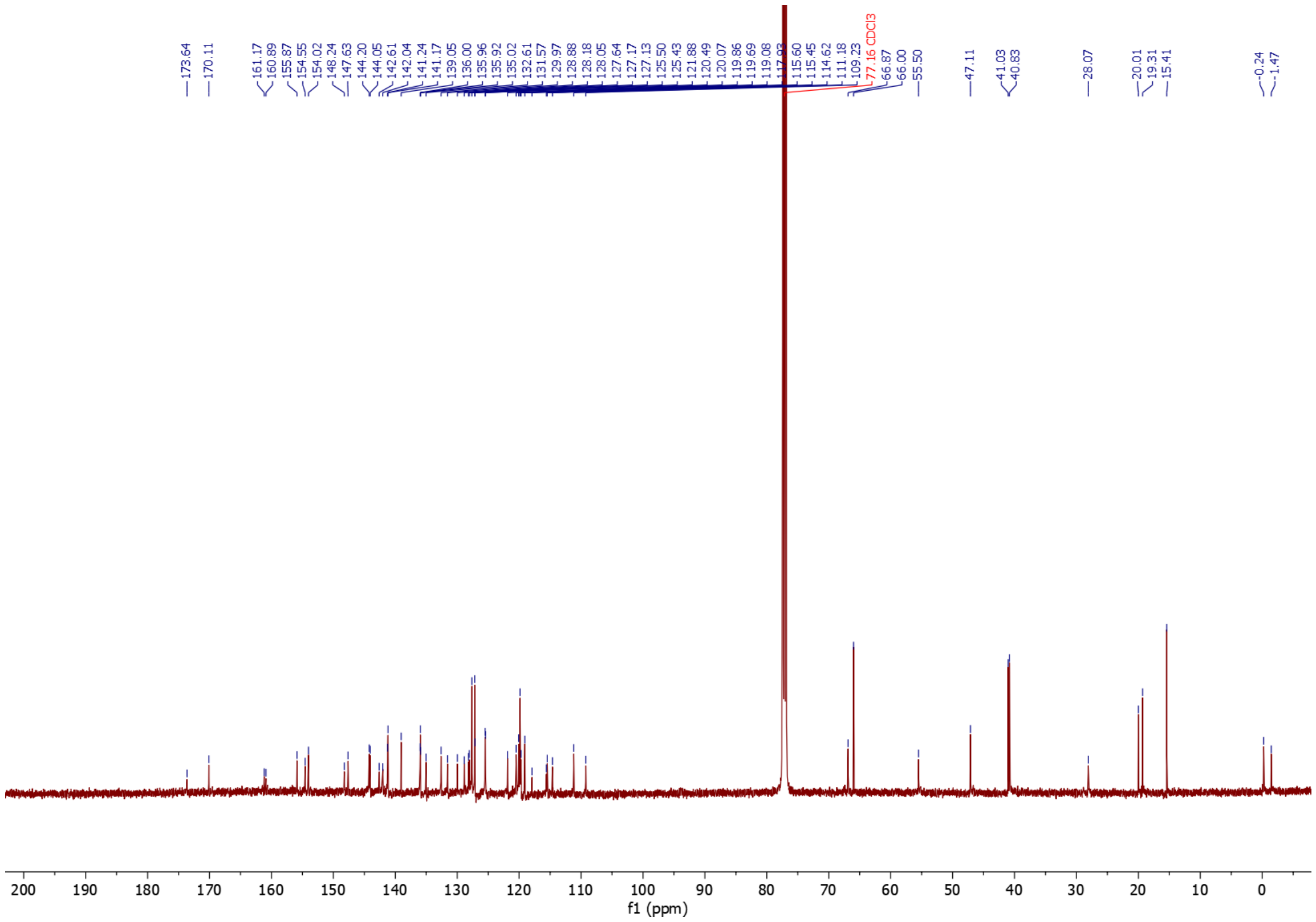

6. Supplementary References

1. Frisch, M.J. et al. (Gaussian, Inc., Wallingford CT., 2016).

2. Zhao, Y. & Truhlar, D.G. The M06 suite of density functionals for main group thermochemistry, thermochemical kinetics, noncovalent interactions, excited states, and transition elements: two new functionals and systematic testing of four M06-class functionals and 12 other functionals. *Theor. Chem. Acc.* **120**, 215-241 (2008).

3. Weigend, F. & Ahlrichs, R. Balanced basis sets of split valence, triple zeta valence and quadruple zeta valence quality for H to Rn: Design and assessment of accuracy. *Phys. Chem. Chem. Phys.* **7**, 3297-3305 (2005).

4. Marenich, A.V., Cramer, C.J. & Truhlar, D.G. Universal solvation model based on solute electron density and on a continuum model of the solvent defined by the bulk dielectric constant and atomic surface tensions. *J Phys. Chem. B* **113**, 6378-6396 (2009).

5. Caricato, M. et al. Formation and relaxation of excited states in solution: A new time dependent polarizable continuum model based on time dependent density functional theory. *J. Chem. Phys.* **124**, 124520 (2006).

6. Le Bahers, T., Adamo, C. & Ciofini, I. A Qualitative Index of Spatial Extent in Charge-Transfer Excitations. *J. Chem. Theory Computat.* **7**, 2498-2506 (2011).

7. Peach, M.J.G. et al. Excitation energies in density functional theory: An evaluation and a diagnostic test. *J. Chem. Phys.* **128**, 044118 (2008).

8. Chi, W. et al. A General Descriptor ΔE Enables the Quantitative Development of Luminescent Materials Based on Photoinduced Electron Transfer. *J. Am. Chem. Soc.* **142**, 6777-6785 (2020).

9. Guido, C.A. et al. D. Simple Protocol for Capturing Both Linear-Response and State-Specific Effects in Excited-State Calculations with Continuum Solvation Models. *J. Chem. Theor. Comput.* **17**, 5155-5164 (2021).

10. Sepali, C., Lafiosca, P., Goletto, L., Giovannini, T. & Cappelli, C. Vertical excitation energies of embedded systems: the vertical excitation model (VEM) within polarizable QM/MM. Preprint at https://arxiv.org/abs/2511.02429 (2025).

11. Hanwell, M.D. et al. Avogadro: an advanced semantic chemical editor, visualization, and analysis platform. *J. Cheminformatics* **4**, 17 (2012).

12. Lu, T. & Chen, F. Multiwfn: A multifunctional wavefunction analyzer. *J. Comput. Chem.* **33**, 580-592 (2012).

13. Humphrey, W., Dalke, A. & Schulten, K. VMD: Visual molecular dynamics. *J. Mol. Graphics* **14**, 33-38 (1996).
