# Supplementary material for: A general strategy towards multicolour fluorogenic peptides for wash-free bioassays
Source: Nat Chem. 2026 Aug 26;18(11):1968–78. doi: 10.1038/s41557-026-02228-0 (PMC13719488; doi:10.1038/s41557-026-02228-0)
Supplement: Supplementary file 2 [file 41557_2026_2228_MOESM2_ESM.pdf]

Corresponding author(s): Marc Vendrell
Last updated by author(s): May 26, 2026

Reporting Summary

Nature Portfolio wishes to improve the reproducibility of the work that we publish. This form provides structure for consistency and transparency in reporting. For further information on Nature Portfolio policies, see our Editorial Policies and the Editorial Policy Checklist.

Statistics

For all statistical analyses, confirm that the following items are present in the figure legend, table legend, main text, or Methods section.

n/a	Confirmed	
☐	☒	The exact sample size (*n*) for each experimental group/condition, given as a discrete number and unit of measurement
☐	☒	A statement on whether measurements were taken from distinct samples or whether the same sample was measured repeatedly
☐	☒	The statistical test(s) used AND whether they are one- or two-sided<br>*Only common tests should be described solely by name; describe more complex techniques in the Methods section.*
☒	☐	A description of all covariates tested
☒	☐	A description of any assumptions or corrections, such as tests of normality and adjustment for multiple comparisons
☐	☒	A full description of the statistical parameters including central tendency (e.g. means) or other basic estimates (e.g. regression coefficient) AND variation (e.g. standard deviation) or associated estimates of uncertainty (e.g. confidence intervals)
☐	☒	For null hypothesis testing, the test statistic (e.g. *F*, *t*, *r*) with confidence intervals, effect sizes, degrees of freedom and *P* value noted<br>*Give P values as exact values whenever suitable.*
☒	☐	For Bayesian analysis, information on the choice of priors and Markov chain Monte Carlo settings
☒	☐	For hierarchical and complex designs, identification of the appropriate level for tests and full reporting of outcomes
☒	☐	Estimates of effect sizes (e.g. Cohen's *d*, Pearson's *r*), indicating how they were calculated

Our web collection on statistics for biologists contains articles on many of the points above.

Software and code

Policy information about availability of computer code

Data collection	Standard commercial softwares were used for data collection. Specifically, HPLC-MS: OpenLab CDS; Leica Stellaris Falcon: Leica Application Suite X; Synergy HT spectrophotometer: Gen 5; Flow cytometry (Novocyte 3 Lasers): NovoExpress Software; Biacore 8K SPR instrument (Cytiva): Biacore Insight Evaluation Software. Quantum-chemical calculation data were collected using Gaussian 16 Rev. A.01.
Data analysis	Data analysis, including statistical analysis, was performed using Graphpad Prism 10.0. FCS express 7 was used to analyze Flow cytometry data. Fluorescence image analysis was performed with ImageJ or Leica Application Suite X software. SPR data analysis was performed with Biacore Insight Evaluation Software. For the computational study, molecular structures and frontier molecular orbitals were visualized using Avogadro; electron–hole distributions and charge-transfer characteristics were analyzed using Multiwfn and visualized using IQmol and VMD. Computational graphics were plotted using MatLab 2025b and finalized using CorelDraw 2025.

For manuscripts utilizing custom algorithms or software that are central to the research but not yet described in published literature, software must be made available to editors and reviewers. We strongly encourage code deposition in a community repository (e.g. GitHub). See the Nature Portfolio guidelines for submitting code & software for further information.

Data

Policy information about availability of data

All manuscripts must include a data availability statement. This statement should provide the following information, where applicable:

- Accession codes, unique identifiers, or web links for publicly available datasets
- A description of any restrictions on data availability
- For clinical datasets or third party data, please ensure that the statement adheres to our policy

Data from Figures 1–5 and Supplementary Figures 1–52 of this study are available from the corresponding authors upon reasonable request.

Research involving human participants, their data, or biological material

Policy information about studies with human participants or human data. See also policy information about sex, gender (identity/presentation), and sexual orientation and race, ethnicity and racism.

Reporting on sex and gender	Information on donors was limited to age and gender and blinded for analysis of experiments.
Reporting on race, ethnicity, or other socially relevant groupings	Information on donors was limited to age and gender and blinded for analysis of experiments.
Population characteristics	Healthy male and female volunteers were used for cell isolation in this study (age range: 20-60 years). All donor samples were anonymized following blood sample collection.
Recruitment	Participants for the T cell isolation from blood were randomly recruited according to their availability (based on sample volume and frequency of donation), according to local ethical guidelines of the blood donor register. No potential self-selection bias was expected.
Ethics oversight	Whole blood was collected from healthy volunteers following informed consent and ethical approval (25-EMREC-023) at the University of Edinburgh.

Note that full information on the approval of the study protocol must also be provided in the manuscript.

Field-specific reporting

Please select the one below that is the best fit for your research. If you are not sure, read the appropriate sections before making your selection.

☒ Life sciences ☐ Behavioural & social sciences ☐ Ecological, evolutionary & environmental sciences

For a reference copy of the document with all sections, see nature.com/documents/nr-reporting-summary-flat.pdf

Life sciences study design

All studies must disclose on these points even when the disclosure is negative.

Sample size	Sample/replicate size: All experiments contained at least 3 technical repeats, in line with current standards in the field. Numbers for original data of the manuscript were based on previous experience. Sample size is clearly stated in figure legends and/or experimental procedures.
Data exclusions	N/A
Replication	At least 3 independent experiments were performed in order to check the reproducibility of results and all attempts at replication were successful.
Randomization	No randomization method was used for experiments. Cells from human peripheral blood have been allocated a specific donor coding system to protect donor anonymity and allow exact recording of experimental use without biases.
Blinding	N/A

Reporting for specific materials, systems and methods

We require information from authors about some types of materials, experimental systems and methods used in many studies. Here, indicate whether each material, system or method listed is relevant to your study. If you are not sure if a list item applies to your research, read the appropriate section before selecting a response.

Materials & experimental systems

n/a	Involved in the study
☐	☒ Antibodies
☒	☐ Eukaryotic cell lines
☒	☐ Palaeontology and archaeology
☒	☐ Animals and other organisms
☒	☐ Clinical data
☒	☐ Dual use research of concern
☒	☐ Plants

Methods

n/a	Involved in the study
☒	☐ ChIP-seq
☐	☒ Flow cytometry
☒	☐ MRI-based neuroimaging

Antibodies

Antibodies used

Anti-human PD-1 antibody (Biolegend, clone: EH12.2H7, Cat: 329936).
Anti-human CD3 and CD28 antibodies covalently bound to magnetic beads (Dynabeads Human T-Activator CD3/CD28, ThermoFisher Scientific, Cat: 11131D).

Validation

Validation was performed as indicated in the manufacturer's website. Antibodies have been rigorously tested and validated using IHC, Western blotting and flow cytometry.

Plants

Seed stocks

Report on the source of all seed stocks or other plant material used. If applicable, state the seed stock centre and catalogue number. If plant specimens were collected from the field, describe the collection location, date and sampling procedures.

Novel plant genotypes

Describe the methods by which all novel plant genotypes were produced. This includes those generated by transgenic approaches, gene editing, chemical/radiation-based mutagenesis and hybridization. For transgenic lines, describe the transformation method, the number of independent lines analyzed and the generation upon which experiments were performed. For gene-edited lines, describe the editor used, the endogenous sequence targeted for editing, the targeting guide RNA sequence (if applicable) and how the editor was applied.

Authentication

Describe any authentication procedures for each seed stock used or novel genotype generated. Describe any experiments used to assess the effect of a mutation and, where applicable, how potential secondary effects (e.g. second site T-DNA insertions, mosiacism, off-target gene editing) were examined.

Flow Cytometry

Plots

Confirm that:

☒ The axis labels state the marker and fluorochrome used (e.g. CD4-FITC).

☒ The axis scales are clearly visible. Include numbers along axes only for bottom left plot of group (a 'group' is an analysis of identical markers).

☒ All plots are contour plots with outliers or pseudocolor plots.

☒ A numerical value for number of cells or percentage (with statistics) is provided.

Methodology

Sample preparation

Sample preparation was performed as described in detail in the Supplementary Information. T cells were isolated from the peripheral blood by immunomagnetic negative selection using easySepTM Direct Human T cell Isolation Kit (Stemcell technologies). Isolated T cells were plated at 100,000 cells/well on a 96-well flat-bottomed plate and cultured for 72 h in DMEM containing 10% fetal bovine serum, 2-mercaptoethanol, L-glutamine and Pen-Strep at 37 °C and 5% CO2. For activation, T cells were stimulated using DynabeadsTM human T-activator CD3/CD28 for 72 hours (cell to bead ratio 1:1). Cells were harvested after 72 hours and beads were separated using an Easysep magnet (Stemcell technologies).
When indicated, cells were stained with anti PD-1/Zombie Violet for 20 min at 4°C or PD-1 binding peptides for 15-30 min at r.t. For flow cytometry analysis, the cells were washed with PBS and resuspended in FACS buffer (PBS + 0.1% BSA).

Instrument

Novocyte (Agilent)

Software

Data collection and analysis was performed with FCS express 7 software.

Cell population abundance

The T cells were isolated from peripheral blood using Stemcell EasysepTM Direct human T cell isolation kit, up to 97% purity according to Manufacturer's protocol (StemCell, Cat- 19661).

Gating strategy

Cells were gated first based on the forward and side scattering eliminating all debris, and singlets were gated next based on

Gating strategy

forward scatter area versus height. Live cells were selected from the singlets based on live-dead staining (Zombie Violet, 1:100). Cells were assessed via staining with anti-human PD-1 (Alexa Fluor 488).

☒ Tick this box to confirm that a figure exemplifying the gating strategy is provided in the Supplementary Information.
