# Supplementary material for: A general strategy towards multicolour fluorogenic peptides for wash-free bioassays
Source: Nat Chem. 2026 Aug 26;18(11):1968–78. doi: 10.1038/s41557-026-02228-0 (PMC13719488; doi:10.1038/s41557-026-02228-0)
Supplement: Supplementary file 3 [file 41557_2026_2228_MOESM6_ESM.pdf]

Charge, Spin, and Coordinates of Modelled Compounds in Water and Toluene

(Ground and Excited States)

Table of Contents

Coumarin-AA (**10**) S_0min in water @ M06-2X/def2-SVP, SMD(water) 5

Coumarin-AA (**10**) S_1min(LE) in water @ M06-2X/def2-SVP, SMD(water) 7

Coumarin-AA (**10**) S_1min(TICT) in water @ M06-2X/def2-SVP, SMD(water) 9

BODIPY-AA (**11**) S_0min in water @ M06-2X/def2-SVP, SMD(water) 11

BODIPY-AA (**11**) S_1min(LE) in water @ M06-2X/def2-SVP, SMD(water) 14

BODIPY-AA (**11**) S_1min(ET) in water @ M06-2X/def2-SVP, SMD(water) 17

HPF-AA(OH) (**12**) S_0min in water @ M06-2X/def2-SVP, SMD(water) 20

HPF-AA(OH) (**12**) S_1min(LE) in water @ M06-2X/def2-SVP, SMD(water) 22

HPF-AA(OH) (**12**) S_1min(ET) in water @ M06-2X/def2-SVP, SMD(water) 24

HPF-AA(O^-) (**12**) S_0min in water @ M06-2X/6-31G(d,p), SMD(water) 26

HPF-AA(O^-) (**12**) S_1min(LE) in water @ M06-2X/6-31G(d,p), SMD(water) 28

HPF-AA(O^-) (**12**) S_1min(ET) in water @ M06-2X/6-31G(d,p), SMD(water) 30

O-RDM-AA (**13**) S_0min in water @ M06-2X/def2-SVP, SMD(water) 32

O-RDM-AA (**13**) S_1min(LE) in water @ M06-2X/def2-SVP, SMD(water) 35

O-RDM-AA (**13**) S_1min(ET) in water @ M06-2X/def2-SVP, SMD(water) 38

Oxazone-AA (**14**) S_0min in water @ M06-2X/def2-SVP, SMD(water) 41

Oxazone-AA (**14**) S_1min(LE) in water @ M06-2X/def2-SVP, SMD(water) 43

Oxazone-AA (**14**) S_1min(TICT) in water @ ωB97X-D/def2-SVP, SMD(water) ... 45

Si-RDM-AA (**15**) S_0min in water @ M06-2X/def2-SVP, SMD(water) 47

Si-RDM-AA (**15**) S_1min(LE) in water @ M06-2X/def2-SVP, SMD(water) 50

Si-RDM-AA (**15**) S_1min(ET) in water @ M06-2X/def2-SVP, SMD(water) 53

Si-RDM-AA (**28**) S_0min in water @ M06-2X/def2-SVP, SMD(water) 56

Si-RDM-AA (**28**) S_1min(LE) in water @ M06-2X/def2-SVP, SMD(water) 59

Si-RDM-AA (**28**) S_1min(ET) in water @ M06-2X/def2-SVP, SMD(water) 62

Si-RDM-AA (**29**) S_0min in water @ M06-2X/def2-SVP, SMD(water) 65

Si-RDM-AA (**29**) S_1min(LE) in water @ M06-2X/def2-SVP, SMD(water) 68

Si-RDM-AA (**29**) S_1min(ET) in water @ M06-2X/def2-SVP, SMD(water) 71

Si-RDM-AA (**30**) S_0min in water @ M06-2X/def2-SVP, SMD(water) 74

Si-RDM-AA (**30**) S_1min(LE) in water @ M06-2X/def2-SVP, SMD(water) 77

Si-RDM-AA (**30**) S_1min(ET) in water @ M06-2X/def2-SVP, SMD(water) 80

Carbopyronine-AA S_0min in water @ M06-2X/def2-SVP, SMD(water) 83

Carbopyronine-AA S_1min(LE) in water @ M06-2X/def2-SVP, SMD(water) 86

Carbopyronine-AA S_1min(ET) in water @ M06-2X/def2-SVP, SMD(water)....... 89
Phenyl-Cy3-AA S_0min in water @ M06-2X/def2-SVP, SMD(water) 92
Phenyl-Cy3-AA S_1min(LE) in water @ M06-2X/def2-SVP, SMD(water)............ 95
Phenyl-Cy3-AA S_1min(ET) in water @ M06-2X/def2-SVP, SMD(water) 98
Phenyl-Cy5-AA S_0min in water @ M06-2X/def2-SVP, SMD(water) 101
Phenyl-Cy5-AA S_1min(LE) in water @ M06-2X/def2-SVP, SMD(water).......... 104
Phenyl-Cy5-AA S_1min(ET) in water @ M06-2X/def2-SVP, SMD(water) 107
BODIPY-AA (**11**) S_0min in toluene @ M06-2X/def2-SVP, SMD(toluene)..........110
BODIPY-AA (**11**) S_1min(LE) in toluene @ M06-2X/def2-SVP, SMD(toluene)...113
BODIPY-AA (**11**) S_1min(ET) in toluene @ M06-2X/def2-SVP, SMD(toluene)...116
HPF-AA(OH) (**12**) S_0min in toluene @ M06-2X/def2-SVP, SMD(toluene)........119
HPF-AA(OH) (**12**) S_1min(LE) in toluene @ M06-2X/def2-SVP, SMD(toluene) 121
HPF-AA(OH) (**12**) S_1min(ET) in toluene @ M06-2X/def2-SVP, SMD(toluene) 123
O-RDM-AA (**13**) S_0min in toluene @ M06-2X/def2-SVP, SMD(toluene) 125
O-RDM-AA (**13**) S_1min(LE) in toluene @ M06-2X/def2-SVP, SMD(toluene)... 128
O-RDM-AA (**13**) S_1min(ET) in toluene @ M06-2X/def2-SVP, SMD(toluene)... 131
Si-RDM-AA (**15**) S_0min in toluene @ M06-2X/def2-SVP, SMD(toluene) 134
Si-RDM-AA (**15**) S_1min(LE) in toluene @ M06-2X/def2-SVP, SMD(toluene)... 137
Si-RDM-AA (**15**) S_1min(ET) in toluene @ M06-2X/def2-SVP, SMD(toluene) .. 140
Si-RDM-AA (**28**) S_0min in toluene @ M06-2X/def2-SVP, SMD(toluene) 143
Si-RDM-AA (**28**) S_1min(LE) in toluene @ M06-2X/def2-SVP, SMD(toluene)... 146
Si-RDM-AA (**28**) S_1min(ET) in toluene @ M06-2X/def2-SVP, SMD(toluene) .. 149
Si-RDM-AA (**29**) S_0min in toluene @ M06-2X/def2-SVP, SMD(toluene) 152
Si-RDM-AA (**29**) S_1min(LE) in toluene @ M06-2X/def2-SVP, SMD(toluene)... 155
Si-RDM-AA (**29**) S_1min(ET) in toluene @ M06-2X/def2-SVP, SMD(toluene) .. 158
Si-RDM-AA (**30**) S_0min in toluene @ M06-2X/def2-SVP, SMD(toluene) 161
Si-RDM-AA (**30**) S_1min(LE) in toluene @ M06-2X/def2-SVP, SMD(toluene)... 164
Si-RDM-AA (**30**) S_1min(ET) in toluene @ M06-2X/def2-SVP, SMD(toluene) .. 167
C-RDM-AA S_0min in toluene @ M06-2X/def2-SVP, SMD(toluene)................. 170
C-RDM-AA S_1min(LE) in toluene @ M06-2X/def2-SVP, SMD(toluene) 173
C-RDM-AA S_1min(ET) in toluene @ M06-2X/def2-SVP, SMD(toluene) 176
Cy3 -AA S_0min in toluene @ M06-2X/def2-SVP, SMD(toluene) 179
Cy3-AA S_1min(LE) in toluene @ M06-2X/def2-SVP, SMD(toluene)................ 182

Cy3-AA S_1min(ET) in toluene @ M06-2X/def2-SVP, SMD(toluene) 185
Cy5 -AA S_0min in toluene @ M06-2X/def2-SVP, SMD(toluene) 188
Cy5-AA S_1min(LE) in toluene @ M06-2X/def2-SVP, SMD(toluene)................ 191
Cy5-AA S_1min(ET) in toluene @ M06-2X/def2-SVP, SMD(toluene) 194

Coumarin-AA (**10**) S_0min in water @ M06-2X/def2-SVP, SMD(water)

0 1

C	-3.403864	-1.607198	0.064013
C	-3.923938	-0.310749	-0.237481
C	-2.988764	0.738620	-0.376994
C	-1.630252	0.481873	-0.255172
C	-1.106908	-0.787278	0.025502
C	-2.049394	-1.826211	0.184229
O	-0.795183	1.549027	-0.416385
C	0.564554	1.453058	-0.357864
C	1.136912	0.141395	-0.056326
C	0.323430	-0.946360	0.145319
O	1.187612	2.472820	-0.559748
C	0.864803	-2.296988	0.503178
N	-5.266776	-0.090608	-0.397722
C	-6.219551	-1.091117	0.075632
C	-5.713771	1.302451	-0.494056
C	-7.203644	1.480099	-0.718967
C	-6.382334	-1.097408	1.589549
C	2.608763	0.077063	-0.013278
C	3.489676	0.803789	0.760332
C	4.807709	0.362310	0.389278
C	4.653080	-0.634790	-0.610566
N	3.312359	-0.781711	-0.837263
C	6.103614	0.714270	0.814105
C	7.193166	0.076606	0.240029
C	7.017509	-0.912785	-0.753992
C	5.753913	-1.282135	-1.191330
C	3.154495	1.845459	1.779063

H	-4.074103	-2.450937	0.207287
H	-3.283040	1.763817	-0.583827
H	-1.701510	-2.835015	0.409200
H	0.315937	-2.704973	1.363072
H	0.721876	-2.998168	-0.333568
H	1.930217	-2.260516	0.751800
H	-7.183082	-0.896730	-0.407464
H	-5.911296	-2.078691	-0.287937
H	-5.407068	1.857299	0.412056
H	-5.184348	1.769631	-1.336575
H	-7.403965	2.550854	-0.858970
H	-7.541125	0.951713	-1.621807
H	-7.803047	1.136671	0.135105
H	-7.113110	-1.859940	1.894071
H	-5.427331	-1.317809	2.089456
H	-6.738057	-0.118957	1.945995
H	2.887887	-1.425772	-1.496069
H	6.244020	1.479247	1.580946
H	8.203751	0.339361	0.556843
H	7.895009	-1.396343	-1.186681
H	5.616178	-2.045940	-1.957885
H	2.095089	1.792985	2.066557
H	3.760106	1.714715	2.688142
H	3.347269	2.861132	1.398975

Coumarin-AA (**10**) S_1min(LE) in water @ M06-2X/def2-SVP, SMD(water)

0 1

C	-3.383407	-1.607174	0.013334
C	-3.906204	-0.294971	-0.224015
C	-2.964135	0.779838	-0.250409
C	-1.623417	0.544217	-0.079474
C	-1.073980	-0.759475	0.138402
C	-2.037267	-1.820496	0.184604
O	-0.808257	1.644688	-0.078254
C	0.575897	1.544473	-0.089458
C	1.156026	0.245644	0.145941
C	0.309356	-0.919307	0.327255
O	1.153011	2.587427	-0.345820
C	0.856675	-2.233907	0.800773
N	-5.232447	-0.065880	-0.418523
C	-6.202205	-1.132027	-0.189620
C	-5.700696	1.323528	-0.491485
C	-7.181086	1.491690	-0.772796
C	-6.515874	-1.339578	1.287195
C	2.578624	0.146410	0.049700
C	3.603914	1.030492	0.487548
C	4.838690	0.417004	0.148349
C	4.547362	-0.830904	-0.476388
N	3.187888	-0.967773	-0.504094
C	6.189586	0.796940	0.331210
C	7.186692	-0.050824	-0.120872
C	6.871894	-1.277015	-0.751547
C	5.554452	-1.684440	-0.936579
C	3.467823	2.280026	1.289995

H	-4.049583	-2.465576	0.062976
H	-3.272207	1.813111	-0.388896
H	-1.687128	-2.840065	0.346879
H	0.146635	-2.723863	1.482689
H	1.049864	-2.947918	-0.020456
H	1.799698	-2.098307	1.345956
H	-7.115726	-0.884532	-0.739361
H	-5.826065	-2.056928	-0.642995
H	-5.442812	1.837542	0.451878
H	-5.135009	1.830982	-1.286262
H	-7.390113	2.567682	-0.840067
H	-7.469272	1.031667	-1.728029
H	-7.810388	1.076952	0.026122
H	-7.251073	-2.147772	1.405651
H	-5.609074	-1.609957	1.847699
H	-6.934711	-0.422890	1.727775
H	2.690585	-1.732613	-0.946751
H	6.434584	1.746033	0.812030
H	8.234027	0.226905	0.006676
H	7.682101	-1.920826	-1.097973
H	5.311961	-2.633444	-1.415937
H	2.528657	2.286549	1.859503
H	4.308107	2.364505	1.994637
H	3.468509	3.180551	0.657197

Coumarin-AA (**10**) S_1min(TICT) in water @ M06-2X/def2-SVP, SMD(water)

0 1

C	-3.375756	-1.522215	-0.593656
C	-3.914657	-0.233393	-0.389237
C	-2.984061	0.812736	-0.182883
C	-1.618363	0.577414	-0.220716
C	-1.062339	-0.701924	-0.446460
C	-2.002091	-1.740702	-0.621655
O	-0.811996	1.679494	-0.006838
C	0.575794	1.576300	0.015499
C	1.132606	0.311471	-0.245800
C	0.362360	-0.868783	-0.469804
O	1.179566	2.627704	0.267766
C	0.970430	-2.218158	-0.687628
N	-5.293257	0.011693	-0.413811
C	-6.172854	-1.103332	-0.074085
C	-5.717501	1.328084	0.060851
C	-7.214001	1.573433	-0.019667
C	-6.138349	-1.491020	1.399329
C	2.581322	0.243964	-0.155455
C	3.342040	-0.067486	1.036584
C	4.706670	-0.118944	0.640564
C	4.745483	0.157189	-0.749519
N	3.429884	0.362771	-1.177950
C	5.902911	-0.362981	1.340743
C	7.095650	-0.324851	0.630985
C	7.104536	-0.049037	-0.747523
C	5.928014	0.199577	-1.465547

C	2.728374	-0.281795	2.355700
H	-4.030242	-2.379232	-0.738624
H	-3.295849	1.836242	0.010511
H	-1.638622	-2.756821	-0.784937
H	0.676879	-2.937279	0.098614
H	0.652421	-2.666970	-1.645572
H	2.068982	-2.176911	-0.696612
H	-7.194270	-0.831767	-0.366079
H	-5.918449	-1.965045	-0.703114
H	-5.370156	1.503714	1.098768
H	-5.219559	2.086431	-0.559163
H	-7.410859	2.627412	0.219223
H	-7.595486	1.377344	-1.032371
H	-7.782336	0.959378	0.692271
H	-6.816350	-2.334538	1.593095
H	-5.124829	-1.790110	1.706205
H	-6.453614	-0.649112	2.034609
H	3.148864	0.561788	-2.136474
H	5.884427	-0.574190	2.410566
H	8.039179	-0.510674	1.143634
H	8.057365	-0.027171	-1.277772
H	5.944022	0.414045	-2.533463
H	2.075128	-1.171522	2.318487
H	3.479969	-0.415391	3.141155
H	2.069115	0.568266	2.598232

BODIPY-AA (**11**) S_0min in water @ M06-2X/def2-SVP, SMD(water)

0 1

C	2.035874	1.005677	0.689192
N	3.255996	1.426370	0.162301
B	4.264826	0.535034	-0.604878
N	3.598038	-0.854912	-0.760489
C	2.364683	-1.228749	-0.229027
C	1.594714	-0.311292	0.497864
C	1.453881	2.108653	1.390192
C	2.351116	3.163791	1.251267
C	3.449919	2.712187	0.491537
C	4.110154	-1.892526	-1.438180
C	3.211710	-2.977842	-1.367510
C	2.109230	-2.582222	-0.616284
F	4.542675	1.082316	-1.857387
F	5.456718	0.423066	0.116467
C	0.288835	-0.734308	1.069935
C	-0.896943	-0.344184	0.448117
C	-2.139769	-0.719011	0.979967
C	-2.167257	-1.489462	2.153474
C	-0.980562	-1.883824	2.767992
C	0.250785	-1.512502	2.230846
C	-3.383683	-0.284438	0.330208
N	-3.433567	0.919167	-0.348700
C	-4.685596	1.101884	-0.866829
C	-5.458752	-0.036372	-0.517927
C	-5.217981	2.163531	-1.613210
C	-6.541815	2.062020	-2.014530
C	-7.326679	0.933305	-1.685646

C	-6.799368	-0.112844	-0.943992
C	0.157824	2.181233	2.135491
C	0.921138	-3.441235	-0.317082
C	4.658665	3.475961	0.075289
C	5.418018	-1.824831	-2.147127
H	2.239475	4.166656	1.657986
H	3.364310	-3.949763	-1.831968
H	-0.846403	0.254538	-0.464217
H	-3.123873	-1.756756	2.603160
H	-1.016744	-2.477086	3.682654
H	1.182307	-1.816634	2.711828
H	-2.673961	1.590473	-0.393550
H	-4.609079	3.032101	-1.867619
H	-6.986946	2.870055	-2.597543
H	-8.364011	0.890326	-2.021312
H	-7.411343	-0.980838	-0.689303
H	0.019313	1.323243	2.807726
H	0.130007	3.103425	2.730084
H	-0.700783	2.195136	1.447167
H	0.966653	-4.354141	-0.924654
H	0.895465	-3.736709	0.741878
H	-0.024070	-2.922908	-0.530599
H	4.658008	4.467270	0.541975
H	5.573440	2.939603	0.364465
H	4.679414	3.597084	-1.017710
H	6.196083	-1.417523	-1.486568
H	5.715852	-2.823853	-2.484786
H	5.347234	-1.162313	-3.022427
C	-4.612533	-0.915100	0.245292

C	-5.014075	-2.257516	0.773479
H	-4.155625	-2.940298	0.839124
H	-5.463809	-2.192175	1.777044
H	-5.762052	-2.716798	0.111246

BODIPY-AA (**11**) S_1min(LE) in water @ M06-2X/def2-SVP, SMD(water)

0 1

C	1.976026	0.993991	0.563603
N	3.195270	1.427421	0.049103
B	4.284216	0.539045	-0.587429
N	3.694016	-0.883586	-0.662464
C	2.440734	-1.278626	-0.204002
C	1.573373	-0.362452	0.446638
C	1.332156	2.130241	1.193651
C	2.195649	3.201644	1.010627
C	3.337593	2.747892	0.307431
C	4.299368	-1.939480	-1.253316
C	3.430184	-3.055850	-1.195518
C	2.259167	-2.672669	-0.556125
F	4.609546	1.001083	-1.866208
F	5.444181	0.551043	0.196182
C	0.273721	-0.814014	0.997776
C	-0.924765	-0.381054	0.422962
C	-2.164050	-0.779456	0.944695
C	-2.185587	-1.633265	2.059414
C	-0.993295	-2.079628	2.626502
C	0.232905	-1.673524	2.104019
C	-3.411965	-0.284351	0.347086
N	-3.455878	0.968389	-0.236767
C	-4.714074	1.208163	-0.715380
C	-5.498024	0.056923	-0.440558
C	-5.244097	2.332108	-1.365901
C	-6.577263	2.281475	-1.746016
C	-7.373261	1.141999	-1.489431

C	-6.847679	0.033180	-0.843540
C	0.030272	2.183709	1.925189
C	1.064049	-3.540685	-0.332541
C	4.535563	3.516932	-0.101738
C	5.647039	-1.849357	-1.861475
H	2.045513	4.222872	1.356809
H	3.654163	-4.038748	-1.606678
H	-0.884280	0.273087	-0.451228
H	-3.138785	-1.924577	2.501558
H	-1.021904	-2.736142	3.497393
H	1.166582	-2.012905	2.557049
H	-2.687005	1.630716	-0.241164
H	-4.626610	3.209064	-1.564794
H	-7.021071	3.139061	-2.254500
H	-8.417696	1.140377	-1.805208
H	-7.468391	-0.843049	-0.644188
H	-0.084126	1.344486	2.626548
H	-0.029606	3.123798	2.489506
H	-0.833149	2.146478	1.240839
H	1.151759	-4.446607	-0.946918
H	0.970222	-3.853568	0.719236
H	0.128304	-3.026507	-0.596191
H	4.450592	4.558673	0.226316
H	5.446379	3.070800	0.329570
H	4.660103	3.488210	-1.196314
H	6.368129	-1.424946	-1.145172
H	5.987557	-2.840093	-2.182079
H	5.633509	-1.174119	-2.733015
C	-4.651501	-0.889669	0.236329

C	-5.063742	-2.263000	0.667937
H	-4.213391	-2.958877	0.669490
H	-5.498758	-2.268334	1.680118
H	-5.826745	-2.662381	-0.015722

BODIPY-AA (**11**) S_1min(ET) in water @ M06-2X/def2-SVP, SMD(water)

0 1

C	-2.214282	-1.122967	0.569063
N	-3.438328	-1.271191	-0.067620
B	-4.223001	-0.155288	-0.773920
N	-3.410439	1.142331	-0.647313
C	-2.188868	1.273977	-0.002434
C	-1.576329	0.147405	0.611808
C	-1.825574	-2.384578	1.080239
C	-2.861936	-3.283480	0.724902
C	-3.832583	-2.578929	0.025449
C	-3.778636	2.359071	-1.155454
C	-2.793736	3.285413	-0.839360
C	-1.775273	2.623914	-0.109162
F	-4.427986	-0.479614	-2.132956
F	-5.498934	0.000752	-0.189577
C	-0.261808	0.290437	1.277024
C	0.908872	0.100361	0.557790
C	2.179115	0.243398	1.168904
C	2.242394	0.587542	2.545801
C	1.078239	0.769946	3.265250
C	-0.171029	0.628114	2.640183
C	3.362843	0.022590	0.394734
N	3.331653	-0.609092	-0.809579
C	4.590311	-0.656206	-1.354589
C	5.478588	-0.000517	-0.455439
C	5.025864	-1.215362	-2.553140
C	6.381148	-1.095723	-2.841987
C	7.288439	-0.441172	-1.969650

C	6.855860	0.108893	-0.783315
C	-0.586960	-2.745637	1.844395
C	-0.531523	3.268351	0.425722
C	-5.117044	-3.067453	-0.558561
C	-5.050524	2.553260	-1.913208
H	-2.898139	-4.346504	0.958440
H	-2.808498	4.340333	-1.109114
H	0.831075	-0.134116	-0.505026
H	3.201502	0.669577	3.050479
H	1.129434	1.017128	4.325776
H	-1.087746	0.779597	3.213274
H	2.513070	-1.047519	-1.222612
H	4.335819	-1.715860	-3.231379
H	6.760019	-1.517565	-3.773894
H	8.339357	-0.376912	-2.250747
H	7.547110	0.612668	-0.106661
H	-0.473339	-2.156268	2.768175
H	-0.622251	-3.806472	2.128777
H	0.333171	-2.588001	1.258405
H	-0.540445	4.342259	0.193107
H	-0.438935	3.165051	1.518737
H	0.387149	2.840838	-0.007444
H	-5.228584	-4.142725	-0.371756
H	-5.980563	-2.544833	-0.120164
H	-5.154227	-2.894601	-1.644596
H	-5.924330	2.273504	-1.305868
H	-5.153100	3.604156	-2.210867
H	-5.075927	1.928837	-2.819055
C	4.726580	0.430597	0.654983

C	5.260520	1.195174	1.810198
H	4.627767	2.062340	2.043182
H	5.305507	0.563514	2.711390
H	6.276178	1.544887	1.592539

HPF-AA(OH) (**12**) S_0min in water @ M06-2X/def2-SVP, SMD(water)

0 1

C	2.936415	-1.133900	-0.121361
C	4.345916	-1.074803	-0.105607
O	4.995166	0.109175	-0.012608
C	4.331014	1.288273	0.075854
C	2.882611	1.291630	0.089124
C	2.193913	0.098351	-0.013749
C	5.077022	2.426507	0.157637
C	4.442379	3.721551	0.278167
C	2.973701	3.722229	0.332568
C	2.249652	2.583508	0.247114
C	2.346120	-2.410229	-0.268206
C	3.115513	-3.550586	-0.358549
C	4.522189	-3.454663	-0.310381
C	5.140646	-2.211610	-0.193184
C	0.710306	0.062417	-0.012898
O	5.308344	-4.541558	-0.382775
O	5.086631	4.778344	0.348504
C	0.019037	-0.621774	0.996144
C	-1.371422	-0.638788	1.013108
C	-2.108925	0.006456	0.006719
C	-1.410576	0.684631	-1.006089
C	-0.020559	0.718559	-1.012227
C	-5.774360	-0.481160	0.221065
C	-4.451288	-0.984261	0.477269
C	-3.576841	-0.013632	0.017958
N	-4.296020	1.040138	-0.514738
C	-5.634221	0.789599	-0.395750

C	-4.138853	-2.322060	1.073026
C	-6.743555	1.562102	-0.771115
C	-8.002129	1.033579	-0.526345
C	-8.164116	-0.234103	0.078375
C	-7.066348	-0.993654	0.452342
H	6.165353	2.367568	0.140983
H	2.488836	4.691931	0.457759
H	1.161924	2.622799	0.308906
H	1.260618	-2.494400	-0.317736
H	2.652616	-4.532314	-0.470537
H	6.226356	-2.125553	-0.171315
H	4.766473	-5.343091	-0.446142
H	-1.888971	-1.136207	1.833378
H	0.506931	1.247400	-1.808272
H	-3.887864	1.896516	-0.874246
H	-3.189408	-2.723808	0.692778
H	-4.065737	-2.282004	2.171531
H	-4.933462	-3.040521	0.825875
H	-6.614953	2.538712	-1.239682
H	-8.886781	1.607740	-0.806900
H	-9.171480	-0.615355	0.252904
H	-7.197617	-1.970378	0.922988
H	-1.957841	1.181457	-1.809080
H	0.576586	-1.124169	1.788981

HPF-AA(OH) (**12**) S_1min(LE) in water @ M06-2X/def2-SVP, SMD(water)

0 1

C	2.926957	-1.127825	-0.089725
C	4.362039	-1.073270	-0.056087
O	5.014211	0.109007	-0.006715
C	4.324454	1.290147	0.024077
C	2.907776	1.321925	0.012327
C	2.170672	0.073714	-0.045266
C	5.100208	2.426954	0.080481
C	4.491515	3.749970	0.156414
C	3.041557	3.773030	0.194295
C	2.307291	2.619232	0.130266
C	2.361998	-2.433585	-0.220932
C	3.141888	-3.565142	-0.250976
C	4.552201	-3.462986	-0.165655
C	5.156315	-2.206907	-0.082558
C	0.703401	0.061018	-0.019710
O	5.341017	-4.545153	-0.181421
O	5.191700	4.781265	0.197635
C	-0.000238	-0.739265	0.902200
C	-1.388191	-0.758966	0.928131
C	-2.137098	0.011057	0.020022
C	-1.438261	0.816512	-0.899207
C	-0.051638	0.847918	-0.912888
C	-5.798649	-0.498067	0.202145
C	-4.475938	-1.015361	0.419702
C	-3.600549	-0.009053	0.035290
N	-4.324675	1.079776	-0.415039
C	-5.661962	0.817020	-0.315584

C	-4.162602	-2.395239	0.908949
C	-6.773322	1.612301	-0.631691
C	-8.030536	1.062473	-0.429320
C	-8.189300	-0.248593	0.076008
C	-7.089734	-1.030821	0.392304
H	6.187764	2.343273	0.076491
H	2.555053	4.745771	0.282427
H	1.220875	2.683264	0.183629
H	1.281949	-2.535050	-0.317155
H	2.689488	-4.553184	-0.355759
H	6.241040	-2.111178	-0.044634
H	4.805160	-5.352379	-0.236420
H	-1.896831	-1.351806	1.688137
H	0.460922	1.466691	-1.651630
H	-3.921461	1.965586	-0.700845
H	-3.243718	-2.789164	0.452512
H	-4.029429	-2.430021	2.002036
H	-4.985469	-3.079736	0.658864
H	-6.647786	2.622345	-1.023914
H	-8.916676	1.653591	-0.666576
H	-9.195645	-0.645127	0.219410
H	-7.218034	-2.041320	0.786146
H	-1.987384	1.410547	-1.631933
H	0.556315	-1.328866	1.633225

HPF-AA(OH) (**12**) S_1min(ET) in water @ M06-2X/def2-SVP, SMD(water)

0 1

C	2.923748	-1.173956	-0.201019
C	4.340906	-1.098307	-0.194763
O	4.987217	0.096075	-0.034585
C	4.288485	1.271535	0.133526
C	2.866137	1.266679	0.139275
C	2.172076	0.032056	-0.029526
C	5.032027	2.418733	0.292291
C	4.409075	3.704456	0.478282
C	2.967932	3.690151	0.483091
C	2.242135	2.527541	0.321379
C	2.356961	-2.455640	-0.376321
C	3.146994	-3.590362	-0.532048
C	4.539861	-3.476474	-0.515609
C	5.138274	-2.219782	-0.347973
C	0.705288	0.005116	-0.026779
O	5.364141	-4.548774	-0.656626
O	5.082167	4.768978	0.627547
C	-0.011682	-0.173553	1.181180
C	-1.387998	-0.191482	1.193126
C	-2.121911	-0.018965	-0.014000
C	-1.399024	0.164177	-1.228511
C	-0.024196	0.170427	-1.231357
C	-5.762758	-0.256117	0.443544
C	-4.476848	-0.469796	0.979495
C	-3.547393	-0.006888	-0.021701
N	-4.267146	0.445208	-1.094343
C	-5.605580	0.327504	-0.846235

C	-4.198013	-1.106095	2.293323
C	-6.699783	0.683874	-1.638091
C	-7.958693	0.432141	-1.112804
C	-8.144849	-0.157873	0.166576
C	-7.067028	-0.506206	0.947698
H	6.121461	2.354975	0.278801
H	2.458634	4.646082	0.621674
H	1.149776	2.568172	0.333064
H	1.269740	-2.554541	-0.388587
H	2.689893	-4.572739	-0.664940
H	6.223785	-2.121559	-0.335265
H	4.834419	-5.354235	-0.746993
H	-1.906338	-0.294593	2.142451
H	0.520992	0.297898	-2.167670
H	-3.876921	0.890968	-1.919559
H	-3.427368	-1.884277	2.208353
H	-3.843645	-0.363181	3.025021
H	-5.113472	-1.558987	2.691353
H	-6.565512	1.133494	-2.621305
H	-8.838387	0.693389	-1.702811
H	-9.159190	-0.332475	0.524974
H	-7.202534	-0.957506	1.931358
H	-1.928990	0.271336	-2.175094
H	0.543037	-0.284597	2.113945

HPF-AA(O$^-$) (**12**) S_0min in water @ M06-2X/6-31G(d,p), SMD(water)

-1 1

C	2.93614300	-1.21451900	-0.05229500
C	4.36406800	-1.17922900	-0.05273000
O	5.02030200	0.01955600	-0.04922500
C	4.34308000	1.20651100	-0.03748000
C	2.91495200	1.21613000	-0.01940000
C	2.22018300	-0.00546700	-0.03127100
C	5.10264100	2.34696100	-0.03500300
C	4.48201300	3.64142400	-0.00137100
C	3.02740000	3.65117700	0.05210300
C	2.29587300	2.50514500	0.04680600
C	2.33960500	-2.51447000	-0.11099200
C	3.09148400	-3.64717900	-0.12635800
C	4.54635800	-3.61120000	-0.09258400
C	5.14390600	-2.30578200	-0.06585700
C	0.73433300	-0.01736600	-0.02235000
O	5.22894200	-4.67094200	-0.09957000
O	5.14511900	4.71359800	-0.00506600
C	0.03395400	-0.61488700	1.03099700
C	-1.35534600	-0.62156200	1.04151100
C	-2.08083000	-0.02968100	-0.00191600
C	-1.37478800	0.55714800	-1.06230100
C	0.01364100	0.56669100	-1.07021200
C	-5.74709300	0.01353700	0.49678600
C	-4.42945100	0.03120600	1.06936400
C	-3.54716900	-0.03227700	0.00996500
N	-4.25712100	-0.06948500	-1.17899400
C	-5.59960300	-0.05740600	-0.91015900

C	-4.12780600	0.15968600	2.53067400
C	-6.69908000	-0.09976200	-1.77561700
C	-7.95989600	-0.06056100	-1.20363100
C	-8.13090000	0.02028700	0.19535300
C	-7.04031100	0.05761600	1.04735000
H	6.18463800	2.27402800	-0.05162000
H	2.54142800	4.62048900	0.10552700
H	1.21352500	2.55665100	0.10194900
H	1.25772500	-2.58538200	-0.15195300
H	2.62248900	-4.62512900	-0.17379100
H	6.22454200	-2.21333100	-0.06352400
H	-1.88073500	-1.11298200	1.85333300
H	0.54698400	1.02968000	-1.89503200
H	-3.84351800	-0.18746400	-2.09357700
H	-3.17209500	0.66228400	2.70084400
H	-4.90841700	0.74547900	3.02483600
H	-4.08442800	-0.81326900	3.03265200
H	-6.56293900	-0.15893600	-2.85056800
H	-8.83539200	-0.09041600	-1.84438700
H	-9.13568500	0.05068000	0.60430900
H	-7.17754500	0.11489500	2.12356800
H	-1.91279200	1.02767100	-1.87967100
H	0.58300700	-1.07928600	1.84464000

HPF-AA(O$^-$) (**12**) S_1min(LE) in water @ M06-2X/6-31G(d,p), SMD(water)

C	2.94840600	-1.21361900	-0.08763000
C	4.37900200	-1.17749700	-0.08154200
O	5.06003000	0.01945000	-0.04864000
C	4.36498900	1.20799700	-0.00796200
C	2.93436500	1.22602600	0.02043900
C	2.19726500	0.00165400	-0.02728300
C	5.12908100	2.34191600	0.01821200
C	4.52385800	3.65096100	0.09888400
C	3.07652300	3.67138300	0.18273400
C	2.33791000	2.52187200	0.15175300
C	2.36647800	-2.51677700	-0.21218800
C	3.11895000	-3.65684100	-0.25766500
C	4.56712000	-3.61803400	-0.19732900
C	5.15680400	-2.30155500	-0.12250900
C	0.72435400	-0.00634200	-0.01676700
O	5.26806900	-4.66644800	-0.22676500
O	5.21121200	4.70876500	0.11346000
C	0.00669800	-0.77528600	0.91492100
C	-1.38152400	-0.78643100	0.92304000
C	-2.11104400	-0.01791400	0.00412400
C	-1.40028500	0.74845200	-0.93240700
C	-0.01298000	0.75203800	-0.94266100
C	-5.77967800	-0.07193400	0.49037200
C	-4.46376700	-0.14886400	1.06226700
C	-3.57663700	-0.02426900	0.01201100
N	-4.28337800	0.14292500	-1.16822800
C	-5.62727900	0.10315300	-0.90726000
C	-4.16593700	-0.27449700	2.52460400

C	-6.72316700	0.20754600	-1.77122300
C	-7.98685500	0.14273900	-1.20674700
C	-8.16279800	-0.02028500	0.18399700
C	-7.07507700	-0.12752500	1.03435800
H	6.21085700	2.26162000	-0.01073400
H	2.59428000	4.63898500	0.28382900
H	1.25885300	2.58301200	0.23873600
H	1.28702300	-2.59150200	-0.28195100
H	2.64746400	-4.63028000	-0.35326200
H	6.23780500	-2.20762400	-0.11066100
H	-1.90573100	-1.41135900	1.63865600
H	0.51390600	1.34506100	-1.68464200
H	-3.86557700	0.18079800	-2.08773600
H	-3.20740500	0.18521800	2.77924200
H	-4.94399400	0.22325900	3.11102500
H	-4.12948300	-1.31914900	2.85338600
H	-6.58297600	0.33611300	-2.83964800
H	-8.85979600	0.22023000	-1.84696900
H	-9.16905400	-0.06440300	0.58817000
H	-7.21669900	-0.25804800	2.10365900
H	-1.93777900	1.35493200	-1.65577200
H	0.54879900	-1.36987600	1.64456100

HPF-AA(O$^-$) (**12**) S_1min(ET) in water @ M06-2X/6-31G(d,p), SMD(water)

-1 1

C	2.93366400	-1.23891400	0.03685700
C	4.34981700	-1.18610900	0.02575200
O	5.01549700	0.02628700	-0.04558600
C	4.31000800	1.21630000	-0.10860000
C	2.89292700	1.22238000	-0.09808500
C	2.19051600	-0.01971200	-0.02359100
C	5.06254500	2.36769100	-0.18053900
C	4.45119300	3.66268000	-0.24964800
C	3.01880400	3.66392400	-0.23549900
C	2.28286200	2.49649500	-0.16454600
C	2.36709100	-2.53250800	0.10985700
C	3.14198600	-3.67541900	0.16723100
C	4.57363500	-3.62677800	0.15982300
C	5.14082600	-2.31218000	0.08392900
C	0.72674700	-0.04143200	-0.01609400
O	5.30240800	-4.68428500	0.21882200
O	5.14262200	4.74427000	-0.32081600
C	0.00589100	0.01697200	1.20161700
C	-1.36788300	0.01608400	1.21490000
C	-2.09828100	-0.05553400	-0.00285800
C	-1.37607600	-0.13257400	-1.22745500
C	-0.00382400	-0.11908800	-1.22897300
C	-5.73237500	0.17225900	0.46392500
C	-4.44639500	0.35148500	1.00710800
C	-3.52102100	-0.06746600	-0.00872700
N	-4.24348400	-0.46099600	-1.10701100
C	-5.58215300	-0.35118700	-0.84947100

C	-4.16104600	0.93417000	2.34645200
C	-6.67611600	-0.66502100	-1.65577000
C	-7.93063000	-0.42967600	-1.11885800
C	-8.11135000	0.10306600	0.18480400
C	-7.03270500	0.40838100	0.97861900
H	6.14598300	2.29190300	-0.18601200
H	2.51516500	4.62527100	-0.28656700
H	1.19613200	2.54698900	-0.15914600
H	1.28266100	-2.61886500	0.12073400
H	2.67100300	-4.65288800	0.22348500
H	6.22112400	-2.20086500	0.07295100
H	-1.88867700	0.02336200	2.16238800
H	0.54150700	-0.15508400	-2.16625800
H	-3.85757800	-0.89060400	-1.93833800
H	-3.36369700	1.67958200	2.29769300
H	-5.05904500	1.41091400	2.74164600
H	-3.85361500	0.15843800	3.05624700
H	-6.54558600	-1.06714700	-2.65358100
H	-8.80784500	-0.65646400	-1.71556100
H	-9.11893700	0.26694000	0.54904700
H	-7.16357200	0.81247800	1.97685200
H	-1.90555200	-0.15970600	-2.17331200
H	0.55875800	0.05283000	2.13447700

O-RDM-AA (**13**) S_0min in water @ M06-2X/def2-SVP, SMD(water)

1 1

C	2.161800	-1.212608	-0.058761
C	3.585687	-1.168864	-0.048010
O	4.232009	0.018086	-0.037822
C	3.567911	1.195064	-0.020617
C	2.143760	1.216976	0.003362
C	1.436240	-0.003236	-0.024392
C	1.573808	-2.511361	-0.135736
C	2.331582	-3.646572	-0.148874
C	3.768692	-3.580280	-0.087403
C	4.375266	-2.300014	-0.056907
C	4.339988	2.338252	-0.017224
C	3.714177	3.608968	0.022766
C	2.276818	3.653080	0.096022
C	1.536478	2.506356	0.086922
N	4.507977	-4.701165	-0.071990
C	3.871910	-6.011046	-0.098602
C	5.958829	-4.610284	-0.029611
N	4.436245	4.741060	0.005286
C	3.780980	6.040742	0.054917
C	5.887481	4.672853	-0.060777
C	-0.045717	-0.014037	-0.016964
C	-0.746597	-0.653015	1.014807
C	-2.137191	-0.658004	1.024619
C	-2.155657	0.594253	-1.040240
C	-0.765882	0.610250	-1.044998
C	-7.481468	0.004711	-1.786469
C	-8.745464	0.008725	-1.216196

C	-8.921158	0.001503	0.186782
C	-7.831670	-0.014297	1.043724
C	-6.534246	-0.023779	0.493930
C	-6.380355	-0.006532	-0.916944
C	-5.216906	-0.042231	1.071105
N	-5.039770	-0.005204	-1.181718
C	-4.919978	-0.013239	2.538565
C	-4.331946	-0.039193	0.005463
C	-2.864339	-0.032049	-0.001019
H	0.489075	-2.597140	-0.197082
H	1.835259	-4.611959	-0.216065
H	5.454296	-2.167693	-0.041727
H	5.420712	2.222620	-0.041638
H	1.766011	4.610582	0.167234
H	0.451034	2.575378	0.156562
H	3.302008	-6.162086	-1.027128
H	4.647795	-6.779546	-0.039594
H	3.190107	-6.139463	0.754388
H	6.296136	-4.078602	0.872836
H	6.378147	-5.620079	-0.017045
H	6.349668	-4.078933	-0.910396
H	3.209146	6.165981	0.985956
H	4.545676	6.821332	0.011161
H	3.097027	6.175178	-0.795576
H	6.217546	4.142969	-0.966853
H	6.290368	5.689160	-0.084887
H	6.302000	4.151338	0.815133
H	-0.196611	-1.147827	1.817458
H	-2.662980	-1.178635	1.824824

H	-2.693895	1.091990	-1.848710
H	-0.231118	1.105736	-1.857472
H	-7.342356	0.012759	-2.868308
H	-9.623881	0.018689	-1.863722
H	-9.932544	0.006406	0.596237
H	-7.973267	-0.023933	2.126581
H	-4.624488	-0.050616	-2.106143
H	-3.980437	0.514854	2.752841
H	-5.727949	0.502431	3.076848
H	-4.836713	-1.024977	2.966405

O-RDM-AA (**13**) S_1min(LE) in water @ M06-2X/def2-SVP, SMD(water)

1 1

C	2.165605	-1.210825	-0.047192
C	3.595003	-1.168612	-0.034434
O	4.262810	0.022103	-0.035688
C	3.580048	1.204249	-0.029606
C	2.150526	1.228231	0.000496
C	1.415151	0.004037	-0.018524
C	1.589325	-2.515757	-0.128307
C	2.347641	-3.658956	-0.132339
C	3.777331	-3.589674	-0.060027
C	4.378465	-2.295267	-0.031904
C	4.348862	2.340855	-0.041726
C	3.731569	3.627436	-0.005689
C	2.302080	3.678476	0.082856
C	1.558433	2.525645	0.087413
N	4.532700	-4.703473	-0.031053
C	3.908335	-6.016315	-0.048259
C	5.981910	-4.606355	0.020495
N	4.472448	4.750735	-0.042223
C	3.832187	6.055435	-0.006094
C	5.921448	4.672286	-0.123049
C	-0.061517	-0.005029	-0.009609
C	-0.774857	-0.712107	0.972703
C	-2.165489	-0.723612	0.980822
C	-2.184409	0.679870	-0.980530
C	-0.794582	0.688592	-0.987683
C	-7.508307	0.135540	-1.783461
C	-8.774768	0.096917	-1.218954

C	-8.954687	-0.011421	0.178925
C	-7.867617	-0.087255	1.036352
C	-6.568356	-0.055354	0.492209
C	-6.410303	0.063208	-0.913614
C	-5.252288	-0.111566	1.070488
N	-5.068448	0.085357	-1.172811
C	-4.957939	-0.186122	2.536932
C	-4.363598	-0.030900	0.011109
C	-2.895790	-0.022595	0.007141
H	0.505843	-2.605452	-0.199405
H	1.848870	-4.623558	-0.200593
H	5.458111	-2.162167	-0.012241
H	5.429788	2.221719	-0.073716
H	1.791639	4.636696	0.154897
H	0.474695	2.601411	0.170117
H	3.338256	-6.170713	-0.977179
H	4.687349	-6.780328	0.018204
H	3.220950	-6.134644	0.802780
H	6.308025	-4.067197	0.923111
H	6.404389	-5.614245	0.039136
H	6.372470	-4.073801	-0.860138
H	3.263295	6.190105	0.926413
H	4.601748	6.829598	-0.063913
H	3.140532	6.177224	-0.853394
H	6.235609	4.132139	-1.029197
H	6.330119	5.685525	-0.156539
H	6.337765	4.150146	0.752067
H	-0.229442	-1.259710	1.744052
H	-2.690287	-1.301622	1.741768

H	-2.722826	1.236148	-1.750333
H	-0.264742	1.236468	-1.769636
H	-7.365655	0.221611	-2.861486
H	-9.650941	0.151766	-1.867248
H	-9.967302	-0.037464	0.584597
H	-8.013135	-0.174934	2.115210
H	-4.649042	0.106881	-2.096154
H	-4.015687	0.320752	2.787628
H	-5.763587	0.295617	3.109289
H	-4.880438	-1.225215	2.894778

O-RDM-AA (**13**) S_1min(ET) in water @ M06-2X/def2-SVP, SMD(water)

1 1

C	-2.157828	1.235651	-0.022383
C	-3.573435	1.178528	-0.027335
O	-4.229765	-0.024944	-0.031277
C	-3.538753	-1.208751	-0.031562
C	-2.122015	-1.224249	-0.030938
C	-1.412942	0.016121	-0.026231
C	-1.590994	2.529910	-0.009750
C	-2.367142	3.677514	-0.006242
C	-3.783133	3.600574	-0.024721
C	-4.368050	2.309326	-0.022387
C	-4.299388	-2.362485	-0.038733
C	-3.676507	-3.636003	-0.032970
C	-2.258750	-3.671324	-0.045473
C	-1.516978	-2.501226	-0.039921
N	-4.563762	4.727185	-0.044307
C	-3.943759	6.019329	0.172384
C	-5.992061	4.598312	0.164659
N	-4.424037	-4.784768	-0.015827
C	-3.765872	-6.058642	-0.227870
C	-5.854824	-4.697844	-0.229631
C	0.054674	0.036519	-0.020984
C	0.771241	0.040586	1.199162
C	2.148227	0.044965	1.210910
C	2.155751	0.073451	-1.236295
C	0.780395	0.054314	-1.237736
C	7.460758	0.588356	-1.691623
C	8.719037	0.384280	-1.142557

C	8.902352	-0.074610	0.189338
C	7.822476	-0.336574	1.001180
C	6.519773	-0.131350	0.473488
C	6.365619	0.319066	-0.868853
C	5.233110	-0.283681	1.027417
N	5.026407	0.415627	-1.129034
C	4.948223	-0.785344	2.396601
C	4.305483	0.078480	-0.017779
C	2.879366	0.060567	-0.009258
H	-0.503271	2.625944	0.002559
H	-1.869485	4.644799	0.009396
H	-5.446532	2.165900	-0.020617
H	-5.381585	-2.251115	-0.045090
H	-1.732521	-4.623462	-0.058406
H	-0.426783	-2.564596	-0.047805
H	-3.190789	6.233856	-0.599663
H	-4.711988	6.797419	0.113382
H	-3.449656	6.089494	1.157424
H	-6.234113	4.142802	1.141038
H	-6.449673	5.592074	0.122608
H	-6.456092	3.982625	-0.619492
H	-3.010847	-6.250360	0.548137
H	-4.511598	-6.858546	-0.171789
H	-3.265047	-6.115439	-1.210357
H	-6.106854	-4.250780	-1.207387
H	-6.283177	-5.704583	-0.187908
H	-6.339182	-4.095140	0.552256
H	0.217436	0.048428	2.138948
H	2.668602	0.081652	2.164270

H	2.684439	0.060572	-2.189632
H	0.233662	0.042881	-2.181644
H	7.329431	0.936264	-2.715610
H	9.599803	0.580219	-1.755786
H	9.915729	-0.217876	0.563873
H	7.954936	-0.686977	2.025530
H	4.637780	0.779541	-1.994483
H	4.155713	-1.545824	2.390438
H	5.854663	-1.223056	2.830700
H	4.620544	0.033940	3.056052

Oxazone-AA (**14**) S_0min in water @ M06-2X/def2-SVP, SMD(water)

0 1

C	-2.872409	-1.011059	-0.187109
C	-3.349028	0.297355	0.077173
O	-2.453445	1.297545	0.291173
C	-1.124818	1.042338	0.253375
C	-0.694487	-0.318652	-0.029343
N	-1.545715	-1.290944	-0.236986
C	-0.241309	2.047231	0.480761
C	1.189181	1.801257	0.477702
C	1.639849	0.426808	0.112535
C	0.724317	-0.559401	-0.089265
C	-3.844836	-2.017606	-0.399421
C	-5.186889	-1.740107	-0.352691
C	-5.653998	-0.409256	-0.086499
C	-4.691354	0.610034	0.129106
O	1.995708	2.681753	0.783741
N	-6.974696	-0.135139	-0.042880
C	-7.953639	-1.187599	-0.260174
C	-7.423467	1.221028	0.219583
C	3.078639	0.142002	0.055909
N	3.566404	-1.041102	0.581436
C	4.915850	-1.111341	0.379819
C	5.301199	0.065089	-0.315097
C	4.116326	0.854743	-0.518580
C	5.843639	-2.099207	0.743208
C	7.168143	-1.893543	0.387331
C	7.571550	-0.733982	-0.314080
C	6.653801	0.242960	-0.667835

C	4.062013	2.147506	-1.270288
H	-0.592225	3.053047	0.711370
H	1.032446	-1.579229	-0.329202
H	-3.492717	-3.029901	-0.603224
H	-5.900979	-2.543035	-0.521574
H	-4.971274	1.640099	0.337131
H	-7.851641	-1.632980	-1.261412
H	-7.854425	-1.989882	0.486355
H	-8.956301	-0.758951	-0.173671
H	-7.078310	1.574190	1.203602
H	-7.051662	1.920539	-0.544951
H	-8.517020	1.242886	0.206961
H	3.006748	-1.718278	1.088843
H	5.529278	-2.994247	1.281689
H	7.915340	-2.643343	0.652848
H	8.623249	-0.610517	-0.577363
H	6.970442	1.138372	-1.207150
H	3.051130	2.353941	-1.644741
H	4.362417	2.999413	-0.641222
H	4.745819	2.114206	-2.131349

Oxazone-AA (**14**) S_1min(LE) in water @ M06-2X/def2-SVP, SMD(water)

0 1

C	-2.850847	-1.037469	-0.137822
C	-3.339154	0.298994	0.063225
O	-2.451968	1.321131	0.237657
C	-1.112364	1.040014	0.219280
C	-0.676077	-0.319232	0.012077
N	-1.547741	-1.347904	-0.170280
C	-0.237553	2.067181	0.396758
C	1.199261	1.847938	0.423295
C	1.653008	0.477000	0.128375
C	0.710607	-0.542427	-0.020298
C	-3.848593	-2.047065	-0.306882
C	-5.188395	-1.764711	-0.284356
C	-5.651334	-0.418732	-0.087352
C	-4.674354	0.607799	0.087189
O	1.980381	2.767117	0.725260
N	-6.965926	-0.129977	-0.067117
C	-7.955073	-1.181022	-0.241662
C	-7.411134	1.239863	0.126578
C	3.065352	0.172127	0.080234
N	3.518812	-1.071935	0.482370
C	4.869821	-1.157949	0.305247
C	5.302900	0.071105	-0.262980
C	4.155569	0.910527	-0.408723
C	5.761086	-2.200864	0.590038
C	7.098359	-1.998217	0.280002
C	7.550247	-0.788779	-0.300228
C	6.669634	0.243127	-0.575152

C	4.171614	2.246249	-1.077227
H	-0.606490	3.079077	0.568250
H	1.031035	-1.567589	-0.215136
H	-3.500175	-3.069647	-0.458125
H	-5.904310	-2.573328	-0.418832
H	-4.954959	1.647640	0.242425
H	-7.836850	-1.678309	-1.216391
H	-7.867033	-1.942628	0.547878
H	-8.953565	-0.738458	-0.192489
H	-7.064326	1.635234	1.093591
H	-7.028805	1.895607	-0.670702
H	-8.503960	1.263963	0.108116
H	2.941618	-1.774284	0.932751
H	5.411502	-3.134440	1.032150
H	7.819200	-2.791421	0.485223
H	8.610418	-0.673752	-0.530378
H	7.021156	1.176939	-1.018611
H	3.199322	2.485339	-1.525635
H	4.412927	3.054522	-0.370686
H	4.934483	2.255517	-1.869246

Oxazone-AA (**14**) S_1min(TICT) in water @ ωB97X-D/def2-SVP, SMD(water)

0 1

C	2.878862	-1.060883	-0.139636
C	3.320547	0.268012	-0.138144
O	2.407056	1.296348	-0.158816
C	1.076810	1.000569	-0.174180
C	0.669241	-0.402215	-0.177977
N	1.529149	-1.402148	-0.163019
C	0.189627	2.032168	-0.186091
C	-1.244145	1.803283	-0.191019
C	-1.641413	0.418033	-0.208683
C	-0.736771	-0.633017	-0.198185
C	3.878740	-2.039333	-0.107327
C	5.231724	-1.719257	-0.078036
C	5.662090	-0.372788	-0.092732
C	4.661752	0.622438	-0.107553
O	-2.088311	2.739025	-0.163401
N	7.008587	-0.036171	-0.100035
C	7.968762	-1.058436	0.265351
C	7.370764	1.329272	0.222278
C	-3.070438	0.149119	-0.166760
N	-3.856092	-0.026853	-1.228624
C	-5.164467	-0.310907	-0.820271
C	-5.182250	-0.319515	0.592350
C	-3.853510	-0.031681	1.028176
C	-6.295937	-0.551129	-1.578855
C	-7.477439	-0.808427	-0.875290
C	-7.522259	-0.823388	0.528504

C	-6.378638	-0.579985	1.278575
C	-3.319202	0.101783	2.388588
H	0.553914	3.061722	-0.181822
H	-1.081491	-1.668395	-0.203228
H	3.569895	-3.087308	-0.099050
H	5.955356	-2.532508	-0.048844
H	4.901511	1.684698	-0.102398
H	7.812578	-1.451952	1.288076
H	7.937611	-1.908198	-0.432036
H	8.979765	-0.636421	0.212627
H	6.948392	2.039716	-0.503467
H	7.037725	1.637218	1.232095
H	8.461645	1.432858	0.176265
H	-3.543932	0.038611	-2.194214
H	-6.268870	-0.540394	-2.668529
H	-8.392480	-1.003632	-1.437635
H	-8.468312	-1.029139	1.031123
H	-6.401919	-0.588778	2.369435
H	-2.468246	-0.586577	2.529366
H	-2.907652	1.117880	2.520949
H	-4.081666	-0.088466	3.152272

Si-RDM-AA (**15**) S_0min in water @ M06-2X/def2-SVP, SMD(water)

1 1

C	-1.815366	1.291004	0.017653
C	-3.239955	1.479558	0.020581
Si	-4.411406	0.010027	-0.036300
C	-3.237877	-1.458556	-0.072012
C	-1.813660	-1.268635	-0.048831
C	-1.183782	0.011421	-0.011185
C	-1.010475	2.473318	0.038941
C	-1.546382	3.733525	0.070438
C	-2.962427	3.922505	0.081016
C	-3.779377	2.745656	0.052457
C	-3.775490	-2.725210	-0.110388
C	-2.956996	-3.901245	-0.126436
C	-1.541373	-3.710841	-0.096668
C	-1.007266	-2.450058	-0.058341
N	-3.503195	5.147908	0.111861
C	-2.648091	6.326489	0.135551
C	-4.949598	5.319560	0.117283
C	-5.450673	0.062176	-1.591321
C	-5.480565	-0.042990	1.498222
N	-3.496197	-5.127152	-0.163452
C	-2.639871	-6.305028	-0.175251
C	-4.942278	-5.300076	-0.186838
C	0.313401	0.012376	-0.001515
C	1.012699	0.082658	1.206920
C	2.404985	0.088172	1.215202
C	2.417855	-0.048268	-1.194021
C	1.026469	-0.050053	-1.202748

C	7.744445	0.847337	-1.578918
C	9.010286	0.567728	-1.085651
C	9.189125	-0.103085	0.145690
C	8.101412	-0.502310	0.907319
C	6.802796	-0.228679	0.434383
C	6.645685	0.438610	-0.808574
C	5.486177	-0.492686	0.951064
N	5.303861	0.566374	-1.036514
C	5.190040	-1.229275	2.220857
C	4.598932	0.019862	0.019941
C	3.129800	0.016806	0.014962
H	0.075607	2.386157	0.026641
H	-0.877999	4.591752	0.084089
H	-4.864297	2.862371	0.052359
H	-4.860186	-2.842969	-0.125696
H	-0.871898	-4.568314	-0.100663
H	0.078460	-2.361572	-0.030818
H	-2.002337	6.330248	1.025775
H	-2.008842	6.370613	-0.758298
H	-3.278662	7.219329	0.157979
H	-5.404388	4.884852	-0.784654
H	-5.402808	4.844772	0.999607
H	-5.177346	6.388723	0.141724
H	-4.815657	0.089743	-2.488824
H	-6.097748	-0.826619	-1.647353
H	-6.093847	0.955587	-1.589806
H	-6.129663	0.845045	1.541530
H	-6.122621	-0.937099	1.484288
H	-4.863180	-0.069760	2.407945

H	-1.981337	-6.307800	-1.056053
H	-2.013558	-6.349079	0.727714
H	-3.269319	-7.198356	-0.207350
H	-5.384655	-4.828525	-1.076383
H	-5.168873	-6.369498	-0.210623
H	-5.408765	-4.862807	0.707842
H	0.462723	0.145899	2.147912
H	2.933328	0.175676	2.164888
H	2.955485	-0.114736	-2.141566
H	0.487611	-0.107244	-2.150525
H	7.602852	1.362721	-2.529837
H	9.887041	0.870706	-1.660516
H	10.201372	-0.305581	0.499192
H	8.245837	-1.016734	1.859838
H	4.885653	1.050389	-1.823858
H	4.238821	-1.776145	2.160159
H	5.986360	-1.957987	2.429885
H	5.129587	-0.554631	3.089698

Si-RDM-AA (**15**) S_1min(LE) in water @ M06-2X/def2-SVP, SMD(water)

1 1

C	-1.814212	1.289398	0.015143
C	-3.247158	1.472191	0.016003
Si	-4.419134	0.010572	-0.038498
C	-3.244111	-1.449346	-0.068246
C	-1.811671	-1.264202	-0.046098
C	-1.157881	0.013103	-0.010773
C	-1.015942	2.486976	0.039161
C	-1.561743	3.739980	0.069297
C	-2.984468	3.922338	0.075683
C	-3.790694	2.741992	0.047216
C	-3.784994	-2.720120	-0.104754
C	-2.976432	-3.899128	-0.119448
C	-1.554206	-3.714324	-0.094127
C	-1.011024	-2.460329	-0.057797
N	-3.528896	5.151712	0.103066
C	-2.681894	6.333812	0.124886
C	-4.973044	5.318202	0.106509
C	-5.480746	0.056947	-1.583385
C	-5.514755	-0.037479	1.482337
N	-3.518441	-5.129461	-0.151685
C	-2.669309	-6.310191	-0.161488
C	-4.962181	-5.298332	-0.171776
C	0.337241	0.014417	-0.000855
C	1.044634	0.061737	1.206223
C	2.437424	0.069137	1.217504
C	2.451271	-0.025351	-1.192888
C	1.059328	-0.025034	-1.199965

C	7.778638	0.876513	-1.561878
C	9.044689	0.587401	-1.074327
C	9.223540	-0.105391	0.144657
C	8.135750	-0.517382	0.899585
C	6.837082	-0.234831	0.432202
C	6.679992	0.454816	-0.798564
C	5.520323	-0.508151	0.943872
N	5.338033	0.587066	-1.023691
C	5.223918	-1.268272	2.199694
C	4.632846	0.021169	0.022375
C	3.163459	0.018591	0.016930
H	0.070346	2.403823	0.031642
H	-0.899891	4.604133	0.085889
H	-4.876855	2.852540	0.045775
H	-4.870871	-2.832406	-0.119519
H	-0.890606	-4.577274	-0.100506
H	0.074926	-2.375169	-0.034917
H	-2.037902	6.342741	1.017321
H	-2.038121	6.375292	-0.766749
H	-3.317613	7.222850	0.141177
H	-5.423300	4.874849	-0.794763
H	-5.423691	4.838101	0.988558
H	-5.206513	6.385890	0.128758
H	-4.859634	0.081815	-2.490772
H	-6.130047	-0.831148	-1.629140
H	-6.126094	0.949111	-1.577685
H	-6.166099	0.849736	1.513960
H	-6.158732	-0.930419	1.461912
H	-4.913984	-0.061952	2.403313

H	-2.012834	-6.318059	-1.044776
H	-2.038040	-6.350621	0.739101
H	-3.303279	-7.200256	-0.186808
H	-5.403026	-4.823221	-1.061475
H	-5.193766	-6.366478	-0.191589
H	-5.423792	-4.851300	0.721876
H	0.495791	0.106500	2.149289
H	2.965823	0.140342	2.168718
H	2.989865	-0.075549	-2.141120
H	0.522396	-0.063690	-2.150116
H	7.636948	1.408740	-2.503476
H	9.921359	0.900058	-1.644117
H	10.235782	-0.314868	0.494116
H	8.280322	-1.048775	1.842739
H	4.919526	1.083865	-1.802819
H	4.271955	-1.812615	2.128804
H	6.019341	-2.001922	2.394638
H	5.164471	-0.610459	3.081438

Si-RDM-AA (**15**) S_1min(ET) in water @ M06-2X/def2-SVP, SMD(water)

1 1

C	1.81380700	-1.29773700	0.04000000
C	3.23290300	-1.46143400	0.04307700
Si	4.37281000	0.02665900	-0.01035400
C	3.19521000	1.48626800	-0.02229400
C	1.78055500	1.28706100	-0.01642000
C	1.15356800	-0.01343500	0.00930900
C	1.04129400	-2.49245100	0.06210600
C	1.61140700	-3.75101200	0.09267400
C	3.01820800	-3.91777100	0.11401600
C	3.79726300	-2.73334800	0.07510500
C	3.72734000	2.77207600	-0.04324300
C	2.91881800	3.93732000	-0.05059500
C	1.51653300	3.73478600	-0.06214100
C	0.97818800	2.46209300	-0.04105500
N	3.59378700	-5.16047500	0.16831300
C	2.75974800	-6.33204400	-0.01028700
C	5.02413700	-5.28938800	-0.02705900
C	5.43220900	0.00552000	-1.56170200
C	5.48960200	0.07605800	1.49925100
N	3.46337400	5.19480700	-0.04690100
C	2.60089800	6.33829800	-0.26730700
C	4.89096300	5.35172600	-0.24176700
C	-0.32213900	-0.03272000	0.00683000
C	-1.04710700	-0.01694100	1.22264400
C	-2.42345200	-0.02062700	1.22710500
C	-2.41742700	-0.08967200	-1.21929900
C	-1.04273700	-0.07005700	-1.21327100

C	-7.71950100	-0.61304000	-1.70027200
C	-8.98086500	-0.40430800	-1.16173900
C	-9.17242800	0.07204300	0.16314200
C	-8.09773900	0.34685100	0.97756000
C	-6.79156700	0.13720100	0.46065000
C	-6.62874600	-0.33058700	-0.87470100
C	-5.50771600	0.29879900	1.02020100
N	-5.28880900	-0.42803800	-1.12576400
C	-5.23257200	0.81925900	2.38498300
C	-4.57459500	-0.07510300	-0.01305900
C	-3.14894300	-0.05657900	0.00329800
H	-0.04865100	-2.43444700	0.04936400
H	0.95411800	-4.61885200	0.10173100
H	4.88576200	-2.82030300	0.07124400
H	4.81325200	2.88641000	-0.05259100
H	0.83764400	4.58560900	-0.08549500
H	-0.11003400	2.37611200	-0.04757300
H	1.98649800	-6.39409400	0.76928200
H	2.25282100	-6.34162600	-0.99132500
H	3.38330800	-7.22877800	0.06587600
H	5.34912300	-4.91736900	-1.01467300
H	5.58358200	-4.73787900	0.74242800
H	5.30056200	-6.34591800	0.05277500
H	4.80726500	-0.02086200	-2.46675300
H	6.06909300	0.90278400	-1.60611400
H	6.08948700	-0.87801600	-1.56816900
H	6.15157900	-0.80372700	1.51923700
H	6.12308700	0.97663900	1.48055200
H	4.89935700	0.08681200	2.42760200

H	2.09129600	6.29769300	-1.24612100
H	1.82831000	6.41189100	0.51197100
H	3.20320000	7.25175600	-0.22812100
H	5.22975100	4.94951200	-1.21273300
H	5.14058100	6.41717000	-0.20265300
H	5.46031600	4.84486200	0.55091100
H	-0.49830800	-0.00985000	2.16555100
H	-2.94905100	-0.04111600	2.17808200
H	-2.94063100	-0.09300700	-2.17572000
H	-0.49061000	-0.07393300	-2.15428300
H	-7.58133200	-0.97441600	-2.71872000
H	-9.85782500	-0.61016900	-1.77720100
H	-10.18818700	0.21829000	0.53013200
H	-8.23706800	0.71042400	1.99643200
H	-4.89454600	-0.80442700	-1.98313100
H	-4.44245000	1.58218100	2.37387400
H	-6.14283100	1.26051100	2.80749000
H	-4.90696800	0.01022200	3.05782400

Si-RDM-AA (**28**) S_0min in water @ M06-2X/def2-SVP, SMD(water)

1 1

C	-1.904525	-1.257669	0.072128
C	-3.328170	-1.446458	0.113065
Si	-4.498848	0.024812	0.124620
C	-3.327830	1.491440	0.012894
C	-1.904235	1.299979	-0.009067
C	-1.272192	0.020901	0.025811
C	-1.098625	-2.439102	0.064600
C	-1.633023	-3.700030	0.095279
C	-3.048543	-3.889732	0.138974
C	-3.866293	-2.713252	0.147360
C	-3.865242	2.758126	-0.035393
C	-3.046499	3.931453	-0.110409
C	-1.630844	3.739031	-0.134030
C	-1.097323	2.478433	-0.082715
N	-3.588027	-5.115932	0.168481
C	-2.732327	-6.294247	0.159002
C	-5.033813	-5.288394	0.206087
C	-5.465592	0.082346	1.725929
C	-5.637507	-0.027746	-1.358997
N	-3.585133	5.157519	-0.155501
C	-2.728340	6.332909	-0.227456
C	-5.030932	5.332259	-0.133884
C	0.226546	0.020625	0.010616
C	0.927111	0.007599	-1.206579
C	2.321633	0.012579	-1.149501
C	2.315370	0.024408	1.280651
C	0.918289	0.025644	1.222624

C	7.724100	1.321374	1.041750
C	8.963154	0.877038	0.603118
C	9.079077	-0.177276	-0.330754
C	7.953511	-0.804111	-0.844631
C	6.681721	-0.373784	-0.419291
C	6.587549	0.683932	0.522813
C	5.340074	-0.789232	-0.733354
N	5.258754	0.894779	0.768418
C	4.968320	-1.897972	-1.667397
C	4.502220	0.015881	0.016216
C	3.001575	-0.010504	2.621270
C	0.198934	-0.004373	-2.522626
C	3.027101	0.026045	0.062720
H	-0.012898	-2.348822	0.029794
H	-0.964275	-4.558027	0.084759
H	-4.950673	-2.830319	0.179205
H	-4.949626	2.877803	-0.014120
H	-0.961455	4.594656	-0.191406
H	-0.011328	2.385582	-0.101511
H	-2.116529	-6.330139	-0.751533
H	-2.063602	-6.305750	1.032024
H	-3.361865	-7.187559	0.190129
H	-5.465805	-4.829444	1.107271
H	-5.510141	-4.837951	-0.676784
H	-5.260940	-6.357913	0.216895
H	-4.790004	0.112370	2.593241
H	-6.107277	0.976479	1.750783
H	-6.109668	-0.805959	1.814425
H	-6.285496	0.862021	-1.374659

H	-5.062537	-0.058333	-2.296003
H	-6.281208	-0.919650	-1.313992
H	-2.061042	6.390530	0.644779
H	-2.110994	6.319445	-1.137586
H	-3.356964	7.227237	-0.245107
H	-5.503711	4.840469	-0.996485
H	-5.467287	4.917239	0.786229
H	-5.257417	6.401164	-0.175542
H	2.884417	0.020873	-2.085837
H	0.350456	0.015837	2.156569
H	7.631991	2.133749	1.763951
H	9.867640	1.350971	0.988207
H	10.071675	-0.498245	-0.650800
H	8.048137	-1.616381	-1.568677
H	4.879294	1.609382	1.379212
H	3.934022	-2.230374	-1.505140
H	5.059601	-1.593779	-2.722256
H	5.631145	-2.764143	-1.522357
H	3.876372	-0.675539	2.606193
H	3.350780	0.988023	2.926865
H	2.306568	-0.364305	3.393456
H	-0.431649	0.891546	-2.632250
H	0.905942	-0.034297	-3.361109
H	-0.465517	-0.879093	-2.596257

Si-RDM-AA (**28**) S_1min(LE) in water @ M06-2X/def2-SVP, SMD(water)

1 1

C	-1.904477	-1.251977	0.057191
C	-3.335791	-1.435579	0.118393
Si	-4.501780	0.028796	0.213639
C	-3.337056	1.484451	0.020272
C	-1.905403	1.298769	-0.024408
C	-1.248311	0.023965	0.025571
C	-1.105745	-2.448477	0.012349
C	-1.650458	-3.702457	0.022470
C	-3.071996	-3.885924	0.079850
C	-3.878391	-2.706077	0.126756
C	-3.880566	2.752495	-0.053225
C	-3.074584	3.927804	-0.168342
C	-1.652818	3.742604	-0.211075
C	-1.107249	2.490931	-0.140612
N	-3.615603	-5.116071	0.089475
C	-2.768541	-6.297837	0.054898
C	-5.058963	-5.283704	0.134012
C	-5.372161	0.086169	1.874102
C	-5.768661	-0.019987	-1.166867
N	-3.618897	5.155985	-0.230443
C	-2.772184	6.334733	-0.325253
C	-5.062398	5.324003	-0.197800
C	0.247919	0.023861	0.022268
C	0.961047	0.006152	-1.190062
C	2.355214	0.010012	-1.132407
C	2.341730	0.027782	1.297346
C	0.943985	0.031618	1.233413

C	7.753674	1.318920	1.070529
C	8.993605	0.874402	0.634263
C	9.111037	-0.180291	-0.298892
C	7.986231	-0.807349	-0.814374
C	6.713698	-0.376875	-0.391485
C	6.617998	0.681449	0.549886
C	5.372539	-0.792291	-0.707553
N	5.288684	0.892629	0.792651
C	5.002403	-1.901568	-1.641686
C	4.533043	0.013441	0.039727
C	3.023911	-0.005133	2.640463
C	0.233456	-0.011336	-2.506212
C	3.057689	0.025295	0.082309
H	-0.020364	-2.361821	-0.036772
H	-0.989001	-4.566145	-0.017721
H	-4.963263	-2.817501	0.176466
H	-4.965653	2.866158	-0.013131
H	-0.992013	4.602804	-0.303018
H	-0.021569	2.402270	-0.180128
H	-2.162058	-6.323533	-0.863184
H	-2.088329	-6.321804	0.919640
H	-3.403029	-7.187487	0.082231
H	-5.480290	-4.841082	1.049615
H	-5.538344	-4.803765	-0.732677
H	-5.292247	-6.351566	0.119223
H	-4.647051	0.114659	2.700703
H	-6.011352	0.980331	1.939889
H	-6.011756	-0.801000	2.001443
H	-6.412883	0.872130	-1.128390

H	-5.280866	-0.055951	-2.151985
H	-6.413796	-0.906358	-1.064038
H	-2.090313	6.400867	0.535887
H	-2.167489	6.315578	-1.244726
H	-3.406726	7.224654	-0.340156
H	-5.539656	4.799767	-1.039770
H	-5.485004	4.928568	0.738616
H	-5.296882	6.389414	-0.268112
H	2.921420	0.015374	-2.066868
H	0.375172	0.025556	2.167102
H	7.660360	2.131664	1.792178
H	9.897430	1.348532	1.020666
H	10.104145	-0.501438	-0.617207
H	8.082135	-1.619932	-1.537921
H	4.908099	1.607457	1.402448
H	3.968207	-2.234579	-1.480017
H	5.094037	-1.597835	-2.696684
H	5.665724	-2.767290	-1.496016
H	3.897896	-0.671476	2.630599
H	3.373812	0.993211	2.946185
H	2.325877	-0.356473	3.411192
H	-0.410182	0.876128	-2.611900
H	0.938611	-0.030806	-3.346937
H	-0.421733	-0.893479	-2.579734

Si-RDM-AA (**28**) S_1min(ET) in water @ M06-2X/def2-SVP, SMD(water)

1 1

C	-1.84172200	1.28281700	-0.12744500
C	-3.25162000	1.51118200	-0.13780900
Si	-4.45965600	0.07683000	-0.10496000
C	-3.35072700	-1.43451000	-0.05251500
C	-1.92921700	-1.29966600	-0.05298900
C	-1.24099600	-0.02994800	-0.09150600
C	-1.01481100	2.44070100	-0.13487700
C	-1.52640500	3.72462000	-0.16062900
C	-2.92400900	3.95545100	-0.19162900
C	-3.75684100	2.80763400	-0.16684000
C	-3.94061900	-2.69467500	-0.00937400
C	-3.18549000	-3.89420100	0.03146100
C	-1.77571800	-3.75457800	0.05109900
C	-1.18062600	-2.50783900	0.00487300
N	-3.44207200	5.22284600	-0.24227100
C	-2.55816000	6.35607600	-0.05645500
C	-4.86824200	5.41537300	-0.07054600
C	-5.53674800	0.06927800	-1.64389600
C	-5.55689700	0.15937800	1.41756000
N	-3.78557200	-5.12666700	0.05116100
C	-2.97827600	-6.29873200	0.32521600
C	-5.21999000	-5.21411200	0.23894400
C	0.23406900	-0.07873000	-0.08275000
C	0.95539000	-0.06226700	1.15069600
C	2.33280700	-0.07544100	1.09917100
C	2.32413300	-0.17506800	-1.36690000
C	0.94326900	-0.13255500	-1.29977300

C	7.77302200	-0.91999200	-1.32909900
C	8.98036100	-0.59130900	-0.73064200
C	9.04863600	0.12807600	0.49182900
C	7.90251500	0.53020700	1.14127300
C	6.65046100	0.20206900	0.56037400
C	6.61078900	-0.50726600	-0.67135800
C	5.31742400	0.43441300	0.96646200
N	5.29892900	-0.67079400	-1.01960500
C	4.92452400	1.20955900	2.17420100
C	4.48355500	-0.13920200	-0.05564900
C	2.97628500	-0.19823400	-2.71939400
C	0.21479800	-0.04019500	2.45189800
C	3.05357800	-0.13333900	-0.12403700
H	0.07152700	2.33162000	-0.11615100
H	-0.83003300	4.56155000	-0.16020400
H	-4.84015700	2.94442300	-0.17056600
H	-5.03062600	-2.75915200	-0.00650300
H	-1.13588500	-4.63409600	0.09863200
H	-0.08940300	-2.46953400	0.01737300
H	-2.05214600	6.33758600	0.92480300
H	-1.78222700	6.38980200	-0.83528100
H	-3.14295700	7.27895600	-0.12700700
H	-5.43872700	4.89625700	-0.85453400
H	-5.22699000	5.05013100	0.90777700
H	-5.09480900	6.48405200	-0.14489900
H	-4.92315100	0.02342600	-2.55588300
H	-6.21360000	-0.79932200	-1.63718600
H	-6.15396600	0.98024500	-1.68674800
H	-6.23765000	-0.70555300	1.45084100

H	-4.95500400	0.16239400	2.33849300
H	-6.17061200	1.07359400	1.40123200
H	-2.19698300	-6.43286500	-0.43697100
H	-2.48385000	-6.24714800	1.31132600
H	-3.61975100	-7.18588100	0.30545900
H	-5.54640700	-4.78011200	1.20057400
H	-5.75975000	-4.69533400	-0.56638900
H	-5.51827900	-6.26741800	0.21525200
H	2.88168400	-0.10391700	2.03800700
H	0.37050800	-0.12680200	-2.22944700
H	7.72767600	-1.46780300	-2.26983000
H	9.90983600	-0.89092000	-1.21708000
H	10.02608600	0.36066200	0.91452200
H	7.94736900	1.08087300	2.08189600
H	4.98585900	-1.20680100	-1.82159500
H	4.04643300	1.84073400	1.98513200
H	4.67892100	0.53914500	3.01347500
H	5.75817000	1.84705700	2.49387400
H	3.81629200	0.50647000	-2.78322900
H	3.35267700	-1.20443600	-2.96574100
H	2.23974600	0.06511800	-3.48718200
H	-0.44416400	-0.91804700	2.53492900
H	0.90878900	-0.03308200	3.30101000
H	-0.42925500	0.85095200	2.50857900

Si-RDM-AA (**29**) S_0min in water @ M06-2X/def2-SVP, SMD(water)

1 1

C	1.261810	1.256746	0.552975
C	2.449206	1.767131	-0.074929
Si	3.810765	0.610425	-0.659507
C	3.119667	-1.088255	-0.246491
C	1.820393	-1.234541	0.350065
C	0.990929	-0.134955	0.724795
C	0.320874	2.224954	1.026834
C	0.503035	3.573877	0.870205
C	1.666992	4.080728	0.214952
C	2.631716	3.122116	-0.235478
C	3.877640	-2.194468	-0.556870
C	3.416736	-3.528655	-0.311214
C	2.109409	-3.673644	0.246233
C	1.357059	-2.572019	0.557790
N	1.853643	5.395778	0.039362
C	0.863200	6.348054	0.522561
C	3.050254	5.890503	-0.627569
C	5.390525	0.935439	0.288997
C	4.076755	0.774126	-2.504341
N	4.175707	-4.593779	-0.601169
C	3.681283	-5.939273	-0.343249
C	5.499456	-4.421441	-1.183964
C	-0.317200	-0.476424	1.367392
C	-1.492664	-0.409920	0.617064
C	-2.732265	-0.707755	1.201621
C	-1.594953	-1.170009	3.294916
C	-0.367069	-0.857766	2.711814

C	-7.345150	-1.694940	-0.309222
C	-8.192182	-1.153713	-1.265007
C	-7.788812	-0.069413	-2.077277
C	-6.526391	0.489383	-1.948955
C	-5.641668	-0.041887	-0.989861
C	-6.070254	-1.124676	-0.177856
C	-4.296155	0.284421	-0.598101
N	-5.040671	-1.433839	0.666989
C	-3.478919	1.405657	-1.162401
C	-3.967436	-0.601126	0.413724
C	-2.769283	-1.096986	2.550575
H	-0.579776	1.895753	1.544237
H	-0.250577	4.256137	1.257477
H	3.542757	3.482297	-0.715928
H	4.861391	-2.055929	-1.008022
H	1.695181	-4.663467	0.425471
H	0.363691	-2.741057	0.971937
H	-0.113337	6.179315	0.045735
H	0.736462	6.269735	1.612169
H	1.199062	7.360520	0.282343
H	3.958787	5.616268	-0.071534
H	3.130112	5.487571	-1.647469
H	2.994669	6.980732	-0.688516
H	5.226262	0.840458	1.372270
H	6.170328	0.218788	-0.011243
H	5.758426	1.951371	0.078323
H	4.842794	0.060054	-2.843559
H	3.145980	0.577019	-3.055982
H	4.421436	1.790118	-2.751008

H	2.774505	-6.148861	-0.929534
H	4.453769	-6.659897	-0.625046
H	3.446088	-6.076045	0.722205
H	6.152096	-3.836876	-0.519492
H	5.948934	-5.406485	-1.336005
H	5.442077	-3.909234	-2.155434
H	-1.437952	-0.137898	-0.438308
H	-1.636602	-1.465357	4.344191
H	0.553443	-0.911737	3.296015
H	-7.654293	-2.529959	0.320918
H	-9.191785	-1.572775	-1.392075
H	-8.484986	0.329296	-2.816857
H	-6.218618	1.324523	-2.581909
H	-5.035301	-2.197849	1.334121
H	-2.767709	1.802344	-0.423690
H	-2.898707	1.094777	-2.045815
H	-4.134240	2.230331	-1.477711
H	-3.725084	-1.324664	3.026178

Si-RDM-AA (**29**) S_1min(LE) in water @ M06-2X/def2-SVP, SMD(water)

1 1

C	1.256660	1.243975	0.551915
C	2.452068	1.752975	-0.079338
Si	3.812907	0.606792	-0.667287
C	3.124006	-1.084638	-0.248288
C	1.816254	-1.240539	0.344862
C	0.964457	-0.150806	0.730877
C	0.318903	2.227279	1.028927
C	0.509501	3.571821	0.868793
C	1.678843	4.075729	0.208496
C	2.635080	3.112716	-0.240934
C	3.893188	-2.192035	-0.549236
C	3.445810	-3.527580	-0.305400
C	2.128194	-3.683041	0.239287
C	1.361884	-2.592076	0.542581
N	1.867236	5.395797	0.034770
C	0.884954	6.351771	0.521896
C	3.060286	5.886393	-0.635523
C	5.412824	0.940030	0.251870
C	4.096266	0.775879	-2.513129
N	4.216719	-4.593871	-0.582811
C	3.734587	-5.941396	-0.322613
C	5.542174	-4.413864	-1.152131
C	-0.342703	-0.494069	1.368818
C	-1.523837	-0.421316	0.624449
C	-2.766423	-0.716812	1.205162
C	-1.631353	-1.206366	3.292011
C	-0.403393	-0.888061	2.710995

C	-7.382163	-1.675405	-0.316719
C	-8.225755	-1.125730	-1.270894
C	-7.817029	-0.038460	-2.076369
C	-6.552694	0.514939	-1.942790
C	-5.671444	-0.024918	-0.985311
C	-6.105416	-1.110755	-0.180135
C	-4.325231	0.293735	-0.589876
N	-5.078026	-1.428686	0.664153
C	-3.503265	1.415002	-1.147208
C	-4.000825	-0.598884	0.417472
C	-2.806329	-1.120657	2.549878
H	-0.581600	1.900517	1.547811
H	-0.239660	4.259901	1.256406
H	3.547510	3.468522	-0.723343
H	4.879701	-2.045393	-0.993029
H	1.721372	-4.677854	0.412227
H	0.365056	-2.766844	0.945597
H	-0.094270	6.187365	0.047324
H	0.759383	6.268022	1.611958
H	1.226216	7.362723	0.284033
H	3.969466	5.603911	-0.082508
H	3.134776	5.477559	-1.654508
H	3.009943	6.976578	-0.697164
H	5.272293	0.848878	1.338902
H	6.190665	0.226135	-0.060768
H	5.776105	1.955689	0.030727
H	4.866074	0.063711	-2.848675
H	3.171738	0.580850	-3.076117
H	4.444984	1.791739	-2.755804

H	2.836039	-6.162817	-0.918735
H	4.518259	-6.655293	-0.589369
H	3.484661	-6.071556	0.741041
H	6.180790	-3.817500	-0.482785
H	6.002417	-5.394615	-1.297820
H	5.487283	-3.900372	-2.124214
H	-1.470012	-0.142506	-0.429431
H	-1.673868	-1.513319	4.338120
H	0.515709	-0.948810	3.297439
H	-7.695438	-2.512841	0.308172
H	-9.226670	-1.540428	-1.401927
H	-8.510454	0.366927	-2.814914
H	-6.240791	1.352419	-2.570635
H	-5.076409	-2.196461	1.326940
H	-2.788879	1.802453	-0.406700
H	-2.925747	1.107747	-2.033676
H	-4.154903	2.245228	-1.455691
H	-3.763111	-1.349055	3.023311

Si-RDM-AA (**29**) S_1min(ET) in water @ M06-2X/def2-SVP, SMD(water)

1 1

C	1.48093900	1.26298400	0.57485300
C	2.73191100	1.50800300	-0.07576100
Si	3.80648000	0.08763700	-0.65877600
C	2.83414500	-1.43477800	-0.16037700
C	1.56828700	-1.31358200	0.49538300
C	0.95109600	-0.05359200	0.82900800
C	0.74177200	2.41827100	0.96158400
C	1.19024200	3.70588600	0.73633400
C	2.43493000	3.95009500	0.10335800
C	3.17474900	2.80844600	-0.29704400
C	3.36828500	-2.68735400	-0.44727900
C	2.70964600	-3.89739300	-0.11129900
C	1.44823000	-3.77516000	0.52446500
C	0.91014200	-2.53590200	0.81419300
N	2.89654500	5.22176600	-0.10876300
C	2.02284700	6.34803500	0.15054400
C	4.08490500	5.42440000	-0.91255500
C	5.48589500	0.12147700	0.18365400
C	4.05395600	0.15010400	-2.52081400
N	3.26123500	-5.12012300	-0.38420200
C	2.47329200	-6.31876100	-0.18106200
C	4.47496500	-5.19725800	-1.17133000
C	-0.38197300	-0.11570600	1.48827000
C	-1.53438000	-0.12055900	0.71055900
C	-2.81429200	-0.16431100	1.30562000
C	-1.77166700	-0.21518800	3.49350800
C	-0.50729900	-0.15283900	2.88641000

C	-7.52259700	-0.69407900	-0.00069100
C	-8.24707300	-0.41001100	-1.15737900
C	-7.64565800	0.15615800	-2.30569600
C	-6.29716000	0.45116100	-2.32541700
C	-5.53265000	0.16690200	-1.16670900
C	-6.16583900	-0.39422700	-0.02437600
C	-4.16314400	0.33060200	-0.86466400
N	-5.20614800	-0.55311300	0.95190200
C	-3.15144300	0.93931800	-1.76021500
C	-3.99764600	-0.14824300	0.49315600
C	-2.91538100	-0.22624000	2.71917800
H	-0.22275500	2.30340500	1.45690400
H	0.56473500	4.53670700	1.05893800
H	4.13237500	2.95342400	-0.80142700
H	4.33509800	-2.73869800	-0.95241100
H	0.88199700	-4.66386100	0.79882500
H	-0.06170600	-2.51396900	1.30826800
H	1.10570300	6.31687500	-0.46326100
H	1.71986500	6.38588100	1.20749800
H	2.55954900	7.27480500	-0.07683100
H	4.96038100	4.93092600	-0.46488400
H	3.96707600	5.03751800	-1.93983600
H	4.29811100	6.49683300	-0.96953300
H	5.37927600	0.08991700	1.27822300
H	6.09304800	-0.74185600	-0.13052000
H	6.03470700	1.03764600	-0.08490400
H	4.65190100	-0.71037300	-2.85962200
H	3.08913600	0.13088300	-3.04915600
H	4.58863600	1.06886800	-2.80848300

H	1.55432800	-6.32341400	-0.79267600
H	3.07453800	-7.19191400	-0.45468500
H	2.17693700	-6.43077100	0.87271800
H	5.30510500	-4.66688000	-0.68117400
H	4.76476600	-6.24764100	-1.27697600
H	4.34809900	-4.76768400	-2.18032500
H	-1.42994000	-0.12367300	-0.37255300
H	-1.85053900	-0.24210100	4.58056900
H	0.39244100	-0.13508300	3.50470700
H	-7.99553800	-1.12584800	0.88059400
H	-9.31556700	-0.62880600	-1.17553100
H	-8.26158300	0.36014900	-3.18125500
H	-5.82489700	0.88751000	-3.20645000
H	-5.37578300	-0.97645500	1.86055100
H	-2.51136700	1.65104700	-1.22010400
H	-2.49210500	0.16764100	-2.19106100
H	-3.64701200	1.45761000	-2.58935400
H	-3.89334400	-0.24519100	3.20106200

Si-RDM-AA (**30**) S_0min in water @ M06-2X/def2-SVP, SMD(water)

1 1

C	-1.683069	-1.303472	0.273494
C	-2.952616	-1.385909	-0.394005
Si	-3.893235	0.168262	-0.878550
C	-2.764894	1.545100	-0.273742
C	-1.525125	1.248295	0.388468
C	-1.045637	-0.075919	0.623469
C	-1.045369	-2.541675	0.598314
C	-1.596670	-3.759531	0.299564
C	-2.857219	-3.843189	-0.368199
C	-3.506549	-2.609163	-0.697405
C	-3.158954	2.849158	-0.473154
C	-2.369641	3.960312	-0.031988
C	-1.143086	3.663593	0.638319
C	-0.749639	2.365495	0.832085
N	-3.407681	-5.025379	-0.675582
C	-2.720768	-6.264278	-0.337520
C	-4.691034	-5.087964	-1.361446
C	-5.541103	0.239394	0.005195
C	-4.108622	0.256866	-2.735076
N	-2.765631	5.224000	-0.236785
C	-1.946312	6.333912	0.229610
C	-4.016633	5.505307	-0.927624
C	0.284530	-0.183306	1.304964
C	1.444564	-0.149502	0.533969
C	1.644489	-0.373556	3.273519
C	0.370966	-0.302789	2.701799
C	7.102623	-1.383380	-0.909972

C	7.864297	-0.849635	-1.939614
C	7.431680	0.283917	-2.664051
C	6.225024	0.902150	-2.370903
C	5.428465	0.381283	-1.332816
C	5.883984	-0.755278	-0.614237
C	4.156333	0.759138	-0.775464
N	4.939053	-1.047223	0.330669
C	3.322080	1.921991	-1.214250
C	3.896597	-0.144811	0.238012
C	2.823079	-0.332394	2.521181
C	-0.867662	-0.343752	3.553191
C	4.155276	-0.372859	3.222786
C	2.715852	-0.223556	1.118894
H	-0.080027	-2.531768	1.104552
H	-1.059119	-4.664289	0.574991
H	-4.470945	-2.645775	-1.206513
H	-4.104063	3.047274	-0.981216
H	-0.507267	4.467996	1.002022
H	0.194364	2.194528	1.349714
H	-1.737790	-6.318151	-0.827769
H	-2.572960	-6.352131	0.748718
H	-3.326612	-7.108881	-0.677001
H	-5.481400	-4.603571	-0.770064
H	-4.640674	-4.597247	-2.344423
H	-4.960961	-6.137100	-1.508767
H	-5.404605	0.191864	1.095483
H	-6.066313	1.174869	-0.241424
H	-6.177028	-0.603863	-0.305094
H	-4.609009	1.196276	-3.016095

H	-3.136582	0.211294	-3.247426
H	-4.729664	-0.581084	-3.087179
H	-1.809956	6.295086	1.320145
H	-0.954506	6.322241	-0.245488
H	-2.445554	7.272422	-0.025986
H	-4.010321	5.087954	-1.945023
H	-4.874695	5.084406	-0.383475
H	-4.145329	6.589037	-0.996657
H	1.361677	-0.069269	-0.552486
H	1.722300	-0.447447	4.361320
H	7.434853	-2.257480	-0.348260
H	8.817989	-1.314634	-2.195066
H	8.059549	0.674276	-3.466604
H	5.893646	1.776232	-2.935797
H	4.974801	-1.827570	0.976904
H	2.560715	2.178143	-0.464254
H	3.950712	2.810129	-1.377798
H	2.799673	1.716666	-2.162170
H	-0.609667	-0.392564	4.618467
H	-1.490791	0.548061	3.384947
H	-1.485200	-1.221080	3.305132
H	4.866963	0.332199	2.770538
H	4.034724	-0.117398	4.283299
H	4.608733	-1.375063	3.175182

Si-RDM-AA (**30**) S_1min(LE) in water @ M06-2X/def2-SVP, SMD(water)

1 1

C	-1.689198	-1.302002	0.299737
C	-2.953127	-1.377448	-0.394917
Si	-3.867666	0.169022	-0.928741
C	-2.768438	1.536305	-0.268935
C	-1.529794	1.243182	0.413506
C	-1.025683	-0.079376	0.652387
C	-1.076607	-2.555399	0.653906
C	-1.640015	-3.765122	0.356231
C	-2.890932	-3.840860	-0.341598
C	-3.514487	-2.603719	-0.694611
C	-3.170902	2.844182	-0.458175
C	-2.403741	3.958694	0.003848
C	-1.179499	3.666869	0.692340
C	-0.772773	2.375041	0.881630
N	-3.446632	-5.026155	-0.649704
C	-2.788488	-6.268610	-0.277237
C	-4.704042	-5.081750	-1.376932
C	-5.575719	0.244223	-0.159371
C	-4.008273	0.259243	-2.796542
N	-2.807419	5.226360	-0.193232
C	-2.007369	6.339455	0.292996
C	-4.050405	5.503174	-0.894515
C	0.309132	-0.185927	1.318811
C	1.469461	-0.153973	0.545602
C	1.690942	-0.373356	3.282307
C	0.414889	-0.302223	2.716567
C	7.121165	-1.383697	-0.936685

C	7.874791	-0.851973	-1.973397
C	7.435058	0.278502	-2.698228
C	6.229383	0.895646	-2.398387
C	5.441002	0.376872	-1.353032
C	5.903622	-0.756818	-0.634303
C	4.172339	0.754211	-0.787501
N	4.965742	-1.047420	0.318072
C	3.332902	1.914342	-1.223748
C	3.920964	-0.146996	0.230735
C	2.864755	-0.332625	2.521540
C	-0.819718	-0.335198	3.573878
C	4.202072	-0.372015	3.214107
C	2.746552	-0.225291	1.120311
H	-0.123306	-2.550289	1.182373
H	-1.122410	-4.675449	0.653459
H	-4.469605	-2.633575	-1.222526
H	-4.109612	3.037488	-0.980958
H	-0.560073	4.476611	1.074311
H	0.163739	2.206681	1.413363
H	-1.798232	-6.348519	-0.751136
H	-2.655984	-6.333458	0.813165
H	-3.405242	-7.109041	-0.606511
H	-5.509504	-4.587594	-0.812513
H	-4.614997	-4.586361	-2.355903
H	-4.976320	-6.128388	-1.535284
H	-5.518783	0.194219	0.937914
H	-6.082303	1.180611	-0.440389
H	-6.193522	-0.594767	-0.515508
H	-4.496662	1.198839	-3.098342

H	-3.017241	0.213369	-3.271396
H	-4.616032	-0.576811	-3.176040
H	-1.888842	6.293078	1.386131
H	-1.006145	6.335913	-0.163672
H	-2.509761	7.274900	0.033401
H	-4.026145	5.093891	-1.916020
H	-4.908721	5.060615	-0.366172
H	-4.191964	6.585697	-0.951862
H	1.380974	-0.076215	-0.540798
H	1.777089	-0.446131	4.369733
H	7.458828	-2.255499	-0.374609
H	8.827439	-1.316176	-2.234155
H	8.056485	0.667430	-3.506491
H	5.892583	1.767378	-2.963738
H	5.007660	-1.825385	0.966765
H	2.577627	2.172148	-0.468153
H	3.958868	2.802704	-1.396323
H	2.802328	1.704847	-2.166244
H	-0.560436	-0.408758	4.637704
H	-1.425436	0.571945	3.422665
H	-1.457480	-1.193750	3.310431
H	4.911293	0.331764	2.755907
H	4.089084	-0.114051	4.274935
H	4.655491	-1.374300	3.166182

Si-RDM-AA (**30**) S_1min(ET) in water @ M06-2X/def2-SVP, SMD(water)

1 1

C	-1.666033	-1.309267	0.339158
C	-2.918267	-1.377715	-0.346839
Si	-3.814346	0.185052	-0.864192
C	-2.726507	1.559134	-0.199237
C	-1.498923	1.261770	0.471997
C	-1.010422	-0.075870	0.701499
C	-1.063767	-2.556074	0.669209
C	-1.641655	-3.772976	0.359336
C	-2.888968	-3.843641	-0.309272
C	-3.491296	-2.608069	-0.655944
C	-3.129455	2.877322	-0.390965
C	-2.371382	3.985847	0.064314
C	-1.150409	3.689575	0.719259
C	-0.740234	2.383720	0.913668
N	-3.481601	-5.044719	-0.602888
C	-2.725722	-6.267904	-0.422508
C	-4.647405	-5.068029	-1.463375
C	-5.534883	0.259010	-0.113288
C	-3.955600	0.290068	-2.735479
N	-2.795587	5.276455	-0.117903
C	-1.888824	6.366965	0.179143
C	-3.938798	5.534611	-0.970391
C	0.316501	-0.189280	1.361228
C	1.463140	-0.202771	0.591455
C	1.720631	-0.373552	3.339973
C	0.438271	-0.262603	2.773952
C	7.258126	-0.957108	-0.758844

C	7.876614	-0.520831	-1.926433
C	7.212301	0.296954	-2.872339
C	5.906466	0.695744	-2.670351
C	5.247217	0.259827	-1.494890
C	5.941225	-0.550566	-0.557581
C	3.938373	0.465420	-1.000905
N	5.087021	-0.809126	0.488422
C	2.913262	1.328427	-1.640398
C	3.873265	-0.238810	0.259232
C	2.884130	-0.403293	2.584893
C	-0.780319	-0.221148	3.631303
C	4.203725	-0.491621	3.296095
C	2.756461	-0.293945	1.158412
H	-0.104078	-2.571652	1.188000
H	-1.116660	-4.683618	0.643166
H	-4.445020	-2.619252	-1.187486
H	-4.069098	3.063022	-0.915458
H	-0.512185	4.492836	1.083794
H	0.209225	2.227280	1.427620
H	-1.808323	-6.289338	-1.036876
H	-2.431034	-6.404255	0.628288
H	-3.352247	-7.119977	-0.706273
H	-5.479233	-4.497604	-1.024869
H	-4.440262	-4.650073	-2.464337
H	-4.980866	-6.103975	-1.583209
H	-5.490580	0.201180	0.984469
H	-6.037367	1.198721	-0.391061
H	-6.152290	-0.575892	-0.479934
H	-4.442423	1.231848	-3.033483

H	-2.963542	0.247965	-3.209306
H	-4.561082	-0.543425	-3.124564
H	-1.595497	6.365992	1.239151
H	-0.967022	6.324448	-0.427420
H	-2.394685	7.316086	-0.026049
H	-3.780121	5.182146	-2.004756
H	-4.845054	5.046626	-0.582919
H	-4.126344	6.613158	-0.997307
H	1.354776	-0.183805	-0.491373
H	1.804308	-0.421786	4.427678
H	7.777861	-1.581285	-0.032625
H	8.909752	-0.814718	-2.117004
H	7.746707	0.611849	-3.768563
H	5.386326	1.324539	-3.394280
H	5.313960	-1.410777	1.273438
H	2.310781	1.868839	-0.897631
H	3.400329	2.052638	-2.305388
H	2.220053	0.730274	-2.255282
H	-0.529682	-0.297652	4.695712
H	-1.335826	0.714542	3.453992
H	-1.462878	-1.042083	3.356627
H	4.941746	0.209979	2.884117
H	4.067109	-0.269270	4.360777
H	4.623032	-1.508708	3.228132

Carbopyronine-AA S_0min in water @ M06-2X/def2-SVP, SMD(water)

1 1

C	3.412592	1.267247	-0.104537
C	1.984357	1.213455	-0.058665
C	1.309454	-0.025894	-0.015595
C	2.009294	-1.251643	0.008861
C	3.438354	-1.279202	-0.026912
C	4.260899	0.000872	-0.118311
C	4.042366	2.493316	-0.149796
C	3.317411	3.721816	-0.139139
C	1.888558	3.656540	-0.079765
C	1.261069	2.442849	-0.045044
C	1.310114	-2.492661	0.081265
C	1.961430	-3.693503	0.122771
C	3.392069	-3.732873	0.085190
C	4.092684	-2.492851	0.005454
N	3.953885	4.903538	-0.181334
C	-0.182875	-0.038752	-0.000798
C	-0.883939	-0.076295	1.215818
C	-2.278450	-0.081761	1.156787
C	-0.872823	0.000782	-1.213734
C	-7.679285	-1.298256	-1.094130
C	-8.918573	-0.864877	-0.645529
C	-9.034758	0.156604	0.324254
C	-7.909219	0.760867	0.864512
C	-6.637075	0.340819	0.429861
C	-6.542747	-0.683315	-0.548592
C	-5.295256	0.740285	0.763364
N	-5.213826	-0.889679	-0.796907

C	-4.923185	1.813608	1.737788
C	-4.457321	-0.040420	-0.011600
N	4.052173	-4.901714	0.123902
C	-2.982088	-0.050284	-0.056184
C	3.320028	-6.156710	0.219760
C	5.506376	-4.929985	0.076445
C	3.197916	6.147611	-0.147310
C	5.405808	4.956926	-0.263922
C	5.058171	-0.032222	-1.442044
C	5.238277	0.047832	1.076328
H	5.128882	2.526434	-0.191882
H	1.294034	4.567083	-0.067000
H	0.171503	2.413781	-0.003166
H	0.219472	-2.482767	0.107304
H	1.384711	-4.613658	0.181523
H	5.179873	-2.506604	-0.028876
H	-7.586753	-2.085091	-1.844037
H	-9.823102	-1.321522	-1.050906
H	-10.027605	0.470136	0.650839
H	-8.004171	1.547818	1.615963
H	-4.834652	-1.583144	-1.431652
H	-3.888267	2.150430	1.589220
H	-5.584684	2.685399	1.623799
H	-5.016313	1.470949	2.780618
H	2.658226	-6.299826	-0.646988
H	4.037200	-6.981663	0.249952
H	2.708693	-6.191863	1.133336
H	5.883521	-4.469932	-0.848714
H	5.944202	-4.396569	0.933334

H	5.839876	-5.970884	0.107676
H	2.530452	6.233747	-1.017481
H	2.589165	6.217341	0.765785
H	3.899193	6.986541	-0.161667
H	5.873747	4.490765	0.615894
H	5.771576	4.442852	-1.164978
H	5.717927	6.003889	-0.310621
H	4.376086	-0.069737	-2.303647
H	5.686652	0.864429	-1.529805
H	5.710854	-0.915286	-1.470894
H	5.874863	0.940533	1.017219
H	4.685973	0.072171	2.026797
H	5.891939	-0.834433	1.072542
H	-2.842525	-0.125956	2.091319
H	-0.304017	0.049651	-2.145729
C	-0.155631	-0.115264	2.531231
H	-0.861846	-0.096532	3.370681
H	0.457549	-1.026008	2.614395
H	0.525535	0.743844	2.629500
C	-2.269704	-0.001793	-1.272868
C	-2.954635	0.079423	-2.612104
H	-3.299738	-0.908839	-2.953849
H	-3.831781	0.740448	-2.574065
H	-2.259864	0.463300	-3.370020

Carbopyronine-AA S_1min(LE) in water @ M06-2X/def2-SVP, SMD(water)

1 1

C	3.420686	1.261428	-0.104826
C	1.986729	1.208400	-0.044642
C	1.286981	-0.029567	-0.006935
C	2.012883	-1.252205	0.029463
C	3.447893	-1.277513	-0.021675
C	4.281965	-0.001185	-0.132760
C	4.050650	2.487606	-0.155215
C	3.334169	3.723613	-0.134406
C	1.904747	3.662749	-0.052983
C	1.272116	2.451543	-0.012886
C	1.324708	-2.506983	0.124547
C	1.983430	-3.704171	0.165332
C	3.414892	-3.738040	0.106124
C	4.104503	-2.490303	0.009410
N	3.978294	4.905768	-0.185682
C	-0.202912	-0.041821	-0.002151
C	-0.915549	-0.090293	1.210008
C	-2.309978	-0.093968	1.152431
C	-0.900019	0.011760	-1.212430
C	-7.709547	-1.278294	-1.121716
C	-8.949408	-0.849903	-0.669603
C	-9.066388	0.159344	0.312711
C	-7.941164	0.756165	0.862106
C	-6.668582	0.340909	0.424257
C	-6.573477	-0.671008	-0.566907
C	-5.326920	0.735355	0.764386
N	-5.244229	-0.875060	-0.815514

C	-4.955679	1.796134	1.752843
C	-4.487937	-0.036098	-0.018871
N	4.085403	-4.906014	0.142054
C	-3.012513	-0.047477	-0.061352
C	3.364818	-6.163760	0.255589
C	5.536819	-4.927338	0.073284
C	3.231758	6.152435	-0.134040
C	5.426578	4.953386	-0.294646
C	5.063813	-0.037139	-1.464117
C	5.279909	0.048815	1.044104
H	5.137278	2.516462	-0.211819
H	1.313373	4.575863	-0.027336
H	0.183336	2.426200	0.046062
H	0.234587	-2.501663	0.166927
H	1.411322	-4.626799	0.240721
H	5.191751	-2.499128	-0.041785
H	-7.616394	-2.055691	-1.881342
H	-9.853543	-1.301003	-1.082007
H	-10.059495	0.469454	0.641777
H	-8.036884	1.533661	1.623253
H	-4.864456	-1.559786	-1.459243
H	-3.920922	2.135376	1.608712
H	-5.617641	2.669024	1.650286
H	-5.048458	1.440143	2.791263
H	2.686666	-6.310135	-0.598941
H	4.086495	-6.984663	0.276657
H	2.766975	-6.194658	1.179206
H	5.895100	-4.466720	-0.860029
H	5.981850	-4.382181	0.920095

H	5.877431	-5.965567	0.106038
H	2.549660	6.242538	-0.993593
H	2.634503	6.216130	0.787917
H	3.936214	6.988192	-0.154471
H	5.906260	4.482923	0.577621
H	5.771108	4.431498	-1.200501
H	5.743324	5.998289	-0.349096
H	4.372187	-0.077271	-2.318193
H	5.692901	0.858437	-1.564730
H	5.718921	-0.918788	-1.501683
H	5.919411	0.939233	0.973975
H	4.744326	0.077256	2.004180
H	5.935429	-0.832626	1.035580
H	-2.876138	-0.147459	2.085416
H	-0.331676	0.069192	-2.144570
C	-0.186793	-0.139058	2.524807
H	-0.891015	-0.147918	3.366500
H	0.445366	-1.038526	2.591122
H	0.479715	0.730216	2.635724
C	-2.297469	0.013465	-1.275600
C	-2.980133	0.110288	-2.615420
H	-3.324513	-0.873608	-2.970647
H	-3.857540	0.770788	-2.572556
H	-2.283801	0.503244	-3.367406

Carbopyronine-AA S_1min(ET) in water @ M06-2X/def2-SVP, SMD(water)

1 1

C	3.324436	1.347187	-0.067479
C	1.907345	1.234870	-0.080057
C	1.273938	-0.048339	-0.070505
C	2.046190	-1.253386	-0.052406
C	3.466646	-1.207495	-0.045933
C	4.238995	0.116883	-0.049810
C	3.908208	2.608681	-0.072557
C	3.152436	3.807305	-0.073751
C	1.742579	3.682600	-0.097370
C	1.150896	2.433709	-0.095024
C	1.427036	-2.527826	-0.022856
C	2.152883	-3.703841	-0.001514
C	3.568116	-3.671670	-0.018883
C	4.186216	-2.396813	-0.027136
N	3.763888	5.038357	-0.051247
C	-0.193479	-0.124354	-0.070985
C	-0.917186	-0.119345	1.161374
C	-2.294342	-0.135872	1.107794
C	-0.899648	-0.176114	-1.289984
C	-7.734321	-0.935793	-1.335379
C	-8.940537	-0.599860	-0.738484
C	-9.006327	0.115081	0.486697
C	-7.858724	0.505547	1.140539
C	-6.607894	0.169948	0.561171
C	-6.570605	-0.534855	-0.673238
C	-5.274382	0.390946	0.971273
N	-5.259011	-0.706842	-1.019143

C	-4.878827	1.158775	2.182667
C	-4.442113	-0.184869	-0.051729
N	4.314348	-4.826687	-0.026987
C	-3.012185	-0.185075	-0.117498
C	3.650500	-6.088794	0.232032
C	5.740748	-4.743927	0.217120
C	2.963542	6.219707	-0.304923
C	5.192940	5.120409	-0.278115
C	5.145926	0.156854	-1.297481
C	5.115849	0.177200	1.218695
H	4.994910	2.673706	-0.069688
H	1.106929	4.565741	-0.116307
H	0.060361	2.374004	-0.113125
H	0.336298	-2.587599	-0.012576
H	1.618001	-4.651305	0.026502
H	5.273485	-2.341990	-0.019865
H	-7.691064	-1.480057	-2.278245
H	-9.871121	-0.889987	-1.228543
H	-9.982981	0.353699	0.907835
H	-7.901452	1.052810	2.083236
H	-4.947887	-1.241712	-1.822688
H	-3.996079	1.784689	1.997854
H	-5.708945	1.800422	2.503169
H	-4.639346	0.483339	3.019703
H	2.879408	-6.295131	-0.523884
H	4.388077	-6.896860	0.182192
H	3.167682	-6.116878	1.225328
H	6.245546	-4.149750	-0.558016
H	5.975856	-4.292189	1.197444

H	6.165174	-5.753016	0.190216
H	2.484134	6.199529	-1.300035
H	2.171212	6.333854	0.448645
H	3.606211	7.104521	-0.247911
H	5.752383	4.577092	0.496861
H	5.489157	4.710733	-1.260374
H	5.500178	6.170609	-0.234059
H	4.542235	0.116784	-2.216198
H	5.743737	1.078892	-1.314263
H	5.839972	-0.695228	-1.300591
H	5.712698	1.099853	1.234510
H	4.489619	0.152387	2.122895
H	5.809231	-0.674790	1.252889
H	-2.845192	-0.172243	2.045193
H	-0.325520	-0.162663	-2.218612
C	-0.177148	-0.103286	2.462989
H	-0.870945	-0.114773	3.312229
H	0.493412	-0.973119	2.535237
H	0.455446	0.795366	2.530027
C	-2.280690	-0.221099	-1.359404
C	-2.930971	-0.237592	-2.712859
H	-3.311070	-1.241493	-2.962809
H	-3.768172	0.470597	-2.775683
H	-2.192414	0.025399	-3.478811

Phenyl-Cy3-AA S_0min in water @ M06-2X/def2-SVP, SMD(water)

1 1

C	-3.495142	-0.791230	-0.052659
C	-2.635885	0.313897	-0.035039
C	-3.179466	1.604032	-0.028810
C	-3.117567	-2.134992	-0.006126
C	-2.464517	2.803387	-0.063710
C	-1.155303	0.120884	-0.018592
C	-0.455964	0.155800	1.190229
C	0.924171	-0.021802	1.207734
C	1.634979	-0.225258	0.014063
C	0.920921	-0.264049	-1.195049
C	-0.459810	-0.096491	-1.210942
C	3.093525	-0.401612	0.028470
N	3.714192	-1.204994	-0.909984
C	5.064827	-1.200270	-0.697503
C	5.316483	-0.348262	0.409741
C	4.047400	0.157453	0.861357
C	6.097033	-1.867038	-1.373869
C	7.393600	-1.658423	-0.926988
C	7.667019	-0.807236	0.167836
C	6.644414	-0.152332	0.837362
C	-1.883492	-3.948714	0.125036
C	-3.211583	-4.359052	0.043448
N	-3.960786	-3.187224	-0.040222
N	-3.003618	4.039234	-0.023601
C	-1.974018	4.974867	-0.104426
C	-0.799233	4.231722	-0.184873
C	-0.818415	-4.823751	0.220078

C	-1.150816	-6.181567	0.231956
C	-2.483525	-6.610272	0.151609
C	-3.546207	-5.708975	0.055577
C	-1.943673	6.365441	-0.114742
C	-0.681876	6.957372	-0.206458
C	0.492558	6.195087	-0.284380
C	0.458054	4.797624	-0.275007
C	-5.406006	-3.085735	-0.145131
C	-4.425118	4.319273	0.084501
C	3.855926	1.137175	1.977637
H	-4.569118	-0.609600	-0.091874
H	-4.264174	1.707763	-0.006734
H	-0.997266	0.313921	2.125069
H	1.452852	-0.025716	2.161369
H	1.452603	-0.411891	-2.136977
H	-1.002751	-0.126559	-2.157599
H	3.230129	-1.764246	-1.604288
H	5.882952	-2.521397	-2.219973
H	8.220282	-2.161297	-1.431629
H	8.700624	-0.668019	0.488641
H	6.861265	0.501011	1.685253
H	0.209103	-4.467026	0.281614
H	-0.354262	-6.922397	0.305932
H	-2.699253	-7.679121	0.164580
H	-4.580506	-6.046036	-0.006426
H	-2.854086	6.961211	-0.055820
H	-0.610336	8.045449	-0.218124
H	1.455326	6.701851	-0.354815
H	1.356667	4.184496	-0.334999

H	-5.819490	-4.097001	-0.203998
H	-5.813814	-2.575787	0.736895
H	-5.676356	-2.530569	-1.052022
H	-4.838315	3.807600	0.962552
H	-4.556346	5.399086	0.202700
H	-4.948184	3.983763	-0.820191
H	4.729122	1.801192	2.052702
H	2.967194	1.763789	1.818290
H	3.739999	0.639961	2.953880
O	-1.129223	2.898423	-0.158077
O	-1.853545	-2.575329	0.092911

Phenyl-Cy3-AA S_1min(LE) in water @ M06-2X/def2-SVP, SMD(water)

1 1

C	-3.422310	-0.204684	-0.256413
C	-2.340014	0.691117	-0.017507
C	-2.719242	2.051166	0.172153
C	-3.426857	-1.592922	-0.176353
C	-1.923231	3.191049	0.144396
C	-0.937940	0.243692	0.018366
C	-0.057179	0.683784	1.023469
C	1.267582	0.267733	1.050592
C	1.771220	-0.597464	0.063600
C	0.890695	-1.048464	-0.936483
C	-0.435582	-0.640428	-0.955219
C	3.173664	-1.025483	0.074412
N	3.536228	-2.254759	-0.445057
C	4.889477	-2.418393	-0.341219
C	5.413744	-1.240973	0.254651
C	4.306967	-0.358810	0.511814
C	5.709472	-3.492922	-0.715180
C	7.072707	-3.365898	-0.490805
C	7.616595	-2.199057	0.092434
C	6.803102	-1.139622	0.465461
C	-2.777757	-3.648879	0.249218
C	-4.100483	-3.716249	-0.216003
N	-4.474888	-2.418873	-0.476171
N	-2.340374	4.473667	0.373664
C	-1.270312	5.320828	0.206112
C	-0.179804	4.501890	-0.126052
C	-2.043824	-4.762965	0.613889

C	-2.701312	-5.990539	0.494163
C	-4.025527	-6.074285	0.029602
C	-4.755424	-4.944756	-0.335854
C	-1.120574	6.706508	0.306803
C	0.155435	7.211070	0.063156
C	1.238414	6.377539	-0.266610
C	1.090747	4.991410	-0.369108
C	-5.759732	-1.967375	-0.974120
C	-3.697222	4.846912	0.723217
C	4.418642	1.020838	1.083711
H	-4.394730	0.232786	-0.493196
H	-3.781687	2.255519	0.319005
H	-0.426620	1.347623	1.807913
H	1.913772	0.594415	1.865820
H	1.255672	-1.708966	-1.725746
H	-1.093863	-0.992513	-1.752425
H	2.884305	-2.952743	-0.786283
H	5.286418	-4.390774	-1.167774
H	7.739102	-4.183338	-0.771297
H	8.694264	-2.136326	0.251711
H	7.229426	-0.242382	0.919374
H	-1.017785	-4.675496	0.969119
H	-2.173355	-6.904742	0.767116
H	-4.497662	-7.054353	-0.047500
H	-5.781706	-5.014589	-0.694671
H	-1.957931	7.356191	0.559298
H	0.318392	8.287354	0.130212
H	2.217404	6.822334	-0.447438
H	1.916307	4.327528	-0.623082

H	-6.375210	-2.844971	-1.193449
H	-6.261799	-1.348957	-0.217655
H	-5.616768	-1.381327	-1.891447
H	-4.014628	4.294463	1.617428
H	-3.719582	5.920702	0.931649
H	-4.378045	4.620985	-0.108778
H	5.394180	1.458727	0.827846
H	3.636156	1.686008	0.691923
H	4.337264	1.026416	2.182403
O	-0.602195	3.207646	-0.159421
O	-2.387169	-2.344129	0.262624

Phenyl-Cy3-AA S_1min(ET) in water @ M06-2X/def2-SVP, SMD(water)

1 1

C	2.549734	-2.509599	0.053826
C	2.442453	-1.089713	0.037831
C	3.606931	-0.269640	0.027033
C	1.521142	-3.415452	0.064856
C	3.653215	1.099636	0.009436
C	1.108679	-0.463358	0.025772
C	0.456399	-0.175072	-1.198910
C	-0.798394	0.383817	-1.216745
C	-1.467731	0.698759	0.001713
C	-0.800165	0.425042	1.233280
C	0.449288	-0.142534	1.240658
C	-2.767659	1.281215	0.004589
N	-3.323511	1.824341	1.136490
C	-4.578517	2.284529	0.867930
C	-4.865706	2.006035	-0.498726
C	-3.732411	1.378465	-1.057672
C	-5.498113	2.927400	1.704142
C	-6.716043	3.274368	1.142844
C	-7.032548	2.999023	-0.215902
C	-6.127555	2.370185	-1.039347
C	-0.542978	-4.234634	0.074543
C	0.335827	-5.325853	0.087379
N	1.610972	-4.794237	0.081129
N	4.776229	1.904976	-0.002368
C	4.377768	3.227853	-0.018295
C	2.976650	3.214708	-0.016043
C	-1.914282	-4.367470	0.075655

C	-2.414606	-5.681802	0.090852
C	-1.551982	-6.778664	0.103902
C	-0.156926	-6.626240	0.102433
C	5.069728	4.434084	-0.034735
C	4.302638	5.609065	-0.048637
C	2.907595	5.579760	-0.046255
C	2.209463	4.359046	-0.029739
C	2.862209	-5.513559	0.092085
C	6.125688	1.392715	0.002836
C	-3.642558	0.889159	-2.459583
H	3.554726	-2.935269	0.059264
H	4.574249	-0.774996	0.033061
H	0.968456	-0.400343	-2.135522
H	-1.262048	0.614910	-2.171481
H	-1.291457	0.630105	2.184264
H	0.944918	-0.367577	2.186131
H	-2.849885	1.952182	2.025577
H	-5.263724	3.137783	2.747136
H	-7.461041	3.773783	1.764045
H	-8.008616	3.294245	-0.600814
H	-6.364819	2.158002	-2.082587
H	-2.566173	-3.494049	0.065431
H	-3.493109	-5.841557	0.092451
H	-1.969466	-7.786477	0.115555
H	0.509384	-7.488538	0.112485
H	6.159001	4.467122	-0.036887
H	4.816859	6.571063	-0.061843
H	2.346232	6.514322	-0.057512
H	1.120789	4.309061	-0.027800

H	2.652335	-6.588237	0.103095
H	3.452916	-5.273883	-0.803895
H	3.448560	-5.254540	0.985577
H	6.303369	0.765121	-0.882413
H	6.825351	2.234959	-0.012379
H	6.307713	0.793511	0.906710
H	-4.630004	0.932871	-2.933680
H	-2.955302	1.510890	-3.054268
H	-3.278090	-0.146345	-2.502846
O	2.545088	1.912203	0.000903
O	0.189869	-3.074869	0.061391

Phenyl-Cy5-AA S_0min in water @ M06-2X/def2-SVP, SMD(water)

1 1

C	1.218491	0.013217	-0.021864
C	1.926439	1.225379	-0.035585
C	1.393168	2.508859	-0.008051
C	4.073866	4.819143	-0.032521
C	3.005551	5.712422	0.011241
O	3.698139	-3.427767	-0.012803
C	4.248882	-4.688820	-0.027705
C	3.211647	-5.618242	-0.065438
N	2.035974	-4.868825	-0.071078
C	0.675104	-5.377457	-0.102093
C	5.585148	-5.038887	-0.009610
C	5.851795	-6.411230	-0.032479
C	4.818576	-7.358106	-0.071051
C	3.472975	-6.983792	-0.088330
C	5.397388	5.214618	-0.056278
C	5.617208	6.595223	-0.032967
C	4.552427	7.506159	0.011261
C	3.220513	7.085995	0.034360
C	-1.007096	0.587649	-1.043646
C	-2.398144	0.565603	-1.036030
C	-3.101360	-0.066364	0.002946
C	-2.364468	-0.681369	1.027719
C	-0.972869	-0.650269	1.019611
C	-4.570083	-0.090422	0.009724
N	-5.277454	-0.076371	-1.178168
C	-6.618852	-0.092189	-0.915623
C	-6.773973	-0.101390	0.495374

C	-5.456554	-0.096988	1.073689
C	-5.160661	-0.052620	2.541107
C	-7.718702	-0.099973	-1.786163
C	-8.984074	-0.107986	-1.217782
C	-9.161113	-0.108451	0.184570
C	-8.072037	-0.105022	1.042994
C	1.974131	-1.170132	-0.015127
C	1.489512	-2.472751	-0.039703
C	2.361299	-3.563719	-0.041336
C	2.225888	3.630068	-0.011047
C	-0.269649	-0.015561	-0.014208
O	3.566399	3.540169	-0.046530
N	1.856043	4.923244	0.021667
C	0.478712	5.384679	0.064174
H	3.017571	1.140056	-0.058005
H	0.319211	2.689600	0.027695
H	0.714471	-6.470765	-0.098718
H	0.126779	-5.030267	0.782586
H	0.166407	-5.033709	-1.011569
H	6.372393	-4.286550	0.020507
H	6.887853	-6.750501	-0.020178
H	5.070467	-8.418949	-0.088246
H	2.671357	-7.721357	-0.118610
H	6.209736	4.489679	-0.090733
H	6.641062	6.969631	-0.049492
H	4.767984	8.574985	0.028427
H	2.394947	7.796319	0.068995
H	-0.481130	1.083442	-1.862310
H	-2.942494	1.057266	-1.844694

H	-2.883972	-1.207297	1.829154
H	-0.419821	-1.133065	1.828118
H	-4.859411	-0.118500	-2.101391
H	-4.223038	0.481053	2.750017
H	-5.970503	0.465413	3.074602
H	-5.073338	-1.059389	2.979975
H	-7.578342	-0.097108	-2.867917
H	-9.861560	-0.112965	-1.866635
H	-10.172874	-0.113601	0.593201
H	-8.215242	-0.109375	2.125718
H	3.061302	-1.042055	-0.001417
H	0.422613	-2.692121	-0.068266
H	0.479564	6.478738	0.056076
H	-0.065687	5.015014	-0.813764
H	-0.009043	5.027163	0.979796

Phenyl-Cy5-AA S_1min(LE) in water @ M06-2X/def2-SVP, SMD(water)

1 1

C	-1.382686	-0.020525	-0.012848
C	-2.075323	-1.254430	-0.065484
C	-1.484863	-2.517020	0.075376
C	-3.953825	-5.028363	-0.218455
C	-2.831888	-5.838880	0.000623
O	-3.692043	3.598400	0.149447
C	-4.138036	4.891401	0.152075
C	-3.042533	5.741878	-0.048812
N	-1.940964	4.917081	-0.168931
C	-0.563337	5.318841	-0.382373
C	-5.431889	5.347705	0.319715
C	-5.599508	6.735847	0.278125
C	-4.512395	7.600089	0.076897
C	-3.210598	7.125979	-0.091533
C	-5.227034	-5.531178	-0.409148
C	-5.344288	-6.924688	-0.373294
C	-4.229647	-7.749273	-0.156328
C	-2.949099	-7.228369	0.035126
C	0.835996	-0.673935	-0.990571
C	2.225326	-0.644439	-0.988929
C	2.929285	0.058626	0.003952
C	2.193785	0.742772	0.986035
C	0.803526	0.717855	0.978383
C	4.396329	0.083823	0.005162
N	5.100835	-0.029231	-1.179233
C	6.442365	0.006779	-0.921451
C	6.601091	0.130176	0.483778

C	5.285677	0.176062	1.063632
C	4.993933	0.251661	2.530485
C	7.540093	-0.056944	-1.792655
C	8.806395	-0.005297	-1.229539
C	8.987124	0.107733	0.168176
C	7.900723	0.175329	1.026625
C	-2.120969	1.187106	0.032761
C	-1.575893	2.470682	-0.101666
C	-2.351090	3.626179	-0.047538
C	-2.217229	-3.699705	0.012432
C	0.099522	0.006441	-0.006673
O	-3.554840	-3.720133	-0.207385
N	-1.763924	-4.974988	0.144965
C	-0.377804	-5.328891	0.385214
H	-3.162112	-1.206144	-0.174204
H	-0.414532	-2.622930	0.260480
H	-0.514173	6.411755	-0.383399
H	0.067535	4.926356	0.426256
H	-0.204137	4.934642	-1.346536
H	-6.261587	4.659004	0.474194
H	-6.598535	7.153977	0.404720
H	-4.688562	8.676052	0.050300
H	-2.370491	7.801408	-0.249892
H	-6.078682	-4.873129	-0.577221
H	-6.324873	-7.378991	-0.518226
H	-4.366685	-8.831066	-0.136942
H	-2.086084	-7.872283	0.201912
H	0.309182	-1.223272	-1.773132
H	2.769285	-1.187235	-1.764258

H	2.713782	1.319957	1.750650
H	0.251284	1.253655	1.752737
H	4.681475	-0.056344	-2.102499
H	4.064214	-0.274913	2.787209
H	5.812213	-0.208633	3.102359
H	4.894231	1.290730	2.882676
H	7.397006	-0.146769	-2.870288
H	9.682390	-0.053515	-1.878597
H	9.999990	0.144000	0.572354
H	8.046517	0.266642	2.105120
H	-3.206333	1.098349	0.130214
H	-0.508137	2.615215	-0.275292
H	-0.311337	-6.414205	0.506172
H	0.243706	-5.014894	-0.464329
H	-0.022393	-4.837183	1.300450

Phenyl-Cy5-AA S_1min(ET) in water @ M06-2X/def2-SVP, SMD(water)

1 1

C	1.536402	0.128517	-0.048897
C	2.105367	1.405897	-0.106898
C	1.371262	2.621251	-0.167142
C	3.553149	5.394417	-0.282850
C	2.326371	6.068003	-0.326474
O	3.842032	-3.494615	0.115473
C	4.269834	-4.799318	0.170492
C	3.151502	-5.640969	0.207146
N	2.043830	-4.810540	0.174002
C	0.651849	-5.194227	0.193097
C	5.566537	-5.266279	0.189438
C	5.726420	-6.660906	0.248314
C	4.620681	-7.512275	0.284606
C	3.307091	-7.020943	0.264852
C	4.770150	6.041161	-0.304672
C	4.729885	7.443778	-0.374952
C	3.514251	8.128779	-0.419119
C	2.283968	7.455695	-0.396304
C	-0.653465	-0.073927	-1.269263
C	-2.028701	-0.155110	-1.276509
C	-2.760698	-0.123625	-0.056961
C	-2.042149	-0.027413	1.164851
C	-0.664594	0.039887	1.160188
C	-4.187729	-0.200577	-0.075058
N	-4.882082	-0.617933	-1.171436
C	-6.229963	-0.560285	-0.929156
C	-6.414857	-0.055524	0.389188

C	-5.140408	0.173407	0.945886
C	-4.885903	0.746524	2.291987
C	-7.302986	-0.906332	-1.748583
C	-8.576083	-0.725374	-1.220929
C	-8.790743	-0.215426	0.085776
C	-7.729307	0.123431	0.895236
C	2.274895	-1.057696	0.006724
C	1.716395	-2.363886	0.064254
C	2.470245	-3.504617	0.116325
C	1.956987	3.857005	-0.220703
C	0.054981	0.029493	-0.052194
O	3.315994	4.042396	-0.217930
N	1.348542	5.088123	-0.285936
C	-0.084276	5.266099	-0.311313
H	3.197881	1.462828	-0.105287
H	0.279669	2.607559	-0.174036
H	0.584760	-6.285124	0.257919
H	0.144823	-4.751951	1.062705
H	0.146980	-4.857242	-0.723787
H	6.414532	-4.582583	0.160306
H	6.732559	-7.080702	0.265358
H	4.777881	-8.590766	0.329526
H	2.448746	-7.691863	0.292609
H	5.706585	5.485172	-0.269360
H	5.665817	8.002721	-0.394852
H	3.516364	9.218231	-0.473555
H	1.338577	7.996659	-0.432330
H	-0.099505	-0.076705	-2.209209
H	-2.550462	-0.206336	-2.232467

H	-2.567820	-0.048684	2.115786
H	-0.118032	0.097344	2.102692
H	-4.469100	-0.997533	-2.018830
H	-4.122542	1.535558	2.255516
H	-5.811634	1.167236	2.701425
H	-4.530811	-0.028963	2.989308
H	-7.147887	-1.294060	-2.754776
H	-9.441631	-0.981052	-1.833717
H	-9.812522	-0.093335	0.444541
H	-7.885504	0.514458	1.901363
H	3.364749	-0.962873	0.005372
H	0.633509	-2.501905	0.068374
H	-0.309115	6.336319	-0.363172
H	-0.517765	4.765355	-1.189058
H	-0.539020	4.848375	0.598467

BODIPY-AA (**11**) S_0min in toluene @ M06-2X/def2-SVP, SMD(toluene)

0 1

C	2.180184	1.068862	0.622603
N	3.404282	1.314458	0.008424
B	4.266680	0.269085	-0.753438
N	3.448408	-1.052902	-0.752597
C	2.220297	-1.250790	-0.127735
C	1.594297	-0.203496	0.558755
C	1.743825	2.284274	1.239931
C	2.731731	3.223789	0.967669
C	3.741516	2.594206	0.207022
C	3.823020	-2.192601	-1.344038
C	2.834120	-3.176638	-1.118433
C	1.820430	-2.607031	-0.358307
F	4.493764	0.692661	-2.057173
F	5.480087	0.081385	-0.099058
C	0.283300	-0.439731	1.226329
C	-0.902637	-0.185538	0.537112
C	-2.148493	-0.394682	1.148790
C	-2.178138	-0.862632	2.471719
C	-0.993593	-1.126954	3.154971
C	0.239545	-0.917703	2.539481
C	-3.389932	-0.113386	0.414135
N	-3.438172	0.923686	-0.500411
C	-4.684454	0.984869	-1.062886
C	-5.451139	-0.068739	-0.502707
C	-5.215054	1.866996	-2.014702
C	-6.529836	1.668502	-2.407050
C	-7.307122	0.619181	-1.870228

C	-6.781601	-0.247857	-0.925620
C	0.495946	2.547890	2.024013
C	0.579813	-3.310882	0.094968
C	4.998831	3.183077	-0.334130
C	5.094281	-2.315082	-2.111457
H	2.739470	4.265018	1.282696
H	2.871852	-4.200345	-1.484482
H	-0.852159	0.159170	-0.498815
H	-3.137293	-0.994485	2.973523
H	-1.031842	-1.487465	4.184026
H	1.170293	-1.118145	3.073754
H	-2.701622	1.604849	-0.635193
H	-4.614319	2.675708	-2.433338
H	-6.972953	2.336252	-3.147741
H	-8.337701	0.494642	-2.206093
H	-7.390014	-1.054229	-0.510706
H	0.389420	1.857162	2.872096
H	0.513217	3.574076	2.413506
H	-0.406250	2.432615	1.405741
H	0.580598	-4.341803	-0.282025
H	0.508040	-3.345135	1.191350
H	-0.328925	-2.811093	-0.269654
H	5.135537	4.203442	0.042132
H	5.863903	2.568953	-0.046808
H	4.968880	3.206939	-1.433364
H	5.941816	-1.964415	-1.505773
H	5.263818	-3.356835	-2.406928
H	5.058752	-1.687242	-3.013857
C	-4.609828	-0.762814	0.437268

C	-5.003759	-1.981132	1.212801
H	-4.128681	-2.580382	1.499416
H	-5.555085	-1.727673	2.132760
H	-5.661754	-2.621878	0.607499

BODIPY-AA (**11**) S_1min(LE) in toluene @ M06-2X/def2-SVP, SMD(toluene)

0 1

C	2.307047	1.051775	1.025788
N	3.552606	1.322262	0.462286
B	4.242568	0.527526	-0.669936
N	3.288501	-0.639356	-1.011578
C	2.071784	-0.924845	-0.394999
C	1.550121	-0.080954	0.622462
C	2.008814	2.106992	1.970062
C	3.106379	2.956357	1.941935
C	4.042658	2.455163	1.002607
C	3.556996	-1.599366	-1.918424
C	2.503456	-2.548052	-1.906258
C	1.567068	-2.159127	-0.958173
F	4.435551	1.341706	-1.785261
F	5.475403	0.038327	-0.241402
C	0.238935	-0.377775	1.248462
C	-0.941596	-0.217192	0.515780
C	-2.193144	-0.504785	1.080111
C	-2.245609	-0.956445	2.408125
C	-1.072730	-1.125487	3.141027
C	0.165353	-0.838563	2.570135
C	-3.419349	-0.329042	0.288097
N	-3.509649	0.693645	-0.638838
C	-4.729822	0.650113	-1.257178
C	-5.433783	-0.458575	-0.721134
C	-5.286278	1.481282	-2.239436
C	-6.562277	1.175879	-2.687470

C	-7.276101	0.070715	-2.175502
C	-6.725378	-0.746206	-1.200798
C	0.780143	2.296803	2.799518
C	0.322788	-2.908286	-0.604255
C	5.351470	3.024390	0.600552
C	4.794126	-1.594673	-2.735641
H	3.235174	3.868119	2.522612
H	2.461224	-3.438847	-2.530573
H	-0.877225	0.119749	-0.522019
H	-3.212768	-1.152169	2.872549
H	-1.126120	-1.479259	4.171890
H	1.084612	-0.976660	3.142858
H	-2.824760	1.431272	-0.745839
H	-4.734048	2.333582	-2.637998
H	-7.024121	1.801939	-3.452748
H	-8.277724	-0.137919	-2.554571
H	-7.285912	-1.595944	-0.805443
H	0.733136	1.590299	3.643208
H	0.770732	3.313964	3.213932
H	-0.140434	2.151064	2.215775
H	0.371546	-3.920162	-1.028684
H	0.187226	-2.994267	0.483641
H	-0.584190	-2.423434	-0.999504
H	5.575578	3.926972	1.180265
H	6.154613	2.283908	0.745319
H	5.348993	3.269743	-0.474231
H	5.682835	-1.656276	-2.086120
H	4.797351	-2.435961	-3.437907
H	4.884427	-0.648068	-3.292447

C	-4.582447	-1.075694	0.262665
C	-4.910839	-2.315830	1.034119
H	-4.003475	-2.836388	1.369945
H	-5.525101	-2.101305	1.923630
H	-5.483186	-3.015292	0.407007

BODIPY-AA (**11**) S_1min(ET) in toluene @ M06-2X/def2-SVP, SMD(toluene)

0 1

C	2.162305	1.007861	0.760850
N	3.350037	1.309694	0.119052
B	4.157295	0.357966	-0.787390
N	3.424646	-0.998876	-0.800481
C	2.235285	-1.283912	-0.154644
C	1.584625	-0.284287	0.618552
C	1.741653	2.159166	1.473794
C	2.721813	3.147555	1.226973
C	3.692798	2.600680	0.395955
C	3.840165	-2.106427	-1.480269
C	2.915532	-3.124655	-1.274943
C	1.889292	-2.629105	-0.439752
F	4.240509	0.880437	-2.090398
F	5.464481	0.206708	-0.298431
C	0.272686	-0.570720	1.245657
C	-0.896758	-0.261309	0.563678
C	-2.169510	-0.496416	1.129898
C	-2.252574	-1.066300	2.423433
C	-1.092382	-1.366353	3.114508
C	0.160896	-1.121073	2.535074
C	-3.345325	-0.123088	0.390277
N	-3.298723	0.779029	-0.619382
C	-4.542908	0.928537	-1.192269
C	-5.435560	0.051323	-0.511345
C	-4.956538	1.746690	-2.235232
C	-6.299981	1.665919	-2.600392

C	-7.208781	0.797598	-1.950824
C	-6.794672	-0.011712	-0.914199
C	0.531734	2.342053	2.341807
C	0.705274	-3.414728	0.040369
C	4.935597	3.222549	-0.150063
C	5.096433	-2.112735	-2.286922
H	2.726922	4.161703	1.623217
H	2.980314	-4.130101	-1.687587
H	-0.803384	0.138934	-0.447807
H	-3.219411	-1.224135	2.896978
H	-1.152868	-1.782216	4.120718
H	1.074213	-1.357568	3.084374
H	-2.477770	1.321565	-0.870069
H	-4.266162	2.414908	-2.748616
H	-6.660620	2.290487	-3.418638
H	-8.246491	0.772496	-2.281932
H	-7.491078	-0.684108	-0.412149
H	0.480118	1.606030	3.159745
H	0.553613	3.339967	2.802585
H	-0.416506	2.261001	1.784314
H	0.741626	-4.432183	-0.374341
H	0.677620	-3.511334	1.138200
H	-0.256115	-2.970151	-0.263586
H	4.994312	4.276910	0.148983
H	5.832871	2.700393	0.213956
H	4.955509	3.161545	-1.248404
H	5.969769	-1.880294	-1.659672
H	5.243754	-3.096328	-2.750919
H	5.060024	-1.348594	-3.077923

C	-4.703480	-0.615103	0.491480
C	-5.229037	-1.656747	1.411511
H	-4.539093	-2.507959	1.488832
H	-5.375738	-1.251769	2.425971
H	-6.200021	-2.023846	1.058072

HPF-AA(OH) (**12**) S_0min in toluene @ M06-2X/def2-SVP, SMD(toluene)

0 1

C	2.925772	-1.182302	-0.030436
C	4.335355	-1.141208	-0.016949
O	5.000319	0.031270	-0.025835
C	4.358892	1.227571	-0.053481
C	2.901909	1.252877	-0.043247
C	2.199113	0.073095	-0.033743
C	5.114362	2.353088	-0.081111
C	4.497488	3.677714	-0.092292
C	3.017867	3.697100	-0.042212
C	2.282901	2.567211	-0.016589
C	2.316963	-2.452305	-0.063621
C	3.068269	-3.612385	-0.056130
C	4.473483	-3.537311	-0.016926
C	5.108694	-2.296925	-0.003720
C	0.712276	0.049996	-0.024107
O	5.246923	-4.633154	0.001429
O	5.147418	4.711375	-0.128062
C	0.020579	-0.455741	1.084597
C	-1.369752	-0.479466	1.102586
C	-2.109867	0.002555	0.011105
C	-1.411700	0.497908	-1.101907
C	-0.020831	0.522196	-1.119822
C	-5.784344	0.063155	0.490068
C	-4.468394	0.169040	1.064065
C	-3.578233	-0.019487	0.022906
N	-4.280926	-0.212441	-1.153196
C	-5.625018	-0.184416	-0.897343

C	-4.175959	0.488506	2.497119
C	-6.721876	-0.353705	-1.754026
C	-7.988039	-0.258791	-1.197733
C	-8.169065	-0.001609	0.178767
C	-7.083143	0.160095	1.024008
H	6.201864	2.284775	-0.089574
H	2.549955	4.682777	-0.018172
H	1.194526	2.617831	0.033773
H	1.229422	-2.519119	-0.102081
H	2.578253	-4.587414	-0.085361
H	6.195432	-2.228376	0.011867
H	4.698209	-5.428155	-0.014773
H	-1.889974	-0.899555	1.963869
H	0.506348	0.916697	-1.990822
H	-3.859341	-0.470608	-2.036359
H	-3.194309	0.967608	2.614487
H	-4.932572	1.180668	2.894713
H	-4.191497	-0.409944	3.135182
H	-6.583016	-0.549020	-2.818428
H	-8.863448	-0.383759	-1.837202
H	-9.181844	0.067799	0.578543
H	-7.230757	0.353457	2.088567
H	-1.964933	0.892718	-1.956560
H	0.580669	-0.835930	1.941497

HPF-AA(OH) (**12**) S_1min(LE) in toluene @ M06-2X/def2-SVP, SMD(toluene)

0 1

C	2.928447	-1.134822	-0.041303
C	4.361521	-1.063245	-0.033585
O	4.989972	0.124805	-0.027362
C	4.302249	1.284417	-0.011805
C	2.891897	1.319957	0.003543
C	2.165305	0.058956	-0.014788
C	5.087085	2.432365	0.009260
C	4.483473	3.770744	0.068491
C	3.030206	3.781400	0.140031
C	2.302460	2.622303	0.113690
C	2.386848	-2.453705	-0.144953
C	3.189752	-3.568736	-0.168964
C	4.601592	-3.449195	-0.102539
C	5.180245	-2.188228	-0.050652
C	0.699569	0.037355	0.002379
O	5.408104	-4.521137	-0.109960
O	5.190330	4.777740	0.069851
C	-0.011170	-0.757981	0.923751
C	-1.398926	-0.775683	0.942650
C	-2.143786	-0.010494	0.028249
C	-1.438481	0.787146	-0.892121
C	-0.052191	0.820673	-0.897655
C	-5.812741	-0.496659	0.169917
C	-4.494486	-1.039584	0.357841
C	-3.608054	-0.023307	0.040013
N	-4.320336	1.097250	-0.350303

C	-5.662541	0.845671	-0.264707
C	-4.193161	-2.451446	0.753520
C	-6.764994	1.670717	-0.527078
C	-8.027743	1.123704	-0.357710
C	-8.199782	-0.213275	0.062816
C	-7.108462	-1.025092	0.326431
H	6.174082	2.345238	-0.010688
H	2.542483	4.752979	0.225820
H	1.216842	2.683812	0.195511
H	1.308024	-2.575335	-0.230279
H	2.741965	-4.561499	-0.259364
H	6.263056	-2.070317	-0.032742
H	4.885731	-5.332479	-0.160120
H	-1.916942	-1.358382	1.704765
H	0.466405	1.433764	-1.637179
H	-3.907776	2.004522	-0.527047
H	-3.222077	-2.784190	0.361674
H	-4.178198	-2.583694	1.847648
H	-4.962189	-3.129953	0.356309
H	-6.633284	2.702851	-0.855180
H	-8.907289	1.738861	-0.554893
H	-9.209984	-0.607443	0.183276
H	-7.249342	-2.056059	0.657654
H	-1.986784	1.368339	-1.636381
H	0.541713	-1.337303	1.665752

HPF-AA(OH) (**12**) S_1min(ET) in toluene @ M06-2X/def2-SVP, SMD(toluene)

0 1

C	2.936405	-1.192578	0.101573
C	4.354500	-1.102200	0.064682
O	4.980568	0.092042	-0.077860
C	4.283936	1.268756	-0.199275
C	2.850952	1.256646	-0.171835
C	2.178982	0.019868	-0.016555
C	5.016058	2.414024	-0.343700
C	4.385175	3.716161	-0.482573
C	2.934370	3.692983	-0.455325
C	2.221758	2.527231	-0.307969
C	2.390643	-2.483032	0.255474
C	3.196929	-3.615668	0.364910
C	4.585941	-3.489438	0.323144
C	5.163264	-2.221006	0.171532
C	0.705601	-0.022595	-0.000093
O	5.424605	-4.549163	0.423308
O	5.038704	4.768654	-0.615807
C	-0.012536	0.102142	1.208368
C	-1.393819	0.096655	1.223721
C	-2.124248	-0.045545	0.017862
C	-1.403736	-0.182631	-1.196544
C	-0.024939	-0.167437	-1.201799
C	-5.781923	0.196235	0.439410
C	-4.496825	0.420144	0.983204
C	-3.560407	-0.063566	0.007071
N	-4.266980	-0.540837	-1.059935

C	-5.615084	-0.415746	-0.835399
C	-4.219820	1.084645	2.285858
C	-6.699218	-0.783012	-1.629936
C	-7.967324	-0.514753	-1.126353
C	-8.163175	0.101718	0.133495
C	-7.089543	0.461328	0.919492
H	6.104727	2.359663	-0.358398
H	2.426231	4.653324	-0.557580
H	1.130508	2.571130	-0.293955
H	1.305800	-2.598050	0.291074
H	2.744260	-4.603006	0.484678
H	6.246482	-2.109204	0.137916
H	4.906454	-5.356433	0.524081
H	-1.915574	0.169295	2.175079
H	0.523203	-0.247046	-2.141686
H	-3.858220	-1.007626	-1.861663
H	-3.420779	1.833430	2.197898
H	-5.123141	1.586815	2.652986
H	-3.912910	0.353992	3.051433
H	-6.561925	-1.252859	-2.603569
H	-8.840016	-0.784602	-1.722709
H	-9.179435	0.291272	0.478502
H	-7.237992	0.934885	1.890763
H	-1.935855	-0.256551	-2.146006
H	0.545170	0.203605	2.140464

O-RDM-AA (**13**) S_0min in toluene @ M06-2X/def2-SVP, SMD(toluene)

0 1

C	-1.585864	1.400264	-0.001594
C	-3.007098	1.435062	0.001950
O	-3.717332	0.300855	0.002260
C	-3.142016	-0.917083	-0.002239
C	-1.712438	-1.027110	0.016214
C	-0.934483	0.139146	0.012974
C	-0.927625	2.661288	-0.059101
C	-1.624156	3.838922	-0.075935
C	-3.061473	3.852798	-0.039822
C	-3.734200	2.612758	-0.009172
C	-3.996582	-1.992377	-0.016582
C	-3.446806	-3.299423	-0.003016
C	-2.010223	-3.447216	0.058607
C	-1.190129	-2.358603	0.069076
N	-3.744201	5.017775	-0.047338
C	-3.042867	6.286000	-0.117739
C	-5.195149	4.998276	-0.006931
N	-4.247745	-4.372558	-0.034908
C	-3.700848	-5.716472	-0.023893
C	-5.692566	-4.176917	-0.076918
C	0.547982	0.066547	0.019014
C	1.283079	0.614286	1.079089
C	2.672168	0.551369	1.083331
C	2.625016	-0.598576	-1.036658
C	1.235883	-0.541166	-1.039796
C	7.965151	-0.101821	-1.808708
C	9.232045	-0.216650	-1.258284

C	9.418769	-0.368147	0.133263
C	8.338001	-0.401812	0.999339
C	7.038672	-0.280983	0.471228
C	6.872875	-0.141767	-0.930336
C	5.727654	-0.267154	1.065080
N	5.528749	-0.063651	-1.175084
C	5.440165	-0.447520	2.523148
C	4.833828	-0.118464	0.020337
C	3.367105	-0.058141	0.026376
H	0.161555	2.686796	-0.101563
H	-1.072122	4.774731	-0.124213
H	-4.818095	2.524376	-0.006235
H	-5.083583	-1.776459	-0.036036
H	-1.567535	-4.439892	0.109236
H	-0.110864	-2.499714	0.131059
H	-2.439212	6.363735	-1.035673
H	-3.776820	7.097846	-0.124610
H	-2.379810	6.430756	0.749599
H	-5.560507	4.479264	0.892661
H	-5.567478	6.027106	0.012937
H	-5.615281	4.491447	-0.890085
H	-3.142243	-5.918802	0.903415
H	-4.524653	-6.433628	-0.089726
H	-3.030730	-5.884703	-0.880977
H	-5.997403	-3.584839	-0.951835
H	-6.183873	-5.154646	-0.095934
H	-6.049527	-3.592308	0.783030
H	0.758802	1.094532	1.907699
H	3.228456	1.005465	1.903706

H	3.140770	-1.091550	-1.863051
H	0.674716	-0.967892	-1.873479
H	7.821929	0.012065	-2.884257
H	10.103889	-0.191190	-1.914142
H	10.432102	-0.457026	0.527666
H	8.490312	-0.513110	2.074902
H	5.109967	0.143027	-2.073117
H	4.439026	-0.867457	2.691617
H	6.169486	-1.137716	2.971702
H	5.506657	0.501025	3.080257
Cl	-7.276478	-1.263232	-0.085008

O-RDM-AA (**13**) S_1min(LE) in toluene @ M06-2X/def2-SVP, SMD(toluene)

0 1

C	-1.583444	1.395587	-0.040849
C	-3.014992	1.427473	-0.019996
O	-3.730621	0.288160	-0.005052
C	-3.129621	-0.925352	-0.009354
C	-1.715738	-1.046373	-0.006799
C	-0.902752	0.143769	-0.019975
C	-0.949490	2.670782	-0.135603
C	-1.659797	3.846104	-0.151917
C	-3.091520	3.849274	-0.081728
C	-3.747122	2.596051	-0.029370
C	-3.993219	-2.001634	-0.000245
C	-3.457531	-3.324374	0.027382
C	-2.035791	-3.481794	0.074601
C	-1.212560	-2.381314	0.062943
N	-3.793784	5.008255	-0.083047
C	-3.111898	6.282676	-0.183894
C	-5.241224	4.971256	-0.011887
N	-4.290875	-4.384684	0.019827
C	-3.761279	-5.730590	0.048862
C	-5.732750	-4.178263	-0.007640
C	0.568171	0.068385	-0.004625
C	1.318211	0.758870	0.964347
C	2.706592	0.700951	0.975579
C	2.657551	-0.747119	-0.949880
C	1.269610	-0.687634	-0.961205
C	8.000968	-0.326364	-1.797774

C	9.269310	-0.365233	-1.238779
C	9.457180	-0.334889	0.160063
C	8.376579	-0.258946	1.024368
C	7.076546	-0.211065	0.486937
C	6.909449	-0.255002	-0.921135
C	5.765744	-0.124915	1.075073
N	5.564569	-0.212871	-1.171857
C	5.477498	-0.116640	2.543986
C	4.870091	-0.115386	0.021221
C	3.403568	-0.055990	0.020043
H	0.136579	2.711782	-0.213854
H	-1.114369	4.784446	-0.230815
H	-4.830316	2.495868	-0.009626
H	-5.078887	-1.775966	-0.010591
H	-1.589546	-4.472350	0.133850
H	-0.134744	-2.530840	0.124613
H	-2.538248	6.359550	-1.121892
H	-3.853823	7.087067	-0.170208
H	-2.419396	6.437084	0.659150
H	-5.580376	4.447248	0.895768
H	-5.626822	5.995034	0.013759
H	-5.673656	4.454889	-0.884563
H	-3.188473	-5.912378	0.973510
H	-4.589428	-6.443832	0.004538
H	-3.094201	-5.911218	-0.809606
H	-6.041972	-3.593477	-0.885953
H	-6.230158	-5.152474	-0.011058
H	-6.073980	-3.583273	0.851711
H	0.798049	1.345061	1.724703

H	3.262476	1.266780	1.724158
H	3.171748	-1.354086	-1.698117
H	0.713983	-1.224480	-1.732923
H	7.856498	-0.352996	-2.878870
H	10.140452	-0.421604	-1.893651
H	10.470992	-0.368934	0.561890
H	8.529843	-0.229584	2.105194
H	5.142169	-0.123642	-2.087491
H	4.468651	-0.493466	2.761387
H	6.194267	-0.758737	3.076778
H	5.561471	0.892644	2.978738
Cl	-7.295366	-1.235770	-0.043592

O-RDM-AA (**13**) S_1min(ET) in toluene @ M06-2X/def2-SVP, SMD(toluene)

0 1

C	-1.574407	1.438570	0.093374
C	-2.994261	1.457700	0.096828
O	-3.708669	0.309936	0.086707
C	-3.113364	-0.915935	0.040504
C	-1.694086	-1.022320	0.041050
C	-0.914675	0.171593	0.069905
C	-0.937841	2.698443	0.110191
C	-1.649887	3.888576	0.126473
C	-3.067428	3.887659	0.126334
C	-3.722387	2.633686	0.110394
C	-3.959566	-2.003505	-0.001752
C	-3.415267	-3.313621	-0.052295
C	-2.000478	-3.448104	-0.031944
C	-1.176612	-2.335392	0.005813
N	-3.786566	5.054494	0.139691
C	-3.099035	6.322100	0.145799
C	-5.231057	5.005547	0.122033
N	-4.229745	-4.409420	-0.110220
C	-3.654420	-5.728602	-0.022474
C	-5.661547	-4.256048	0.080272
C	0.555601	0.100517	0.050697
C	1.295629	0.027057	1.251376
C	2.672926	-0.052014	1.235321
C	2.635066	0.029730	-1.203526
C	1.259856	0.096532	-1.177231
C	7.949603	0.349618	-1.758597

C	9.213735	0.080753	-1.243870
C	9.406778	-0.427984	0.064648
C	8.333300	-0.677061	0.890266
C	7.028498	-0.407414	0.399732
C	6.865330	0.094408	-0.923637
C	5.748659	-0.531356	0.976029
N	5.519224	0.250515	-1.145253
C	5.467830	-1.066376	2.334738
C	4.809740	-0.097141	-0.034087
C	3.379162	-0.047516	0.004661
H	0.153462	2.737530	0.109470
H	-1.100602	4.827725	0.137855
H	-4.806231	2.538520	0.103472
H	-5.040697	-1.795596	-0.038019
H	-1.541576	-4.434325	-0.050011
H	-0.094179	-2.479067	0.012712
H	-2.466851	6.453923	-0.749691
H	-3.835774	7.132769	0.160552
H	-2.453083	6.435447	1.033540
H	-5.633512	4.474166	1.000435
H	-5.625993	6.027253	0.132736
H	-5.614010	4.499281	-0.779785
H	-3.135447	-5.904356	0.938757
H	-4.452494	-6.473062	-0.119402
H	-2.930873	-5.907355	-0.833884
H	-6.088349	-3.453615	-0.541557
H	-6.154675	-5.201425	-0.176967
H	-5.912253	-3.999044	1.126005
H	0.757762	0.041519	2.200163

H	3.214141	-0.075785	2.177807
H	3.143547	-0.006918	-2.168126
H	0.691289	0.134480	-2.107404
H	7.818065	0.736962	-2.768372
H	10.087199	0.265769	-1.870507
H	10.420828	-0.621415	0.413278
H	8.476335	-1.066743	1.898721
H	5.121733	0.662851	-1.981882
H	4.637663	-1.785365	2.323964
H	6.356678	-1.570338	2.732377
H	5.203678	-0.256649	3.033796
Cl	-7.328189	-1.317550	-0.576198

Si-RDM-AA (**15**) S_0min in toluene @ M06-2X/def2-SVP, SMD(toluene)

0 1

C	1.486299	-1.032064	0.030925
C	2.930606	-1.095425	0.019470
Si	3.987455	0.465215	-0.055354
C	2.667808	1.821638	-0.076356
C	1.270002	1.516184	-0.049814
C	0.749153	0.173953	-0.003561
C	0.785084	-2.284463	0.079417
C	1.427615	-3.489695	0.120919
C	2.859102	-3.546358	0.113164
C	3.578465	-2.305104	0.058190
C	3.088618	3.136986	-0.116762
C	2.178893	4.234266	-0.131650
C	0.789818	3.923561	-0.106077
C	0.365926	2.617055	-0.066706
N	3.515020	-4.709153	0.153313
C	2.787505	-5.964731	0.216354
C	4.975734	-4.728032	0.132267
C	4.993045	0.528131	-1.629294
C	5.053969	0.636726	1.469392
N	2.614577	5.512277	-0.166781
C	1.662591	6.606695	-0.180290
C	4.036858	5.801132	-0.189084
C	-0.744834	0.053354	0.008999
C	-1.443543	-0.005236	1.218966
C	-2.831467	-0.114795	1.228391
C	-2.846709	-0.099373	-1.182382
C	-1.458885	0.001685	-1.192443

C	-8.078546	-1.501487	-1.481247
C	-9.367503	-1.261941	-1.030209
C	-9.611174	-0.467570	0.111217
C	-8.566143	0.097296	0.824814
C	-7.245678	-0.132666	0.395254
C	-7.023683	-0.924290	-0.760465
C	-5.958282	0.283688	0.887430
N	-5.671229	-0.973040	-0.965555
C	-5.732991	1.186079	2.060391
C	-5.022602	-0.264551	0.030314
C	-3.556953	-0.155981	0.027714
H	-0.304690	-2.288240	0.083538
H	0.836946	-4.402863	0.157275
H	4.679308	-2.316024	0.045031
H	4.159818	3.343423	-0.137397
H	0.045866	4.717337	-0.115977
H	-0.708291	2.435112	-0.046041
H	2.149958	-6.009899	1.112425
H	2.152925	-6.102894	-0.672580
H	3.504657	-6.789541	0.261024
H	5.374726	-4.231448	-0.762942
H	5.400488	-4.176691	0.982785
H	5.314074	-5.768205	0.159908
H	4.367627	0.371149	-2.520484
H	5.505727	1.498102	-1.725644
H	5.755785	-0.266027	-1.554832
H	5.819112	-0.156789	1.416820
H	5.563035	1.613299	1.481783
H	4.465602	0.535281	2.393226

H	1.007158	6.558724	-1.064165
H	1.029365	6.600205	0.720916
H	2.208293	7.554924	-0.209170
H	4.521119	5.370081	-1.079219
H	4.180016	6.885936	-0.211790
H	4.541870	5.404744	0.705613
H	-0.892586	0.022102	2.161343
H	-3.358923	-0.194982	2.179612
H	-3.389423	-0.109839	-2.129850
H	-0.921833	0.051968	-2.141897
H	-7.890925	-2.112889	-2.365208
H	-10.211543	-1.695409	-1.569251
H	-10.639970	-0.301000	0.434050
H	-8.762085	0.705750	1.710212
H	-5.204875	-1.547152	-1.656604
H	-4.772614	1.714651	1.986973
H	-6.527067	1.945252	2.115528
H	-5.743096	0.636762	3.015715
Cl	6.971936	-2.016853	-0.032712

Si-RDM-AA (**15**) S_1min(LE) in toluene @ M06-2X/def2-SVP, SMD(toluene)

0 1

C	1.477423	-1.030853	0.048555
C	2.919006	-1.098193	0.021974
Si	3.976568	0.448992	-0.082010
C	2.672334	1.809557	-0.078995
C	1.263229	1.517703	-0.043234
C	0.717498	0.189793	0.006572
C	0.780363	-2.282995	0.119973
C	1.429140	-3.489945	0.169936
C	2.857665	-3.555235	0.145214
C	3.566617	-2.321060	0.066211
C	3.109054	3.122941	-0.112891
C	2.218113	4.233290	-0.114494
C	0.819366	3.940540	-0.086476
C	0.373740	2.645088	-0.052195
N	3.509578	-4.738911	0.193333
C	2.771412	-5.980262	0.287993
C	4.963727	-4.769559	0.154629
C	4.973343	0.508373	-1.667906
C	5.103299	0.632599	1.404039
N	2.669675	5.510298	-0.139193
C	1.735277	6.618362	-0.139792
C	4.093682	5.779148	-0.160374
C	-0.772876	0.068976	0.016601
C	-1.482687	0.008300	1.222309
C	-2.870903	-0.102702	1.229961
C	-2.879676	-0.087262	-1.179996

C	-1.491430	0.014297	-1.184346
C	-8.107610	-1.499825	-1.495421
C	-9.399200	-1.260279	-1.051344
C	-9.648831	-0.462616	0.086270
C	-8.607418	0.105593	0.802934
C	-7.284741	-0.124061	0.380466
C	-7.056682	-0.919466	-0.771726
C	-5.999877	0.295849	0.876443
N	-5.703231	-0.967264	-0.970085
C	-5.780174	1.202268	2.047374
C	-5.059561	-0.253947	0.025473
C	-3.593543	-0.144385	0.027670
H	-0.309245	-2.287505	0.137876
H	0.835888	-4.401297	0.226623
H	4.662577	-2.333215	0.037475
H	4.183528	3.314016	-0.138663
H	0.088384	4.747189	-0.089128
H	-0.702545	2.475869	-0.027253
H	2.143507	-6.007326	1.193772
H	2.117519	-6.129374	-0.587342
H	3.478299	-6.814404	0.335154
H	5.354426	-4.288394	-0.753075
H	5.402937	-4.226308	1.004617
H	5.297564	-5.811545	0.182084
H	4.338791	0.347678	-2.552066
H	5.479822	1.481048	-1.773637
H	5.746327	-0.275483	-1.616285
H	5.875313	-0.151951	1.349586
H	5.610750	1.610435	1.389728

H	4.546705	0.542420	2.348641
H	1.076027	6.586929	-1.022315
H	1.103005	6.610853	0.762826
H	2.294795	7.558560	-0.160948
H	4.569621	5.347120	-1.055633
H	4.254698	6.861537	-0.170718
H	4.592894	5.360592	0.728536
H	-0.934844	0.036013	2.166716
H	-3.401365	-0.182654	2.179774
H	-3.420215	-0.098180	-2.128970
H	-0.952193	0.063358	-2.132813
H	-7.915144	-2.113890	-2.376530
H	-10.240198	-1.696402	-1.593027
H	-10.679261	-0.295924	0.403904
H	-8.808145	0.716735	1.685448
H	-5.232173	-1.541506	-1.657798
H	-4.817783	1.727657	1.977825
H	-6.572634	1.963791	2.094711
H	-5.797357	0.656791	3.004836
Cl	7.110066	-2.007540	-0.107213

Si-RDM-AA (**15**) S_1min(ET) in toluene @ M06-2X/def2-SVP, SMD(toluene)

0 1

C	1.449112	-1.032185	0.002049
C	2.881273	-1.106816	0.002061
Si	3.944655	0.443374	-0.044621
C	2.657894	1.827050	-0.060261
C	1.257213	1.547325	-0.054038
C	0.717669	0.206531	-0.027200
C	0.753397	-2.273404	0.036086
C	1.400692	-3.494297	0.072157
C	2.815730	-3.565324	0.076307
C	3.526599	-2.334148	0.037697
C	3.105668	3.143379	-0.081534
C	2.232514	4.258563	-0.098393
C	0.846539	3.975787	-0.095436
C	0.387106	2.671032	-0.074228
N	3.478815	-4.756230	0.116229
C	2.737732	-5.991052	0.163191
C	4.930851	-4.768631	0.119930
C	5.001808	0.515484	-1.591181
C	5.037193	0.578405	1.472644
N	2.705537	5.546763	-0.116950
C	1.778087	6.650925	-0.131908
C	4.128986	5.791624	-0.119552
C	-0.757288	0.094520	-0.018667
C	-1.474285	0.061820	1.199754
C	-2.848429	-0.040535	1.215385
C	-2.856771	-0.103191	-1.226411

C	-1.485431	0.011838	-1.231136
C	-8.092420	-1.099079	-1.678843
C	-9.370268	-0.976601	-1.146157
C	-9.601617	-0.481571	0.162115
C	-8.552695	-0.100628	0.968414
C	-7.232738	-0.219701	0.459598
C	-7.031658	-0.709309	-0.863367
C	-5.966759	0.058878	1.014688
N	-5.682047	-0.703922	-1.106853
C	-5.730079	0.634816	2.365566
C	-5.001256	-0.264451	-0.007546
C	-3.577443	-0.130404	-0.000230
H	-0.338115	-2.285782	0.035494
H	0.800607	-4.402959	0.098411
H	4.625732	-2.344358	0.033850
H	4.183153	3.320599	-0.085292
H	0.118284	4.785254	-0.110024
H	-0.694412	2.523291	-0.073087
H	2.092781	-6.051635	1.057486
H	2.093184	-6.119644	-0.724119
H	3.438967	-6.831966	0.195231
H	5.355059	-4.272302	-0.765982
H	5.351171	-4.220168	0.977137
H	5.277113	-5.807907	0.152193
H	4.403292	0.367510	-2.502643
H	5.520687	1.484598	-1.666850
H	5.761216	-0.280989	-1.503865
H	5.796327	-0.219589	1.398634
H	5.556013	1.550096	1.498966

H	4.460688	0.464498	2.402966
H	1.127338	6.631450	-1.023492
H	1.126194	6.654230	0.759054
H	2.337458	7.592864	-0.143626
H	4.620745	5.353046	-1.004334
H	4.308101	6.872387	-0.133922
H	4.619753	5.376918	0.777240
H	-0.915185	0.106441	2.135397
H	-3.366581	-0.100413	2.169466
H	-3.389530	-0.131082	-2.178180
H	-0.936997	0.050281	-2.173374
H	-7.931400	-1.477120	-2.687874
H	-10.225828	-1.267912	-1.757026
H	-10.625900	-0.404987	0.525941
H	-8.725836	0.278527	1.976226
H	-5.247200	-1.075005	-1.944035
H	-4.989621	1.445342	2.337128
H	-6.665731	1.035014	2.773540
H	-5.364051	-0.131473	3.067441
Cl	7.017628	-2.076498	-0.040335

Si-RDM-AA (**28**) S_0min in toluene @ M06-2X/def2-SVP, SMD(toluene)

0 1

C	1.378906	1.510821	0.061443
C	2.776200	1.815708	0.101019
Si	4.094629	0.457916	0.102454
C	3.038130	-1.101117	-0.003230
C	1.594708	-1.035442	-0.033584
C	0.855805	0.169571	0.004839
C	0.474768	2.611505	0.066977
C	0.898517	3.918259	0.103821
C	2.287493	4.228659	0.142737
C	3.196990	3.131338	0.142237
C	3.684690	-2.311594	-0.048222
C	2.964332	-3.550198	-0.133877
C	1.533304	-3.490473	-0.172159
C	0.892307	-2.284707	-0.118666
N	2.722991	5.506979	0.177452
C	1.771191	6.601567	0.175399
C	4.144949	5.795462	0.215108
C	5.066527	0.512785	1.697642
C	5.193699	0.634540	-1.398510
N	3.618687	-4.714150	-0.175366
C	2.890397	-5.966687	-0.276315
C	5.078189	-4.736746	-0.116048
C	-0.639389	0.048667	-0.013171
C	-1.342257	0.025551	-1.229630
C	-2.732533	-0.084520	-1.172039
C	-2.723362	-0.127910	1.255936
C	-1.330194	-0.025875	1.197497

C	-8.008591	-1.829338	1.039726
C	-9.283354	-1.471197	0.627849
C	-9.491944	-0.407069	-0.275773
C	-8.425125	0.315963	-0.786170
C	-7.119008	-0.026248	-0.390006
C	-6.931807	-1.094265	0.524386
C	-5.816590	0.495137	-0.715169
N	-5.585885	-1.205128	0.748677
C	-5.545818	1.656079	-1.619483
C	-4.907739	-0.260826	-0.000932
C	-3.410437	-0.164538	2.597515
C	-0.618828	0.104681	-2.546813
C	-3.435602	-0.163810	0.038786
H	-0.599307	2.427292	0.039885
H	0.154839	4.712370	0.104028
H	4.267949	3.337613	0.174095
H	4.785046	-2.324447	-0.016416
H	0.942232	-4.401712	-0.238346
H	-0.197646	-2.283551	-0.143864
H	1.148085	6.591280	-0.732814
H	1.105795	6.557791	1.052040
H	2.316695	7.549861	0.206189
H	4.618928	5.367167	1.112098
H	4.660076	5.395886	-0.672408
H	4.288347	6.880287	0.235784
H	4.422518	0.349409	2.574327
H	5.831518	-0.280128	1.634392
H	5.575454	1.482910	1.810786
H	5.957043	-0.159872	-1.332764

H	4.624985	0.537192	-2.335046
H	5.703839	1.610640	-1.396505
H	2.234706	-6.118379	0.594979
H	2.274025	-5.995527	-1.187727
H	3.606533	-6.792585	-0.318295
H	5.527035	-4.182817	-0.952417
H	5.454064	-4.245194	0.791742
H	5.414980	-5.777522	-0.139182
H	-3.297078	-0.125789	-2.106475
H	-0.762489	0.012804	2.131042
H	-7.848637	-2.650348	1.740397
H	-10.143431	-2.022814	1.011153
H	-10.510351	-0.154877	-0.575302
H	-8.592966	1.135348	-1.488404
H	-5.141646	-1.926188	1.301641
H	-4.517978	2.025635	-1.503337
H	-5.691380	1.392491	-2.679461
H	-6.229505	2.489456	-1.396962
H	-4.344717	0.413661	2.587638
H	-3.662726	-1.193093	2.901546
H	-2.753162	0.247422	3.374356
H	0.069421	-0.745185	-2.673423
H	-1.326466	0.101356	-3.385665
H	-0.011462	1.020528	-2.610966
Cl	7.075314	-2.026205	0.127013

Si-RDM-AA (**28**) S_1min(LE) in toluene @ M06-2X/def2-SVP, SMD(toluene)

0 1

C	-1.372819	1.510970	-0.051893
C	-2.780960	1.803460	-0.106260
Si	-4.083905	0.441894	-0.134482
C	-3.028775	-1.104385	0.003920
C	-1.588236	-1.035050	0.053213
C	-0.826267	0.183757	0.001479
C	-0.482723	2.637693	-0.037006
C	-0.927320	3.933853	-0.068412
C	-2.325156	4.227215	-0.119707
C	-3.216583	3.117324	-0.138827
C	-3.675530	-2.327630	0.054839
C	-2.966656	-3.559088	0.167840
C	-1.539382	-3.490930	0.228296
C	-0.891226	-2.283875	0.168484
N	-2.775984	5.504710	-0.145504
C	-1.841258	6.612232	-0.126049
C	-4.199338	5.774131	-0.188109
C	-5.045348	0.491496	-1.742324
C	-5.241314	0.634321	1.326821
N	-3.617946	-4.743438	0.216567
C	-2.880241	-5.981605	0.347918
C	-5.070469	-4.777395	0.140013
C	0.665122	0.062304	0.010392
C	1.377495	0.030729	1.223592
C	2.767440	-0.079905	1.167281
C	2.755647	-0.110462	-1.260429

C	1.362013	-0.007610	-1.198738
C	8.038080	-1.823578	-1.058269
C	9.314603	-1.469073	-0.648084
C	9.526907	-0.407787	0.257795
C	8.462244	0.315939	0.772192
C	7.154661	-0.022458	0.377889
C	6.963680	-1.088044	-0.538934
C	5.853771	0.500653	0.706385
N	5.617100	-1.195624	-0.760402
C	5.586115	1.659737	1.614037
C	4.941826	-0.251718	-0.007811
C	3.441360	-0.141345	-2.603279
C	0.652395	0.107929	2.539723
C	3.469475	-0.153192	-0.045041
H	0.593029	2.465525	0.003562
H	-0.196253	4.740236	-0.051381
H	-4.290434	3.309236	-0.179752
H	-4.770601	-2.341858	0.005389
H	-0.946903	-4.399911	0.320235
H	0.198174	-2.283313	0.212617
H	-1.225855	6.602623	0.788177
H	-1.165696	6.582380	-0.996180
H	-2.399731	7.552850	-0.155582
H	-4.661421	5.345478	-1.092194
H	-4.712721	5.352761	0.691365
H	-4.359965	6.856602	-0.197102
H	-4.390698	0.329754	-2.611507
H	-5.816187	-0.295151	-1.705080
H	-5.553075	1.461747	-1.863055

H	-6.010400	-0.152416	1.264731
H	-4.702734	0.553985	2.282737
H	-5.751048	1.610559	1.294381
H	-2.199703	-6.139224	-0.505317
H	-2.279915	-5.996746	1.272534
H	-3.586091	-6.817238	0.383589
H	-5.533363	-4.224603	0.970997
H	-5.437407	-4.309000	-0.784294
H	-5.403459	-5.819549	0.171825
H	3.333460	-0.125725	2.100816
H	0.793934	0.035327	-2.132025
H	7.875112	-2.642553	-1.760656
H	10.172802	-2.021443	-1.034552
H	10.546355	-0.158190	0.556075
H	8.633095	1.133238	1.476182
H	5.169459	-1.913300	-1.315053
H	4.557852	2.029134	1.501241
H	5.734113	1.393943	2.673173
H	6.269255	2.493697	1.391611
H	4.377860	0.433421	-2.592249
H	3.689318	-1.168792	-2.915010
H	2.784335	0.277861	-3.376594
H	-0.058232	-0.726096	2.649100
H	1.355216	0.075903	3.382448
H	0.065402	1.036635	2.612758
Cl	-7.216694	-2.012677	-0.198086

Si-RDM-AA (**28**) S_1min(ET) in toluene @ M06-2X/def2-SVP, SMD(toluene)

0 1

C	-1.350725	1.531517	-0.115533
C	-2.748539	1.825752	-0.103069
Si	-4.049431	0.455181	-0.065724
C	-3.000865	-1.104943	-0.012846
C	-1.568510	-1.043657	-0.031166
C	-0.823009	0.186247	-0.075309
C	-0.468732	2.645798	-0.148073
C	-0.914606	3.955557	-0.163536
C	-2.297587	4.252679	-0.149501
C	-3.182462	3.146637	-0.122553
C	-3.656448	-2.326139	0.043213
C	-2.955839	-3.562936	0.090897
C	-1.540118	-3.504850	0.074016
C	-0.882920	-2.289977	0.014150
N	-2.757171	5.545470	-0.161805
C	-1.818411	6.640164	-0.178250
C	-4.177959	5.805567	-0.147943
C	-5.116371	0.523248	-1.605529
C	-5.128243	0.615873	1.458988
N	-3.629063	-4.747217	0.149726
C	-2.898946	-5.988182	0.207553
C	-5.081164	-4.746653	0.156656
C	0.651306	0.059814	-0.069475
C	1.374937	0.042350	1.160971
C	2.750737	-0.051048	1.106326
C	2.735167	-0.142560	-1.357216

C	1.358000	-0.019450	-1.286125
C	8.081037	-1.428186	-1.303300
C	9.320265	-1.199126	-0.718156
C	9.465375	-0.445927	0.473432
C	8.366914	0.092663	1.107358
C	7.086011	-0.131228	0.542110
C	6.970787	-0.879564	-0.662239
C	5.784752	0.246538	0.941674
N	5.641302	-0.923463	-0.997741
C	5.469209	1.109534	2.112496
C	4.889593	-0.283029	-0.052334
C	3.387444	-0.183182	-2.713704
C	0.638894	0.108478	2.464272
C	3.462173	-0.159043	-0.117617
H	0.611100	2.485366	-0.165034
H	-0.178318	4.757421	-0.190504
H	-4.257911	3.335536	-0.113774
H	-4.755612	-2.326721	0.049060
H	-0.947856	-4.418513	0.104290
H	0.208885	-2.309888	-0.000774
H	-1.159015	6.631305	0.707111
H	-1.175648	6.619185	-1.075501
H	-2.368187	7.587782	-0.179709
H	-4.684360	5.370113	-1.025845
H	-4.662623	5.398256	0.755610
H	-4.345450	6.888145	-0.163161
H	-4.524574	0.363785	-2.519433
H	-5.880488	-0.267432	-1.506877
H	-5.628826	1.495392	-1.685591

H	-5.898204	-0.172742	1.397708
H	-4.545876	0.501607	2.385670
H	-5.634144	1.594435	1.482135
H	-2.263630	-6.135509	-0.683560
H	-2.246643	-6.041889	1.096846
H	-3.607578	-6.821945	0.258118
H	-5.494747	-4.186085	1.009222
H	-5.502574	-4.255285	-0.733358
H	-5.436550	-5.782433	0.199723
H	3.303097	-0.109839	2.042969
H	0.779780	0.027989	-2.211294
H	7.984395	-2.003289	-2.223818
H	10.212075	-1.608746	-1.194491
H	10.462496	-0.296331	0.887039
H	8.472533	0.668969	2.027569
H	5.259991	-1.470550	-1.759698
H	4.628180	1.784248	1.907325
H	5.207065	0.505500	2.996257
H	6.346875	1.712922	2.377646
H	4.304858	0.419510	-2.755244
H	3.635467	-1.214846	-3.015839
H	2.690251	0.200512	-3.467963
H	-0.060080	-0.736336	2.554228
H	1.331414	0.091456	3.315280
H	0.032165	1.024966	2.513872
Cl	-7.140710	-2.034971	-0.019480

Si-RDM-AA (**29**) S_0min in toluene @ M06-2X/def2-SVP, SMD(toluene)

0 1

C	0.931450	1.499362	0.647357
C	2.177621	1.870580	0.050511
Si	3.467121	0.580491	-0.456721
C	2.629289	-1.024950	0.072137
C	1.309343	-1.031237	0.662296
C	0.552201	0.135102	0.913391
C	0.037450	2.554199	0.990355
C	0.331936	3.877668	0.765651
C	1.568032	4.253643	0.167901
C	2.467776	3.202738	-0.174725
C	3.306537	-2.200107	-0.138150
C	2.740207	-3.472257	0.211964
C	1.430351	-3.484705	0.792336
C	0.757894	-2.313725	1.000056
N	1.874175	5.549237	-0.061860
C	0.942397	6.594960	0.315334
C	3.138307	5.904313	-0.680394
C	5.057563	0.866212	0.481497
C	3.716993	0.630568	-2.308052
N	3.421333	-4.600130	-0.005561
C	2.849528	-5.887014	0.350309
C	4.750749	-4.547935	-0.609356
C	-0.805829	-0.055149	1.520305
C	-1.923215	-0.161703	0.689863
C	-3.207892	-0.318494	1.230898
C	-2.232901	-0.294485	3.453713
C	-0.960855	-0.122346	2.908297

C	-7.678221	-1.750392	-0.349520
C	-8.464344	-1.448442	-1.450835
C	-8.028069	-0.538637	-2.438395
C	-6.792562	0.081494	-2.342398
C	-5.968823	-0.209034	-1.238705
C	-6.431124	-1.117312	-0.252264
C	-4.660291	0.236841	-0.836272
N	-5.458100	-1.205903	0.706336
C	-3.831401	1.249022	-1.564198
C	-4.383231	-0.409547	0.353709
C	-3.348360	-0.387603	2.626452
H	-0.921489	2.322515	1.453455
H	-0.396128	4.633228	1.053159
H	3.424783	3.459781	-0.631182
H	4.307070	-2.158989	-0.595345
H	0.955515	-4.423903	1.068767
H	-0.238164	-2.369007	1.438573
H	-0.014532	6.494272	-0.220782
H	0.736734	6.578228	1.397230
H	1.375028	7.568469	0.064790
H	3.993070	5.589570	-0.061080
H	3.242283	5.442019	-1.674239
H	3.184405	6.990985	-0.802767
H	4.913188	0.771989	1.567897
H	5.781852	0.112702	0.127447
H	5.462159	1.867760	0.266410
H	4.490589	-0.118499	-2.549152
H	2.787415	0.403674	-2.850697
H	4.076817	1.621793	-2.625896

H	1.919253	-6.077117	-0.206863
H	3.566440	-6.676163	0.105649
H	2.629286	-5.939322	1.427409
H	5.449087	-3.959278	0.001879
H	5.127876	-5.569020	-0.719866
H	4.731491	-4.050320	-1.588615
H	-1.790088	-0.142497	-0.393268
H	-2.355467	-0.344228	4.536755
H	-0.085813	-0.042177	3.556156
H	-8.017671	-2.451484	0.414413
H	-9.441280	-1.923130	-1.556510
H	-8.676029	-0.326161	-3.290096
H	-6.458621	0.779047	-3.113323
H	-5.460134	-1.853262	1.484491
H	-3.125353	1.757245	-0.892643
H	-3.248329	0.797091	-2.382737
H	-4.476768	2.018537	-2.012827
H	-4.342768	-0.489513	3.065791
Cl	6.330690	-1.747877	-1.639241

Si-RDM-AA (**29**) S_1min(LE) in toluene @ M06-2X/def2-SVP, SMD(toluene)

0 1

C	0.908874	1.487805	0.648972
C	2.168258	1.865705	0.063443
Si	3.479816	0.592423	-0.398830
C	2.633918	-1.018013	0.065191
C	1.307803	-1.040243	0.634446
C	0.519226	0.130273	0.909617
C	0.009097	2.556557	0.982500
C	0.305174	3.874452	0.749961
C	1.548554	4.251444	0.154625
C	2.453375	3.200683	-0.168041
C	3.320212	-2.198693	-0.162479
C	2.762898	-3.474459	0.144269
C	1.445992	-3.497886	0.701216
C	0.760514	-2.332915	0.931284
N	1.848658	5.550810	-0.084833
C	0.914761	6.596557	0.282221
C	3.110659	5.905598	-0.702863
C	5.038056	0.882283	0.601722
C	3.853705	0.687045	-2.232883
N	3.450527	-4.616024	-0.085397
C	2.862412	-5.901237	0.225479
C	4.788297	-4.556248	-0.654178
C	-0.833988	-0.079730	1.510987
C	-1.960534	-0.169989	0.687632
C	-3.243011	-0.349020	1.228119
C	-2.259539	-0.383325	3.445733

C	-0.991176	-0.189685	2.897893
C	-7.712609	-1.771372	-0.361581
C	-8.507880	-1.448729	-1.450622
C	-8.083918	-0.512508	-2.418487
C	-6.851896	0.113724	-2.314978
C	-6.019146	-0.197168	-1.223712
C	-6.469455	-1.132214	-0.256549
C	-4.711252	0.247635	-0.818226
N	-5.489971	-1.236743	0.693702
C	-3.892432	1.281290	-1.526965
C	-4.422359	-0.424905	0.354667
C	-3.378629	-0.458204	2.621659
H	-0.951038	2.322628	1.442210
H	-0.425204	4.632450	1.027487
H	3.416423	3.457761	-0.612940
H	4.326619	-2.142600	-0.593766
H	0.967822	-4.444375	0.948386
H	-0.241751	-2.404298	1.352872
H	-0.041003	6.489052	-0.255822
H	0.704680	6.584372	1.363861
H	1.346269	7.569257	0.027162
H	3.965145	5.607113	-0.073413
H	3.223230	5.420536	-1.685645
H	3.148924	6.989743	-0.847503
H	4.852469	0.775197	1.680767
H	5.795845	0.149790	0.279351
H	5.437812	1.891852	0.415074
H	4.649807	-0.040011	-2.461148
H	2.963124	0.466293	-2.839838

H	4.220707	1.689987	-2.503771
H	1.935537	-6.070246	-0.347478
H	3.573641	-6.692723	-0.029991
H	2.622047	-5.985317	1.298208
H	5.471076	-3.971613	-0.020997
H	5.176265	-5.574401	-0.758513
H	4.787300	-4.067316	-1.639326
H	-1.832455	-0.120740	-0.395324
H	-2.377134	-0.463776	4.527694
H	-0.113880	-0.124940	3.544742
H	-8.042484	-2.492880	0.387408
H	-9.482170	-1.927649	-1.561667
H	-8.738501	-0.284166	-3.261000
H	-6.527574	0.831795	-3.071066
H	-5.481583	-1.903299	1.455454
H	-3.185119	1.776814	-0.847330
H	-3.310904	0.851648	-2.358533
H	-4.544513	2.057388	-1.954244
H	-4.370801	-0.576638	3.062123
Cl	6.556217	-1.654350	-1.533801

Si-RDM-AA (**29**) S_1min(ET) in toluene @ M06-2X/def2-SVP, SMD(toluene)

0 1

C	0.990453	1.569348	0.706580
C	2.235135	1.831176	0.051084
Si	3.344975	0.426654	-0.549294
C	2.504031	-1.093508	0.166913
C	1.229091	-0.996531	0.821534
C	0.526010	0.240552	1.026437
C	0.208390	2.708107	1.049393
C	0.615361	4.004206	0.791312
C	1.853843	4.267115	0.157471
C	2.632523	3.139414	-0.200297
C	3.129273	-2.321152	0.005101
C	2.546990	-3.535788	0.460658
C	1.292276	-3.442217	1.115182
C	0.665140	-2.222067	1.278166
N	2.275223	5.547754	-0.096282
C	1.454773	6.665179	0.302132
C	3.553845	5.772823	-0.729502
C	5.097760	0.634344	0.079036
C	3.342453	0.382201	-2.425438
N	3.172643	-4.734977	0.276137
C	2.605304	-5.932956	0.843555
C	4.506536	-4.775775	-0.296295
C	-0.839798	0.143004	1.618300
C	-1.939769	0.010567	0.774362
C	-3.247733	-0.083902	1.286562
C	-2.360814	0.105931	3.529900

C	-1.064704	0.193254	3.002794
C	-7.722077	-1.355334	-0.326366
C	-8.392764	-1.227037	-1.545596
C	-7.798283	-0.614347	-2.668356
C	-6.511485	-0.113278	-2.602806
C	-5.803843	-0.234397	-1.383148
C	-6.432002	-0.850574	-0.266368
C	-4.500010	0.149195	-0.991775
N	-5.528141	-0.826630	0.780568
C	-3.516140	0.874781	-1.832934
C	-4.366117	-0.254929	0.389820
C	-3.448003	-0.035115	2.685222
H	-0.751070	2.577252	1.551031
H	-0.034394	4.824365	1.093417
H	3.589862	3.300074	-0.700255
H	4.092725	-2.347051	-0.520853
H	0.803163	-4.334470	1.503640
H	-0.296531	-2.217813	1.792394
H	0.469460	6.650373	-0.195058
H	1.280781	6.681250	1.392156
H	1.956751	7.599174	0.027018
H	4.386222	5.361955	-0.133005
H	3.601674	5.315004	-1.731940
H	3.716448	6.850434	-0.841924
H	5.148479	0.576473	1.176623
H	5.702264	-0.172652	-0.370229
H	5.520779	1.600333	-0.240331
H	4.020196	-0.432221	-2.734290
H	2.332825	0.203848	-2.825733

H	3.720078	1.330865	-2.839809
H	1.593627	-6.127199	0.450411
H	3.236682	-6.788979	0.581665
H	2.534485	-5.883038	1.945607
H	5.260927	-4.337772	0.381314
H	4.774737	-5.820280	-0.493497
H	4.575636	-4.206200	-1.234316
H	-1.759162	-0.063692	-0.296767
H	-2.513386	0.160874	4.608332
H	-0.209575	0.306070	3.672311
H	-8.195574	-1.828418	0.533491
H	-9.409958	-1.611696	-1.629297
H	-8.365170	-0.539223	-3.595892
H	-6.044728	0.357923	-3.468499
H	-5.678127	-1.260801	1.685555
H	-2.970893	1.636158	-1.259094
H	-2.767509	0.181693	-2.252257
H	-4.022990	1.360167	-2.676203
H	-4.456648	-0.065069	3.101542
Cl	5.918607	-2.136919	-2.101523

Si-RDM-AA (**30**) S_0min in toluene @ M06-2X/def2-SVP, SMD(toluene)

0 1

C	-1.391217	-1.025958	0.537297
C	-2.707389	-1.028235	-0.060190
Si	-3.547850	0.571232	-0.602041
C	-2.266265	1.868439	-0.093612
C	-1.023182	1.504235	0.513319
C	-0.636425	0.142769	0.786353
C	-0.842423	-2.302742	0.898297
C	-1.513001	-3.477214	0.703024
C	-2.816382	-3.473792	0.107819
C	-3.381015	-2.207107	-0.262956
C	-2.560445	3.198975	-0.324776
C	-1.669206	4.254710	0.024311
C	-0.438304	3.886061	0.637304
C	-0.138763	2.563994	0.864849
N	-3.493963	-4.605441	-0.102941
C	-2.926068	-5.886676	0.278161
C	-4.814572	-4.562612	-0.726223
C	-5.141575	0.855939	0.331450
C	-3.793709	0.610785	-2.454078
N	-1.978281	5.548485	-0.213166
C	-1.054010	6.599126	0.168330
C	-3.236770	5.896049	-0.847071
C	0.722848	-0.039969	1.394185
C	1.830436	-0.156296	0.556260
C	2.189769	-0.227057	3.284481
C	0.891570	-0.079618	2.788646
C	7.302500	-1.955640	-1.069540

C	8.045027	-1.567252	-2.174484
C	7.658097	-0.467778	-2.970622
C	6.516889	0.261225	-2.675025
C	5.740613	-0.110763	-1.562052
C	6.150441	-1.214621	-0.771250
C	4.528720	0.408080	-0.982975
N	5.238185	-1.349291	0.240467
C	3.769564	1.597858	-1.480430
C	4.254021	-0.384906	0.114279
C	3.316432	-0.333664	2.462875
C	-0.287970	0.037040	3.715153
C	4.682760	-0.447827	3.090007
C	3.128722	-0.302525	1.064847
H	0.150239	-2.348360	1.346679
H	-1.041205	-4.412137	0.998629
H	-4.378733	-2.172708	-0.726712
H	-3.514367	3.450470	-0.790711
H	0.282501	4.645874	0.931949
H	0.817137	2.335337	1.336519
H	-1.990753	-6.086064	-0.267328
H	-2.715712	-5.922173	1.357911
H	-3.641254	-6.679260	0.039510
H	-5.522864	-3.966587	-0.133844
H	-4.781271	-4.077898	-1.711614
H	-5.188932	-5.585518	-0.829254
H	-4.998244	0.770561	1.418769
H	-5.550766	1.853896	0.108415
H	-5.861589	0.096249	-0.018067
H	-4.157955	1.598429	-2.777948

H	-2.861811	0.385747	-2.993461
H	-4.563031	-0.143283	-2.693143
H	-0.860048	6.589620	1.252508
H	-0.090951	6.498252	-0.356672
H	-1.486758	7.569807	-0.092803
H	-3.327935	5.429478	-1.840205
H	-4.097233	5.580375	-0.236150
H	-3.285839	6.982083	-0.974130
H	1.684133	-0.142884	-0.526204
H	2.332947	-0.241989	4.368132
H	7.603481	-2.805078	-0.454421
H	8.947523	-2.123271	-2.433949
H	8.268523	-0.193303	-3.832315
H	6.219875	1.107689	-3.297874
H	5.224157	-2.096312	0.922263
H	3.054142	1.963655	-0.731291
H	4.456591	2.423650	-1.720056
H	3.204869	1.369014	-2.398469
H	0.033370	0.046862	4.764519
H	-0.857119	0.958534	3.518739
H	-0.984585	-0.804249	3.575938
H	5.434718	0.122379	2.527343
H	4.661766	-0.073935	4.122078
H	5.026940	-1.493685	3.134248
Cl	-6.404351	-1.774176	-1.772073

Si-RDM-AA (**30**) S_1min(LE) in toluene @ M06-2X/def2-SVP, SMD(toluene)

0 1

C	-1.421191	-1.031988	0.541611
C	-2.732493	-0.989560	-0.059223
Si	-3.521477	0.630035	-0.586704
C	-2.218503	1.885656	-0.057346
C	-0.980815	1.486807	0.559286
C	-0.608493	0.122881	0.813652
C	-0.916139	-2.330419	0.884249
C	-1.627221	-3.482790	0.668000
C	-2.926776	-3.439497	0.072789
C	-3.441952	-2.158043	-0.279773
C	-2.485278	3.226956	-0.274342
C	-1.581566	4.264093	0.092537
C	-0.362114	3.866551	0.722745
C	-0.084737	2.541998	0.941636
N	-3.637558	-4.568536	-0.149279
C	-3.097018	-5.859475	0.218894
C	-4.951976	-4.488660	-0.767756
C	-5.130300	0.959737	0.316801
C	-3.784215	0.707283	-2.440836
N	-1.862230	5.569750	-0.137253
C	-0.924282	6.600327	0.260950
C	-3.105833	5.946028	-0.779335
C	0.747554	-0.109565	1.401647
C	1.858017	-0.197069	0.561114
C	2.221918	-0.426346	3.279008
C	0.926702	-0.230050	2.792260

C	7.281923	-2.007598	-1.196571
C	8.032986	-1.566078	-2.275821
C	7.675541	-0.404649	-2.993570
C	6.555611	0.333946	-2.644202
C	5.771351	-0.091039	-1.556297
C	6.151870	-1.257118	-0.843498
C	4.574417	0.419258	-0.939880
N	5.237382	-1.435040	0.159665
C	3.845283	1.658984	-1.353046
C	4.278940	-0.438650	0.101515
C	3.345706	-0.505230	2.449926
C	-0.249666	-0.137990	3.725374
C	4.712411	-0.674881	3.063934
C	3.155242	-0.392061	1.057048
H	0.073742	-2.414207	1.333357
H	-1.182684	-4.434778	0.953799
H	-4.431654	-2.088035	-0.745799
H	-3.431233	3.500112	-0.745412
H	0.363467	4.613458	1.040086
H	0.856720	2.289707	1.430220
H	-2.160900	-6.074705	-0.322922
H	-2.887355	-5.914914	1.299846
H	-3.824834	-6.637877	-0.030196
H	-5.643962	-3.877766	-0.169640
H	-4.903964	-4.017267	-1.759806
H	-5.359586	-5.499450	-0.868576
H	-5.008892	0.867738	1.406356
H	-5.501307	1.972516	0.091774
H	-5.882064	0.235680	-0.036903

H	-4.118497	1.711926	-2.745455
H	-2.862490	0.465081	-2.990368
H	-4.575929	-0.011406	-2.707456
H	-0.746808	6.583397	1.348494
H	0.045383	6.478399	-0.248121
H	-1.332753	7.579973	-0.005381
H	-3.196402	5.484855	-1.775977
H	-3.977510	5.638227	-0.178786
H	-3.134632	7.033453	-0.898813
H	1.711475	-0.120474	-0.518858
H	2.366673	-0.502847	4.360091
H	7.560375	-2.905013	-0.641911
H	8.918883	-2.128347	-2.575862
H	8.291592	-0.089076	-3.837034
H	6.280950	1.228620	-3.207119
H	5.200740	-2.227981	0.786673
H	3.131204	1.982566	-0.583495
H	4.551404	2.485441	-1.526402
H	3.283118	1.510632	-2.289026
H	0.067052	-0.217751	4.773508
H	-0.782847	0.816308	3.594865
H	-0.978406	-0.938382	3.522083
H	5.467278	-0.071260	2.540920
H	4.697158	-0.375241	4.120097
H	5.050786	-1.722867	3.031376
Cl	-6.581483	-1.576625	-1.853128

Si-RDM-AA (**30**) S_1min(ET) in toluene @ M06-2X/def2-SVP, SMD(toluene)

0 1

C	-1.312882	-1.012847	0.633468
C	-2.602164	-1.079745	0.004390
Si	-3.490409	0.472144	-0.573191
C	-2.288686	1.842337	-0.079558
C	-1.039728	1.550690	0.554124
C	-0.594209	0.211634	0.861021
C	-0.748422	-2.256181	1.040760
C	-1.382269	-3.467818	0.843331
C	-2.648188	-3.530832	0.207066
C	-3.231819	-2.298937	-0.198365
C	-2.645660	3.158433	-0.350205
C	-1.824018	4.266175	-0.026760
C	-0.592825	3.975169	0.608794
C	-0.224451	2.670549	0.882581
N	-3.288363	-4.714781	-0.008221
C	-2.681495	-5.950523	0.418661
C	-4.582571	-4.720860	-0.666866
C	-5.134169	0.695881	0.300750
C	-3.756088	0.467662	-2.429468
N	-2.201263	5.553813	-0.310897
C	-1.331765	6.649980	0.040088
C	-3.473365	5.808139	-0.946594
C	0.769814	0.087646	1.448898
C	1.865060	-0.067021	0.618435
C	2.288936	-0.025390	3.345240
C	0.981899	0.118974	2.850861

C	7.474348	-1.520980	-0.956575
C	8.059034	-1.284259	-2.199032
C	7.420575	-0.510504	-3.193791
C	6.175891	0.044495	-2.969048
C	5.551669	-0.185310	-1.719731
C	6.221540	-0.959212	-0.734418
C	4.303135	0.212210	-1.183671
N	5.407795	-1.008212	0.376956
C	3.320335	1.097123	-1.862227
C	4.243801	-0.341909	0.146111
C	3.397288	-0.198485	2.525937
C	-0.178189	0.303569	3.770803
C	4.755077	-0.318400	3.164902
C	3.181999	-0.207864	1.111562
H	0.224612	-2.272690	1.534142
H	-0.890578	-4.376702	1.187914
H	-4.214951	-2.302419	-0.689691
H	-3.606254	3.342300	-0.835830
H	0.082721	4.779320	0.896885
H	0.735744	2.515588	1.377369
H	-1.708400	-6.123492	-0.073528
H	-2.513820	-5.971795	1.509743
H	-3.342924	-6.785131	0.162428
H	-5.333114	-4.131252	-0.118500
H	-4.542242	-4.265299	-1.668132
H	-4.931470	-5.755855	-0.755155
H	-5.025974	0.631885	1.393982
H	-5.583512	1.670440	0.050765
H	-5.809122	-0.100490	-0.058382

H	-4.159505	1.433941	-2.772446
H	-2.822634	0.264842	-2.975805
H	-4.499967	-0.318029	-2.648946
H	-1.148581	6.699967	1.127522
H	-0.352029	6.574387	-0.462567
H	-1.796079	7.593327	-0.267236
H	-3.542893	5.321931	-1.934337
H	-4.318031	5.447925	-0.335068
H	-3.595506	6.887251	-1.091285
H	1.682465	-0.129744	-0.452981
H	2.442291	0.010888	4.426075
H	7.979258	-2.115308	-0.195279
H	9.042321	-1.707631	-2.408182
H	7.922114	-0.354622	-4.148707
H	5.677788	0.638773	-3.736193
H	5.599212	-1.561727	1.203109
H	2.810979	1.765541	-1.155615
H	3.825010	1.704766	-2.624232
H	2.541062	0.509191	-2.375336
H	0.129257	0.314024	4.823549
H	-0.703395	1.243070	3.534116
H	-0.914747	-0.500883	3.612592
H	5.520320	0.265188	2.634202
H	4.712680	0.038149	4.201095
H	5.089548	-1.368719	3.208189
Cl	-6.332221	-2.016365	-1.802898

C-RDM-AA S_0min in toluene @ M06-2X/def2-SVP, SMD(toluene)

0 1

C	-3.086000	-0.917942	-0.059019
C	-1.648487	-0.986069	-0.034058
C	-0.871272	0.178703	-0.002818
C	-1.464521	1.473027	0.007991
C	-2.880974	1.619692	-0.002431
C	-3.817459	0.418890	-0.039385
C	-3.825410	-2.078282	-0.097187
C	-3.196704	-3.363763	-0.114122
C	-1.761952	-3.426574	-0.067612
C	-1.031251	-2.277251	-0.029915
C	-0.661313	2.645101	0.031171
C	-1.206614	3.902315	0.053171
C	-2.624752	4.064558	0.048613
C	-3.425074	2.892565	0.017871
N	-3.928197	-4.483236	-0.168997
C	0.618537	0.073199	0.012593
C	1.327879	0.142103	1.224397
C	2.719401	0.053065	1.164522
C	1.302905	-0.093699	-1.193127
C	8.158689	0.824416	-1.195753
C	9.379645	0.363093	-0.727476
C	9.455964	-0.597097	0.304385
C	8.308518	-1.110854	0.888128
C	7.054343	-0.659167	0.437334
C	6.999839	0.300850	-0.605335
C	5.697906	-0.966554	0.811001
N	5.677936	0.551922	-0.856759

C	5.286969	-1.961427	1.850430
C	4.888993	-0.196582	-0.001803
N	-3.185754	5.295412	0.070490
C	3.416672	-0.104329	-0.042063
C	-2.347514	6.478381	0.096881
C	-4.629284	5.440743	0.066737
C	-3.285549	-5.785175	-0.206524
C	-5.385269	-4.407332	-0.190281
C	-4.701423	0.508721	-1.307021
C	-4.728922	0.450892	1.211506
H	-4.923164	-2.012016	-0.115718
H	-1.249005	-4.385603	-0.058430
H	0.057057	-2.340087	0.005697
H	0.424757	2.540739	0.030754
H	-0.549403	4.768897	0.071027
H	-4.507809	2.992373	0.008097
H	8.101241	1.564842	-1.995104
H	10.300896	0.749833	-1.166400
H	10.435567	-0.935823	0.645130
H	8.374494	-1.850513	1.688908
H	5.325795	1.250037	-1.498436
H	4.220897	-2.212870	1.768557
H	5.860857	-2.894400	1.742927
H	5.467524	-1.586254	2.870660
H	-1.697841	6.532474	-0.791072
H	-2.984652	7.368174	0.107714
H	-1.709592	6.501734	0.994678
H	-5.076293	4.998744	-0.837604
H	-5.084676	4.959346	0.946489

H	-4.882493	6.505342	0.089198
H	-2.606619	-5.869996	-1.068533
H	-2.710565	-5.977300	0.712659
H	-4.054516	-6.557745	-0.298957
H	-5.778040	-3.878004	0.688708
H	-5.751373	-3.835753	-1.054553
H	-5.791272	-5.423087	-0.220583
H	-4.090225	0.412752	-2.216943
H	-5.473957	-0.275274	-1.277104
H	-5.219312	1.477493	-1.338968
H	-5.498264	-0.332598	1.129522
H	-4.137346	0.315619	2.129488
H	-5.250201	1.416429	1.274957
H	3.291999	0.123280	2.092231
H	0.727500	-0.165477	-2.119844
C	0.613168	0.312558	2.537982
H	1.319602	0.276529	3.377055
H	0.080446	1.275234	2.579512
H	-0.139141	-0.476895	2.685975
C	2.696460	-0.188740	-1.252092
C	3.370156	-0.406404	-2.583242
H	3.750165	0.535193	-3.011017
H	4.221738	-1.095143	-2.495893
H	2.657893	-0.822722	-3.307558
Cl	-7.276374	-1.698821	-0.127120

C-RDM-AA S_1min(LE) in toluene @ M06-2X/def2-SVP, SMD(toluene)

0 1

C	-3.064514	-0.939283	-0.054626
C	-1.633165	-0.992908	-0.030144
C	-0.840130	0.194713	-0.006202
C	-1.472153	1.470104	-0.005298
C	-2.898180	1.594839	-0.015153
C	-3.828366	0.383593	-0.035881
C	-3.796287	-2.114209	-0.085002
C	-3.170705	-3.395808	-0.095318
C	-1.742524	-3.443220	-0.045127
C	-1.015597	-2.282750	-0.011415
C	-0.697891	2.674482	-0.005167
C	-1.270690	3.918538	0.001939
C	-2.693237	4.051595	0.003277
C	-3.465243	2.856971	-0.008421
N	-3.904485	-4.532278	-0.146254
C	0.645726	0.101132	0.006234
C	1.372134	0.299858	1.196546
C	2.763662	0.207278	1.140045
C	1.332758	-0.196905	-1.175207
C	8.196513	0.727224	-1.293493
C	9.418133	0.309941	-0.786495
C	9.495098	-0.550416	0.329695
C	8.347817	-1.005149	0.961219
C	7.093404	-0.595314	0.473156
C	7.038100	0.263046	-0.654969
C	5.737233	-0.863400	0.876891
N	5.715852	0.491381	-0.925775

C	5.325828	-1.754111	2.006674
C	4.927299	-0.171972	-0.002641
N	-3.284894	5.272310	0.010050
C	3.454442	-0.080957	-0.045866
C	-2.475950	6.474130	0.015341
C	-4.729484	5.385998	0.004871
C	-3.251581	-5.822658	-0.195399
C	-5.357455	-4.464906	-0.158950
C	-4.727500	0.455828	-1.291998
C	-4.725485	0.414527	1.223747
H	-4.889814	-2.051467	-0.100711
H	-1.218868	-4.397168	-0.025347
H	0.072389	-2.342081	0.033350
H	0.390247	2.593502	-0.015942
H	-0.631396	4.799348	0.001294
H	-4.550489	2.933387	-0.014210
H	8.138780	1.390131	-2.158210
H	10.338955	0.652798	-1.261389
H	10.474844	-0.857967	0.698535
H	8.414083	-1.667082	1.827366
H	5.362748	1.125728	-1.630088
H	4.257541	-2.003824	1.953514
H	5.892816	-2.697285	1.984335
H	5.514164	-1.286593	2.986639
H	-1.834431	6.530571	-0.879252
H	-3.133184	7.349173	0.023376
H	-1.828732	6.518487	0.906326
H	-5.165955	4.918342	-0.892707
H	-5.173084	4.904157	0.891295

H	-5.006644	6.444515	0.012329
H	-2.566463	-5.892979	-1.055938
H	-2.671024	-6.019641	0.721641
H	-4.010560	-6.604604	-0.296334
H	-5.745597	-3.924710	0.715446
H	-5.732027	-3.925462	-1.041449
H	-5.760185	-5.482739	-0.160828
H	-4.123251	0.377969	-2.208230
H	-5.480476	-0.345287	-1.267898
H	-5.275338	1.408809	-1.319160
H	-5.477402	-0.386623	1.174705
H	-4.119260	0.307694	2.135863
H	-5.272843	1.366669	1.281788
H	3.342133	0.375495	2.051658
H	0.752749	-0.369929	-2.085831
C	0.667282	0.603280	2.491362
H	1.376046	0.630245	3.329304
H	0.151734	1.575796	2.448355
H	-0.100450	-0.154264	2.709410
C	2.725980	-0.296398	-1.234303
C	3.391800	-0.650913	-2.540043
H	3.752197	0.243342	-3.073758
H	4.255046	-1.313284	-2.387173
H	2.679710	-1.155264	-3.206502
Cl	-7.385085	-1.693093	-0.064412

C-RDM-AA S_1min(ET) in toluene @ M06-2X/def2-SVP, SMD(toluene)

0 1

C	-3.042104	-0.943516	-0.086566
C	-1.615923	-1.003446	-0.100904
C	-0.836378	0.190397	-0.062725
C	-1.447154	1.484705	-0.023306
C	-2.864342	1.608614	-0.006124
C	-3.795155	0.390821	-0.029557
C	-3.781602	-2.116260	-0.119975
C	-3.166393	-3.397565	-0.166070
C	-1.749790	-3.450081	-0.174398
C	-1.010378	-2.284344	-0.142619
C	-0.679276	2.674345	0.014645
C	-1.255982	3.930542	0.060860
C	-2.663441	4.066897	0.072758
C	-3.429191	2.877210	0.038909
N	-3.919096	-4.536100	-0.202150
C	0.633917	0.093808	-0.056673
C	1.351555	0.011689	1.174407
C	2.727880	-0.075763	1.122175
C	1.347700	0.096374	-1.271378
C	8.191373	0.515092	-1.339942
C	9.391063	0.115925	-0.763715
C	9.439911	-0.626011	0.442784
C	8.283032	-0.981856	1.100833
C	7.040941	-0.583077	0.544586
C	7.021530	0.150589	-0.674185
C	5.703893	-0.762330	0.962505
N	5.708915	0.381199	-0.998295

C	5.282954	-1.542111	2.158354
C	4.881513	-0.129717	-0.037603
N	-3.265635	5.301103	0.115747
C	3.450494	-0.058255	-0.100237
C	-2.453225	6.491469	0.153020
C	-4.706298	5.401461	0.123569
C	-3.272953	-5.823185	-0.247311
C	-5.367200	-4.443678	-0.195472
C	-4.721254	0.482166	-1.263329
C	-4.668713	0.399906	1.245658
H	-4.877112	-2.045753	-0.106159
H	-1.228299	-4.404967	-0.209821
H	0.079382	-2.359259	-0.156097
H	0.410805	2.605786	0.003640
H	-0.611672	4.807654	0.085908
H	-4.514810	2.951898	0.051183
H	8.168127	1.079736	-2.271555
H	10.326868	0.377671	-1.259487
H	10.409331	-0.915079	0.848021
H	8.315393	-1.550301	2.031126
H	5.402331	0.953569	-1.775174
H	4.364244	-2.112917	1.971691
H	6.077167	-2.241849	2.447690
H	5.096952	-0.881506	3.020825
H	-1.807159	6.578622	-0.738112
H	-3.103207	7.372895	0.184371
H	-1.801258	6.519316	1.043643
H	-5.155368	4.951879	-0.778262
H	-5.150167	4.903479	1.002352

H	-4.993924	6.458216	0.153142
H	-2.639788	-5.937380	-1.144891
H	-2.634541	-5.997433	0.636772
H	-4.035843	-6.608829	-0.271537
H	-5.751618	-3.925830	0.696121
H	-5.753717	-3.859542	-1.044601
H	-5.788193	-5.454902	-0.234042
H	-4.138710	0.399059	-2.193725
H	-5.487127	-0.307790	-1.219871
H	-5.251726	1.445645	-1.275442
H	-5.431828	-0.391960	1.186500
H	-4.046436	0.264441	2.143895
H	-5.201470	1.357835	1.338803
H	3.279093	-0.094556	2.060530
H	0.770766	0.136576	-2.197459
C	0.608266	0.034789	2.475162
H	1.293832	-0.033008	3.329293
H	0.016424	0.958011	2.563286
H	-0.106020	-0.800887	2.520513
C	2.729084	0.032172	-1.340125
C	3.379699	-0.003228	-2.697034
H	3.767614	0.986836	-2.991136
H	4.203483	-0.728000	-2.747184
H	2.635331	-0.280430	-3.452851
Cl	-7.317927	-1.714568	-0.023815

Cy3 -AA S_0min in toluene @ M06-2X/def2-SVP, SMD(toluene)

0 1

C	2.690595	-1.580368	-0.002323
C	1.341137	-1.895050	0.006115
C	0.945509	-3.254194	-0.002601
C	3.251767	-0.285142	0.056780
C	-0.352799	-3.734991	-0.056388
C	0.300387	-0.823017	0.020780
C	-0.281047	-0.420126	1.225039
C	-1.263551	0.564353	1.236383
C	-1.698399	1.155526	0.040053
C	-1.103113	0.746738	-1.164070
C	-0.111042	-0.227273	-1.173654
C	-2.745158	2.186899	0.038000
N	-2.704077	3.215912	-0.885370
C	-3.782385	4.040004	-0.706801
C	-4.553192	3.500393	0.354736
C	-3.879111	2.315215	0.818028
C	-4.157086	5.212253	-1.378097
C	-5.329106	5.833404	-0.974502
C	-6.116713	5.307488	0.072468
C	-5.741089	4.151156	0.737664
C	3.419823	1.887804	0.218786
C	4.712743	1.382826	0.113555
N	4.558514	-0.003494	0.011576
N	-0.716611	-5.044927	-0.050201
C	-2.105571	-5.120057	-0.121553
C	-2.547138	-3.798683	-0.170585
C	3.135676	3.236642	0.340784

C	4.247325	4.080309	0.351084
C	5.555202	3.578380	0.244028
C	5.829564	2.215843	0.122576
C	-3.010484	-6.175071	-0.147166
C	-4.364390	-5.835307	-0.223456
C	-4.792019	-4.503165	-0.272162
C	-3.879277	-3.443497	-0.246521
C	5.625823	-0.987186	-0.124618
C	0.209855	-6.153706	0.024490
C	-4.379972	1.399551	1.891270
H	3.416719	-2.391498	-0.046310
H	1.726869	-4.013162	0.024237
H	0.047121	-0.873233	2.162724
H	-1.683928	0.897572	2.185970
H	-1.438877	1.181023	-2.108060
H	0.339599	-0.538863	-2.118149
H	-1.923216	3.398305	-1.503263
H	-3.550611	5.618135	-2.189035
H	-5.650033	6.747321	-1.477113
H	-7.033012	5.824914	0.360745
H	-6.352341	3.754102	1.550870
H	2.112586	3.601328	0.424738
H	4.094470	5.156191	0.445664
H	6.392499	4.277533	0.256210
H	6.852512	1.810440	0.034704
H	-2.687208	-7.215026	-0.110174
H	-5.107092	-6.633519	-0.245822
H	-5.858560	-4.285022	-0.331935
H	-4.185808	-2.398468	-0.284914

H	6.607794	-0.473422	-0.165263
H	5.608250	-1.665285	0.739157
H	5.476531	-1.557331	-1.051114
H	0.793674	-6.102139	0.953939
H	-0.362137	-7.087198	0.015346
H	0.889913	-6.143849	-0.838586
H	-5.477603	1.334783	1.856572
H	-3.981526	0.381958	1.774877
H	-4.108630	1.752272	2.899482
O	-1.455762	-2.973497	-0.130688
O	2.540591	0.835569	0.180445
Cl	8.703650	0.483848	-0.237312

Cy3-AA S_1min(LE) in toluene @ M06-2X/def2-SVP, SMD(toluene)

0 1

C	-2.713079	1.459870	-0.023957
C	-1.336241	1.806435	-0.030070
C	-1.009221	3.182578	-0.067082
C	-3.266747	0.188008	0.017213
C	0.260494	3.750909	-0.086131
C	-0.269067	0.764738	-0.005625
C	0.254377	0.307561	1.207957
C	1.265823	-0.647693	1.230968
C	1.787978	-1.164978	0.035613
C	1.251771	-0.710798	-1.179902
C	0.235136	0.237743	-1.199120
C	2.864654	-2.165407	0.045095
N	2.910165	-3.145349	-0.929892
C	4.001020	-3.947349	-0.729121
C	4.691162	-3.443111	0.403058
C	3.954276	-2.303299	0.884531
C	4.450355	-5.071352	-1.435875
C	5.615668	-5.680161	-0.995486
C	6.324074	-5.188375	0.122047
C	5.874273	-4.079971	0.822259
C	-3.420317	-1.998112	0.099321
C	-4.731848	-1.491296	0.061416
N	-4.601681	-0.122469	0.011371
N	0.562349	5.089755	-0.120243
C	1.932169	5.232078	-0.132241
C	2.450198	3.926936	-0.103130
C	-3.134290	-3.350200	0.157165

C	-4.240662	-4.204311	0.173746
C	-5.556158	-3.706405	0.134242
C	-5.840782	-2.343609	0.077660
C	2.783703	6.337340	-0.164436
C	4.153848	6.069374	-0.165977
C	4.656576	4.760405	-0.136905
C	3.804822	3.650465	-0.104752
C	-5.666656	0.859707	-0.041187
C	-0.418743	6.150511	-0.140667
C	4.361510	-1.432069	2.031758
H	-3.441600	2.271703	-0.055780
H	-1.831841	3.900330	-0.081034
H	-0.143842	0.699250	2.146255
H	1.640339	-1.016881	2.186615
H	1.653432	-1.088826	-2.122547
H	-0.166186	0.586323	-2.153003
H	2.173729	-3.316187	-1.603086
H	3.904863	-5.450272	-2.301403
H	5.993567	-6.556941	-1.523998
H	7.237755	-5.694271	0.437884
H	6.424365	-3.709880	1.690046
H	-2.106983	-3.711514	0.189691
H	-4.078809	-5.282116	0.219580
H	-6.388295	-4.411874	0.149405
H	-6.866580	-1.949857	0.048597
H	2.403703	7.358134	-0.187030
H	4.854025	6.905387	-0.190548
H	5.735425	4.602255	-0.139921
H	4.172229	2.624831	-0.083084

H	-6.648935	0.354186	-0.033503
H	-5.596926	1.525477	0.831927
H	-5.573283	1.454879	-0.961794
H	-1.047749	6.105895	0.760229
H	0.105023	7.111925	-0.163321
H	-1.053489	6.066387	-1.034770
H	5.456693	-1.334375	2.067788
H	3.938543	-0.421693	1.942559
H	4.041336	-1.844730	3.002210
O	1.416909	3.047132	-0.075206
O	-2.545447	-0.958828	0.070403
Cl	-8.828147	-0.574971	-0.017795

Cy3-AA S_1min(ET) in toluene @ M06-2X/def2-SVP, SMD(toluene)

0 1

C	-2.581480	1.718366	0.009067
C	-1.196273	2.032873	0.011649
C	-0.750956	3.386055	-0.016354
C	-3.155264	0.467333	0.029469
C	0.536833	3.845633	-0.043694
C	-0.207482	0.934468	0.012143
C	0.289422	0.409700	1.227736
C	1.188893	-0.630391	1.235656
C	1.632642	-1.208499	0.014217
C	1.144247	-0.667904	-1.209908
C	0.255746	0.378965	-1.206736
C	2.525061	-2.322733	0.000723
N	2.699129	-3.099854	-1.113995
C	3.614973	-4.086270	-0.867624
C	4.080484	-3.921033	0.468834
C	3.401372	-2.818437	1.029297
C	4.078616	-5.112108	-1.693281
C	5.030645	-5.966730	-1.156189
C	5.519667	-5.819882	0.167884
C	5.059757	-4.811063	0.983053
C	-3.330572	-1.735953	0.070307
C	-4.630380	-1.219992	0.042632
N	-4.491769	0.159981	0.019483
N	0.966181	5.165075	-0.067336
C	2.345904	5.185354	-0.105455
C	2.753426	3.842946	-0.097567
C	-3.056734	-3.088014	0.101975

C	-4.167929	-3.945003	0.102764
C	-5.469495	-3.435921	0.072510
C	-5.739385	-2.061463	0.041638
C	3.288168	6.206451	-0.146322
C	4.640291	5.829966	-0.176705
C	5.031282	4.492979	-0.167530
C	4.076455	3.460754	-0.127304
C	-5.547125	1.148117	-0.012317
C	0.073181	6.292661	-0.054923
C	3.631331	-2.278101	2.397219
H	-3.282945	2.553172	-0.009237
H	-1.524629	4.155656	-0.016281
H	-0.073109	0.830359	2.166268
H	1.519852	-1.041234	2.185796
H	1.501717	-1.058687	-2.163746
H	-0.108360	0.798329	-2.145337
H	2.141401	-3.031853	-1.957816
H	3.712852	-5.233249	-2.712518
H	5.418911	-6.778408	-1.772962
H	6.268108	-6.521203	0.536069
H	5.431103	-4.699162	2.002532
H	-2.032080	-3.458306	0.126681
H	-4.009771	-5.023969	0.127962
H	-6.312376	-4.128974	0.073061
H	-6.763496	-1.657978	0.014894
H	2.994966	7.255996	-0.156145
H	5.402083	6.610074	-0.209323
H	6.091373	4.239604	-0.192741
H	4.356910	2.407944	-0.121093

H	-6.535715	0.651621	-0.024172
H	-5.480480	1.795053	0.876078
H	-5.448990	1.770105	-0.915446
H	-0.557670	6.277847	0.847182
H	0.663855	7.215273	-0.055884
H	-0.577002	6.286677	-0.943499
H	4.557714	-2.690839	2.814050
H	3.713600	-1.182989	2.393611
H	2.809580	-2.552405	3.077938
O	1.648789	3.040796	-0.057463
O	-2.438008	-0.695603	0.061401
Cl	-8.704138	-0.302656	-0.080333

Cy5 -AA S_0min in toluene @ M06-2X/def2-SVP, SMD(toluene)

0 1

C	-0.034688	1.333075	0.010779
C	-0.901643	2.414326	-0.011134
C	-2.307661	2.362349	0.025108
C	-3.570191	5.651188	-0.137680
C	-4.775097	4.955841	-0.026069
O	4.089386	2.356169	0.055850
C	5.461000	2.403034	0.054947
C	5.943677	1.097561	0.003332
N	4.804220	0.285040	-0.026567
C	4.769479	-1.172500	-0.087104
C	6.281418	3.516495	0.096889
C	7.650940	3.248132	0.084064
C	8.145029	1.934233	0.032570
C	7.308458	0.818505	-0.009127
C	-3.496937	7.025954	-0.239570
C	-4.718750	7.708414	-0.225596
C	-5.934000	7.024310	-0.114918
C	-5.989733	5.630798	-0.013026
C	-1.270016	-0.573064	-1.065005
C	-1.748788	-1.879240	-1.058012
C	-1.506166	-2.728852	0.033404
C	-0.764251	-2.228210	1.114415
C	-0.294559	-0.917815	1.107956
C	-2.010686	-4.109125	0.031262
N	-2.071986	-4.825857	-1.150728
C	-2.583042	-6.072478	-0.907948
C	-2.886194	-6.140881	0.475976

C	-2.516642	-4.878526	1.061801
C	-2.716258	-4.499503	2.495971
C	-2.808993	-7.151818	-1.773542
C	-3.358796	-8.302927	-1.230279
C	-3.679301	-8.387604	0.141977
C	-3.448950	-7.321171	0.996532
C	1.360723	1.599802	0.017313
C	2.373224	0.671311	-0.005270
C	3.729624	1.072062	0.006245
C	-3.071341	3.512682	-0.012213
C	-0.539688	-0.068719	0.019962
O	-2.542016	4.745993	-0.127218
N	-4.423750	3.611013	0.053608
C	-5.313694	2.478522	0.191406
H	-0.445991	3.409496	-0.047014
H	-2.832859	1.410702	0.099479
H	5.803568	-1.572279	-0.110703
H	4.249627	-1.558351	0.799674
H	4.237466	-1.482601	-0.996071
H	5.877792	4.527300	0.136869
H	8.353615	4.081865	0.114997
H	9.224111	1.774878	0.024565
H	7.684462	-0.218831	-0.050486
H	-2.540305	7.539735	-0.326243
H	-4.720816	8.795890	-0.302975
H	-6.865618	7.591060	-0.108037
H	-6.940380	5.104976	0.072432
H	-1.466630	0.072858	-1.923683
H	-2.335474	-2.241630	-1.904832

H	-0.534990	-2.880862	1.957890
H	0.280379	-0.545905	1.959000
H	-1.654755	-4.524449	-2.022516
H	-2.767885	-3.409545	2.624022
H	-3.656188	-4.925348	2.877357
H	-1.905343	-4.875746	3.140401
H	-2.562602	-7.086924	-2.834426
H	-3.547664	-9.161560	-1.876793
H	-4.110655	-9.310823	0.531868
H	-3.691811	-7.396576	2.058569
H	1.646502	2.656522	0.034961
H	2.173353	-0.399370	-0.038060
H	-6.341993	2.847418	0.267115
H	-5.231999	1.817671	-0.682866
H	-5.068761	1.913002	1.101117
Cl	7.911085	-2.495189	-0.155962

Cy5-AA S_1min(LE) in toluene @ M06-2X/def2-SVP, SMD(toluene)

0 1

C	0.219545	-1.382522	-0.010655
C	1.291947	-2.308615	-0.084228
C	2.647853	-1.996345	0.069427
C	4.582734	-4.933963	-0.228068
C	5.614368	-4.015388	0.011493
O	-3.736741	-2.984523	0.210341
C	-5.082560	-3.206688	0.225583
C	-5.742223	-1.984606	0.029868
N	-4.741526	-1.038148	-0.102877
C	-4.904474	0.389137	-0.320204
C	-5.748796	-4.405872	0.399127
C	-7.144354	-4.332040	0.368222
C	-7.812500	-3.111460	0.171965
C	-7.136502	-1.904149	-0.002184
C	4.803874	-6.284149	-0.423684
C	6.138638	-6.700562	-0.370963
C	7.180005	-5.793290	-0.132642
C	6.944083	-4.431172	0.063632
C	1.307539	0.664915	-0.975161
C	1.562182	2.030414	-0.962346
C	1.015107	2.857789	0.033828
C	0.191464	2.269269	1.007709
C	-0.066172	0.903598	0.990950
C	1.293703	4.297746	0.043600
N	1.493075	4.977880	-1.145124
C	1.748734	6.296746	-0.883973
C	1.744690	6.456187	0.525356

C	1.453850	5.171579	1.104678
C	1.406372	4.879449	2.571972
C	1.983293	7.371033	-1.753848
C	2.229272	8.611139	-1.185466
C	2.242848	8.789063	0.215429
C	2.003908	7.727288	1.072508
C	-1.104179	-1.871288	0.053746
C	-2.270386	-1.101025	-0.062520
C	-3.543034	-1.662958	0.009052
C	3.650860	-2.960281	0.005058
C	0.489554	0.073291	0.002806
O	3.395885	-4.265596	-0.227929
N	4.994112	-2.788165	0.154775
C	5.626485	-1.515877	0.428684
H	1.025223	-3.361224	-0.212000
H	2.965865	-0.972889	0.273963
H	-5.981199	0.642441	-0.388728
H	-4.451763	0.939723	0.517372
H	-4.404211	0.673826	-1.257165
H	-5.211071	-5.341247	0.549723
H	-7.726408	-5.245316	0.499592
H	-8.903447	-3.103272	0.154147
H	-7.652219	-0.942038	-0.161570
H	3.980957	-6.973826	-0.607695
H	6.372233	-7.755300	-0.519001
H	8.206401	-6.160500	-0.099279
H	7.759346	-3.732029	0.248401
H	1.735019	0.040735	-1.762531
H	2.211424	2.460987	-1.727421

H	-0.271178	2.894829	1.771580
H	-0.704531	0.464811	1.760274
H	1.311273	4.589886	-2.062357
H	1.640692	3.827622	2.786014
H	2.139374	5.500051	3.107865
H	0.417442	5.098823	3.005779
H	1.973732	7.234495	-2.836163
H	2.416161	9.468550	-1.834145
H	2.439997	9.781311	0.623937
H	2.007093	7.874143	2.154528
H	-1.224746	-2.953851	0.150081
H	-2.220922	-0.026269	-0.240373
H	6.706372	-1.671665	0.519054
H	5.430754	-0.809620	-0.390732
H	5.243512	-1.098527	1.370651
Cl	-8.213548	1.251585	-0.567825

Cy5-AA S_1min(ET) in toluene @ M06-2X/def2-SVP, SMD(toluene)

0 1

C	1.184573	-1.257471	-0.000532
C	2.566546	-1.439097	-0.027538
C	3.540341	-0.399957	-0.076608
C	6.791031	-1.753038	-0.084049
C	7.121968	-0.391625	-0.134529
O	-1.727906	-4.459206	0.113223
C	-2.884352	-5.194645	0.108014
C	-3.977573	-4.323034	0.081344
N	-3.436951	-3.043367	0.074936
C	-4.147719	-1.785265	0.045369
C	-3.019242	-6.568295	0.122807
C	-4.332730	-7.058893	0.107692
C	-5.429900	-6.192028	0.076894
C	-5.284960	-4.798993	0.062420
C	7.738759	-2.753173	-0.061259
C	9.083578	-2.346530	-0.089775
C	9.426024	-0.996299	-0.138014
C	8.450851	0.012478	-0.161164
C	0.318554	0.770589	-1.214663
C	-0.281248	2.013475	-1.215491
C	-0.556786	2.678624	0.006144
C	-0.190914	2.051764	1.221721
C	0.387335	0.796226	1.210911
C	-1.236641	3.943215	-0.001528
N	-1.976803	4.356527	-1.060396
C	-2.513704	5.601967	-0.817447
C	-2.059218	6.020683	0.465799

C	-1.258653	4.990502	0.998120
C	-0.547008	5.030398	2.301533
C	-3.353686	6.384448	-1.598974
C	-3.729301	7.619455	-1.072070
C	-3.283578	8.069185	0.193425
C	-2.452655	7.288121	0.968001
C	0.244942	-2.298969	0.059611
C	-1.158142	-2.120437	0.074649
C	-2.075246	-3.146729	0.090421
C	4.885631	-0.630368	-0.095453
C	0.636171	0.125984	-0.001278
O	5.432420	-1.882009	-0.062375
N	5.917513	0.289523	-0.145698
C	5.711808	1.711139	-0.201805
H	2.929155	-2.470326	-0.012143
H	3.227702	0.645115	-0.099574
H	-5.239460	-1.959704	-0.003875
H	-3.912519	-1.202291	0.949419
H	-3.841402	-1.209043	-0.841499
H	-2.148963	-7.223637	0.143486
H	-4.497306	-8.137132	0.117916
H	-6.437742	-6.609829	0.061622
H	-6.142430	-4.110993	0.025912
H	7.448293	-3.802599	-0.022291
H	9.868938	-3.102616	-0.072667
H	10.478798	-0.711548	-0.157705
H	8.728351	1.065868	-0.195978
H	0.533265	0.260220	-2.154588
H	-0.518987	2.491176	-2.167991

H	-0.419891	2.525703	2.173980
H	0.628418	0.294021	2.148769
H	-2.204303	3.778868	-1.862320
H	0.470333	4.625204	2.220182
H	-0.486797	6.062320	2.667879
H	-1.083953	4.440020	3.061975
H	-3.703447	6.052526	-2.575963
H	-4.387852	8.261904	-1.657876
H	-3.608502	9.044790	0.553627
H	-2.109499	7.626101	1.946508
H	0.634559	-3.320667	0.075001
H	-1.585481	-1.118191	0.057703
H	6.684101	2.210904	-0.275290
H	5.109497	1.978998	-1.083708
H	5.196234	2.067679	0.703666
Cl	-7.568274	-2.168373	-0.201709
